# A Proline-Based Tectons and Supramolecular Synthons for Drug Design 2.0: A Case Study of ACEI

**DOI:** 10.3390/ph13110338

**Published:** 2020-10-24

**Authors:** Joanna Bojarska, Milan Remko, Martin Breza, Izabela Madura, Andrzej Fruziński, Wojciech M. Wolf

**Affiliations:** 1 Faculty of Chemistry, Institute of General and Ecological Chemistry, Lodz University of Technology, Żeromskiego 116, 90-924 Lodz, Poland; andrzej.fruziński@p.lodz.pl (A.F.); wojciech.wolf@p.lodz.pl (W.M.W.); 2 Remedika, Luzna 9, 85104 Bratislava, Slovakia; milan.remko@gmail.com; 3 Department of Physical Chemistry, Slovak Technical University, Radlinskeho 9, SK-81237 Bratislava, Slovakia; martin.breza@stuba.sk; 4 Faculty of Chemistry, Warsaw University of Technology, Noakowskiego 3, 00-664 Warsaw, Poland; izabela@ch.pw.edu.pl

**Keywords:** proline, supramolecular synthon engineering, noncovalent interactions, drug design and discovery, ACEI, medicinal applications, coronavirus (COVID-19)

## Abstract

Proline is a unique, endogenous amino acid, prevalent in proteins and essential for living organisms. It is appreciated as a tecton for the rational design of new bio-active substances. Herein, we present a short overview of the subject. We analyzed 2366 proline-derived structures deposited in the Cambridge Structure Database, with emphasis on the angiotensin-converting enzyme inhibitors. The latter are the first-line antihypertensive and cardiological drugs. Their side effects prompt a search for improved pharmaceuticals. Characterization of tectons (molecular building blocks) and the resulting supramolecular synthons (patterns of intermolecular interactions) involving proline derivatives, as presented in this study, may be useful for in silico molecular docking and macromolecular modeling studies. The DFT, Hirshfeld surface and energy framework methods gave considerable insight into the nature of close inter-contacts and supramolecular topology. Substituents of proline entity are important for the formation and cooperation of synthons. Tectonic subunits contain proline moieties characterized by diverse ionization states: -N and -COOH(-COO^−^), -N^+^ and -COOH(-COO^−^), -NH and -COOH(-COO^−^), -NH^+^ and -COOH(-COO^−^), and -NH_2_^+^ and -COOH(-COO^−^). Furthermore, pharmacological profiles of ACE inhibitors and their impurities were determined via an in silico approach. The above data were used to develop comprehensive classification, which may be useful in further drug design studies.

## 1. Introduction

Proline was isolated for the first time by Richard Willstätter in 1900 [1], but its name was coined by Emil Fisher [2] who derived it from its pyrrolidine ring. Proline is an untypical and multifunctional endogenous amino acid which is a key constituent of almost all proteins. Nowadays, it is gaining relevance in diverse medical applications and drug design studies. In this context, key issues are being discussed, briefly summarizing either the most important or the most up to date findings across the world literature.

### 1.1. Proline as Unique Amino Acid: Conformational Inclinations

Proline is an exclusive cyclic, natural amino acid with the side-chain cyclized onto the backbone *N*-atom. Its ring, constrained by the tertiary amide bond, is a remarkably rigid structure in the world of peptides [3,4]. Proline adopts two distinct conformations governed by the cis–trans amide bond isomerism and additionally extended by the exo/endo ring puckers. Both cis and trans isomers are almost energetically equivalent [5]. Conformation of the main peptide chain is described by three dihedral angles, φ (phi), ψ (psi) and ω (omega). The former two are responsible for the chain fold and were defined by Gopalasamudram Narayanan Ramachandran [6], while the third is usually restricted to a relatively straight angle by the conjugation of the carbonyl group with the nitrogen lone pair. The ω angle adopts values close to 0 or 180° and is correlated with the cis or trans conformation of the peptide bond [6,7]. The pyrrolidine ring usually exists in half-chair or envelope conformations [8]. They may be conveniently described by two pseudo-rotation parameters, as defined by Altona and Sundaralingam, or the exo/endo puckers [9]. The latter (also known as up and down puckering states) [10,11,12] depend on the direction of the Cγ-atom towards the ring plane with respect to the orientation of the carboxylic group [13]. The Cγ-endo is placed out of the ring basal plane at the same site as the pivotal carboxylate C1 atom, while Cγ-exo is located at the opposite side of that plane [4,13,14,15]. The exo pucker describes more a compact, folded conformation, while the endo pucker is referred to as an extended architecture. Peptide bonds involving proline residue may adopt either a trans or cis arrangement, which further affects ring conformations. In trans proline, both exo and endo forms are isoenergetic, while for cis proline, the endo form is more favorable. Either cis-trans or endo/exo states can be modulated by steric and stereochemical properties of substituents [16]. Hence, the proline ring defines the properties of the proline residue as a peptide building block and substantially controls oligopeptide and protein folding. In the case of trans proline, both exo and endo forms are isoenergetic. For cis proline, the endo form is more popular [4,12,16,17,18]. It is also worth noting that substitutions on the pyrrolidine ring affect additional steric and stereochemical effects modulating the cis/trans and endo/exo states [16]. 

To conclude, the characterization of the proline ring pucker can control all protein backbone dihedral angles [4,18,19,20,21].

### 1.2. Chirality of Proline

In nature, proline exists as an *L*-enantiomer. The substituted *L* and *D* proline entity has attracted much attention as a constituent in numerous therapeutic agents or chiral ligands, such as antibiotics, anti-hepatitis, antidiabetic, antihypertensive, antiarrhythmic, antirheumatic, antiprotozoal, analgesic, antibacterial, against influenza, etc. (e.g., dalfopristin, idasanutlin, miridesap, ledipasvir, saxagliptin, telaprevir, clindamycin, dalfoprostin, miridesap, rotigaptide, anatoxin A, nargenicin A, indanomycin) [22,23,24,25,26]. In the case of numerous drugs, their pharmacodynamic, pharmacokinetic, and toxicological properties may depend on enantiomeric purity [27]. This is a key issue in contemporary pharmacology and is further accentuated by the correlation of a cofactor’s chirality with its binding affinities [28]. Therefore, the synthesis of optically active proline derivatives is of primary medical interest. While almost all residues in natural peptides are *L*-amino acids, peptides containing *D*-amino acids, such as muscoride A, trapoxin B, chlamydocin or apicidin B, were also identified over the years [29,30,31,32,33]. Likewise, *D*-proline is involved in biological processes. Therefore, it is considered an interesting selective enzyme inhibitor [22].

### 1.3. Zwiterion vs. Neutral Form of Proline

In mammalian body fluids, *L*-proline exists mostly in the zwitterionic form [34] with the protonated NH_2_^+^ and the deprotonated COO^−^ groups. It is conformationally rigid and highly soluble in water and polar organic solvents. It is noteworthy that either *L*- or *D*-proline is the most popular substance among common amino acids that facilitate the formation of cocrystals [35,36]. Therefore, it is used to modulate the physicochemical properties of several drugs applied in the treatment of different diseases, including cancer [36,37,38,39,40,41,42,43,44]. Interestingly, the modification of proline to hydroxyproline prompts a decrease in its solubility [45]. The latest findings revealed that proline can have an “important scientific basis in regulating properties in vitro and in vivo of drugs and has potential as an auxiliary agent in the treatment of joint diseases” [46].

### 1.4. Proline Cis-Trans Isomerization 

The influence of proline isomerization on the protein structure was recognized by Tanford and Ramachandran as early as in 1968 [47]. This issue was further investigated inter alia by Brandts [48,49,50] and Stewart and further MacArthur identified and characterized cis proline residues in protein crystal structures as deposited in the PDB [51,52], while Cornelius Frommel predicted the existence of cis-proline in protein chains from their primary structures [53]. The increasing abundance of high-resolution protein X-ray single-crystal data in line with advanced in silico simulated annealing studies help to detect and characterize cis-proline residues and describe their irreversible trans-to-cis flip transformations [54,55,56,57]. The majority of peptide bonds adopt a more stable trans conformation [12,47,58]. Proline is an exception. Due to the steric hindrance of the pyrrolidine ring, it has one of the lowest configurational entropy among naturally occurring amino acids [51,59,60]. The cis-non-proline residues are observed, but they are detected almost 1000 times less frequently than their trans counterparts [61]. Proline is four times less likely to adopt cis conformation than the trans residue. Trans proline has been identified in all protein secondary structure motifs, while the cis form stabilizes the bend and turn fragments [54]. The cis-trans isomerization is catalyzed by the peptidyl–prolyl cis-trans isomerases [2,62,63]. It facilitates the conformational switch in proteins which are responsible for bioprocesses regulation [64]. As pointed out by Shinoda et al. [65], this cis–trans interconversion may be intrinsically slow, with time ranging from seconds to minutes [65]. Proline isomerization is a key element in controlling the rate of protein folding and unfolding [66,67], determination of the conformational stability and aggregation [68], protein splicing, and cell signaling [69], triggering receptor-mediated transmembrane signaling, activation of peptide hormones and ligand binding [3,66,70,71,72,73,74], ion channel gating and gene expression [75,76,77,78], direct conformer-specific ligand recognition [79], open/close flaps over active sites [80], etc. Dysfunctions in proline isomerization can lead to AIDS, cancer, or neurodegenerative disorders like Alzheimer’s disease [81,82,83,84,85,86,87]. The diverse active site binding affinities of cis and trans isomers should be considered during the design of new therapeutics, with ACE inhibitors being a good example [63,88,89,90]. In particular, lisinopril exists as a mixture of cis–trans isomers in solution. However, only its *trans*-state is biologically active [91,92].

### 1.5. Further Insight into the Biological and Medicinal Relevance of Proline

Proline plays diverse functions in bio-processes. It is crucial for protein synthesis and cell growth [23]. It has relevance inter alia in cellular bioenergetics [93], redox signaling [94], osmoregulation [95], stress protection and metabolic reprogramming [23,96] etc. Proline metabolism is a promising target for an adjunctive cancer therapy [96,97].

Peptides rich in proline molecules have unique cell-penetrating features [98] and either antioxidant or antimicrobial bioactivity [26,99]. They enter the blood circulatory system faster and are resistant to digestion in intestines [100]. 

### 1.6. Inhibitory Activity of Proline

Nowadays, either the *L*- or *D*-proline receives continuously growing attention where the design and development of enzyme inhibitors are concerned [22,101,102,103]. In particular, enzyme inhibitors containing proline moiety are used in the treatment of several disorders, namely cancer, cardiovascular diseases, AIDS, HIV or other virus infections [104]. They are cofactors of numerous proteins of which metallo-beta-lactamases, matrix metalloproteases, histone deacetylase, cyclooxygenase-2 (COX-2), blood coagulation factor XIa, flaviviral [22], Zika and dengue virus proteases [103] and cholinesterase [105] are mostly appreciated. Recently, proline-based cyclic 2,5-diketopiperazines (DKP) have been recognized as promising of *α,β*-tubulin inhibitors [106]. It is worth mentioning that DKP, which has attracted attention due to its unique features (rigidity) and various bio-applications [107,108,109], is also a good candidate in anti-Alzheimer drugs [110] and is used in the synthesis of bio-active substances or peptidomimetics [111,112,113] among others. Proline-based DKPs are useful as a scaffold for building small compound libraries. It has been thoroughly documented that proline is the crucial amino acid binding to angiotensin-converting enzyme (ACE) [114,115,116]. In particular, proline at the *C*-terminal of the oligopeptide inhibitor improves stability and bioavailability, further enhancing its binding to ACE [117,118]. It supports the formation of H-bonds, facilitates either stacking or hydrophobic interactions and adjusts the carbonyl group towards the zinc cation in the active site [118,119]. The important role of proline in the contemporary world of enzymes was recently summarized by Lenci and Trabocchi [22], who pointed out that “proline is a template that can direct appendages into specific clefts of the enzyme binding site”.

### 1.7. ACE Inhibitors: Recent Updates

According to the WHO, the leading cause of death in the world is cardiovascular diseases, with hypertension being one of the major chronic disorders that lead to cardiac dysfunctions [120,121,122,123]. Proline-derived ACEI are the major antihypertensives and cardiological drugs [115,124], with captopril being the first and still one of the most popular. Its design was stimulated by the structure of a nonapeptide teprotide that was isolated from the venom of snake *Bothrops jararaca* [125,126,127]. Recent studies revealed that captopril, similar to other ACEI, e.g., enalapril [128] or ramipril [129], can play a role in an effective anti-tumor therapy via the inhibition of tumor angiogenesis and promotion of anti-tumor immunity [130,131]. A further development led to the synthesis of the third-generation ACEI, with the dipeptide perindopril being the major example [132,133]. It is characterized by a very strong enzyme inhibition ability [134]. There is firm evidence that its administration, when extended over time, protects against many different cancers [135,136,137,138] and cardiovascular diseases [133,139,140,141]. In particular, perindopril is effective in congestive heart failure prevention and improves cardiac functions [142,143]. It can serve as either a neuroprotectant or antidepressant [144] and is a good potential drug for the treatment of Alzheimer’s disease. It also offers renal protection [143]. In clinical practice, perindopril is used as *tert*-butylamine (erbumine) or arginine salts [140,145]. Like almost all ACEIs (apart from lisinopril), it is a prodrug that metabolizes in the liver to diacid perindoprilat through the hydrolysis of the ester group [146,147,148,149]. It may undergo internal cyclization to piperazinedione derivatives (diketopiperazine, DKP) or isomerization at chiral carbon centers further followed by oxidation or glucuronidation [150]. The latter reactions lead to impurities and are good examples of instabilities that plague contemporary ACEI [150]. The medical use of ACEI is limited by numerous side effects (cough, edema, diarrhea, fatigue, dizziness, headache, and erectile dysfunction) [151] which depend on physicochemical (e.g., acidity, liphophilicity, solubility) and pharmacokinetic features with the ability to penetrate the cell [152]. Nowadays, ACEI are well placed in the medical market. However, their side effects and vulnerability make them ideal targets for further developments.

### 1.8. ACEI vs. Coronavirus Studies

Currently, the global pandemic has captured the attention of scientists from all over the world. The identification of efficient therapeutic agents against Coronavirus is of the highest importance, particularly the drugs that could stop the virus from entering cells via binding to ACE2. Recently, Kim et al. [153] discovered that ACEIs have very high binding scores and may be considered successful ACE2 inhibitors. Notably, the COVID-19 virus uses ACE2 as the main cellular receptor to enter the cell. On the other hand, ACE inhibitors bind to ACE1, which catalyzes the conversion of angiotensin I to angiotensin II and thereby block the renin-angiotensin system. Unfortunately, there is no firm evidence of ACEI binding to ACE2. The latter lacks the carboxypeptidase activity of ACE1, but it has a zinc-binding domain and displays pronounced metallopeptidase activity with ~40% homology with ACE1 [153,154,155].

Moreover, peptide drugs containing proline residue are recognized as potential ligands that can combat the coronavirus over a relatively short time [156].

### 1.9. Supramolecular Synthon Engineering for the Design of Drugs 2.0

The discovery of new drugs that inhibit an enzyme activity in a selective way and lead to therapeutic benefits is a difficult and complex process. The efficacy of an inhibitor toward its target enzyme substantially depends on the non-covalent interactions between the inhibitor and enzyme. The latter are pivotal for rational and efficient drug design strategies. Crystal structure data are the major and most accurate source of information on the geometry of those interactions [157], which exhibit a high level of synergy with those existing in the protein environment [158]. In this respect, biological macromolecular systems are governed by the same forces as those that rule the supramolecular entities [159,160]. Hence, a thorough analysis of spacial properties as related to either amino acids or their derivatives, with a special emphasis on proline, is of particular importance. Nowadays, the supramolecular science efficiently employs the tecton-synthon approach. The definition of “supramolecular synthon” was introduced by Desiraju as “structural units within supermolecules which can be formed and/or assembled by known or conceivable synthetic operations involving intermolecular interactions” [161], while the definition of the “tecton” (in Greek, “builder, architect”) was proposed by Wuest as “molecular building blocks that have peripheral functional groups, capable of hydrogen- or halogen-bonding, joined to a molecular core; their number and their arrangement dictate the topology of the network” [162,163]. The tecton concept emphasizes the relevance of the shape and rigidity of molecular units [164]. In other words, tectons are building blocks of any supramolecular assembly, while synthons are related to particular patterns of intermolecular interactions linking the building blocks together [165]. This simplifies the design of supramolecular entities of predicted properties for biological applications. Either a proline residue or its pyrrolidine ring is a useful moiety for the formation of molecular and supramolecular scaffolds [22,166].

In this work, we focus on proline-based tectons and, stemming from them, supramolecular synthons for biomedical applications. It continues in our ongoing studies on the supramolecular chemistry of amino acids and short peptides [158,167,168,169,170,171,172,173]. In this respect, a comprehensive search in the Cambridge Structure Database (CSD, Version 5.41, last update May 2020) [157] aimed at supramolecular properties of proline-based compounds was carried out. Next, the proline tectons’ impact on the hierarchy of supramolecular synthons in crystal structures of all forms of ACEI was investigated. This yielded a library of relevant supramolecular motifs, which was further employed throughout this article. This approach follows the increasing interest in the design and synthesis of modern, selective idealized bio-ligands, as pointed out by Spackman et al. [174] via ”adding synthon-based functional groups in the model ligands for effective binding and utilize these models for screening from given libraries”.

## 2. Results and Discussion

### 2.1. Molecular Focus on Proline Tectons: Database Story

Proline moiety containing amine and carboxylic functional groups can serve as a supramolecular building block for a rational design of new drugs. Herein, we summarized major proline-based tectons. 

**Scheme 1:**
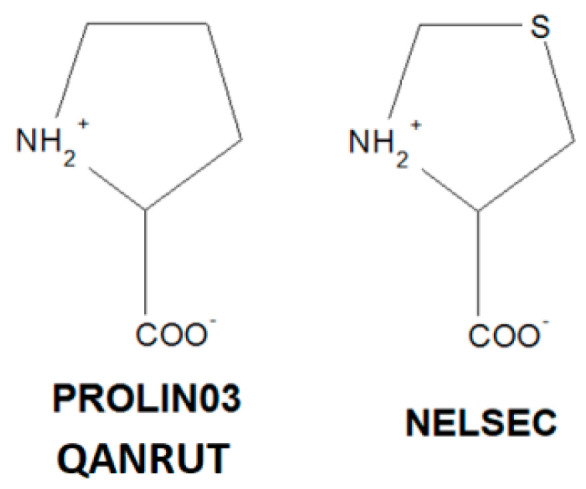
Chemical structure of proline and its analogue.

A survey of the Cambridge Structural Database (CSD, Version 5.41, last update May 2020) [157] revealed 2366 proline-based structures, while in the Research Collaboratory for Structural Bioinformatics Protein Data Bank (RCSB PDB) [175], 158 352 biomplexes containing proline moiety have beed deposited so far. An advanced search for proline crystal structures and its analogues in the CSD yielded *L*-proline (PROLIN03) [38], *D,L*-proline (QANRUT) [176], *R*-thioproline (NELSEC) [177], *L*-thioproline (TAZOLC02, without H-atoms) [178]. In addition, several solvated forms of proline were found: *D,L*-proline monohydrate (DLPROM02) [179], *L*-proline monohydrate (RUWGEV) [180], *D,L*-proline chloroform solvate (WERMIQ) [181]. Besides, 4-hydroxy-*L*-proline (HOPROL12) [182], 4-hydroxy-*R*-proline (UPIKUB) [183] can be mentioned. Notably, all of them represent the zwitterionic form, with NH_2_^+^ and COO^−^ functionalities. For the sake of simplicity, only the unsubstituted structures of proline, i.e., PROLIN03, QANRUT and NELSEC, were subjected to supramolecular analysis as presented in the next section, see Scheme 1.

**Figure 1:**
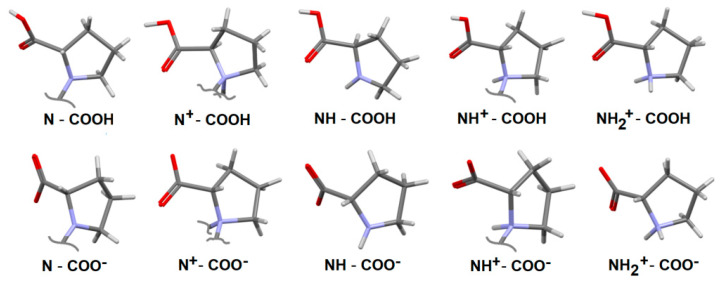
Neutral, zwitterionic, cationic and anionic forms of proline moieties as identified in 2366 relevant structures retrieved from the Cambridge Structure Database (CSD) ([157]; CSD, ver. 5.41, May 2020).

**Figure 2:**
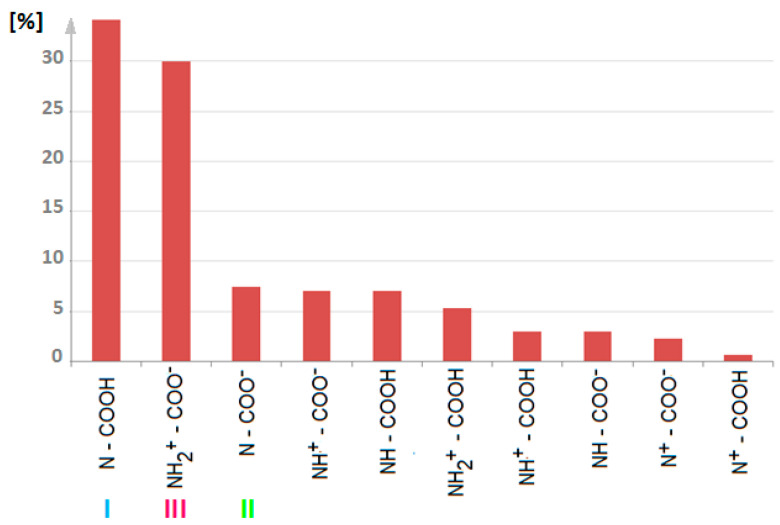
Percentage occurence of proline-based tectons in the CSD (three types of proline-based tectons, I, II, III, are color-coded, see chapter below).

A further exploration of the CSD revealed various modifications in the proline skeleton and its derivatives, which leads to both a neutral form and different ionization states (either protonation, such as cation or anion, or zwitterionic forms) of proline-based tectonic subunits containing positively and negatively charged functional groups, in particular, proline moiety with –N and –COOH(–COO^−^) groups, –N^+^ and–COOH(-COO^−^), –NH and –COOH(-COO^−^), –NH^+^ and –COOH(–COO^−^), –NH_2_^+^ and –COOH(–COO^−^), see Figure 1 (and Table S1 in Supplementary Materials). An analysis showed that zwitterionic proline tecton with NH_2_^+^ and COO^−^ groups is nearly as widespread as the neutral one. More specifically, ~34% of the entries with –N and COOH groups, ~30% of those with NH_2_^+^ and COO^−^ functionalities, ~7% occurrence of hits with –N and COO^−^, –NH and COOH, and –NH^+^ and COO^−^, whereas all the remaining motifs are below 5% (Figure 2). The ionization state of proline moieties affected the supramolecular synthons involved. 

### 2.2. Further Insight into the ACEI Crystal Structures

Despite the long-term history of ACEI, their supramolecular features have not been thoroughly characterized yet. Despite the problems obtaining good quality single crystals of ACEI, several X-ray structures have been successfully determined. In particular, unsolvated and hydrated perindopril *tert*-butylamine, UZOVAH03 and IVEGIA, hydrate and DMSO solvate of its active metabolite—perindoprilat, FEFKEI and BECWIR, and either tetragonal or orthorhombic polymorphs of its impurity F, BILNAN and BILNAN01 as well as captopril disulfide, YOZTIS, have been published elsewhere [184,185,186,187,188,189,190,191]. Herein, we report a more comprehensive supramolecular characterization of all structures of proline-based ACEI deposited in the CSD. 

Additionally, the CSD contains the following structures of ACEI and their derivatives: proline-based (enalaprilat, CSD reference code: CIYNIH [192]; enalapril maleate, DIVHOF01 [193]; lisinopril monohydrate, GERXAE [194]; lisinopril, GERXEI [194]; lisinopril dihydrate, GERWUX01 [195]; sodium hemi zofenopril, TUHMUE [194]; sodium fosinopril, TUHMOY [196] and modified-proline-based ACE inhibitors with their derivatives; 1,3-dihydroxy-2-(hydroxymethyl)propan-2-aminium ramiprilate, EDALEC [197]; ramipril, QOQWAU [198]; ramiprilat methanol clathrate, FIFGEG [199]; trandolapril, IQISAE [200]; spirapril hydrochloride monohydrate, RUWBAM [201] (Schemes S1 and S2). Chemical names of all compounds are summarized in Table S2. Molecular diagrams are shown in Figures S1–S6. Crystal data are summarized in Table S3. Remarkably, all ACEI were determined in the non-centrosymmetric space groups: *P*2_1_ (structures with proline-based tecton type II) or *P*2_1_2_1_2_1_ (structures with proline-based tecton type I). The only exception was the tetragonal (*P*4_1_2_1_2_1_) DKP perindopril derivative, known also as impurity F (BILNAN01). The two most popular proline-based ACEI tectons contain an endocyclic nitrogen atom augmented by carboxyl or unprotonated carboxylate groups. Zwitterionic structures of proline represent the frequently observed tecton of type III (Table 1). General conformational features of proline are presented in Scheme S3. Puckering parameters [202] are summarized in Table S4, while conformations of COOH/COO^−^ groups are presented in Table S5.

### 2.3. DFT Study

In silico geometry, optimization of proline molecules involved in all three types of tectons (proline structure-PROLIN03, perindoprilat-FEFKEI, perindopril-IVEGIA), was carried out.

**Figure 3:**
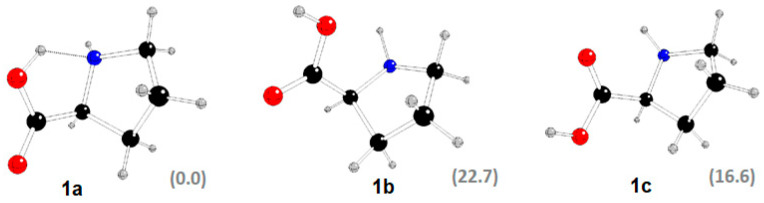
Optimized geometry of *L*-proline, structure **1a, b** and **c** with corresponding relative Gibbs free energies at 298 K (in kJ/mol) in parentheses (*C*—black, *N*—blue, *O* – red, *H*—grey).

The X-ray structure of *L*-proline PROLIN03 **1** has two hydrogen atoms located at the N1 atom (see Figure S1 for atom notation) due to two inter-molecular O^…^H-N hydrogen bonds [38]. During the geometry optimization of its single molecule in an aqueous solution, one N-bonded H atom is moved to the O1 atom with O1-H^…^N1 hydrogen bonding—structure **1a** (Table S6, Figure 3). Other stable structures with the H atom bonded to O1 **1b** or O2 **1c** without any hydrogen bonding were found as well. N1 atomic charges are more negative than the oxygen ones, whereas the charge of hydrogen atoms bonded to N1 is less positive than that of the oxygen-bonded ones. Structure **1a** is the most stable, in agreement with a similar study of Schmidt and Kass [203].

**Figure 4:**
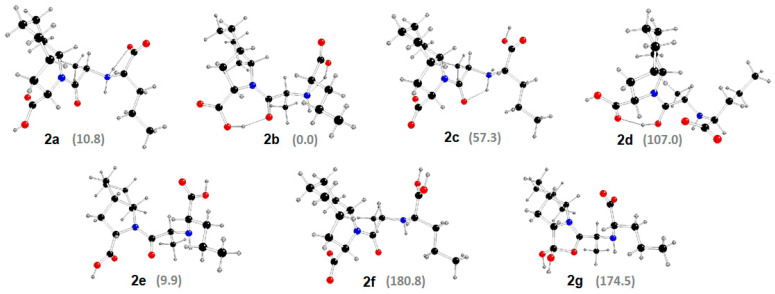
Optimized geometry of FEFKEI, structure **2** with corresponding relative Gibbs free energies at 298 K (in kJ/mol) in parentheses (see Figure 3 for atom notations).

FEFKEI **2** contains five oxygen sites and two nitrogen sites, where three hydrogen atoms might be located (see Figure S3 for atom notation). O2 and O3 sites of carboxyl groups are nearly equivalent from this point of view and the same holds for the O4 and O5 ones. The protonation at the N1 site is very improbable. At least one hydrogen must be bonded to the N2 site. These suppositions significantly reduce the number of possible protonation isomers (Figure 4, Table S7). The X-ray structure of FEFKEI **2** has one H atom bonded to the O5 site and two H atoms bonded to the N2 site due to single intermolecular N2-H^…^O hydrogen bonding with a H_2_O molecule [184]. Its geometry optimization without this molecule leads to **2a** with N2-H^…^O2 hydrogen bonding. The most stable isomer **2b** has two hydrogen bonds: O2-H^…^N2 and O4-H^…^O1. Tetravalent nitrogen with N2-H^…^O1 hydrogend bonding and a protonated O2 site in the **2c** isomer confirm that H atoms prefer ammonium N2 site over the carbonyl O1 one. On the other hand, the **2d** isomer with O1-H^…^O4 hydrogen bonding points to the H preference of the carbonyl O1 site over the carboxyl O4 one if the neighbouring O5 site is protonated. Different carboxyl protonation at O2 and O5 sites leads to a significant energy decrease in the **2e** isomer, and only the absence of hydrogen bonds causes this isomer to not be the most stable one. The protonation of both oxygen sites in the same carboxyl group (O2 and O3 in **2f** isomer, O4 and O5 in **2g** isomer) causes a significant energy increase. Moreover, an additional O1-C15 bond is formed in the **2g** isomer. N1 atoms are always less negative than the N2 ones and these are less negative than the oxygen ones. The heteroatom protonation increases its negative charge. The protonation of a single carboxyl oxygen atom increases the negative charge of both oxygen atoms in the same carboxyl group.

**Figure 5:**
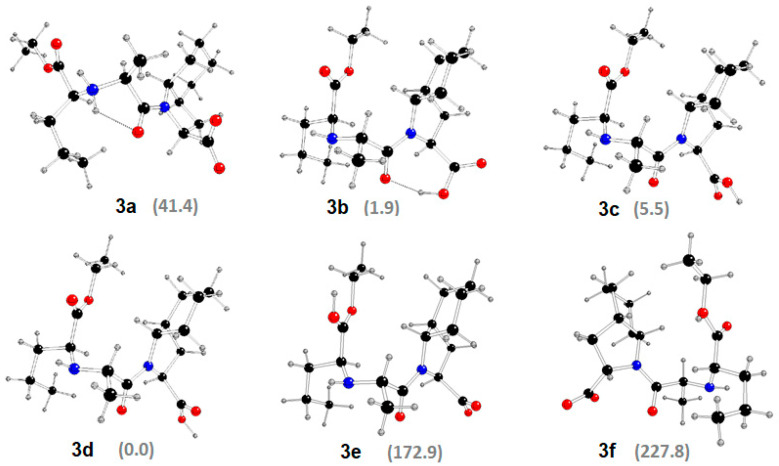
Optimized geometry of protonated IVEGIA, structure **3** with corresponding relative Gibbs free energies at 298 K (in kJ/mol) in parentheses (see Figure 3 for atom notations).

The X-ray structure of IVEGIA contains its anions compensated by *tert*-butylamonium cations [189] (Figure S4). If we suppose that the N1 site protonation is highly improbable and ought to lead to the N1-C9 bond break, the N2 site must be bonded to at least one hydrogen, thus the number of protonation sites to obtain a neutral IVEGIA molecule **3** is significantly reduced (Table S8, Figure 5). Protonation at the O1 site leads to H moving to N2 (isomer **3a**) or O4 sites (isomer **3b**) with O1^…^H-N2 or O1^…^H-O4 hydrogen bonding, respectively. The protonation at carboxyl O4 (isomer **3c**) or O5 sites (isomer **3d**) leads to practically equal structures. Only slight energy differences between the most stable 3**d** and **3b-c** structures may be observed. The protonation at the carboxyl O2 site (isomer **3e**) is highly improbable due to high energy demands, and the same holds for the carboxyl O3 site (isomer **3f**). N1 atoms are always less negative than the N2 ones. The heteroatom protonation increases its negative charge. 

In view of the above, a detailed analysis of supramolecular interactions participating in the synthon patterns’ formation is justified and needed for the design and development of new drugs.

### 2.4. Survey of Supramolecular Interactions in ACEI Crystal Structures

**Figure 6:**
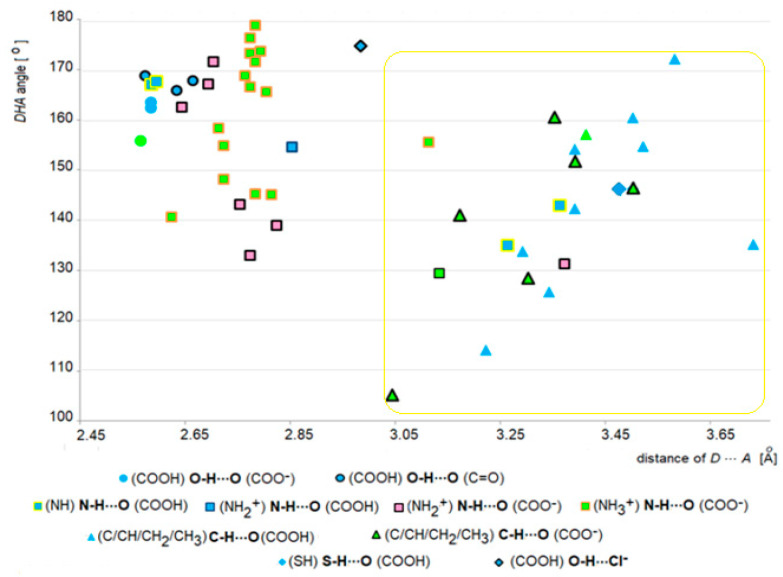
Diagram of the H-bond distance (Donor^…^Acceptor) and bond angle (Donor-Hydrogen^…^Acceptor) in proline and ACEI structures (19 structures; reference codes are included in the Table 1). In the perindopril-derived crystals, _(OH_COOH)_O-H^…^O_(COO-)_, _(NH2+)_N-H^…^O_(COO-)_ and _(NH3+)_N-H^…^O_(COO-)_ and _(C-)_C-H^…^O_(COOH)_ interactions are observed (colors of fulfilment are consistent with the three types of proline-based tectons observed in the crystals of proline and ACEI: blue-I, green-II, pink-III; weak interactions are marked by the yellow frame).

The lengths and angles of H-bonds in intermolecular interactions resulting from the three types of proline-based tectons have been compared (Figure 6). In particular, related geometrical parameters in O-H^…^O, N-H^…^O and C-H^…^O are observed in the range of: 2.58–2.67 Ǻ and 156–169°, 2.59–3.38 Ǻ and 129.4–179.1°, 3.05–3.59 Ǻ and 114–172.4°, respectively. More specifically, these interactions were divided in terms of functional groups. In particular, in the case of O-H^…^O: COOH^….^COO^−^ and COOH^…^C=O, for N-H^…^O: NH^…^COOH, NH_2_^+…^COOH, NH_2_^+…^COO^−^ and NH_3_^+…^COO^−^, for C-H^…^O: COOH^…^C(CH/CH_2_/CH_3_) and C(CH/CH_2_/CH_3_)^…^COO^−^. Among these three subtypes, the following order is observed: _COOH_O-H^…^O_COO-_ (2.57–2.59 Ǻ and 156–164°) < _COOH_O-H^…^O_C=O_ (2.58–2.67 Ǻ and 166–169°) < _NH_N-H^…^O_COOH_ (2.59–3.37 Ǻ and 135–167.8°) < _NH2+_ N-H^…^O_COO-_ (2,65–3,38 Ǻ and 129.4–171.8°) {_NH3+_N-H^…^O_COO-_ (2.63–3.12 Ǻ and 140.7–179.1°) < _C/CH/CH2/CH3_C-H^…^O_COO-_ (3.05–3.51 Ǻ and 128.4–160.7°) < _COOH_C-H^…^O_C/CH/CH2/CH3_ (3.23–3.59 Ǻ and 114–172.4°. Additionally, O-H^…^Cl^−^ (2.99 Ǻ and 175°) and S-H^…^O (3.51 Ǻ and 146.2°) interactions are observed. According to Figure 6, we can elegantly differentiate between strong and weak interactions participating in the formation of supramolecular synthons.

The occurrence of interactions in three types of analysed proline and ACEI compounds is shown in Figure S7. H-bond geometries of all analysed crystal structures, geometrical parameters for the π-stacking moieties involved in the π^…^π interactions, and those for the other π-stacking moieties are summarized in Tables S9–S11, respectively.

#### 2.4.1. Hirshfeld Surface Study

**Figure 7:**
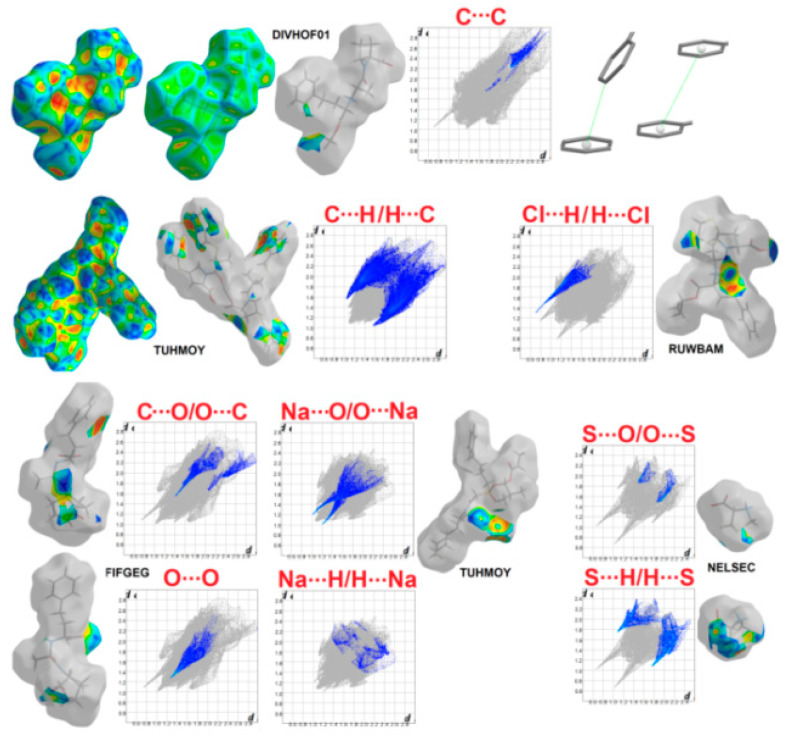
Selected 3-D Hirshfield surface (HS) maps and 2-D fingerprint plots (FPs) representing main inter-contacts in the proline-derived crystals.

The nature of close inter-contacts participating in the formation of supramolecular synthon patterns within the crystals of proline, ACE inhibitors and related compounds was extensively studied through Hirshfeld surface (HS) analysis. In this context, 3-D HS maps and 2-D fingerprint plots (FP) (Figure 7 and Figures S8–S10) were generated for the sake of qualitative and quantitative assessment, respectively. Red areas mapped on the normalized contact distance (*d_norm_*) vary in relations to the strength of the interactions. Bright red regions characterize strong inter-contacts (O-H^…^O) between the functionalities, lighter red spots reveal weaker interactions (e.g., N-H^…^O), while the least red surfaces reflect the weakest interactions in the crystal packing (C-H^…^O in BILNAN). The *d_i_* and *d_e_* disruptors characterize inner and outer distances of the Hirshfeld surface to the nearest nucleus, respectively. The adjacent red and blue triangles on the surfaces mapped over the shape index and flat surface patches below the ring plotted on the curvedness represent π^…^π contacts (e.g., DIVHOF01, Figure 7). The subtle indicators of these stacking inter-contacts are also visible on the surface of BILNAN. The fragment patches identify the coordination environments of the moieties.

**Figure 8:**
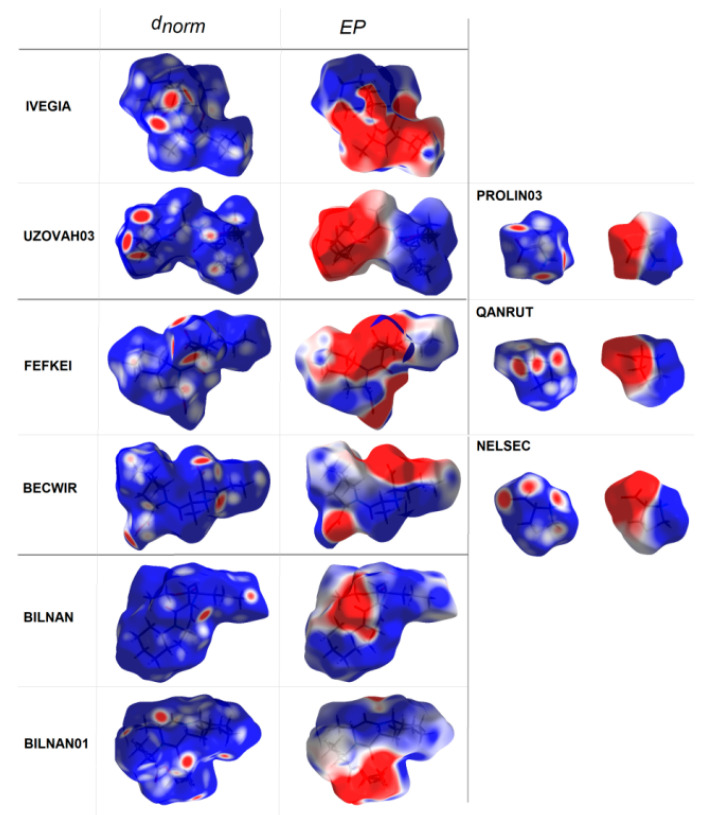
Views of the HSs mapped with *d_norm_* and EPs for perindopril-derived (on the left) and proline compounds (on the right).

Electrostatic potential (EP) provides a further direct insight into the interactions in relation to the electrostatic complementarity between neighbouring moieties within the crystal. It proves the localisations of the main close inter-contacts mapped over the *d_norm_* property. Notably, proline crystals have the nature of a dipole, with an H-bond donor (positive potential) and acceptor sites (negative potential), represented by blue and red areas, respectively. Perindopril structures and other analysed compounds containing COO^−^ groups (associated with the negative EP) are of a rather similar nature. EP over the Hirshfeld surfaces of proline, perindopril derived structures and other proline-based ACEI crystals are shown in Figure 8 and Figure S11, respectively. 

**Figure 9:**
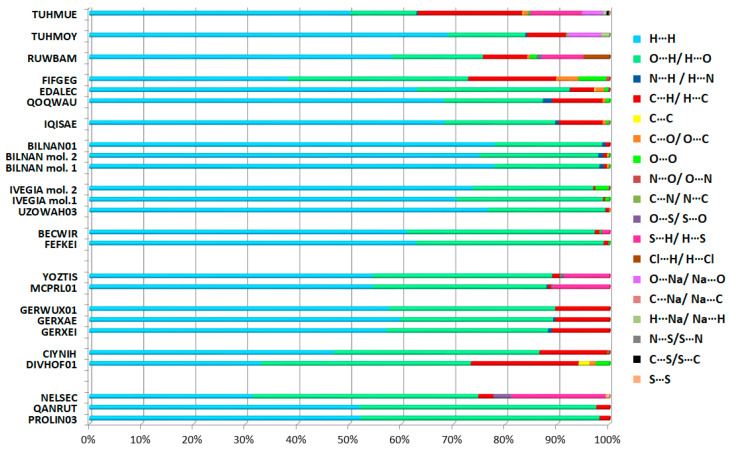
Relative contributions of the different interactions to the Hirshfeld surface for all analysed proline-derived structures.

The percentage contributions of major interactions are presented in Figure 9. The H^…^H, O^…^H/H^…^O and C^…^H/H^…^C inter-contacts are the major players, at the level over 78% (and 100% in GERWUX01). The highest share of H^….^H, O^…^H/H^…^O and C^…^H/H^…^C contacts is in BILNAN (78%), CIYNIH (39%) and DIVHOF01 (20%), respectively. S^…^H/H^…^S contacts are another significant factor, sharing total HS area at the level from 1.5% in BECWIR to ~10% in MCPRL01, TUHMUE, YOZTIS and RUWBAM. On the other hand, S^…^O/O^…^S contribute only in 1% in RUWBAM. Surprisingly, the C^…^C and N^…^H/H^…^N contacts are less visible and contribute to the overall crystal packing at 2% in DIVHOF01 and QOQWAU, respectively. C^…^O/O^…^C have relevance in DIVHOF01 and EDALEC (~1.5%) and in FIFGEG (4%). O^…^O wascfound in DIVHOF01 and RUWBAM (~2%) and in FIFGEG (5%). Cl^…^H/H^…^Cl and Na^…^O/O^…^Na are observed at the level of 5% in RUWBAM and TUHMOY, TUHMUE, respectively. In addition, Na^…^H/H^…^Na contacts exist (1.5%) in TUHMOY (Table S12).

Additionally, HS parameters are summarized in Table S13. The area-to-volume ratio, globularity or asphericity indicators show insignificant differences due to the interplay of conformations and crystal packing features among the corresponding polymorphs or solvates.

Crystal structures of ACEI are a relevant source of supramolecular information for the identification and modelling of new target-oriented biologically active ligands. 

In this context, HS analysis provides an additional value for the screening of suitable modelled ligands, which can be based on the shapes of the small molecules and their interactions with the neighbouring molecules due to their compatibility with the protein environment. The HSs and the complementarity of EPs between the ligand and the receptor pocket are important features in the thorough binding characterization. Interestingly, HSs can be also generated directly for the ligands located within the active sites. Moreover, the 3D pharmacophore models can be built following the molecular shapes and interaction surfaces (HS maps) of ligands. The H-bond donor and acceptor sites are visualized [174]. EP maps, as detailed molecular landscapes, provide subtle information, which is significant in the prediction of H-bond preferences. 

#### 2.4.2. Interaction Enrichment Ratio Analysis

The enrichment ratios (ER) of the interactions available in the analysed crystal structures were based on the HS methodology, see Section 3.2. [204]. Results of calculations are collected in Table 2, Table 3 and Table 4 and Table S14. Privileged contacts in relation to the ER are as follows:

- O^…^H/H^…^O in all cases, apart from sodium fosinopril (TUHMOY);
- C^…^H/H^…^C: in all proline structures, both perindoprilat forms (BECWIR, FEFKEI) and enalapril maleate (DIVHOF01), enalaprilat (CIYNIH), lisinopril (GERXEI), lisinopril hydrates (GERXAE, GERWUX01), captopril disulfide (YOZTIS), trandolapril (IQISAE), ramipril (QOQWAU), ramiprilat methanol solvate (FIFGEG), spirapril (RUWBAM), sodium fosinopril (TUHMOY), sodium hemi zofenopril (TUHMUE);
- S^…^H/H^…^S: in proline analog (NELSEC), perindoprilat DMSO solvate (BECWIR) and YOZTIS, RUWBAM, TUHMUE;
- N^…^H/H^…^N: DKP perindopril structure (BILNAN) and QOQWAU;
- C^…^C: DIVHOF01;
- O^…^O: perindopril form (IVEGIA);
- C^…^O/O^…^C: ramiprilat (EDALEC), RUWBAM;
- Cl^…^H/H^…^Cl and S^…^O/O^…^S: RUWBAM;
- Na^…^O/O^…^Na: TUHMOY, TUHMUE;
- H^…^H: perindoprilat DMSO solvate (BECWIR), both DKP perindopril forms [BILNAN(01)], perindopril structures and also EDALEC, GERXEI, GERWUX01, TUHMOY, TUHMUE.

The highest value of ER is calculated for the contacts O^..^H, C^…^H and S^…^H (1.61) in perindoprilat, followed by O^…^O (1.25) in perindopril and N^…^H (1.15) in DKP polymorph, among the structures of the perindopril family. The H^…^H contacts are the most abundant interactions (~70%), but they are rather moderately enriched with the ER close to unity (0.9).

In the case of other proline-based ACEI, the most enriched contact is between oxygen and sodium atoms, in TUHMOE and TUHMUE, although it represents ~5% of the contact surfaces. 

The O^…^O, C^…^O, Na^…^O, O^…^H/C^…^H, S^…^O, Cl^…^H, C^…^C, N^…^H have ER values of 2.03 (in DIVHOF01), 1.88 (in EDALEC), 1.41 (in TUHMUE), 1.38 (in CIYNIH), 1.33 (in RUWBAM), 1.29 (in RUWBAM), 1.25 (in DIVHOF01) and 1.2 (in QOQWAU), respectively.

On the other hand, weak contacts O^…^H (0.86 in TUHMOY), C^…^O (0.8 in FIFGEG), S^…^H (0.77 in MCPRL01), S^…^O (0.63 in NELSEC and 0.5 in TUHMUE), Na^…^H (0.23 in TUHMOY) and C^…^O (0.21 in DIVHOF01) are disfavoured in the crystal packing of proline-based ACEI. The two latter contacts are impoverished.

#### 2.4.3. Portfolio of Supramolecular Synthons Resulting from the Proline-Based Tectons

**Figure 10:**
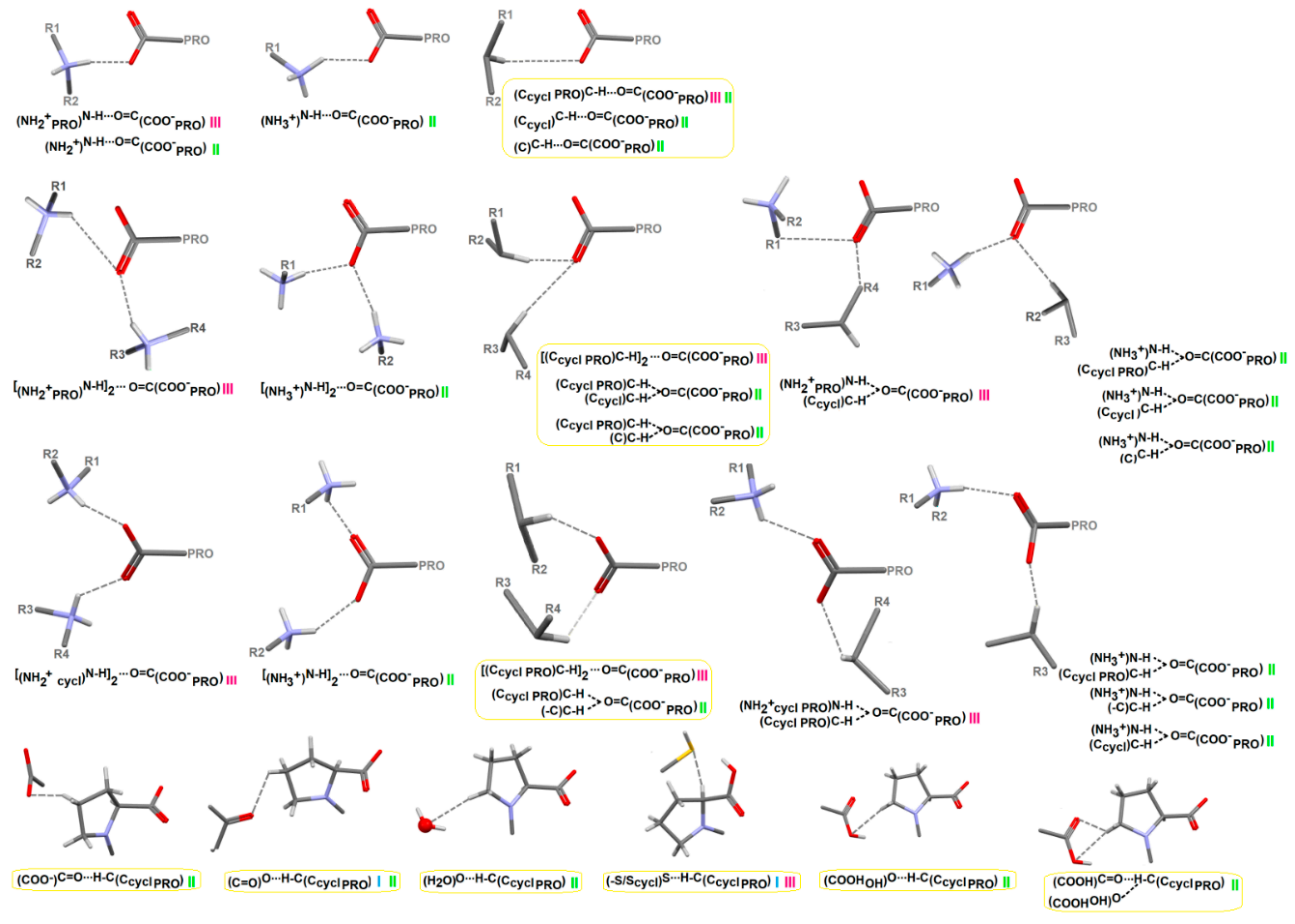

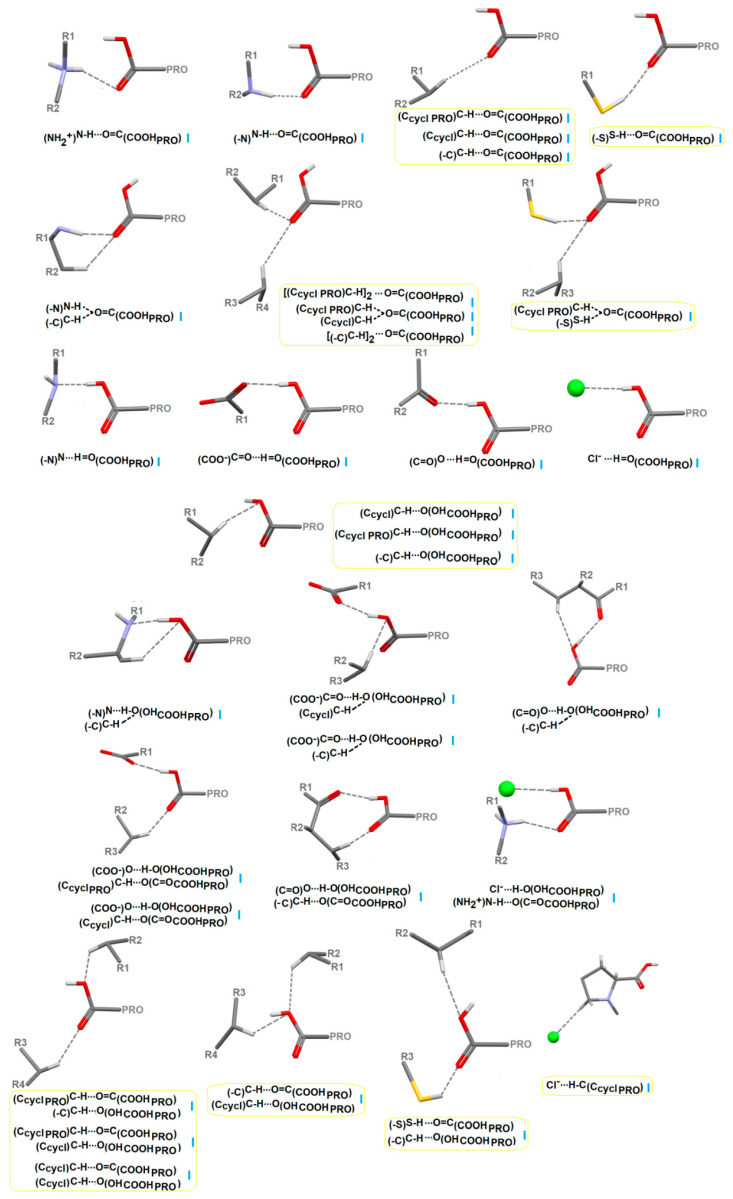
Supramolecular synthons resulting from three types of proline-based tectons observed in ACEI structures (three types of proline-tectons are color coded: I-blue, II-green, III-pink; synthons forming only by weak interactions are marked in yellow frames).

**Figure 11:**
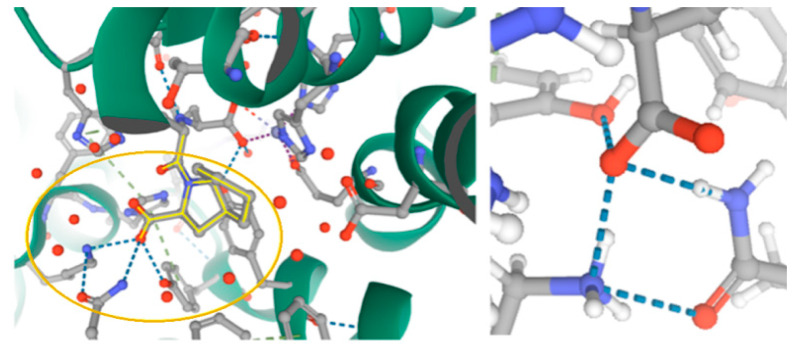
Proline-based synthons in bio-complexes of ACE with perindoprilat, 2x94.pdb (proline moiety is marked by orange colour) [205]—on the left, and fosinoprilat, 6s1z.pdb [206]—on the right.

The thorough investigation revealed a wide variety of supramolecular interactions (synthons) resulting from three types of proline-based tectons (Figure 10 and Table S15). Generally, O-H^…^O, O-H^…^N, N-H^…^O, C-H^…^O, S-H^…^O and O-H^…^Cl^−^ synthons are formed. More specifically, COOH and COO^−^ groups of proline participate in either single or bifurcated H-bonding interactions. The latter play an important role in supramolecular architectures of ACEI crystals. The COO^−^ group is involved in the N-H^…^**O**^…^H-N, C-H^…^**O**^…^H-C, N-H^…^**O**^…^H-C, while the COOH group builds two types of H-bonds systems: N-H^…^**O**^…^H-C, C-H^…^**O**^…^H-C, C-H^…^**O**^…^H-S and O^…^H-**O**^…^H-C, N^…^H-**O**^…^H-C. Moreover, the pyrrolidine ring of proline participates in the formation of _(-CcyclPRO)_C-H^…^O[S, Cl^−^]_(COOH/COO-/SH/Cl-)_ interactions. Synthons formed by weak inter-contacts only are marked in yellow frames in Figure 10. Notably, similar interactions are observed inter alia in the biocomplex of ACE with perindoprilat (2 × 94.pdb) [205] or fosinoprilat (6s1z.pdb) [206], see Figure 11. Supramolecular synthons, formed by the same interactions of proline, in relation to three types of proline-based tectons, observed in the ACEI crystal structures, are summarized in Table S16. COOH/COO^−^ groups of pyrrolidine, aliphatic groups COOH/COO^−^ and NH (terminal) have relevance in the ACE binding. Therefore, from the point of view of the bio-complexes of inhibitors with ACE, in the Supplementary Materials we included synthon patterns formed by COOH(COO^−^) of proline (Table S17), COOH(COO^−^) in the chain of proline (Table S18) and by (NH_2_^+^) in the proline chain (Table S19) in ACEI crystals, as well as the library of all observed synthon patterns in all analyzed structures (Table S20). In Figure S12, recurrent supramolecular synthons observed that result from the three types of proline-based tectons in proline and perindoril-derived structures are presented. The most common supramolecular synthon motifs are: the linear chain *C*^2^_2_(6) based on the N-H^…^O hydrogen bonds and the cyclic ring *R*^2^_2_(12) based on the C-H^…^O and N-H^…^O bonds. The same patterns exist most commonly in perindopril-derived structures.

**Figure 12:**
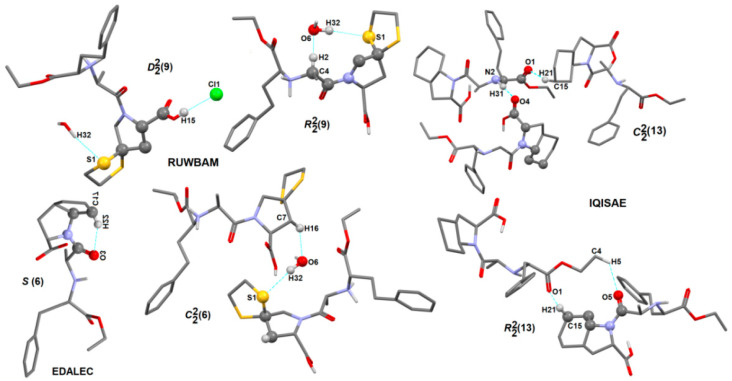
Proline-based synthon patterns in substitued proline-based ACEI.

In addition, the effects of substituents in the proline ring on the supramolecular motifs were studied. Substituents play significant roles in the formation of either linear or cyclic synthons. In RUWBAM, *C*^2^_2_(6), *R*^2^_2_(9), *D*^2^_2_(5), *D*^2^_2_(6), *D*^2^_2_(9), *D*^2^_2_(10), *D*^3^_3_(16), *D*^3^_3_(20), while in the IQISAE: *R*^2^_2_(13), *C*^1^_2_(16), *C*(11), *C*^2^_2_(13), *C*^2^_2_(15), *C*^2^_2_(19), *C*^2^_2_(20) graphs are observed. The sulphur atom participates in the formation of synthons via O–H^…^S H-bonding with water molecules. In EDALEC, intramolecular synthon *S*(6) is observed. A *Tert*-butyl moiety in perindopril crystals is also involved in the synthons (Figure 12). Furthermore, all substituted-proline-based ACEI (mainly TUHMUE, TUHMOY, RUWBAM, but also BECWIR, FEFKEI, EDALEC, IVEGIA, UZOVAH03, QOQWAU, IQISAE, FIFGEG, BILNAN with the 6-membered ring fused with pyrrolidine), cooperate in building large synthons (above 20-membered descriptors).

#### 2.4.4. LSAM: Long- Range Synthon Aufbau Modules

##### Proline Structures

**Figure 13:**
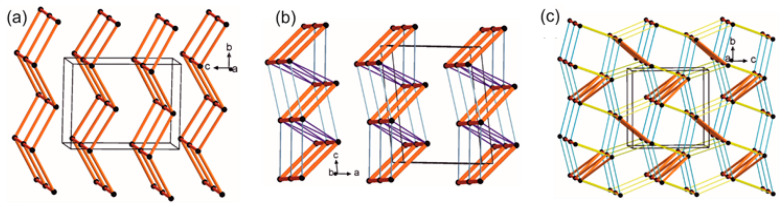
Simplified hydrogen-bonded networks showing LSAMs in proline crystals: (**a**) L-proline, (**b**) rac-proline, (**c**) L-thioproline. The black dot represents a center of gravity of the molecule, red and orange lines stand for N-H^…^O hydrogen bonds, blue and yellow lines for C-H^…^O and C-H^…^S contacts, respectively.

The analysis of the *L*-proline crystal structure PROLIN03 revealed that both N-H^…^O hydrogen bonds organize molecules into a square grid zigzag layer with alternating proline rings on both sides of this 2D entity. The layers are parallel to the (001) plane and constitute Long-Range Synthon Aufbau Modules (LSAM) [207] (Figure 13a), whereas in a racemic crystal QANRUT the same type of hydrogen bonds organize molecules into 1D ladders along (010). They are further joined by C–H^…^O contacts into a double layer parallel to the (100) plane with proline rings located on its both sides. In this case, these double layers might be regarded as the LSAMs (Figure 13b). In enantiopure thioproline NELSEC or TAZOLC02, the strongest hydrogen bonds (N-H^…^O) form zigzag zipped double chains along the (100) direction, which are next connected via C-H^…^O and C-H^…^S H-bonds into a 3D structure (Figure 13c). The main chains LSAMs are oriented in such a way that a pseudo 4_1_/4_3_ axis might be found. 

##### Proline-Based ACEI

In the enalaprilat hydrate **CIYNIH** crystal structure, the molecules are connected via O-H^…^O and N-H^…^O hydrogen bonds, forming a four-connected uninodal diamondoid network (the LSAM in this case). Water molecules are located in voids (blue spheres in Figure S13). DIVHOF01 and DIVHOF02 reference codes stand for enalapril maleate crystal structures of monoclinic and orthorhombic polymorphs, respectively. Although there are missing protons in the deposited files, a careful analysis of possible contacts revealed short distances, indicating the presence of N-H^…^O and O-H^…^O charge-assisted hydrogen bonds between cations and anions. In both polymorphs, these interactions join molecules into tubes along the (010) direction. Further, they are connected via cation-cation N-H^…^O H-bonds into undulated double layers with six-membered ring meshes. These layers constitute LSAMs in enalapril maleate crystals. In the monoclinic form, the layers are related by translation in the (001) direction, while in the orthorhombic one they are parallel to the (100) planes and related by the *2*_1_ screw axis. In GERXEI (lisinopril) crystal structure, N-H^…^O H-bonded tubes along the shortest unit cell axis *b* are observed. They represent LSAMs and are hexagonally packed (the β angle is close to 120°). Very weak N-H^…^O contacts with the carboxyl group as well as C-H^…^O and C-H^…^π interactions are present between the large synthons (thin gray lines in Figure S13). In the lisinopril monohydrate crystal structure GERXAE, molecules are joined by N-H^…^O interactions into tubes along the unit cell axis *b*. These entities can be treated as LSAMs, as they are almost hexagonally packed. Water molecules are present in voids and join the 1D tubes by numerous hydrogen bond interactions into layers on the plane (-102). Weak C-H^…^π and C-H^…^O interactions are present between the layers. In the crystal structure of lisinopril dihydrate GERWUX01, similar LSAMS are created and water molecules located in channels along the (010) direction, forming H-bonds with the molecules as well as between themselves. In the captopril crystal structure MCPRPL01, zigzag O-H^…^O chains along the (010) direction are formed, which are further joined by S-H^…^O interactions into layers on the (001) plane. The layer is undulated with a square-grid topology and constitutes a large synthon here. Weak C-H^…^O contacts join these layers into a diamondoid-like 3D structure. In the captopril disulfide crystal structure YOZTIS, a square-grid O-H^…^O hydrogen-bonded layer on the (102) plane forms LSAM. The layers are joined by C-H^…^S and C-H^…^O interactions into a 3D structure. The crystal structure of sodium fosinopril TUHMOY is dominated by ionic interactions organizing sodium cations along the shortest unit cell vector *b*. The anion–anion interactions are of C-H^…^O and C-H^…^π type and the first ones constitute a square-grid-like layer parallel to the (-101) plane. The aromatic interactions form stacks along the (010) direction. Cations are located between these layers. In the other sodium salt of proline-based ACEI (hemi zofenopril, TUHMUE) apart from ions, an electroneutral drug molecule is present. An O-H^…^O H-bonded heterodimer is formed between the anion and the molecule. The dimers are further joined by C-H^…^ π, C-H^…^O and C-H^…^S interactions into layers on the (010) plane. The cations are located between these undulated layers.

##### Perindoprilat Crystals

**Figure 14:**
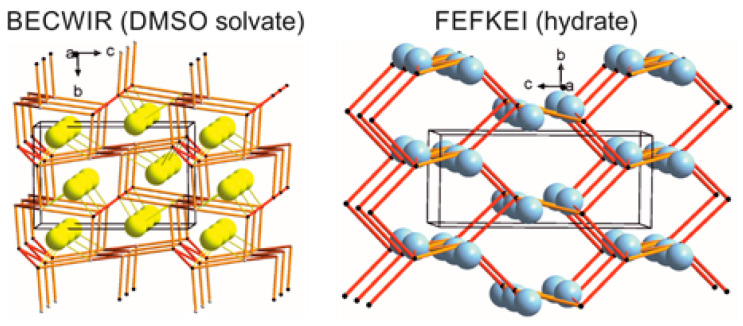
Simplified hydrogen-bonded networks showing LSAMs in perindoprilat crystals. The black and gray dots represent a center of gravity of the molecules, orange-shaded lines stand for O-H^…^O or N-H^…^O hydrogen bonds. Blue and yellow spheres represent the positions of water or DMSO molecules, respectively.

In the BECWIR crystal structure, there are two crystallographically independent molecules. Molecules of each type are individually joined into zigzag chains running along the [100] direction. Next, these chains are joined by N-H^...^O bonds, creating a 3D structure. Topologically, this framework can be simplified as an uninodal (if two molecules are considered indistinguishable) four-connected distorted diamondoid network with the point symbol of {6^6^}. The solvent molecules (dimethyl sulfoxide) are located in the channels parallel to the unit cell *a* axis (Figure 14) and they are bound to the 3D framework (an LSAM in this case) by N-H^…^O interactions (the solvent is represented by yellow spheres corresponding to the position of sulfur atoms). In FEFKEI crystals, topologically similar 3D LSAMs are observed with O-H^…^O zigzags chains along [010] interwoven by N-H^…^O zigzags along the [100] direction. In channels, along the [100] direction, water molecules (blue spheres in Figure 14) are located and interact with the framework via O-H^…^O hydrogen bonds.

##### Perindopril Erbumine Structures

**Figure 15:**
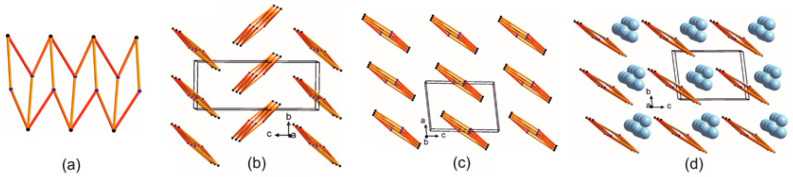
Simplified hydrogen-bonded networks in perindopril erbumine structures: a ladder LSAM (**a**); packing of LSAMs in orthorhombic (**b**) and monoclinic (**c**) polymorphs and hydrate (**d**). The black and gray dots represent a center of gravity of crystallographically independent ions, orange-shaded lines stand for O-H^…^O or N-H^…^O hydrogen bonds, water molecules are represented as blue spheres.

In perindopril erbumine polymorphs (UZOVAH and UZOVAH03) as well as its hydrate (IVEGIA), an undulated ladder formed by N-H^…^O hydrogen bonds between counter ions is observed (Figure 15a) In the orthorhombic polymorph (UZOVAH), a herringbone arrangement of the 1D LSAM, is observed, whereas in the monoclinic form the ladders align parallelly (Figure 15b,c). In the hydrate, two independent cations and anions are observed, but topologically they form an analogue to the anhydrate 1D ladder large synthon with one type of anion placed along each ladder “rail”. The ladders are parallelly oriented with disordered water molecules occupying channels between main synthons and running along the [100] direction (Figure 15d).

##### DKP Perindopril Erbumine Structures

**Figure 16:**
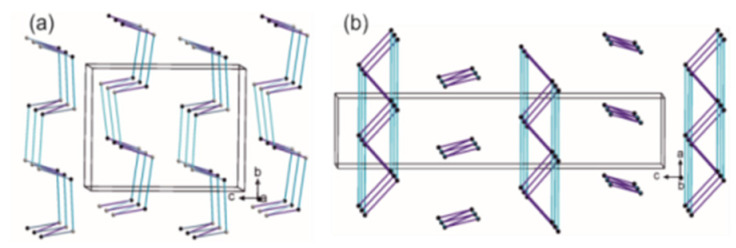
Simplified hydrogen-bonded networks in DKP perindopril erbumine orthorhombic (**a**) and tetragonal (**b**) polymorphic structures. The black and gray dots represent a center of gravity of crystallographically independent molecules, blue-shaded lines stand for weak C-H^…^O hydrogen bonds.

In the orthorhombic polymorph BILNAN two crystallographically independent molecules are bound to each other by C-H^…^O contacts only. The most meaningful interactions join molecules into honeycomb-like undulated layers parallel to the (001) planes (Figure 16), whereas, in the tetragonal polymorph BILNAN01 the molecules are also joined by relatively short C-H^…^O interactions, but in this case they arrange into zigzag ladders parallel to the *a* (and *b*) unit cell axis. These LSAMs are related by the 4_1_ screw axis.

##### Other Crystal Structures of Other Modified Proline-Based ACEI

In the EDALEC ionic crystal structure, O-H^…^O hydrogen-bonded (anion–anion) chains running parallel to the (010) direction are observed (red lines in Figure S14). The chains are joined by C-H^…^O charge assisted H-bonds (blue lines) leading to the formation of 1D tubes. Further, the cation–cation C-H^…^O interactions, forming themselves into zigzag ladders (green lines), join the tubes into layers (LSAM) on (001) planes. In the ramiprilat methanol clathrate (FIFGEG), the short O^…^O contacts between carboxylic fragments of the molecule indicate the presence of O-H^…^O hydrogen bonds (the structure is incomplete due to the disorder of the solvent). These interactions lead to the formation of zigzag chains running along the (100) direction. These motives are almost hexagonally packed and thus may be treated as LSAM. The disordered methanol molecules occupying the space between the large synthons (blue spheres in Figure S14) are in accordance with the given position of methanol oxygen atoms). In the trandolapril crystal structure (IQISAE), H-bonded zigzag chains running along the (100) direction are observed. These LSAM are pseudo-hexagonally packed but the relatively short C-H^…^O contacts join them along the chains and also into a 3D diamondoid-like structure. Similarly, in the ramipril crystal structure (QOQWAU), LSAMs are in the form of pseudo-hexagonally packed H-bonded zigzags parallel to the (100) direction. In this case, similar connections are formed by weak interactions, but along the unit cell axis *a,* N-H^…^π contacts can be found (gray lines in Figure S14), while the C-H^…^O ones lead to the 3D diamantoid-like network. In the ionic spirapril hydrochloride monohydrate crystals (RUWBAM), pseudo-hexagonally N-H^…^O H-bonded chains along (010) are observed. These LSAM are joined by the weak C-H^…^O contact into the 3D diamondoid-like structure (blue lines in Figure S14). Additionally, water molecules and chlorine anions interact with the drug and each other, leading together to the tubes in accordance with the direction of the main chains. 

The exploration of LSAM gives a thorough insight into the interactions, represented by synthons and their cooperation. These additional data, which are useful to establish synthon hierarchies, may have relevance in selecting the binding preferences vital for the design of novel inhibitors with higher specificity and efficiency. 

#### 2.4.5. Energy Frameworks on Interaction Energies

**Figure 17:**
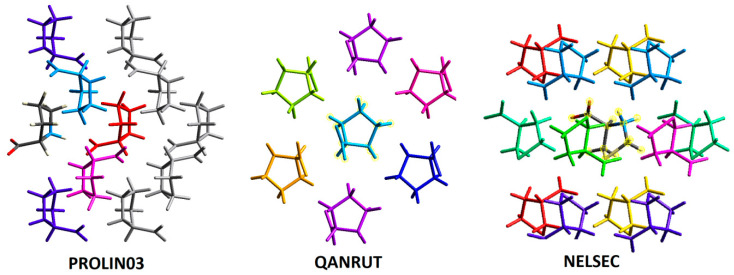
Color coding of neighbouring molecules in proline structures in relations to the central molecule. A view along the ***b***-axis (for interpretation of the references to colour in this figure legend, the readers are referred to the web version of this article).

**Figure 18:**
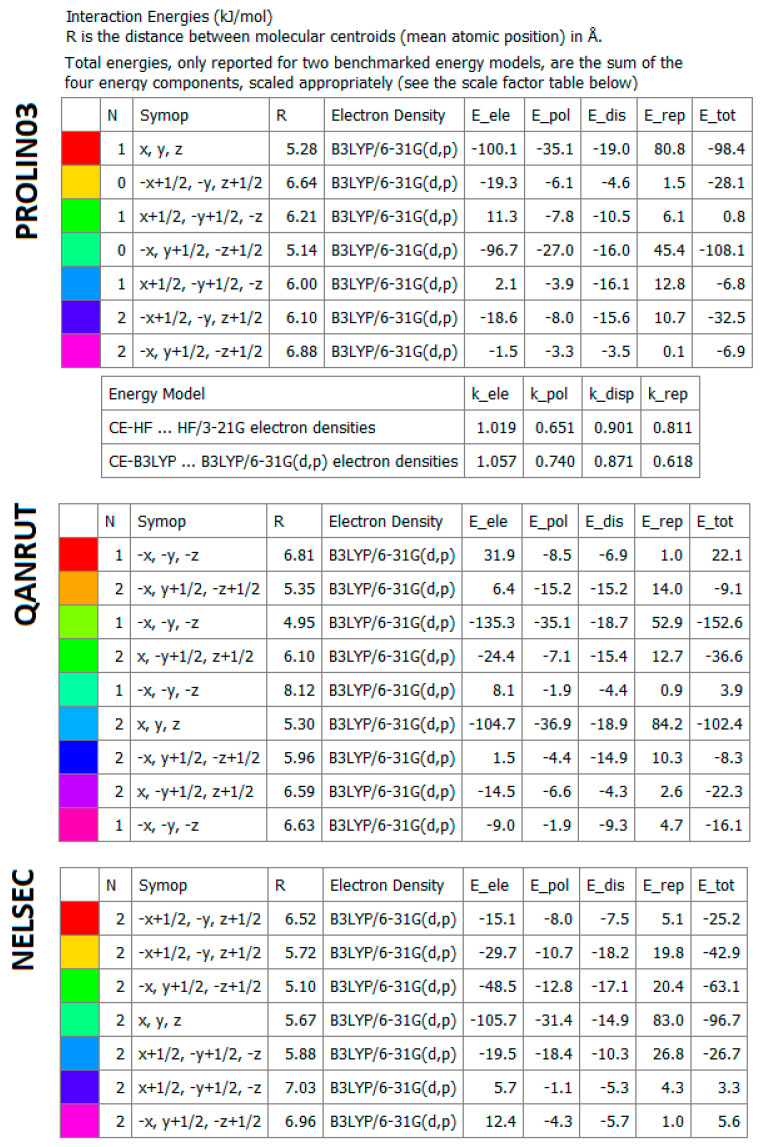
Interaction energies of the molecular pairs related to the energy frameworks of proline structures (scale factors for benchmarked energy models).

**Figure 19:**
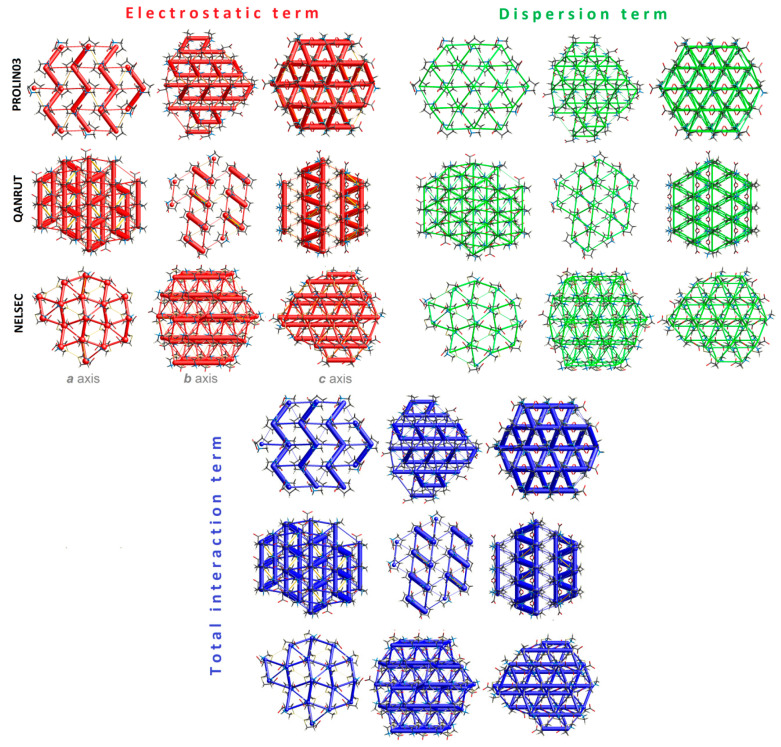
Energy frameworks of proline structures corresponding to the electrostatic and dispersion energy components and the total energy framework along *a, b* and *c*-axis (the tube size: 300).

**Figure 20:**
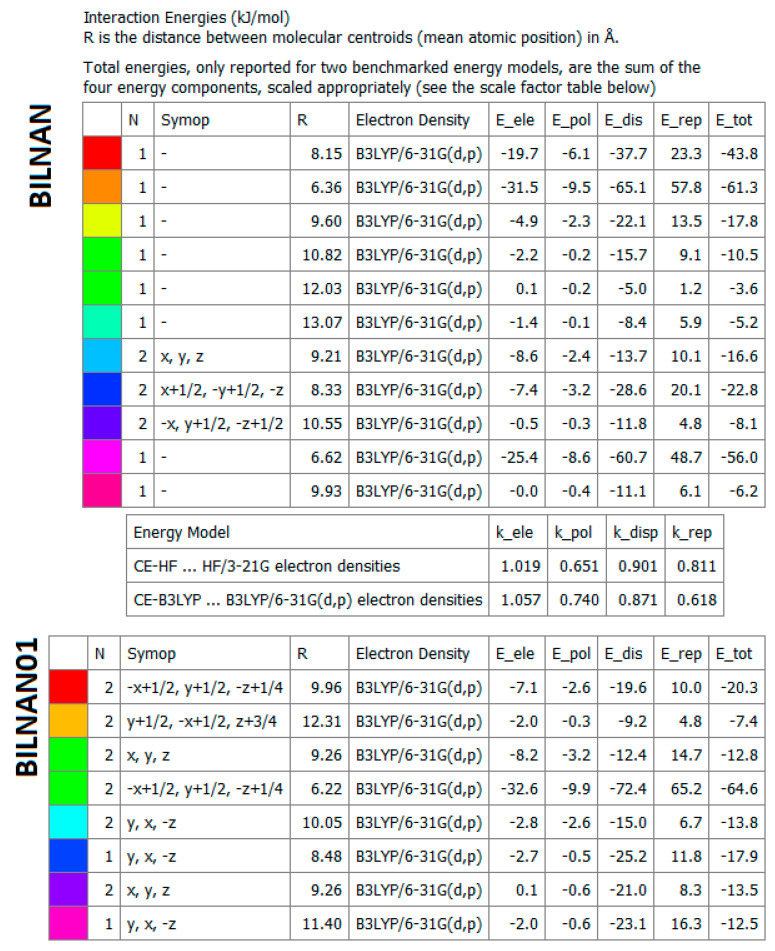
Interaction energies of the molecular pairs related to energy frameworks of DKP perindopril structures (Scale factors for benchmarked energy models).

**Figure 21:**
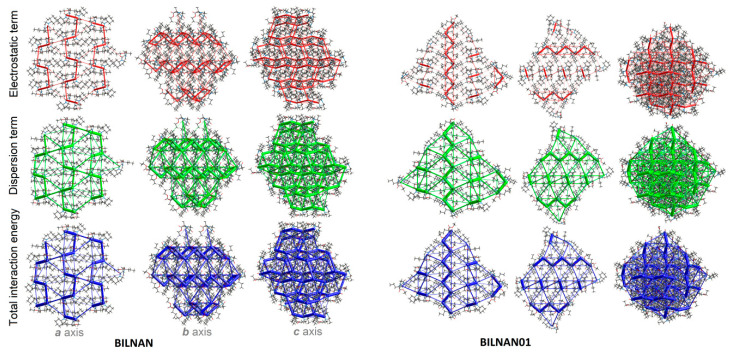
Energy frameworks of diketopiperazine perindopril structures corresponding to the electrostatic and dispersion energy components, and total energy framework along the *a, b* and *c*-axis (the tube size: 300).

**Figure 22:**
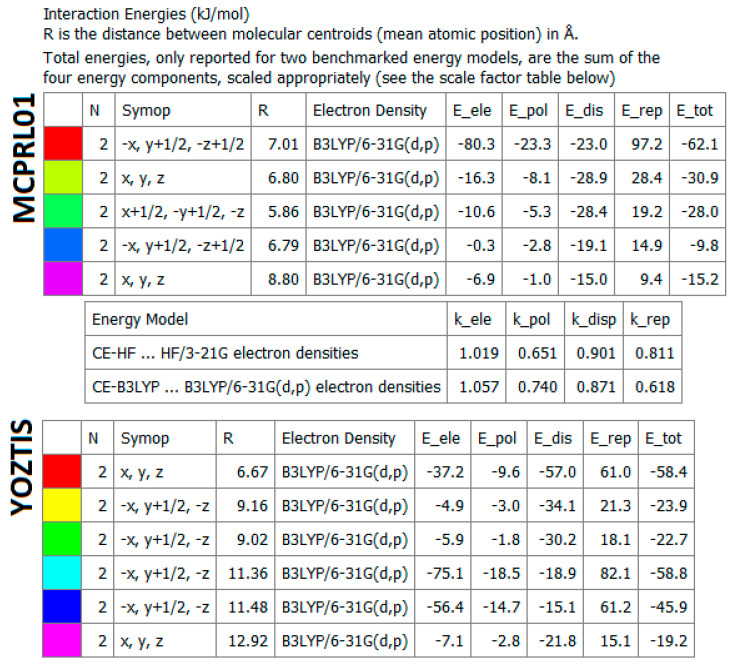
Interaction energies of the molecular pairs related to energy frameworks of captopril, on the left and captopril disulfide, on the right (scale factors for benchmarked energy models).

**Figure 23:**
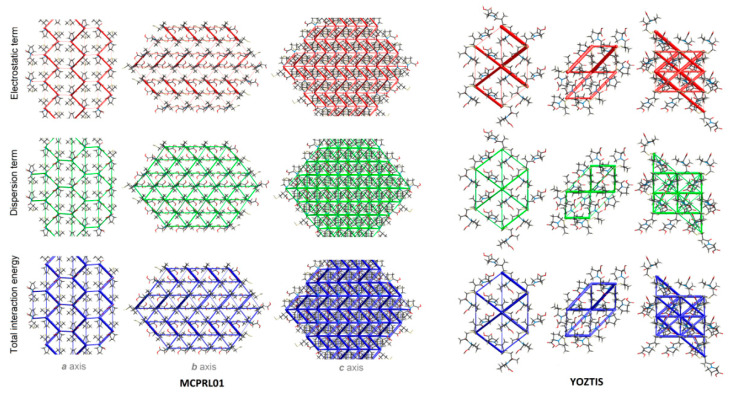
Energy frameworks of captopril (on the left) and captopril disulfide (on the right) corresponding to the electrostatic and dispersion energy components, and the total energy framework along the *a, b* and *c*-axis (the tube size: 300).

Quantitative analysis of intermolecular interactions is based on model energies consisting of electrostatic (E_ele_), dispersion (E_dis_), polarization (E_pol_) and exchange-repulsion (E_rep_) terms, which is expressed by the equation: E_tot_ = k_ele_ E′_ele_ + K_pol_ E′_pol_ + k_disp_ E′_disp_ + k_rep_ E′_rep_, where *k* is the scale factor. The 3-D topology of inter-contacts, providing a more balanced perspective on the nature of the predominant intermolecular interactions in ACEI, has been characterized via representing the network of the nearest energies in the form of a cylinders framework. The strength of the interaction is proportionally visualized by the width of theses cylinders. The corresponding molecular pairs are uniquely color-coded. The observed various values of energies result from the differences in crystal packing. More specifically, in proline structures (similar to e.g., perindoprilat, BECWIR or lisinopril, GERXEI) electrostatic terms related to the strong classical interactions playing a dominant role within the crystal lattice of the investigated systems (Figure 17, Figure 18 and Figure 19, Figures S15 and S16). On the other hand, in the supramolecular architecture of DKP perindopril polymorphs (BILNAN(01)), dispersion is dominated over the electrostatic energy, due to the high contribution of weak interactions (Figure 20 and Figure 21). Similar situation is observed in ramipril (QOQWAU) or trandolapril (IQISAE) (Figures S17 and S18). In the latter structure, E_disp_ is the highest stabilizing term. It is related to π^...^π interactions. Instead, in captopril (MCPRPL01) and its disulphide derivative (YOZTIS), the contributions of electrostatic and dispersion terms are comparable (both reach large values) as a result of a delicate balance between strong and weak interactions (Figure 22 and Figure 23). 

To sum up, quantifying inter-contacts in relation to their energies is helpful in deeper insight into the essential interactions leading to the construction of a supramolecular architecture, which is useful in rational drug design.

### 2.5. In Silico Control of Pharmacological Profile and Toxicity of ACEI and Their Impurities

There are no doubts that the safety and the highest quality of medicines are of primary importance [208,209,210,211,212]. The side effects of a given drug can also be induced by impurities, even if they are present in small amounts [213]. However, the determination of impurities may be difficult due to their structural similarity to the active substances [148,214,215]. Therefore, to improve the development of better ACEI, the complex quality control of current drugs together with their metabolites as well as the comprehensive pharmacological-toxicological investigations of ACEI were carried out in silico. Names and chemical formulas of impurities encountered in perindopril formulations are shown in Table S21.

**Figure 24:**
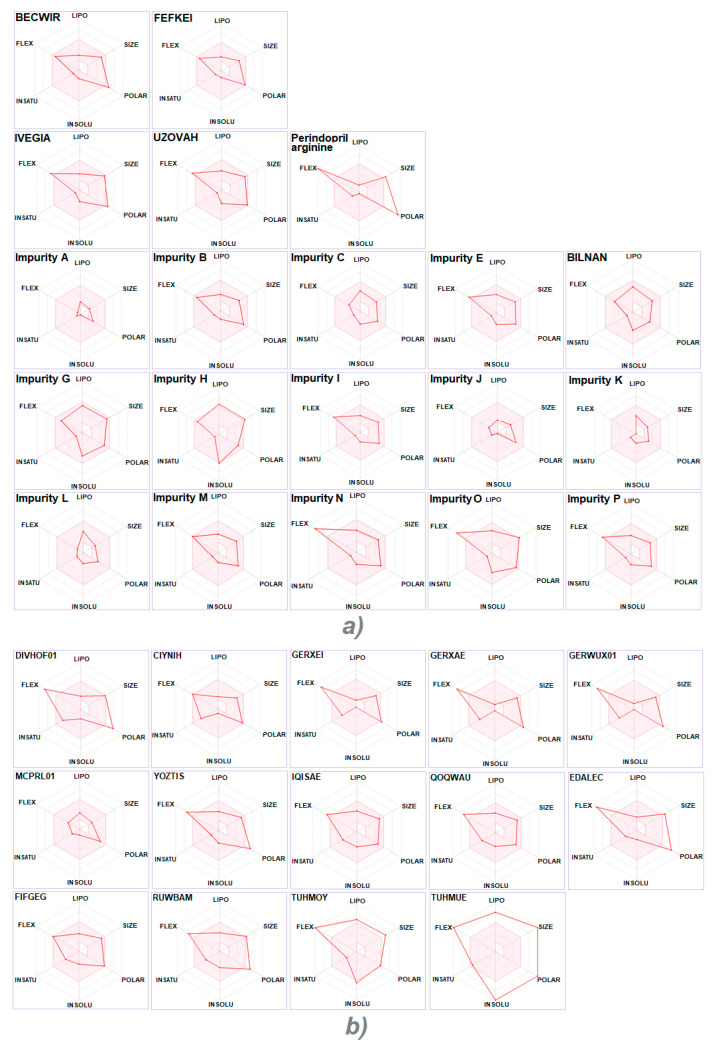
Bioavailability radars of major perindopril-derived compounds (**a**) augmented by remaining proline-based ACEI structures (**b**) as calculated by the Swiss ADME predictor.

Generally, investigated ACEI have similar pharmacokinetic profiles and toxic properties. The bioavailability radars [216] reveal satisfactory parameters of ACEI. The only three exceptions (TUHMOE, TUHMUY and perindopril arginine) are due to their high molecular weight (>500), see Figure 24. In particular, a PSA higher than 140 indicates strong polarity and a low body absorption [217], while below 100 shows good absorption and permeability. Liphophilicity is estimated by the AlogP98 parameter: ≤5 shows good liphophilicity; >5: illustrate poor liphophilicity [218]. The drug absorption as related to the membrane Caco-2 permeability (colon cancer cell line), skin permeability and intestinal absorption may be estimated by the Papp coefficient. Its value higher than 8 × 10 ^−6^ indicates substantial Caco-2 permeability and good absorption. Analyses revealed that the impurities A, BILNAN01 (imp. F), MCPRL01 and TUHMOY have high permeability. Intestinal absorbance <30% means poor absorption. In this regard, impurities B, FEFKEI, IVEGIA, FIFGEG, IQISAE, RUWBAM, CIYNIH, GERXAE, GERXEI, GERWUX01, TUHMUE, YOZTIS are poorly absorbed. All compounds have high skin permeability (log Kp < –2.5). Impurities B, C, D, E, F, H, EDALEC, FIFGEG, IQISAE, RUWBAM, QOQWAU, DIVFOF01, TUHMUE are substrates of P-glycoprotein. The latter is a member of the ATP-binding transmembrane glycoprotein family which excretes compounds from cells. The drug distribution is related to the blood–brain barrier (logBB), CNS permeability and the distribution volume (VDs), which describes the drug distribution in vivo. The value of VDs < 0.71 L kg^−1^ (log VDs < –0.15) indicates low distribution. On the other hand, VDDs > 2.81 L kg^−1^ (log VDs > 0.45) means the high distribution volume. All perindopril-derived compounds have low distribution volumes. The easy brain–blood barrier (BBB) permeability is connected with the log BB > 0.3, while difficult crossing of the barrier is related to the logBB < –1. Therefore, the impurity B, perindopril arginine, FIFGEG, IQISAE, TUHMUE, YOZTIS are unlikely to cross the barrier. From the CNS permeability point of view, logPS < –3 predicts difficulties in penetration of CNS. Nearly all studied compounds are unable to permeate CNS. The only exception is TUHMOY. Metabolism parameters depend on cytochrome P450 models (CYP2D6 and CYP3A4 substrates or CYP1A2, CYP2C19, CYP2C9, CYP2D6 and CYP3A4 inhibitors). These are crucial enzyme systems for drug metabolism in the liver. Impurities B, C, D and E, UZOVIP, UZOVAH, BILNAN01, perindopril arginine, EDALEC, FIFGEG, IQISAE, RUWBAM, QOQWAU, DIVHOF01, TUHMOY, TUHMUE are substrates of either CYP2D6 or CYP3A4 enzymes. Estimation of the drug excretion is based on the total clearance model and the renal OCT2 substrate [216]. The highest total clearance was detected for impurities F, IQISAE, GERWUX01.

Toxicity was evaluated by the AMES test which predicts the mutagenic activity, the hERG inhibition, hepatotoxicity and skin sensitization. None of the compounds exhibited either cardiotoxicity due to hERG channel inhibitions or skin sensitization. Nevertheless, nearly all active substances are hepatotoxic with the captopril being the only exception. Impurities of perindopril: G, K, L are not hepatotoxic. All ADMET parameters are summarized in Tables S22–S26. 

## 3. Materials and Methods

### 3.1. DFT Studies

Standard geometry optimizations of the neutral molecules under study in singlet ground spin states starting from their experimental X-ray structures [38,184,185,189] were performed at the DFT level of theory using the M06 hybrid functional [219] and 6–311++G(d,p) basis sets for all atoms [220] in aqueous solutions, where solvent effects were approximated within the Conductor-like Polarizable Continuum Model (CPCM) [221,222,223]. Stability of the optimized structures was confirmed by vibrational analysis (no imaginary vibrations). Atomic charges were evaluated within Natural Population Analysis (NPA) [224,225,226,227]. All calculations were performed by the Gaussian09 program package [228]. Moldraw [229] software was used for molecular structure manipulation and visualization.

### 3.2. Hirshfeld Surface Analysis and Molecular Electrostatic Potentials

Qualitative and quantitative analysis of inter-contacts were performed via Hirshfeld surface (HS) study [230] using the CrystalExplorer 17.5 [231] program, based on the final X-ray crystallographic information files as the inputs. For this purpose, 3-D Hirshfeld surface maps and 2-D fingerprint plots [232] were generated. The bond lengths of the H-atoms were normalized to standard neutron diffraction values. The color schemes of the surface areas are related to the normalized contact distance, *d_norm_,* defined in terms of the van der Waals (vdW) radii of the atoms and *d_e_*, *d_i_*, the distances from the Hirshfeld surface to the nearest atom outside (external) and inside (internal) the surface, respectively. Red spots show interatomic contact shorter than the sum of vdW radii, white–distances equal to the sum of vdW radii, while blue—distances longer than the sum of vdW radii. The electrostatic potential is mapped on Hirshfeld Surface using wave function STO-3 G basis sets at the Hartree-Fock theory level over the range of ±0.020 au. Red and blue regions represent a negative (H-acceptors) and positive electrostatic potential (H- donors), respectively [233].

### 3.3. Enrichment Ratio

Privileged and disfavoured interactions from a chemical elements point of view can be highlighted in a crystal structure via the enrichment ratios.

The enrichment ratio of atomic contacts is a ratio between the actual interactions within the crystal structure and those derived from the theoretical calculations as if all types of contacts had the same probability of forming. This indicator derives from the Hirshfeld surface calculations decomposing the crystal surface between pairs of interacting chemical species. The value larger than unity is related to the contacts with a high propensity in the crystal, while if the enrichment ratio is lower than unity, the propensity is low. The contacts between one (X^…^X) or two (X^…^Y) chemical elements in a crystal packing is information given by Hirshfeld surface (HS) analysis. *C_XY_* (including either X^…^Y or Y^…^X contacts related to the interior and exterior atoms to the HS) is the proportion of HS contacts [204]. The proportion *S_X_* on the molecular surface is given by the following equation

*S_X_* = *C_XX_* + ½ Σ*_Y≠X_ C_XY_*  (Σ*_X_ S_X_* = 1). (1)

Then, the ratio of inter-contacts are defined as:

*R_XY_* = *S_X_S_X_andR_XY_* = 2 *S_X_S_Y_*    (Σ_*X*_*R_XX_* + Σ_*Y≠X*_*R_XY_* = 1). (2)

Finally, the enrichment ratio:

*E_XY_* = *C_XY_*/*R_XY_* (3)

### 3.4. Energy Frameworks

The crystal packing in relations to the interactions energy was constructed by the method of energy frameworks integrated with the newest version of CrystalExplorer 17.5 [231] using a single-point molecular wavefunction at B3Lyp/6-31G(d,p) level of theory. In particular, calculated energies between the molecular pairs were used to generate 3D topology frames of the main interactions. A cluster of radius 3.8 Å was formated with respect to the central molecule [231,234]. The neighbouring molecules (density matrices) were generated by applying crystallographic symmetry operations. The energy benchmark is calculated by Mackenzie’s method to scale various energy frameworks

### 3.5. SwissADMET Profile

Absorption, distribution, metabolism, excretion, toxicity (ADMET) profiles for ACEI were calculated using the pkCSM web-platform [235,236]. The information was supplemented by other pharmacological parameters analyzed by SwissADME web-based interface, provided by the Molecular Modeling Group of the Swiss Institute of Bioinformatics [237]. 2D structural models were drawn in the molecular sketcher into the ChemAxon‘s Marvin JS window and transferred to the simplified molecular-input line-entry system (SMILES) format to predict suitable properties.

## 4. Conclusions

Proline is a unique amino acid that is attracting increasing attention in contemporary medicinal chemistry and supramolecular pharmacology with special emphasis on its specific topological properties. It is frequently localized in turns and other active sites of protein chains and contrains conformation of short peptides. In this work, we thoroughly analysed supramolecular features of prolines and further applied this approach to angiotensine converting enzyme inhibitors. The latter are frontline therapeutics applied in hypertension and cardiovascular diseases. Their supramolecular structures have not been precisely systematically analysed by contemporary approaches to date. The rational drug design involves multidisciplinary studies. A detailed characterization of synthons and tectons involving proline derivatives may be useful for in silico molecular docking and macromolecular modelling studies. The comprehensive search of the Cambridge Structural Database yielded an impressive number of entries (2366) which were used to create the libraries of conformational and supramolecular features. Twenty proline-based ACEI were subjected to detailed DFT, Hirshfield surface and energy framework studies for the comprehensive description of tectons and synthons. Tectonic subunits contain proline moieties characterized by diverse ionization states: –N and –COOH(–COO^−^), –N^+^ and –COOH(–COO^−^), –NH and –COOH(–COO^−^), –NH^+^ and –COOH(–COO^−^) and –NH_2_^+^ and –COOH(–COO^−^). In particular, tectons with NH_2_^+^ and COO^−^ and -N and COOH(COO^−^) functionalities are the most popular. They are also observed in the ACEI structures, in which they facilitate the formation of single and bifurcated O–H^…^O, O–H^…^N, N–H^…^O, C–H^…^O, S–H^…^O, and O–H^…^Cl^−^ supramolecular synthons. Similar interactions were found in the proline containing bio-complexes. The pyrrolidine ring participates in the _(-CcyclPRO)_C–H^…^O[S, Cl^−^]_(COOH/COO-/SH/Cl–)_ interactions. The Hirshfeld surface analysis revealed that C^…^C, C^…^O/O^…^C, O^…^O and Na^…^O(H)/(H)O^…^Na inter-contacts cannot be neglected. Energy framework calculations identified the most important inter-contacts in ACEI. In particular, proline, perindoprilat, and lisinopril structures are governed by the classic, electrostatic forces while, in DKP, perindopril polymorphs, ramipril, trandolapril, dispersion effects are preferred. The tiny balance between electrostatic and dispersion was observed in captopril and its disulfide. ADMET pharmacological profiles are similar for all ACEI and their impurities. Prodrugs are mostly hepatotoxic with the captopril and perindopril impurities, with G, K and L being the exception. Libraries of structural, conformational, and pharmacological data for ACE inhibitors may be used in studies on smart ligands containing proline and acting via inhibition of either ACE or similar targets. A comprehensive characterization of related synthonic interactions can help to design model ligands for efficient enzyme binding. 

The comprehensive summary of weak intermolecular interactions in proline-based ACEI enhances knowledge on the supramolecular recognition in bio-complexes of ACE with potential new effective inhibitors. As highlighted in this paper, the synthon concept can also be successfully applied in the protein environment. Structural data determined by X-ray crystallography gave insight into the supramolecular forces. Additional methods, like Hirshfeld surface analysis and energy frameworks help to explain how tiny preferences of H-bonds donors and acceptors affect the overall supramolecular topology. Those results allow the identification of binding predispositions of conformationally flexible ACEI. The advanced bio-structural summary sheds light on new supramolecular strategies of rational design of modern inhibitors with better biological activity and reduced side effects combined with a high affinity and specificity. In this respect, we proved that not only the macromolecular, but also small-molecular, approaches have relevance. 

Future work will focus on proline substituents as building blocks for the formation of synthons. Special attention will be paid to cooperative effects, which are frequently neglected in the crystal structure interpretations. They affect the entropy of supramolecular entities and are important for their overall characterization. 

## Figures and Tables

**Table 1:** Proline tectons in proline and ACEI structures as found in the CSD (three types of proline-based tectons, I, II, III are color-coded).

I	Conformation	II	Conformation	III	Conformation
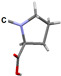		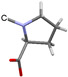		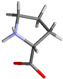	
BECWIR	trans	IVEGIA	trans	NELSEC	trans
FEFKEI	trans	UZOVAH03	trans	PROLIN03	trans
MCPRL01	trans	DIVHOF01	trans	QANRUT	trans
CIYNIH	trans	EDALEC	trans		
IQISAE	cis	FIFGEG	trans		
QOQWAU	cis	GERXAE	trans		
RUWBAM	trans	GERXEI	trans		
YOZTIS	trans	GERWUX01	trans		

**Table 2:** Major interactions, proportions on the HS and ER for perindopril structures.

Interactions	IVEGIA	UZOWAH03		
Surface (%)	H		O	
	85.4	87.9	14.2	11.2
Major contacts	H^…^H		O*^…^*H	
Proportion (%)	*73.8*	*76.6*	*23.2*	*22.6*
ER	1	1.0	0.96	1.14

**Table 3:** Major interactions, proportions on the HS and ER for perindoprilat structures.

Interactions	BECWIR	FEFKEI						
Surface (%)	H		O		C		S	
	61.95	81.4	18	18.05	0.45	0.45	0.7	-
Major contacts	H^…^H		O*^…^*H		C^…^H		S^…^H	
Proportion (%)	60.8	62.9	36	36.1	0.9	0.9	1.4	-
ER	1.58	0.95	1.61	1.23	1.61	1.23	1.61	-

**Table 4:** Major interactions, proportions on the HS and ER for DKP perindopril polymorphs.

Interactions	BILNAN	BILNAN01				
Surface (%)	H		O		N	
	86.3	88.25	11.35	10.35	0.45	
Major contacts	H^…^H		O*^…^*H		N^…^H	
Proportion (%)	74.5	77.9	22.7	20.7	0.9	
ER	1.0	1.0	1.16	1.1	1.15	

## Supplementary Materials

The following are available online at https://www.mdpi.com/1424-8247/13/11/338/s1. Additional figures and tables can be found. In particular: Scheme S1. Proline-based ACEI. Scheme S2. Modified proline-based ACEI and their derivatives. Scheme S3. Conformational properties for proline: type of isomer, conformation of 5-membered ring and puckers of pyrrolidine ring. Figure S1. Molecular diagrams of proline structures, retrieved from the CSD. Figure S2. Molecular diagrams of crystal structures of proline-based ACEI. Figure S3. Molecular diagrams of perindoprilat structures. Figure S4. Molecular diagrams of perindopril erbumine structures. Figure S5. Molecular diagrams of DKP perindopril erbumine structures. Figure S6. Molecular diagrams of other modified proline-based ACEI crystal structures. Figure S7. Occurence of interactions forming proline-based synthons in three types of investigated proline and ACEI structures. Figure S8. View of the 3D Hirshfeld surface of perindopril-derived crystal structures. Figure S9. Full FPs for all investigated proline-based structures. Figure S10. FPs for perindopril-derived structures decomposed into the major non-covalent interactions: H^...^H, O^…^H/H^…^O, C^…^H/H^…^C and S^…^H/H^…^S. Figure S11. HS *d_norm_* and EPs of proline-based and other modified-proline-based ACEI crystals, retrieved from the CSD. Figure S12. Proline-based synthons occuring in perindopril-derived and proline structures, resulting from three types of proline-based tectons. Figure S13. Simplified hydrogen-bonded networks showing LSAMs in proline-based ACEI crystals. The black or blue dots represent a center of gravity of the molecule or ion, orange-shaded lines stand for O-H^…^O or N-H^…^O hydrogen bonds, blue, grey, and yellow lines for C-H^…^O, C-H^…^π and C-H^…^S contacts, respectively. Blue and gray spheres represent the positions of water molecules and sodium cations, respectively. Figure S14. Simplified hydrogen-bonded networks in other modified proline-based ACEI structures. The black and blue dots represent a center of gravity of molecules or ions. Red lines stand for O-H^…^O or N-H^…^O hydrogen bonds, blue and green lines depict C-H^…^O contacts and gray lines N-H^…^π contacts. Water molecules or methanol oxygen atoms are represented as blue spheres. Chlorine anions are presented as green spheres. Figure S15. Color coding of neighbouring molecules in perindoprilat structure (BECWIR) in relations to the central molecule (gray). Energy frameworks corresponding to the electrostatic and dispersion energy components, and total energy framework along *a, b* and *c*-axis in crystal packing of perindoprilat structure. Figure S16. Interaction energies of lisinopril and energy frameworks corresponding to the electrostatic and dispersion energy components, and total energy framework. Figure S17. Interaction energies of ramipril. Figure S18. Interaction energies of the molecular pairs related to energy frameworks of IQISAE01. Table S1. Proline-based tectons and proline-derived structures, retrieved from the CSD. Table S2. Chemical names of analyzed compounds. Table S3. Crystal data of proline structures, proline-based and modified-proline-based ACEI structures and their derivatives. Table S4. Conformation of pyrrolidine ring in investigated structures. Table S5. Conformation of COOH/COO^−^ group of proline ring in investigated structures. Table S6. Relevant atom charges, heteroatom-hydrogen bonds, d(H-X), relative DFT, ΔE_DFT_, and Gibbs free energies at room temperature, ΔG_298_, of stable structures of L-proline (1) obtained at M06/6-311++G(d,p)/CPCM(H_2_O) level of theory. Table S7. Relevant atom charges, heteroatom-hydrogen bonds, d(H-X), relative DFT, ΔE_DFT_, and Gibbs free energies at room temperature, ΔG_298_, of stable structures of FEFKEI (2) obtained at M06/6-311++G(d,p)/CPCM(H_2_O) level of theory. Table S8. Relevant atom charges, heteroatom-hydrogen bonds, d(H-X), relative DFT, ΔE_DFT_, and Gibbs free energies at room temperature, ΔG_298_, of stable structures of protonated IVEGIA (3) obtained at M06/6-311++G(d,p)/CPCM(H_2_O) level of theory. Table S9. H-bond geometries of all investigated crystal structures. Table S10. Geometrical parameters (in Ǻ and in °) for the π-stacking moieties involved in the π^…^π interactions for studied compounds. Table S11. Geometrical parameters (in Ǻ and in °) for the other π-stacking moieties for studied compounds. Table S12. HS analysis of ACEI and their derivatives available in the CSD. Table S13. HS parameters for analyzed structures. Table S14. HS interaction surfaces, random contacts and enrichment ratios for all analyzed structures. Table S15. Supramolecular synthons resulting from three types of proline-based tectons observed in ACEI structures. Table S16. Supramolecular synthons in relations to three types of proline-based tectons, observed in the ACEI crystal structures. Table S17. Supramolecular synthon patterns forming by COOH(COO^−^) of pyrrolidine ring of proline in investigated crystals. Table S18. Supramolecular synthon patterns forming by COOH(COO^−^) in chain of proline in investigated crystals. Table S19. Supramolecular synthon patterns formed by (NH_2_^+^) in proline chain of investigated crystal structures. Table S20. Library of all supramolecular synthon patterns in all investigated crystals. Table S21. Names of perindopril *tert*-butylamine impurities. Table S22. ADMET parameters for perindopril-derived compounds. Table S23. ADMET parameters of perindopril impurities. Table S24. ADMET parameters for proline-based ACEI compounds. Table S25. ADMET parameters for other modified proline-based ACEI compounds. Table S26. SwissADME parameters for proline-based and modified-proline-based ACEI compounds.

## Abbreviations

The following abbreviations are used in this manuscript:

ACE	angiotensin-coverting enzyme
ACEI	ACE inhibitors
ADMET	absorption, distribution, metabolism, excretion, toxicity
BBB	blood-brain barrier
CNS	central nervous system
CSD	Cambridge Structure Database
COX	cycloooxygenase
CPCM	conductor like polarizabel continue model
2D	2-dimensional
3D	3-dimensional
d_e_	distances from the HS to the nearest atom outside the surface
DFT	Density Functional Theory
d_i_	distances from the HS to the nearest atom inside the surface
DKP	diketopiperazine
d_norm_	normalized contact distance
EP	electrostatic potential
ER	enrichment ratio
FP	fingerprint plot
HS	Hirshfeld surface
LSAM	Long-range synthon Aufbau modules
RCSB PDB	Research Collaboratory for Structural Bioinformatics Protein Data Bank
E_ele_	electrostatic term of energy:
E_disp_	dispersion term of energy
E_rep_	repulsion term of energy
E_pol_	polarization term of energy
E_tot_	total energy
NPA	natural population analysis
PSA	polar surface area
Vds	
vdW	van der Waals radii
WHO	World Health Organization
