# Supplementary material for: A Proline-Based Tectons and Supramolecular Synthons for Drug Design 2.0: A Case Study of ACEI
Source: Pharmaceuticals (Basel). 2020 Oct 24;13(11):338. doi: 10.3390/ph13110338 (PMC7692516; doi:10.3390/ph13110338)
Supplement: Supplementary file 1 [file pharmaceuticals-13-00338-s001.pdf]

SUPPLEMENTARY MATERIALS

„Corpora non agunt nisi fixata” Paul Ehrlich

A proline-based tectons and supramolecular synthons for drug design 2.0: A case study of ACEI

Joanna Bojarska[1*], Milan Remko[2], Martin Breza[3], Izabela Madura[4], Andrzej Fruziński[1] and Wojciech M. Wolf[1]

1 Institute of General and Ecological Chemistry, Lodz University of Technology, Faculty of Chemistry, Żeromskiego 116, Lodz, 90- 924, Poland; wojciech.wolf@p.lodz.pl (W.M.W.); joanna.bojarska@p.lodz.pl (J.B.); andrzej.fruziński@p.lodz.pl (A.F.)

2 Remedika, Sustekova 1, 85104, Bratislava, Slovakia; milan.remko@gmail.com (M.R.)

3 Department of Physical Chemistry, Slovak Technical University, Radlinskeho 9, SK-81237 Bratislava, Slovakia; martin.breza@stuba.sk (M.B.)

4 Warsaw University of Technology, Faculty of Chemistry, Noakowskiego 3, 00-664, Warsaw, Poland; izabela@ch.pw.edu.pl (I.D.M.)

* Correspondence: joanna.bojarska@p.lodz.pl; Tel.: +48 42 631 31 19.

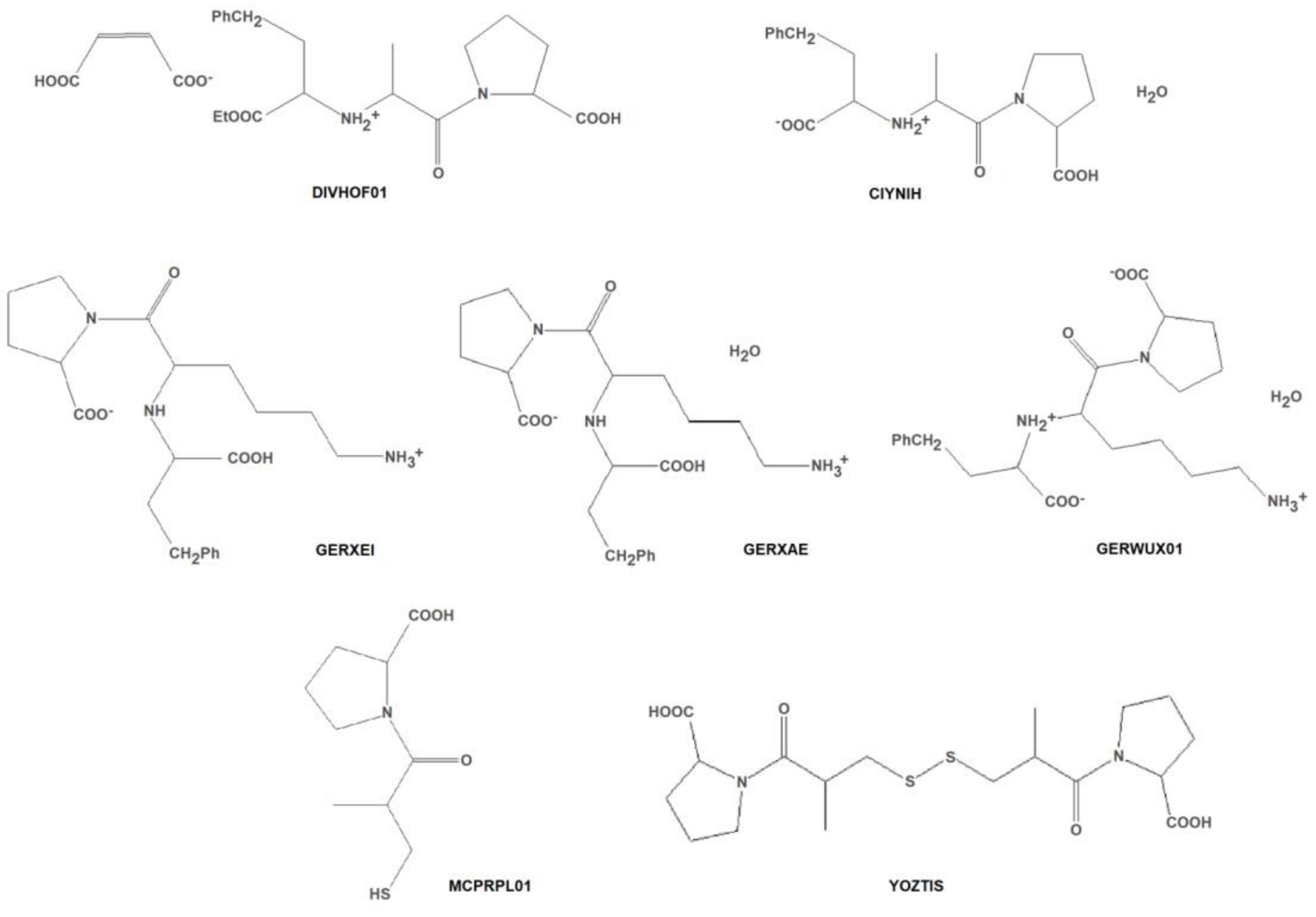

Scheme S1. Proline-based ACEI.

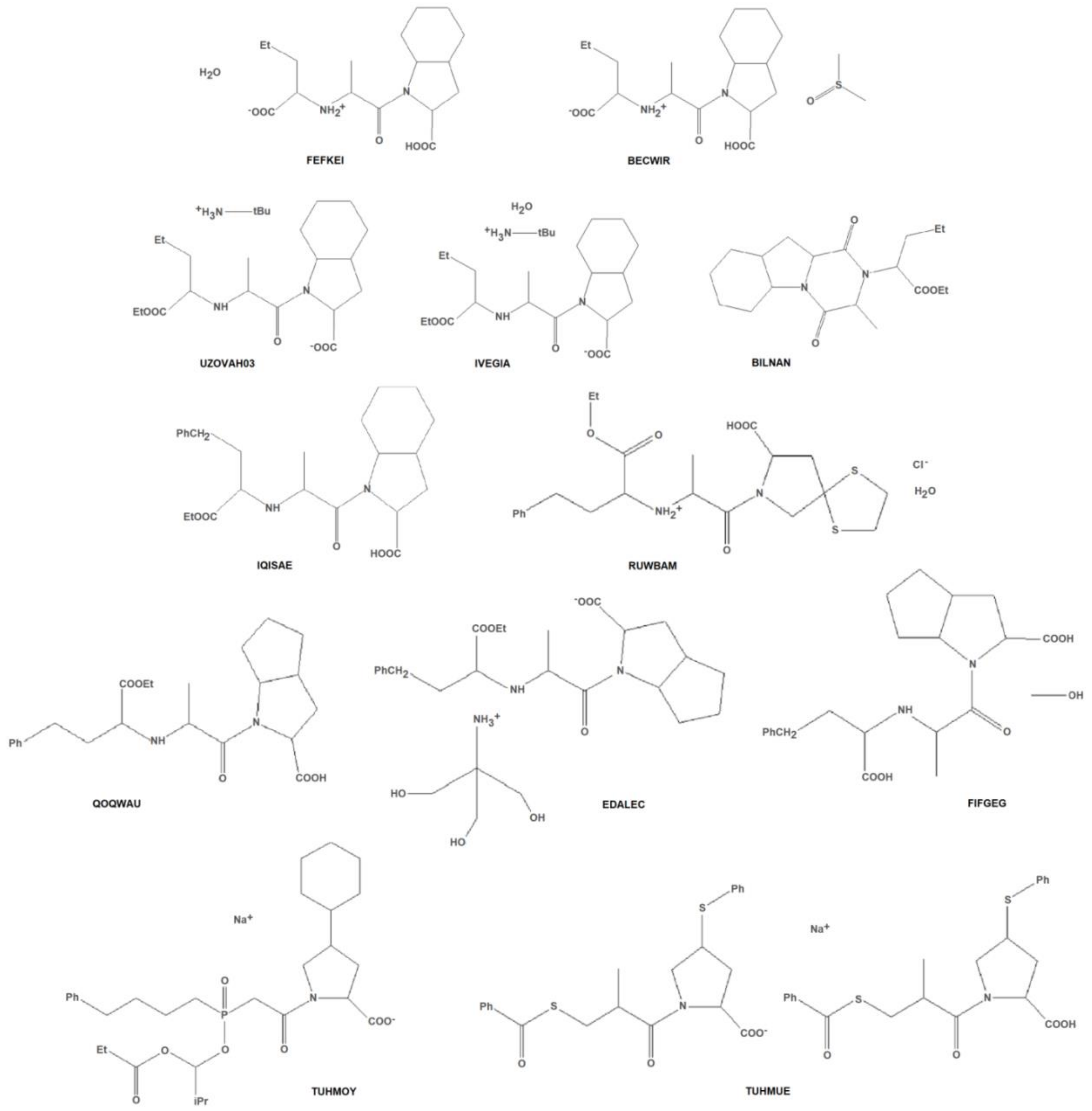

Scheme S2. Modified proline-based ACEI and their derivatives.

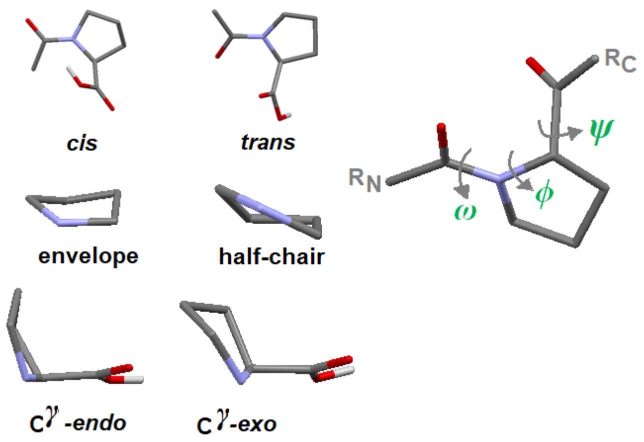

Scheme S3. Conformational properties for proline: type of isomer, conformation of 5-membered ring and puckers of pyrrolidine ring.

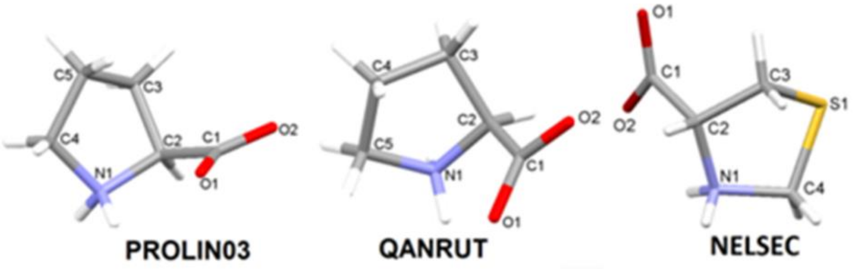

Figure S1. Molecular diagrams of proline structures, retrieved from the CSD.

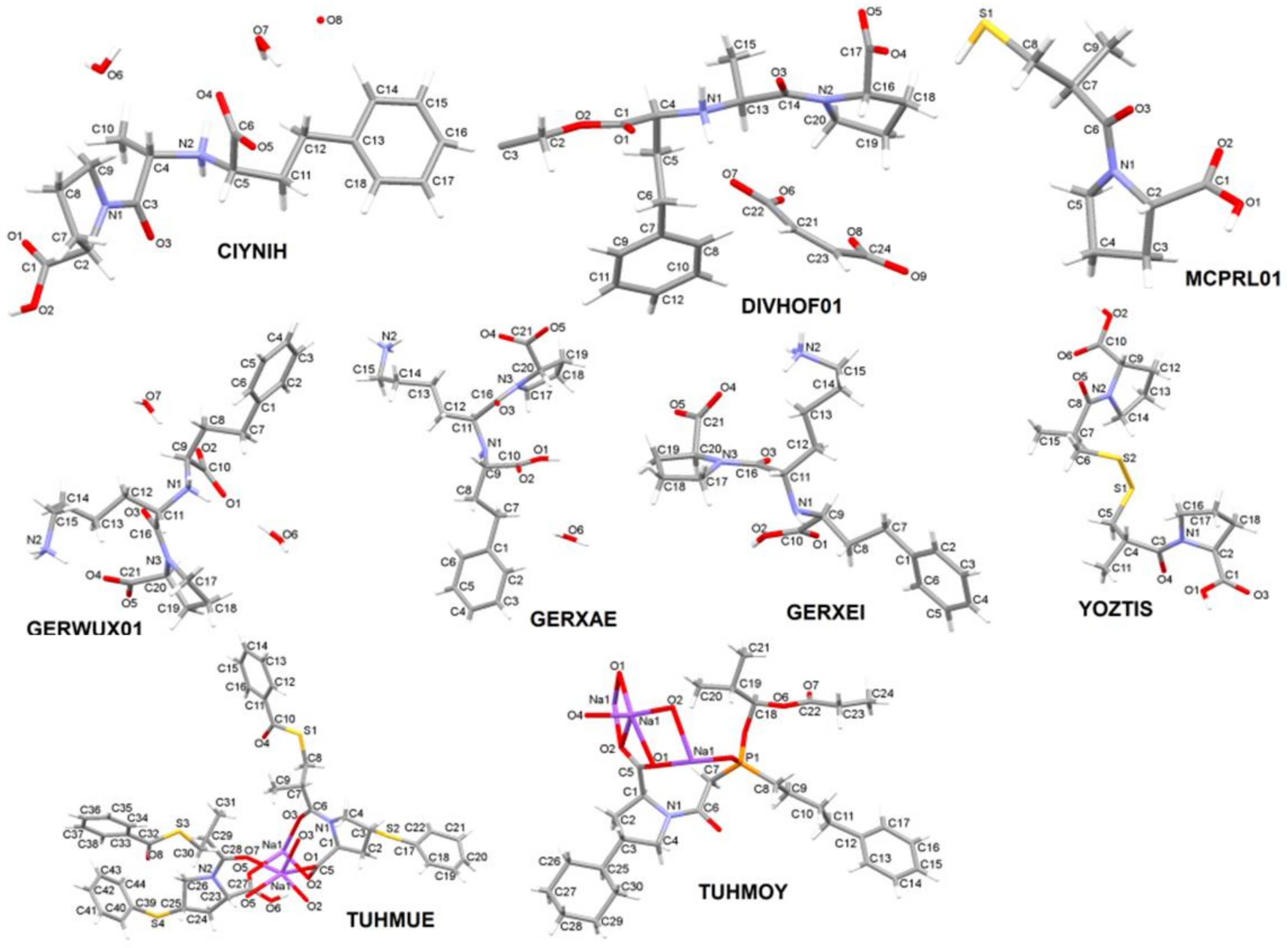

Figure S2. Molecular diagrams of crystal structures of proline-based ACEI.

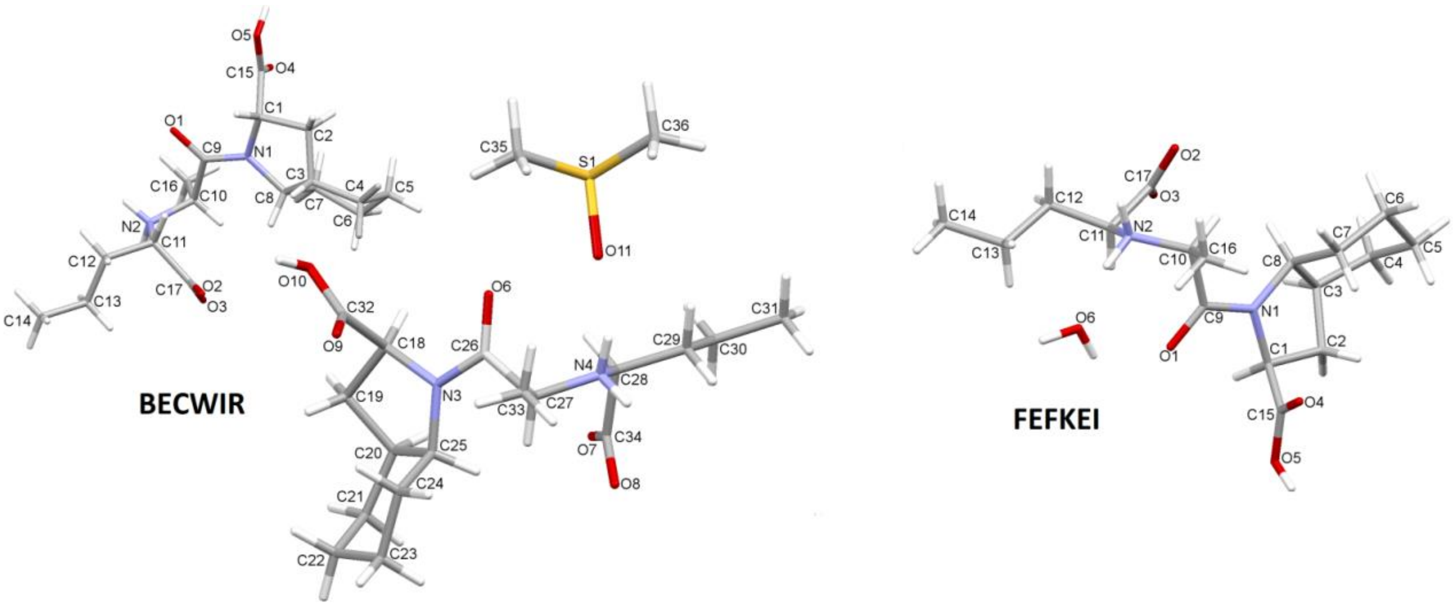

Figure S3. Molecular diagrams of perindoprilat structures.

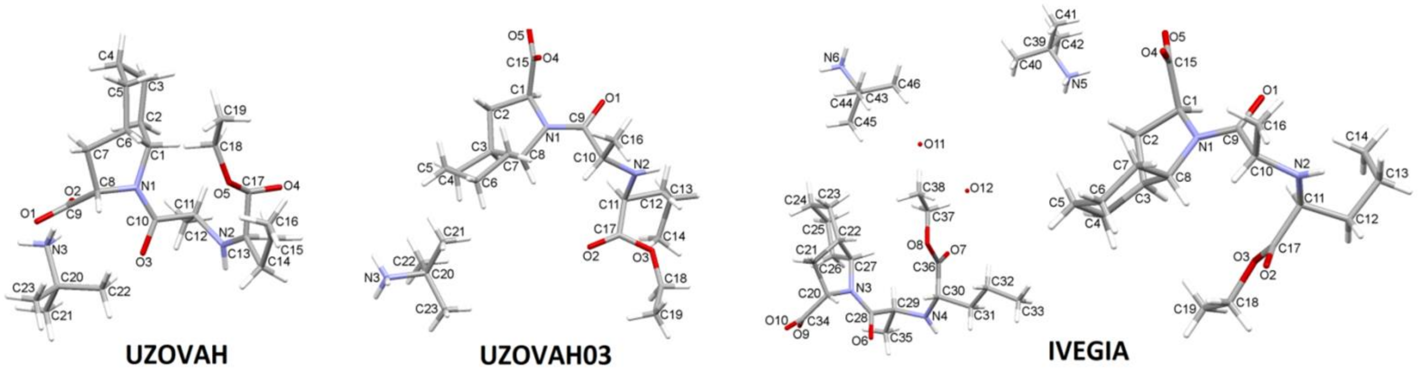

Figure S4. Molecular diagrams of perindopril erbumine structures.

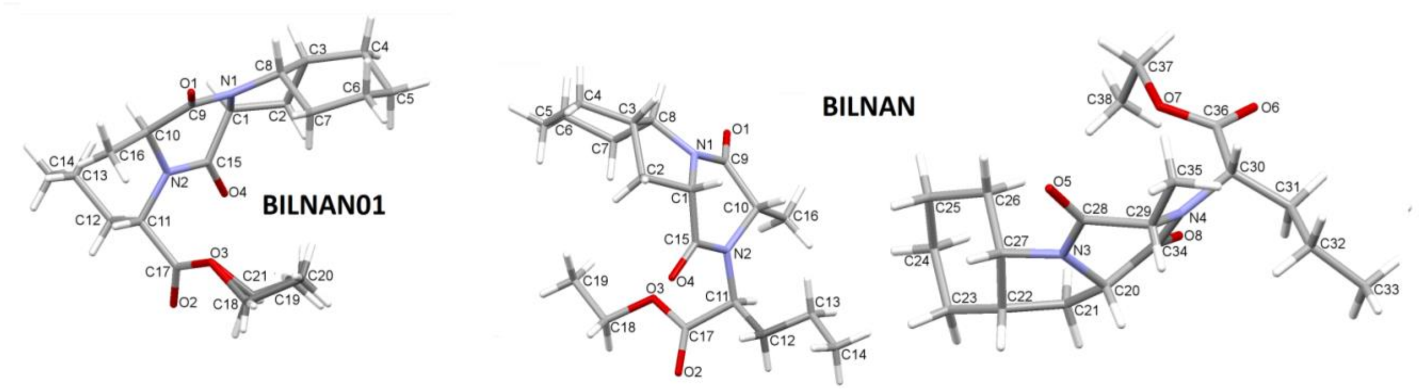

Figure S5. Molecular diagrams o[illegible]KP perindopril erbumine structures.

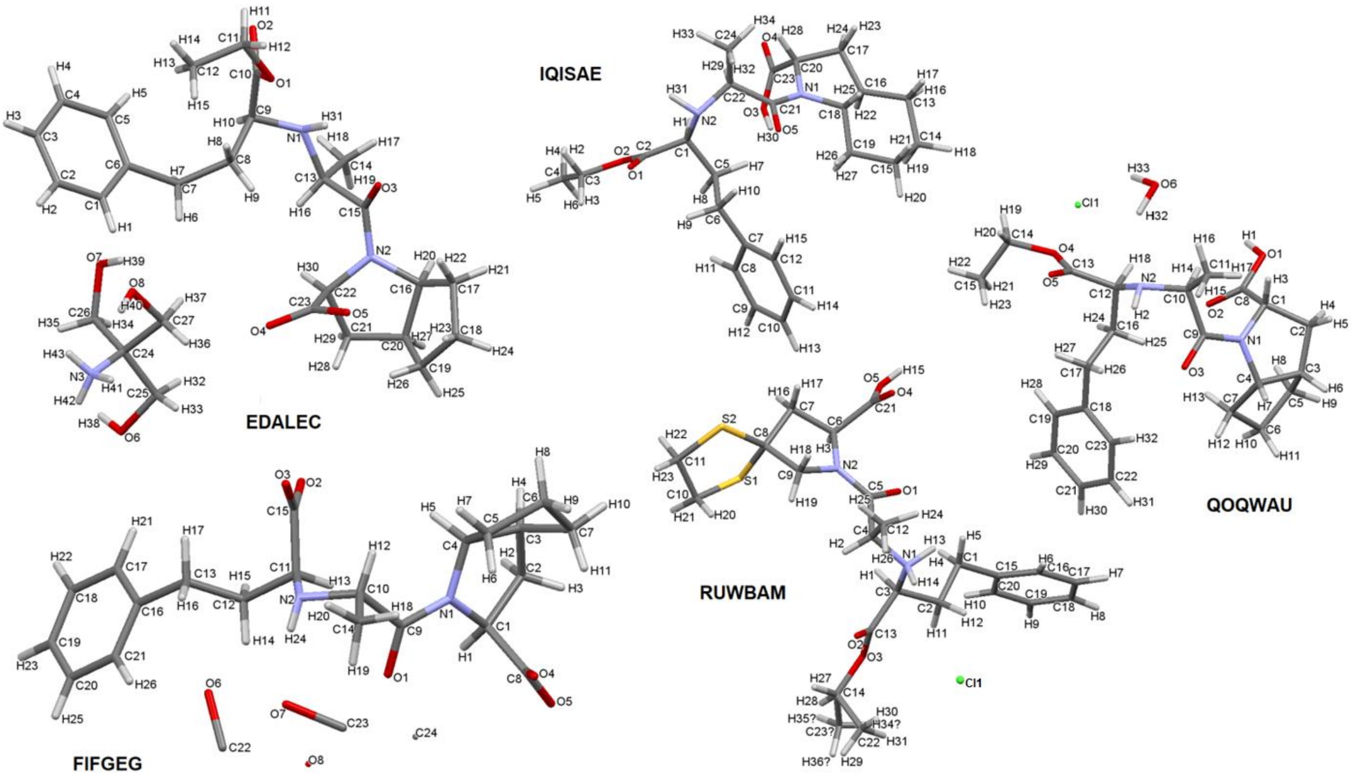

Figure S6. Molecular diagrams of other modified proline-based ACEI crystal structures.

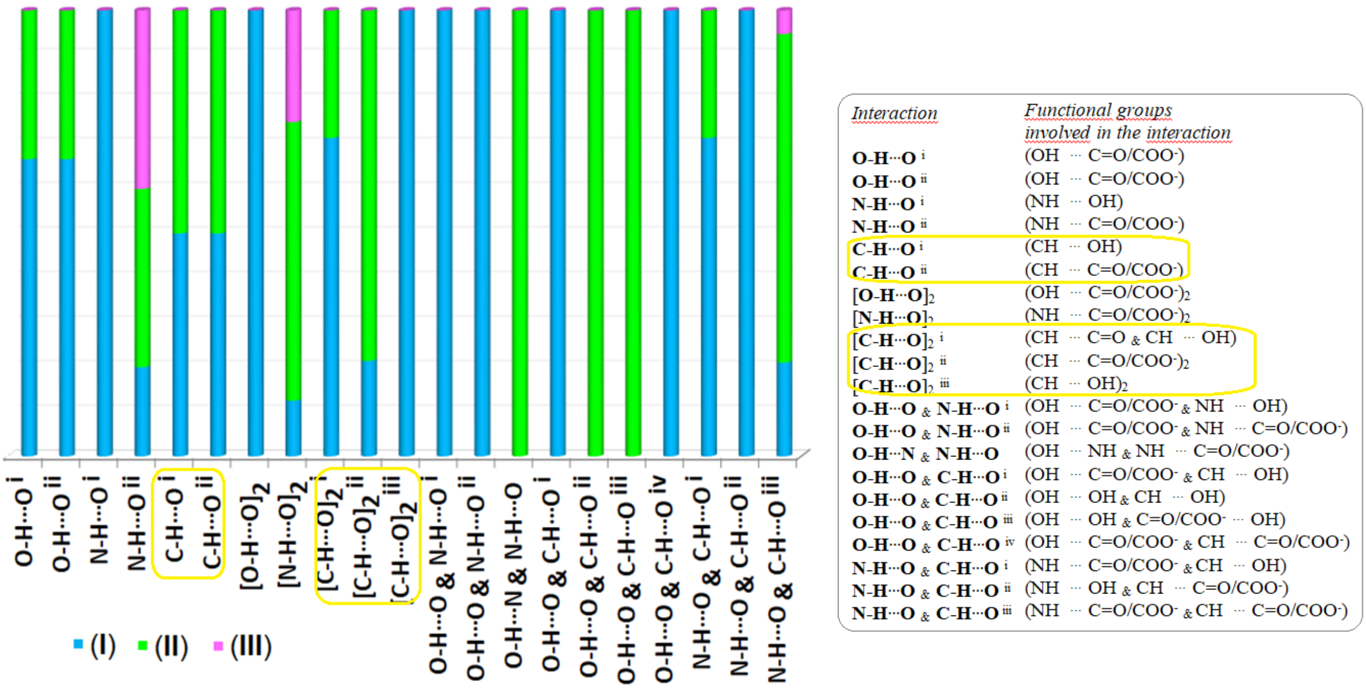

Figure S7. Occurence of interactions, which form proline-based synthon patterns in three types of investigated proline and ACEI structures [(I): (N-C) COOH; (II): (N-C); COO$^-$ (III): NH_2^+ COO$^-$].

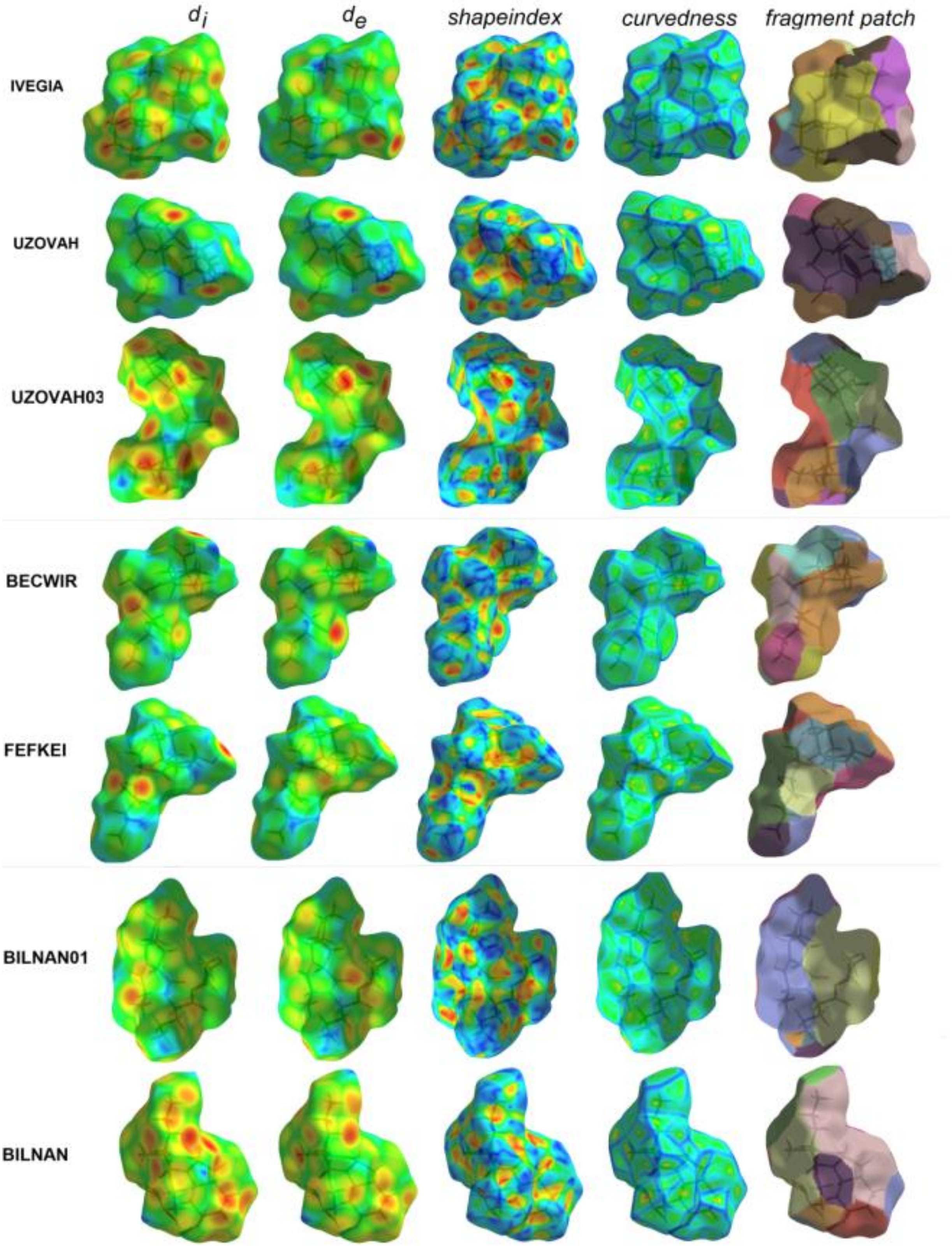

Figure S8. View of the 3D Hirshfeld surface of perindopril-derived crystal structures mapped with $d_{i,}$ d_e, s*hapeindex*, *curvedness* and *fragment patch* profiles. The surfaces are shown as transparent to allow visualization of the orientation and conformation of the functional groups.

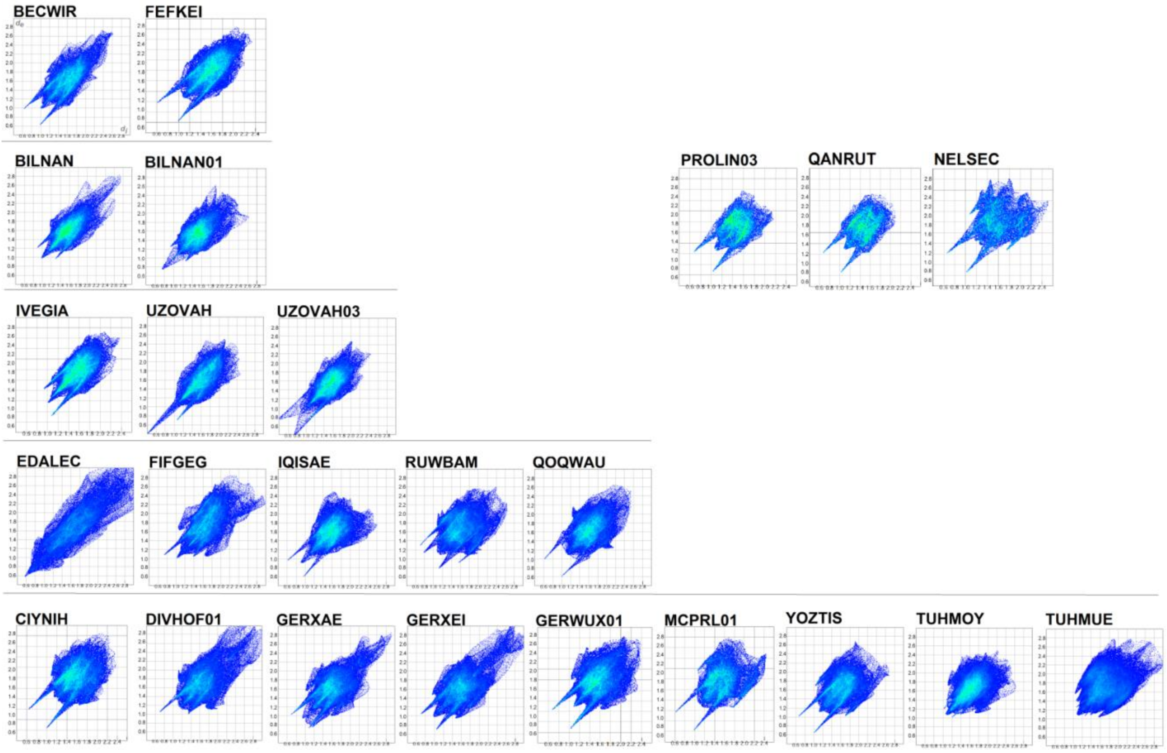

Figure S9. Full FPs for all investigated proline-based structures: proline structures (PROLIN03, QANRUT, NELSEC – SPR.), perindoprilat crystals (BECWIR, FEFKEI), DKP perindopril polymorphs (BILNAN, BILNAN01), perindopril structures (IVEGIA, UZOVAH, UZOVAH03), proline-based crystals (EDALEC, FIFGEG, IQISAE, RUWBAM, QOQWAU) and other modified proline-based structures (CIYNIH, DIVHOF01, GERXAE, GERXEI, GERWUX01, MCPRL01, YOZTIS, TUHMOY, TUHMUE).

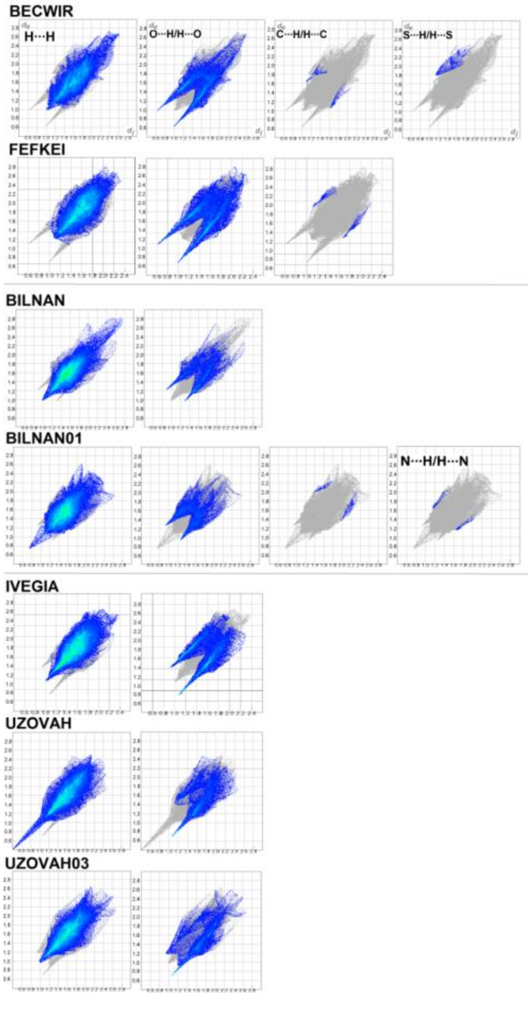

Figure S10. FPs for perindopril-derived structures decomposed into the major non-covalent interactions: H···H, O···H/H···O, C···H/H···C and S···H/H···S.

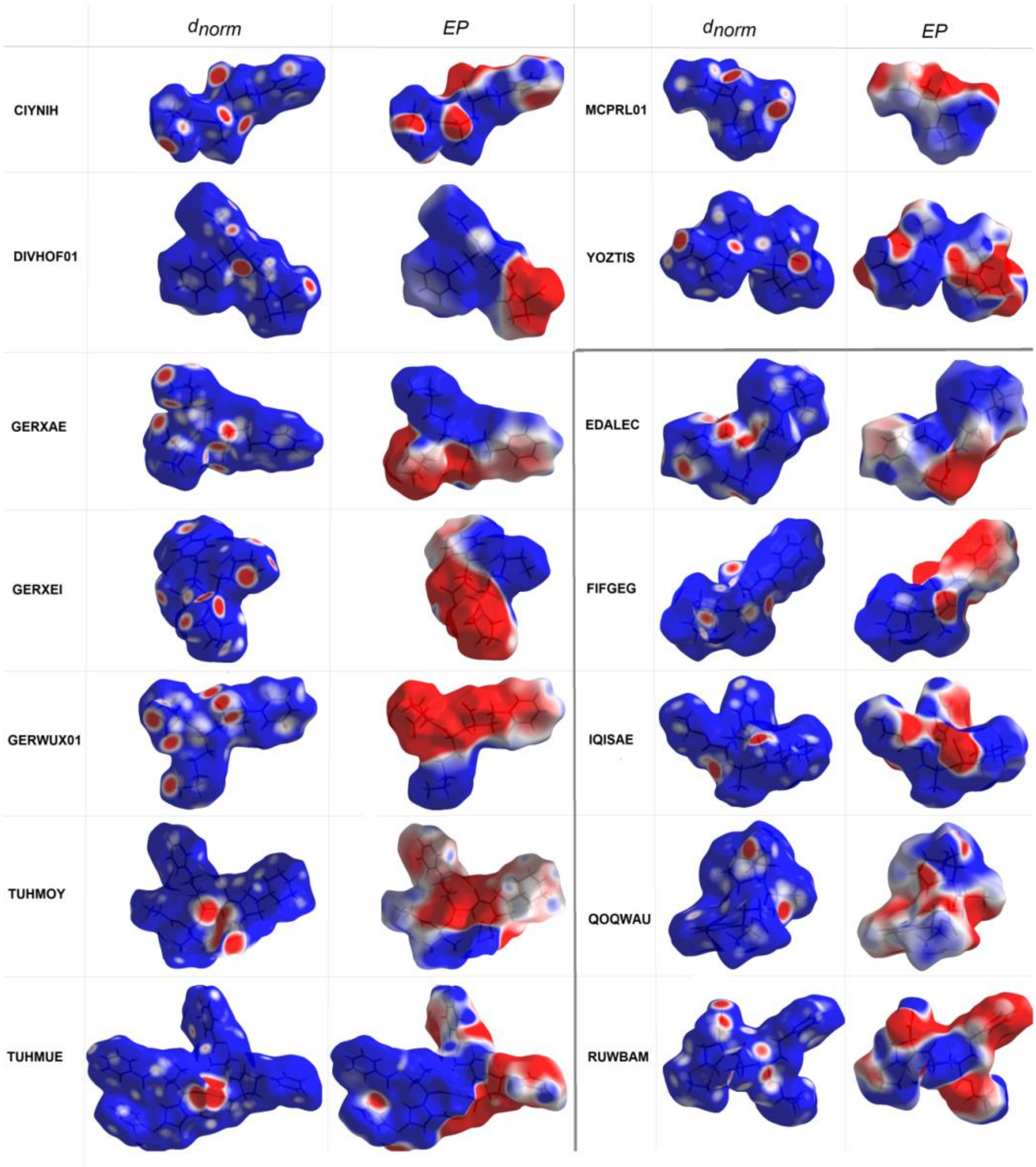

Figure S11. HS d_{norm} and EPs of proline-based (CIYNIH, DIVFOF01, GERXAE, GERXEI, GERWUX01, TUHMOY, TUHMUE) and other modified-proline-based ACEI (EDALEC, FIFGEG, IQISAE, QOQWAU, RUWBAM) crystals, retrieved from the CSD.

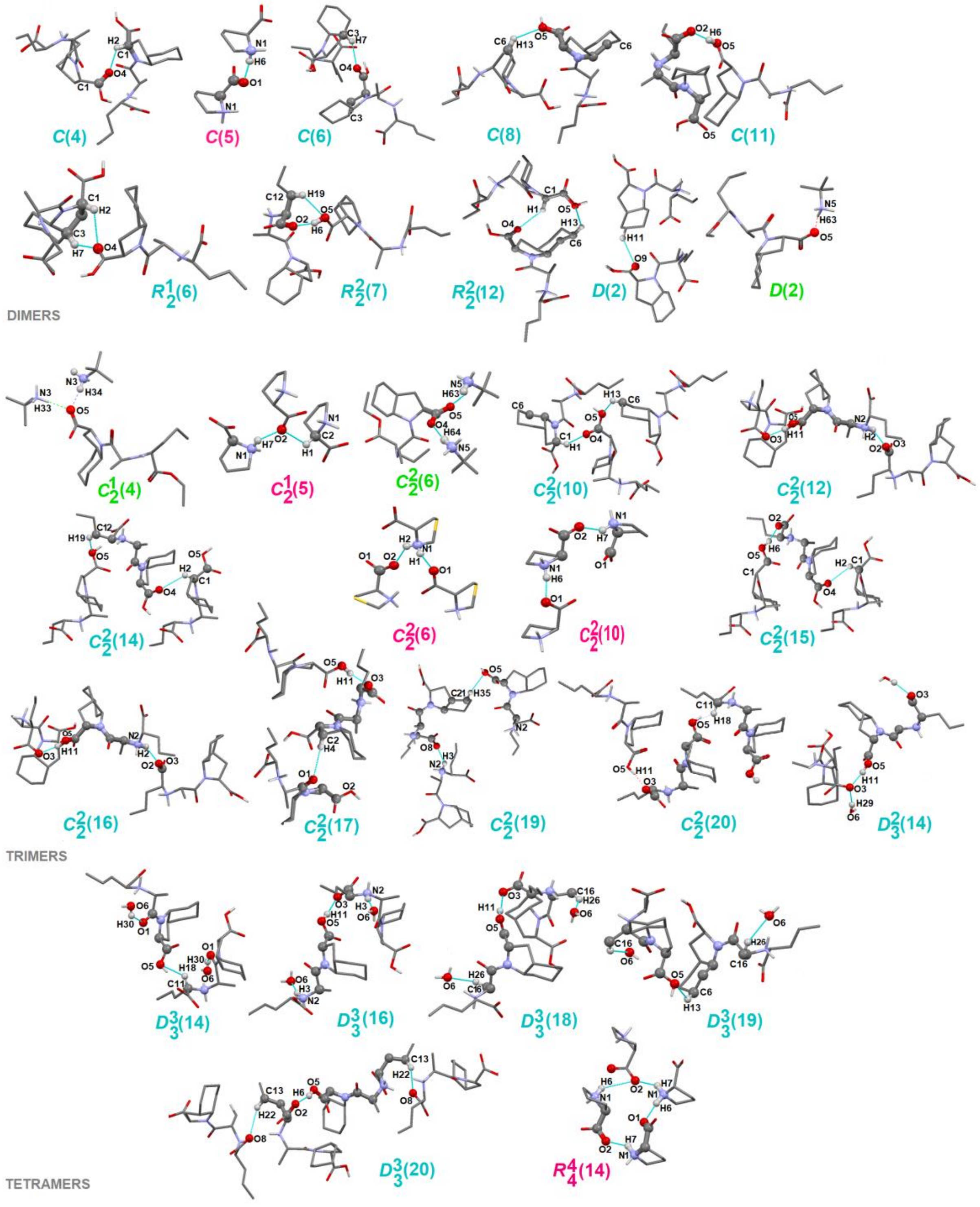

Figure S12. Proline-based synthons in perindopril-derived and proline crystal structures (with the graph-set notations), resulting from three types of proline-based tectons (I-marked in blue, II-green and III-pink).

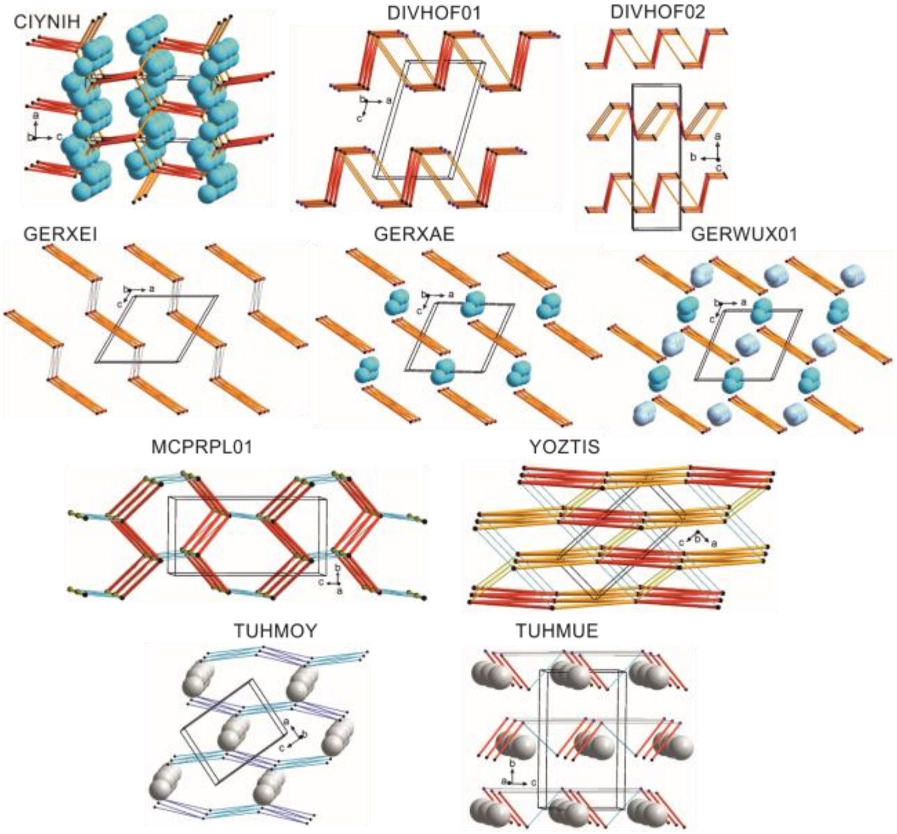

Figure S13. Simplified hydrogen-bonded networks showing LSAMs in proline-based ACEI crystals. The black or blue dots represent a center of gravity of the molecule or ion, orange-shaded lines stand for O-H···O or N-H···O hydrogen bonds, blue, grey, and yellow lines for C-H···O, C-H···π and C-H···S contacts, respectively. Blue and gray spheres represent the positions of water molecules and sodium cations, respectively.

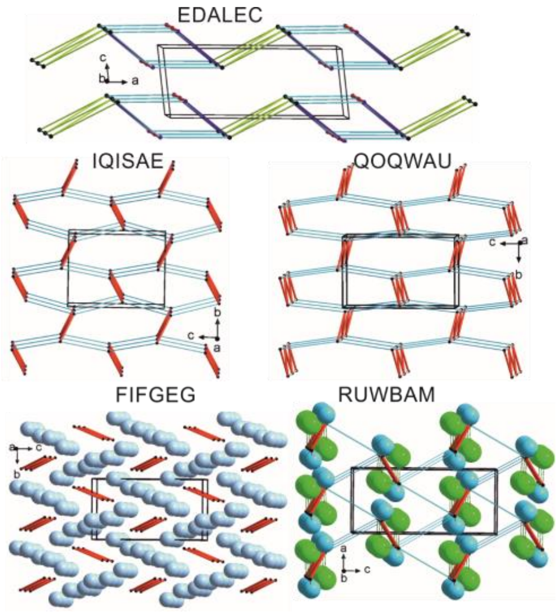

Figure S14. Simplified hydrogen-bonded networks in other modified proline-based ACEI structures. The black and blue dots represent a center of gravity of molecules or ions. Red lines stand for O-H···O or N-H···O hydrogen bonds, blue and green lines depict C-H···O contacts and gray lines N-H···π contacts. Water molecules or methanol oxygen atoms are represented as blue spheres. Chlorine anions are presented as green spheres.

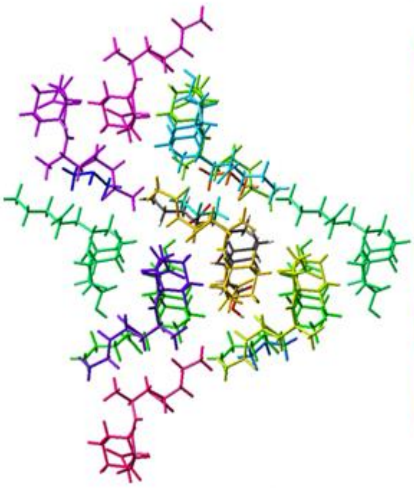

Interaction Energies (kJ/mol)
R is the distance between molecular centroids (mean atomic position) in Å.

Total energies, only reported for two benchmarked energy models, are the sum of the four energy components, scaled appropriately (see the scale factor table below)

	N	Symop	R	Electron Density	E_ele	E_pol	E_dis	E_rep	E_tot
	1	-	6.85	HF/3-21G	-89.7	-44.7	-53.2	56.8	-122.4
	1	-	6.01	HF/3-21G	0.0	nan	0.0	0.0	nan
	1	-	9.27	HF/3-21G	0.0	0.0	0.0	0.0	0.0
	1	-	9.36	HF/3-21G	-12.5	-5.9	-23.1	14.1	-26.0
	1	-	8.30	HF/3-21G	-61.6	-35.1	-43.7	46.2	-87.5
	2	x+1/2, -y+1/2, -z	6.91	HF/3-21G	-103.0	-42.0	-47.0	89.2	-102.4
	2	x, y, z	10.35	HF/3-21G	0.5	-1.2	-7.3	2.1	-5.1
	1	-	5.67	HF/3-21G	-12.4	-1.2	-3.1	0.0	-16.2
	2	-x, y+1/2, -z+1/2	11.15	HF/3-21G	-12.4	-1.2	-3.1	0.0	-16.2
	1	-	9.46	HF/3-21G	1.0	-0.0	-0.2	0.0	0.8
	1	-	11.41	HF/3-21G	0.5	-1.2	-7.3	2.1	-5.1
	1	-	9.41	HF/3-21G	-6.0	-3.9	-24.4	15.9	-17.7
	1	-	13.97	HF/3-21G	1.5	-1.1	-9.6	5.6	-3.3
	1	-	13.30	HF/3-21G	-1.5	-0.2	-1.7	0.0	-3.2
	1	-	14.19	HF/3-21G	-0.8	-0.1	-1.4	0.0	-2.2

Energy Model	k_ele	k_pol	k_disp	k_rep
CE-HF ... HF/3-21G electron densities	1.019	0.651	0.901	0.811
CE-B3LYP ... B3LYP/6-31G(d,p) electron densities	1.057	0.740	0.871	0.618

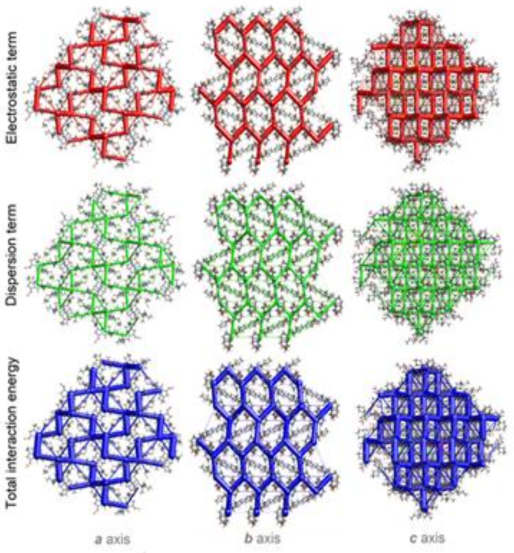

Figure S15. On the left: Color coding of neighbouring molecules in perindoprilat structure (BECWIR) in relations to the central molecule (gray). A view along ***b***-axis (for interpretation of the references to colour in this figure legend, the readers are referred to the web version of this article). In the middle: Interaction energies of the molecular pairs related to energy frameworks of this perindoprilat structure (Scale factors for benchmarked energy models). On the right: Energy frameworks corresponding to the electrostatic and dispersion energy components, and total energy framework along *a*, *b* and *c*-axis in crystal packing of perindoprilat structure (the tube size: 300).

	N	Symop	R	Electron Density	E_ele	E_pol	E_dis	E_rep	E_tot
	2	-x, y+1/2, -z	16.15	B3LYP/6-31G(d,p)	1.6	-0.2	-5.5	1.1	-2.5
	2	x, y, z	15.18	B3LYP/6-31G(d,p)	9.7	-3.3	-17.1	21.3	6.1
	2	x, y, z	16.30	B3LYP/6-31G(d,p)	1.0	-14.1	-8.3	7.6	-12.0
	2	-x, y+1/2, -z	6.68	B3LYP/6-31G(d,p)	-18.2	-9.4	-59.6	46.4	-49.4
	2	x, y, z	5.95	B3LYP/6-31G(d,p)	-119.4	-97.4	-75.2	113.9	-193.4
	2	-x, y+1/2, -z	9.71	B3LYP/6-31G(d,p)	1.5	-16.7	-24.8	25.2	-16.8
	2	-x, y+1/2, -z	10.90	B3LYP/6-31G(d,p)	0.7	-101.9	-21.3	60.2	-56.0
	2	-x, y+1/2, -z	13.77	B3LYP/6-31G(d,p)	-146.6	0.0	-5.9	10.3	-153.8

Energy Model	k_ele	k_pol	k_disp	k_rep
CE-HF ... HF/3-21G electron densities	1.019	0.651	0.901	0.811
CE-B3LYP ... B3LYP/6-31G(d,p) electron densities	1.057	0.740	0.871	0.618

GERXEI

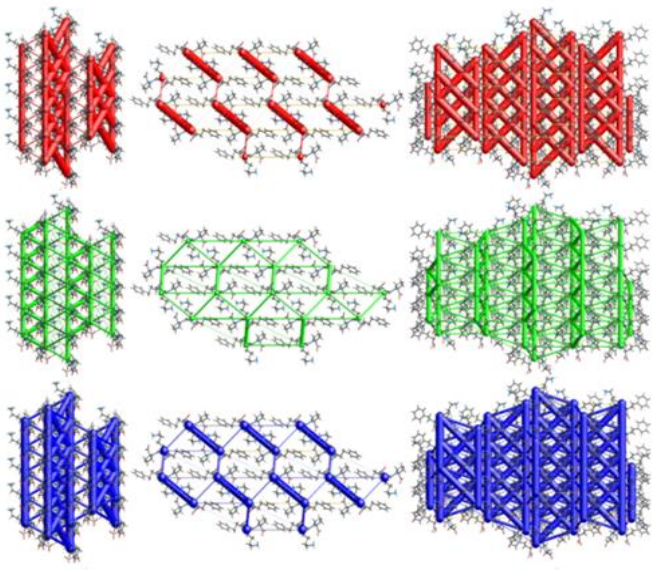

Figure S16. Interaction energies of lisinopril (GERXEI) (Scale factors for benchmarked energy models) and energy frameworks corresponding to the electrostatic and dispersion energy components, and total energy framework along *a*, *b* and *c*-axis (the tube size: 300).

	N	Symop	R	Electron Density	E_ele	E_pol	E_dis	E_rep	E_tot
(red)	2	x, y, z	7.48	B3LYP/6-31G(d,p)	-3.5	-4.0	-51.0	27.2	-34.2
(yellow)	2	-x+1/2, -y, z+1/2	11.34	B3LYP/6-31G(d,p)	-9.2	-3.4	-18.1	11.7	-20.7
(green)	2	-x, y+1/2, -z+1/2	12.19	B3LYP/6-31G(d,p)	-4.2	-1.1	-15.8	7.9	-14.2
(cyan)	2	x+1/2, -y+1/2, -z	9.39	B3LYP/6-31G(d,p)	-6.3	-1.7	-21.3	10.6	-19.9
(blue)	2	x+1/2, -y+1/2, -z	6.66	B3LYP/6-31G(d,p)	-120.4	-33.2	-69.7	165.8	-110.1
(magenta)	2	-x+1/2, -y, z+1/2	12.30	B3LYP/6-31G(d,p)	-2.2	-0.3	-14.7	6.5	-11.4

Energy Model	k_ele	k_pol	k_disp	k_rep
CE-HF ... HF/3-21G electron densities	1.019	0.651	0.901	0.811
CE-B3LYP ... B3LYP/6-31G(d,p) electron densities	1.057	0.740	0.871	0.618

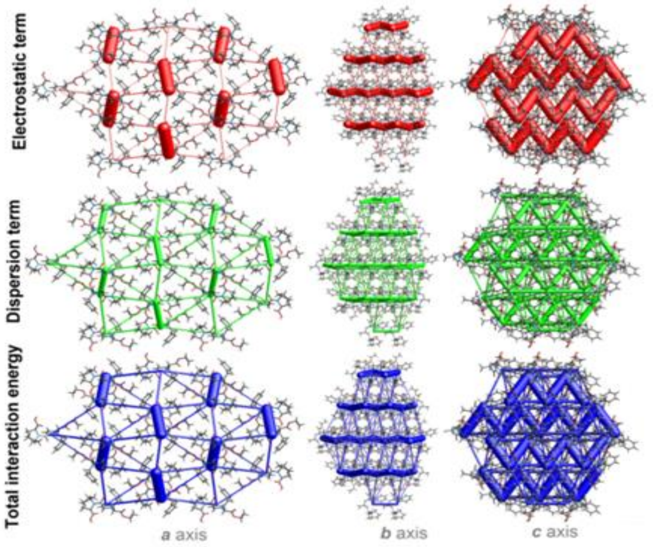

Figure S17. Interaction energies of ramipril (QOQWAU), (Scale factors for benchmarked energy models) and energy frameworks corresponding to the electrostatic and dispersion energy components, and total energy framework along *a*, *b* and *c*-axis (the tube size: 300).

Interaction Energies (kJ/mol)
R is the distance between molecular centroids (mean atomic position) in Å.
Total energies, only reported for two benchmarked energy models, are the sum of the four energy components, scaled appropriately (see the scale factor table below)

	N	Symop	R	Electron Density	E_ele	E_pol	E_dis	E_rep	E_tot
(red)	2	x+1/2, -y+1/2, -z	10.91	B3LYP/6-31G(d,p)	-7.5	-1.7	-39.4	28.6	-25.8
(yellow)	2	-x+1/2, -y, z+1/2	6.94	B3LYP/6-31G(d,p)	-70.2	-18.5	-67.7	160.9	-47.5
(green)	2	x+1/2, -y+1/2, -z	10.82	B3LYP/6-31G(d,p)	-14.4	-5.2	-25.0	15.1	-31.5
(light green)	2	x, y, z	7.67	B3LYP/6-31G(d,p)	0.1	-5.1	-38.4	19.3	-25.2
(light blue)	2	-x, y+1/2, -z+1/2	10.47	B3LYP/6-31G(d,p)	-6.4	-1.7	-24.6	11.2	-22.6
(violet)	2	-x+1/2, -y, z+1/2	10.94	B3LYP/6-31G(d,p)	0.7	-1.1	-18.1	7.8	-11.1
(magenta)	2	-x, y+1/2, -z+1/2	13.06	B3LYP/6-31G(d,p)	-1.0	-0.1	-7.2	2.2	-6.0

Energy Model	k_ele	k_pol	k_disp	k_rep
CE-HF ... HF/3-21G electron densities	1.019	0.651	0.901	0.811
CE-B3LYP ... B3LYP/6-31G(d,p) electron densities	1.057	0.740	0.871	0.618

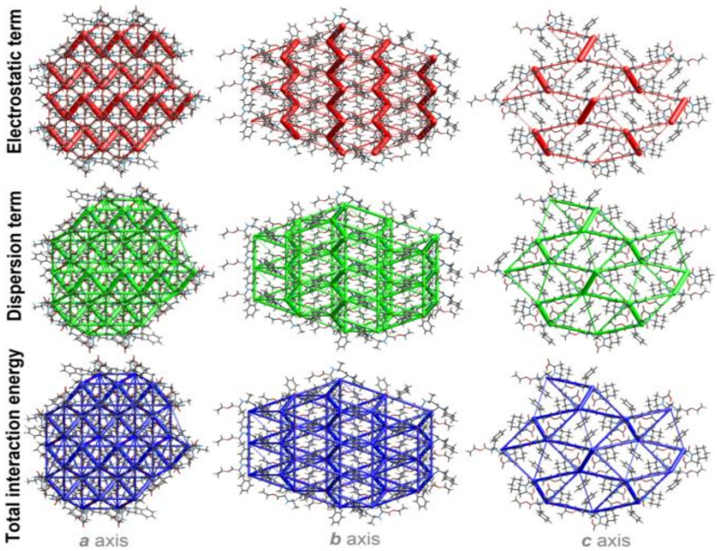

Figure S18. Interaction energies of the molecular pairs related to energy frameworks of **IQISAE01** (Scale factors for benchmarked energy models) and energy frameworks corresponding to the electrostatic and dispersion energy components, and total energy framework along *a*, *b* and *c*-axis (the tube size: 300).

TABLES

Table S1. Proline-based tectons with relevant structures, retrieved from the CSD.

CSD code	*Name*	*Formula*
	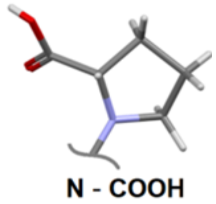	
AFIXOE [Rajalakshmi; 2013]	*N*-*t*-butoxycarbonyl-α-(2-fluorobenzyl)-*L*-proline $C_{17}H_{22}FNO_4$	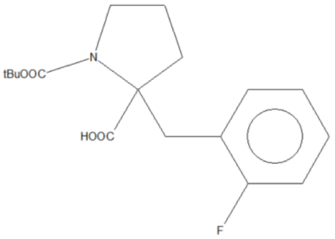
AMOLPR [Benedetti; 1979]	*N*-*t*-amyloxycarbonyl-*L*-proline $C_{11}H_{19}NO_4$	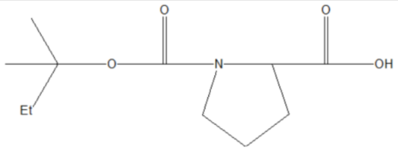
ASEYIG [Hursthouse, 2003]	1-(5-nitropyridin-2-yl)pyrazolidin-2-carboxylic acid $C_{10}H_{11}N_3O_4$	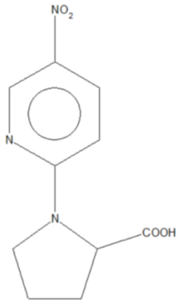
AWAVOK(01) [Huy, 2011]	1-(t-butoxycarbonyl)-3-vinylproline $C_{12}H_{19}NO_4$	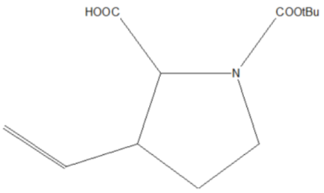
AWAVUQ [Huy, 2011]	1-((benzyloxy)carbonyl)-3-isopropylproline $C_{16}H_{21}NO_4$	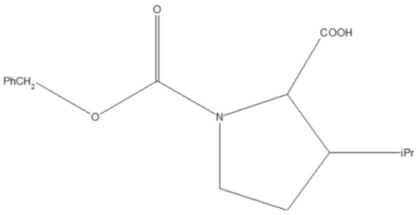
AXCPRO [Kartha, 1974]	$C_{21}H_{33}N_3O_6$ *t*-amyloxycarbonyl-*L*-prolyl-*L*-prolyl-*L*-proline	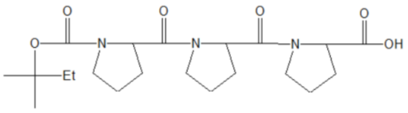
BECWIR	*See scheme 1, 2*	
BEMKIN [Bavoso, 1982]	$C_{20}H_{31}N_3O_6$ *N*-*t*-butyloxycarbonyl-*D*-prolyl-*D*-prolyl-*L*-proline	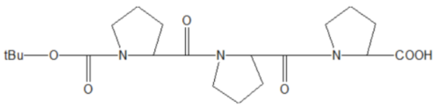

BGPLGP [Ueki, 1971]	$C_{28}H_{38}BrN_5O_8$, H_2O, $C_4H_8O_2$<br>o-bromobenzyloxycarbonyl-glycyl-*L*-prolyl-*L*-leucyl-*L*-proline ethyl acetate solvate monohydrate	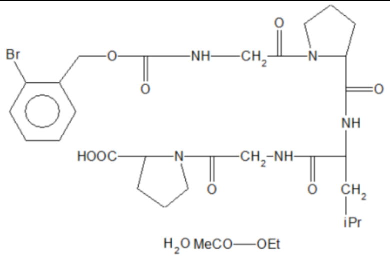
BGPLGQ [Bando, 1978]	$C_{28}H_{39}N_5O_8$, $2H_2O$<br>*N*-benzyloxycarbonyl-glycyl-*L*-prolyl-*L*-leucyl-glycyl-*L*-proline dihydrate	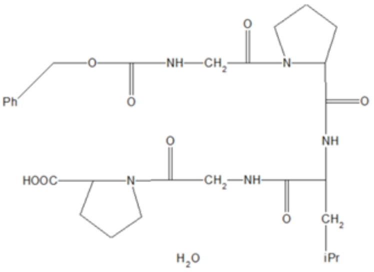
BIMFUY(10) [Busetti, 1982]	$C_{16}H_{18}N_2O_4$, $0.5H_2O$<br>(*Z*)-*N*-acetyldehydprophenylalanyl-*L*-proline hemihydrate	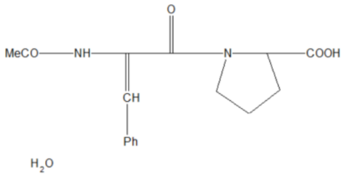
BIRYEG [Martin, 1999]	$C_{10}H_{15}NO_4$<br>(5*R*,6*S*,9*S*)-9-carbozy-5-hydroxy-5-methyl-1-azabicyclo[4.3.0]nonan-2-one	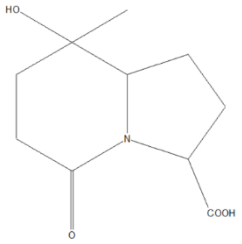
BOCAPR [Nair, 1981]	$C_{25}H_{29}N_3O_6$, H_2O<br>benzyloxycarbonyl-*L*-alanyl-*D*-phenylalanyl-*L*-proline monohydrate	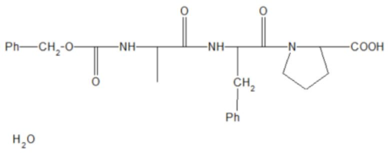
BOCGLP(01,02) [Benedetti, 1976]	$C_{12}H_{20}N_2O_5$<br>*t*-butyloxycarbonylglycyl-*L*-proline	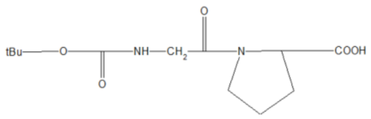
BOCPRO(01) [Kamwaya, 1981]	$C_{15}H_{24}N_2O_5$<br>*t*-butyloxycarbonyl-*L*-prolyl-proline	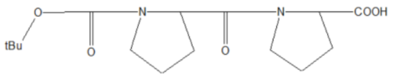
BOZBOI [Ranatunga, 2009]	$C_{13}H_{21}NO_4$<br>1-(*t*-butyloxycarbonyl)hexahydrocyclopenta[b]pyrrole-6a(1H)-carboxylic acid	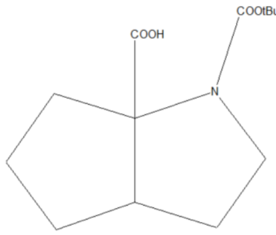
BUCWUT [Roy, 2014]	$C_{24}H_{23}ClN_2O_6$, CH_4O, H_2O<br>1-((1-(4-chlorobenzoyl)-5-methoxy-2-methyl-1H-indol-3-yl)acetyl)-4-hydroxyproline methanol solvate monohydrate	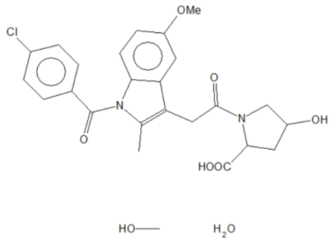
BXALPR(10) [Kamwaya, 1982]	$C_{13}H_{22}N_2O_5$, H_2O<br>*t*-butyloxycarbonyl-(l)-alanyl-(*L*)-proline monohydrate	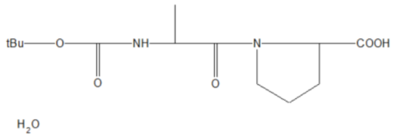

BXCGPL [Kojima, 1978]	$C_{15}H_{18}N_2O_5$<br>benzyloxycarbonylglycyl-*D*,*L*-proline	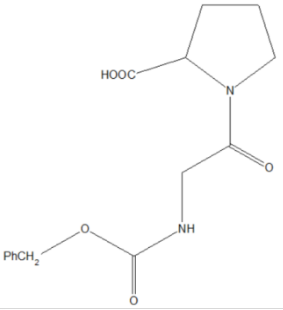
BXCGPR [Meesakul, 2020]	$C_{13}H_{10}O_2$<br>6-(2-phenylethenyl)-2H-pyran-2-one	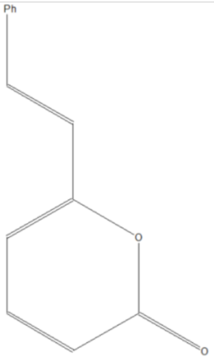
BXGLPR [Tanaka, 1977]	$C_{15}H_{18}N_2O_5$<br>benzyloxycarbonylglycyl-*L*-proline	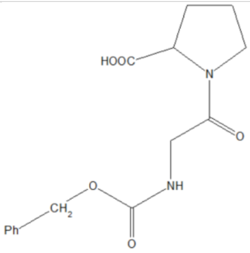
BXPROL(01,02) [Benedetti, 1974]	$C_{10}H_{17}NO_4$<br>*N*-(t-butyloxycarbonyl)-*L*-proline	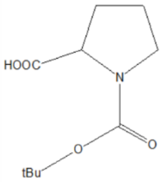
BZCPRO(01,11) [Galitskii, 1977]	$C_{18}H_{22}N_2O_5$<br>*N*-benzyloxycarbonyl-*L*-prolyl-*L*-proline	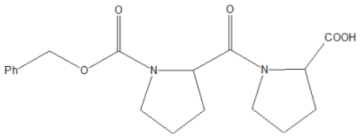
CAHDIA [Carreras, 2011]	$C_{10}H_{12}BrNO_5$, H_2O<br>4-bromo-6-oxotetrahydro-3H,8H-pyrano[3,4-d]pyrrolo[1,2-c][1,3]oxazolo-8-carboxylic acid monohydrate	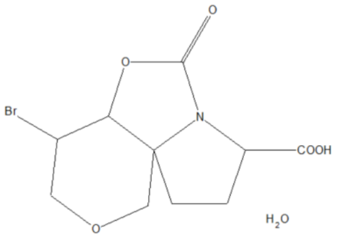
CALFIE [Smith, 1981]	$C_{20}H_{35}N_3O_6$, H_2O<br>*t*-butoxycarbonyl-leucyl-α-aminoisobutyryl-proline monohydrate	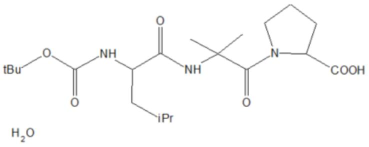
CIDJIJ(01) [Seijas, 2007]	$C_6H_{10}N_2O_3$<br>(2*S*)-1-carbamoylpyrrolidine-2-carboxylic acid (*N*-carbamoyl-*L*-proline)	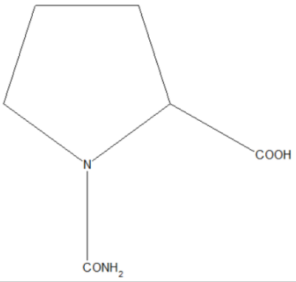
CIYNIH	*See scheme 1, 2*	

COSLAA<br>[Wojnarska, 2019]	$C_{12}H_{15}NO_4S$, $0.5C_6H_6$<br>(2*S*)-1-(4-methylphenyl)sulfonylpyrrolidine-2-carboxylic acid benzene solvate [*N*-p-tosyl-*L*-proline benzene solvate]	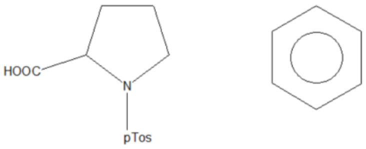
COVMOP<br>[Colapietro, 1985]	$C_{25}H_{38}N_4O_7$<br>*t*-butoxycarbonyl-*L*-prolyl-*D*-prolyl-*L*-prolyl-*D*-proline	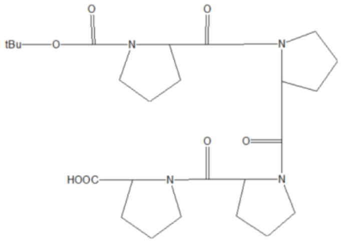
COWHEC<br>[Iminov, 2008]	$C_{18}H_{14}N_2O_4$, H_2O<br>2,3,4,5-tetrahydro-1,5-dioxo-4-phenylpyrrolo[1,2-a]quinazoline-3a(1H)-carboxylic acid monohydrate	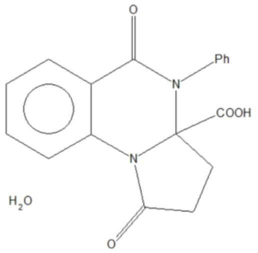
CURFEB<br>[Hanessian, 2009]	$C_{33}H_{43}NO_9$<br>6-((3,5-bis)benzyloxy)-4-hydroxytetrahydro-2H-pyran-2-yl)oxy)-1-(t-butoxycarbonyl)octahydro-1H-indole-2-carboxylic acid.	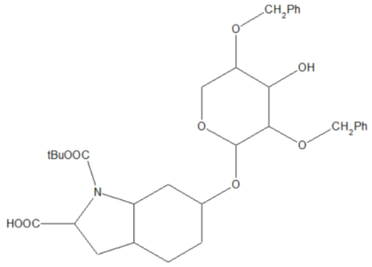
DASQEU<br>[Duggan, 2005]	$C_{10}H_{15}N_2O_3^+$, Cl^-<br>(2*S*,5*R*,9*S*)-6-amino-5-oxo-2,3,5,6,9,9a-hexahydro-1H-pyrrolo[1,2-a]azepine-3-carboxylate hydrochloride	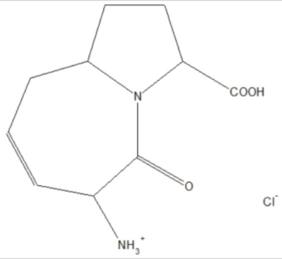
DAVHUF<br>[Delgado, 2012]	$C_6H_{10}N_2O_3$<br>*R*,*S*-1-carbamoylpyrrolidine-2-carboxylic acid [N-carbamoyl-*D*,*L*-proline]	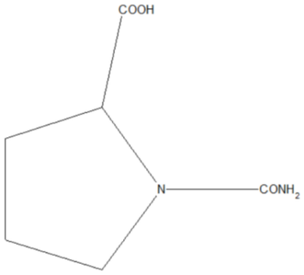
DIVHOF(01,02)	*See scheme 1, 2*	
EGUWEL<br>[Bendzinska, 2019]	$C_{25}H_{23}NO_3$<br>*N*-(triphenylacetyl)-*L*-proline	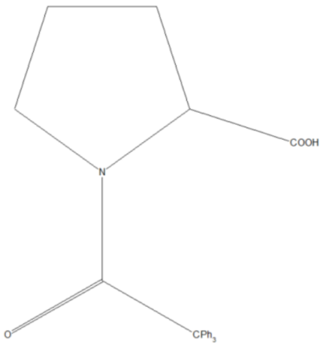

EJIHAG [Doi, 2002]	$C_{10}H_{16}FNO_4$<br>*N-t*-butoxycarbonyl-4-(*S*)-fluoroproline	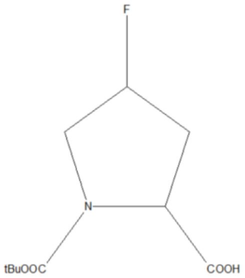
ELOBOW [Hanessian, 2003]	$C_9H_{13}NO_4S$<br>(8*S*)-1,1-dioxo-2,3,5q,6,7,8-octahydro-1H-1λ6-pyrrolo(1,2-b)(1,2)thiazepine-8-carboxylic acid	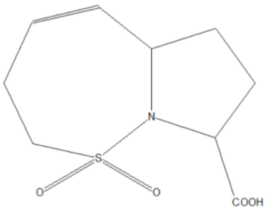
EMITIE [Shoulders, 2010]	$C_{10}H_{19}NO_5$<br>(2*S*,4*S*)-1-(t-butoxycarbonyl)-4-methoxyproline	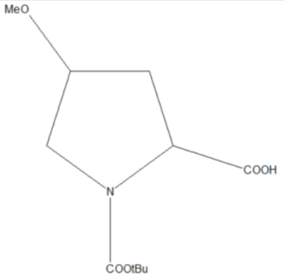
EMOBUE [Bhuyan, 2011]	$C_{18}H_{28}N_2O_6Se_2$<br>1,1`-(diselane-1,2-diylbis(2-methyl-1-oxopropane-3,1-diyl))-bis(pyrrolidine-2-carboxylic acid	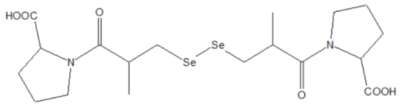
EPUWAP [Kolstoe, 2014]	$C_{16}H_{24}N_2O_6$, H_2O<br>1,1`-(1,6-dioxohexane-1,6-diyl)dipyrrolidine-2-carboxylic acid monohydrate	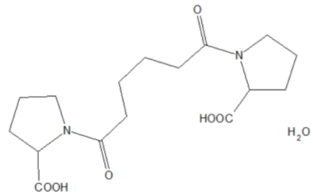
EVOPOU [Messeakul, 2020]	$C_{13}H_{10}O_2$<br>6-(2-phenylelethenyl)-2H-pyran-2-one [(E)-6-styrylpyran-2-one]	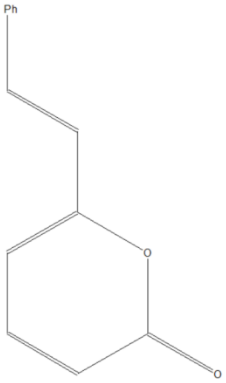
EZEYOX(01) [Hua, 2002]	$C_{12}H_{12}N_2O_2$, $2(H_2O)$<br>1,2,3,9-tetrahydropyrrolo(2,1-b)quinazolin-1-carboxylic acid dihydrate [(-)-linarinic acid dihydrate]	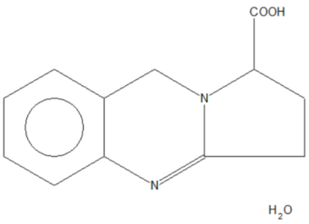
FAMVUM [Luparia, 2011]	$C_{21}H_{15}BrN_2O_6$<br>(1*R*,2*S*,5*S*)-2-(4-bromobenzyl)-3-(4-nitrobenzoyl)-4-oxo-3-azabicyclo[3.2.0]hept-6-ene-2-carboxylic acid	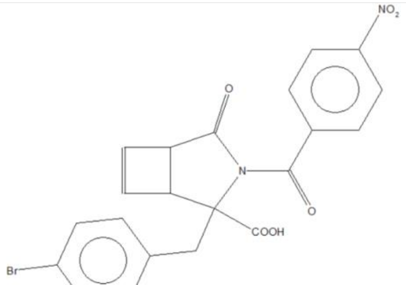

FEFKEI	*See scheme 1, 2*	
FEYMOM [Zhang, 2004]	$C_{11}H_{13}NO_5$, H_2O<br>3-thoxy-4-((2`*S*)-2`-carboxypyrrolidin-1-yl)-3-cyclobutane-1,2-dione monohydrate	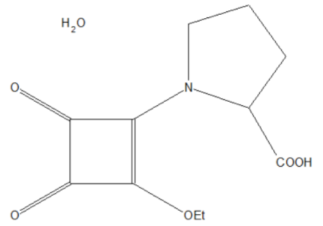
FEZNON [Blankley, 1987]	$C_{16}H_{18}BrNO_3$<br>(2α,3αβ, 7 αβ)-1-(3-bromobenzoyl)octahydro-1H-indole-2-carboxylic acid	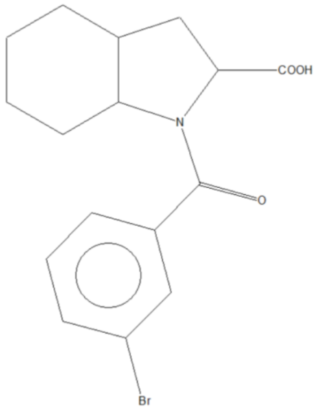
FIFGEG	*See scheme 1, 2*	
FIZXAN [Wu, 1987]	$C_{17}H_{21}N_3O_6$<br>*N*-benzyloxycarbonyl-glycyl-glycyl-*L*-proline	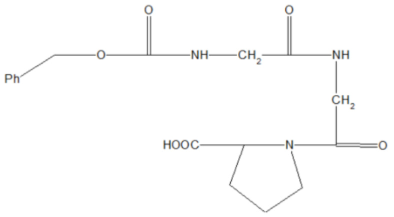
GABNUV [Forbes, 2016]	$C_{16}H_{20}INO_5$<br>1-(*t*-butoxycarbonyl)-4-(4-iodophenoxy)proline	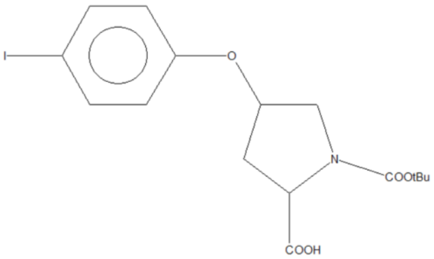
GABPAD [Forbes, 2016]	$C_{26}H_{22}INO_5$, CH_4O<br>1-((9H-fluoren-9-ylmethoxy)carbonyl)-4-(4-iodophenoxy)proline methanol solvate	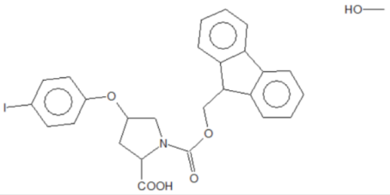
GESKEU [Colapietro, 1986]	$C_{25}H_{38}N_4O_7$<br>*t*-butoxycarbonyl-*D*-prolyl-*L*-prolyl-*D*-prolyl-*L*-proline	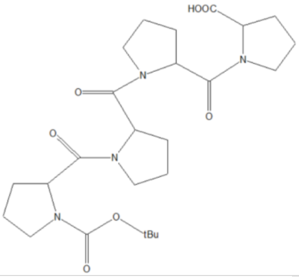
GIWROU [Vamos, 2007]	$C_{17}H_{21}NO_5$<br>syn-3a-hydroxy-1-(4-methoxybenzyl)-2-oxo-octahydro-7aH-indole-7a-carboxylic acid	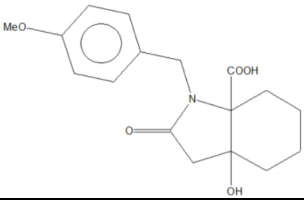

GIXLAC [Kale, 2014]	$C_{30}H_{38}N_8O_9S_2$<br>1-((2-((2-azido-2-methylpropanoyl)amino)phenyl)sulfonyl)prolyl-N-(2-((2-carboxypyrrolidin-1-yl)sulfonyl)phenyl)-2-methylalaninamide	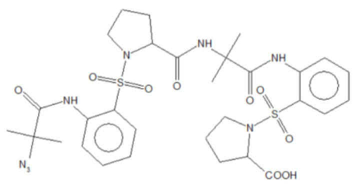
GLHPRC [Garbay, 1980]	$C_{12}H_{18}N_2O_5$<br>*N*-acetyl-*L*-prolyl-*L*-4-hydroxyproline	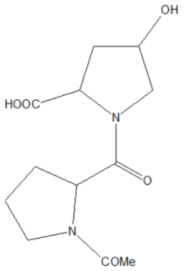
GOYGOT [Li, 2019]	$C_{13}H_{15}NO_3$<br>1-(phenylacetyl)proline	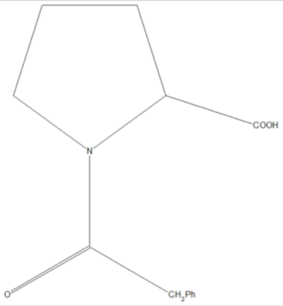
HATWOO [Dijnovic, 1994]	$C_{21}H_{23}N_5O_4$, H_2O<br>*N*-((*Z*)-2-benzylamino-3-(4,6-dimethyl-2-pyrimidinylamino)propenoyl)-*L*-proline monohydrate	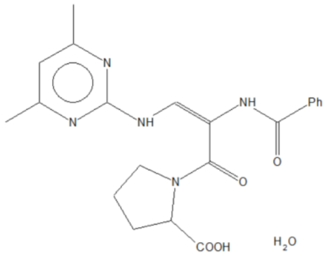
ICOQIB [Vartak, 2006]	$C_{20}H_{34}N_2O_4$<br>(2R,5R,1`R)-2-t-butyl-5-(2`,2`-dimethyl-1-(2S-carboxy-1-pyrrolidinyl)propyl)-1-aza-3-oxabicyclo(3.3.0)octan-4-one	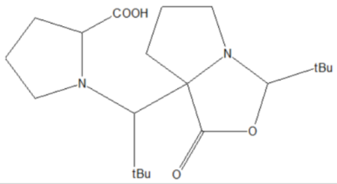
IQISAE(01,02)	*See scheme 1, 2*	
JAWLIC [Panneerselvam, 1990]	$C_{16}H_{20}N_2O_5$<br>benzyloxycarbonyl-*L*-alanyl-*L*-proline	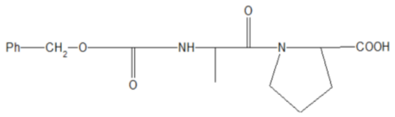
JEGXOI [Viossat, 1990]	$C_{14}H_{24}Cl_2N_2O_8Pt_{2S2}$, $3H_2O$<br>bis((µ2-5-oxo-prolinato-N,O)-chloro-(dimethylsulfoxide-S)-platinum(ii) trihydrate	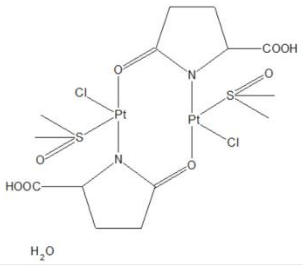
JEKPIY [Hausin, 1990]	$C_{24}H_{26}N_2O_5$<br>(5S)-5-benzamido-4-oxo-6-phenylhexanoyl-L-proline [Ketoace]	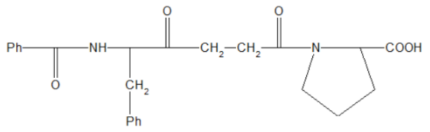
JEKPOE [Hausin, 1990]	$C_{18}H_{21}NO_4S$<br>(1S,2R)-1-((2-(benzoylthio)cyclopentyl)carbonyl)-L-proline	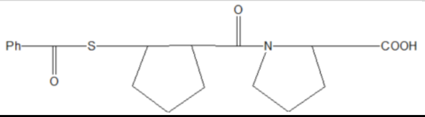

JUJLUL [Meesakul, 2020]	$C_{13}H_{10}O_2$<br>6-(2-phenylethenyl)-2H-pyran-2-one [(E)-6-styrylpyran-2-one]	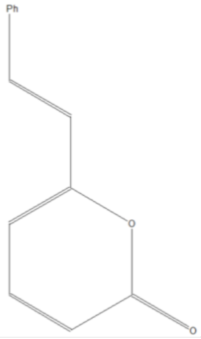
KENYEI [Matsumura, 2006]	$C_{18}H_{25}NO_3$<br>Trans-N-formyl-α`-(2,4,6,-triethylphenyl)-L-proline	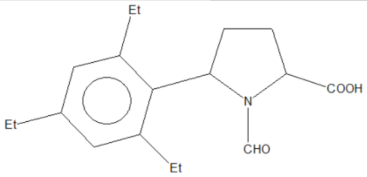
KETSEJ [Stoykova, 2013]	$C_{28}H_{38}N_4O_8$, C_2H_6OS<br><br>6-(2-phenylethenyl)-2H-pyran-2-one	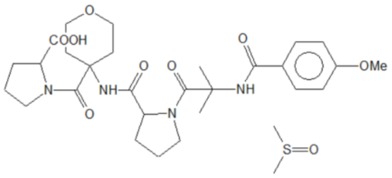
KOKRIL [Kemp, 1991]	$C_{13}H_{18}N_{2}O_{4}S$<br>(1S,4S,7S,10S)-12-acetyl-4-carboxy-3,12-diaza-2-oxo-9-thiatricyclo(8.2.1.0 ^{3,7})tridecane	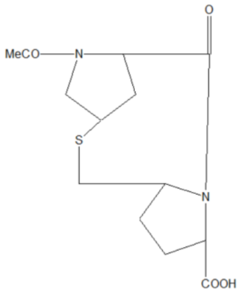
KULROB [Aubry, 1991]	$C_7H_{12}N_2O_4$<br>1-[(methoxycarbonyl)amino]proline	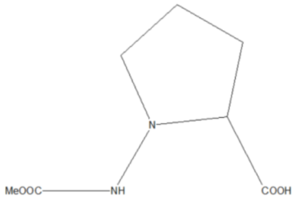
KUSTIB [Finke, 1992]	$C_{16}H_{20}N_2O_9S$<br>3-(acetoxymethyl)-2-((2(S)-carboxypyrrolidino)carbonyl)-7α-methoxy-8-oxo-5-thia-1-azabicyclo(4.2.0)oct-2-one 5,5-dioxide [L-658, 758]	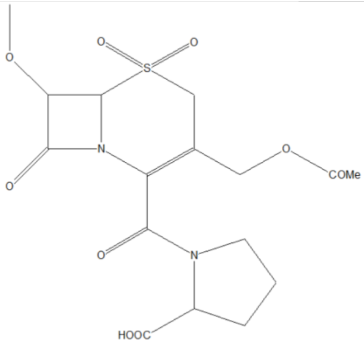
LAFDUU [Naveen, 2016]	$C_{20}H_{20}N_3O_5^+$, H_2O, Cl^-<br>1-((3-carboxy-9H-b-carbolin-2-ium-1-yl)acetyl)proline chloride monohydrate	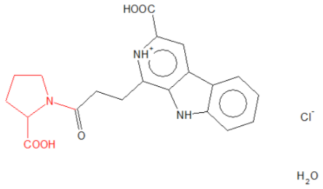
LAWJOI [Milne, 1993]	$C_{24}H_{33}N_3O_6$<br>*N-t*-Butoxycarbonyl-prolyl-phenylalanyl-proline	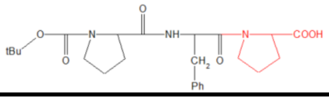

LEPSOP(01) [Odame, 2015]	$C_{13}H_{14}N_2O_3S$, H_2O<br>1-(benzoylcarbamothioyl)pyrrolidine-2-carboxylic acid monohydrate { 1-(benzoylcarbamothioyl)proline monohydrate}	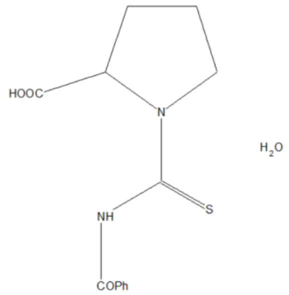
LIKMUP [Rajalakshmi, 2013]	$C_7H_{11}NO_3$, H_2O<br>1-acetyl-2-pyrrolidine carboxylic acid monohydrate {*N*-acetyl-*L*-proline monohydrate}	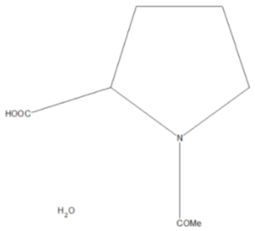
LIZCED [Krasnov, 2008]	$C_{18}H_{18}N_2O_5S$<br>(2*S*,4*S*)-4-((4-Methylbenzenesulfonyl)amino)-5-oxo-1-phenylproline	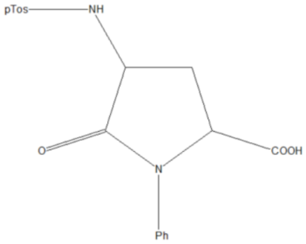
LIZCUT [Krasnov, 2008]	$C_{19}H_{14}N_2O_5$, CH_4O<br>(2*S*,4*S*)-5-oxo-1-phenyl-4-phthalimidoproline methanol solvate	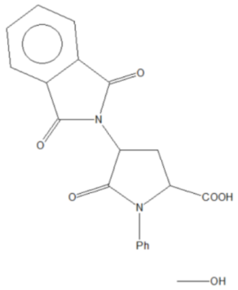
LODTIH(01) [Xinghua, 2014]	$C_{11}H_{17}NO_4$<br>2-(t-butoxycarbonyl)-2-azabicyclo[3.1.0]hexane-3-carboxylic acid	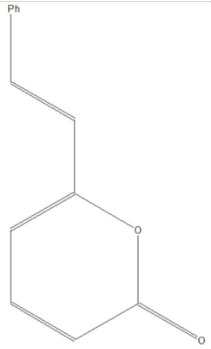
LODTON [Hanessian, 1997]	$C_{11}H_{17}NO_4$<br>*cis*-(4*S*,5*S*)-methano-N-t-butoxycarbonylproline	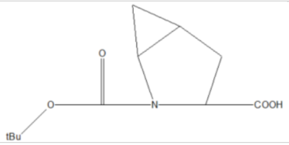
LUZWEK [Chandrarekha, 2016]	$C_{20}H_{19}NO_4$, H_2O<br>1-((9H-fluoren-9-ylmethoxy)carbonyl)proline monohydrate	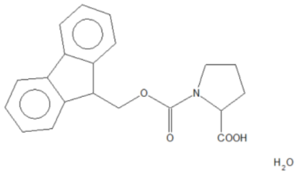
MAMROH [Abdaoui, 2000]	$C_7H_{12}ClN_3O_5S$<br>*N*-(*N'*-nitroso-*N'*-(2-chloroethyl)sulfamoyl)proline	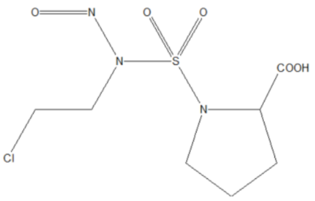

MAYYOB [Basak, 2005]	$C_{21}H_{20}N_2O_4$, H_2O<br>5-oxo-1-(2-oxo-1,4(*S*)-diphenylazetidin-3(*R*)-ylmethyl)pyrrolidine-2-carboxylic acid monohydrate	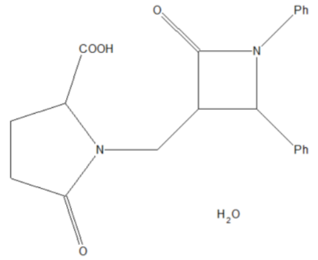
MCPRPL(01)	*See scheme 1, 2*	
MOGQOO [Del Vale, 2002]	$C_{11}H_{16}F_3NO_4$<br>*N*-(*t*-Butoxycarbonyl)-(4*S*)-trifluoromethyl-*L*-proline	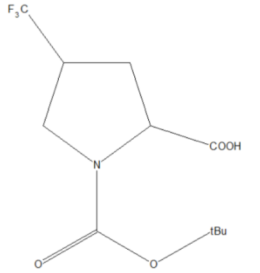
MOLNEG [Bernardi, 2002]	$C_{15}H_{20}N_2O_7$<br>*t*-butoxycarbonyl-*L*-pyroglutamato-*L*-pyroglutamatic acid	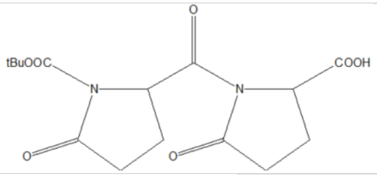
MPRPRO [Eckle, 1978]	$C_{11}H_{18}N_2O_3$, H_2O<br>*N*-Methylprolyl-proline monohydrate	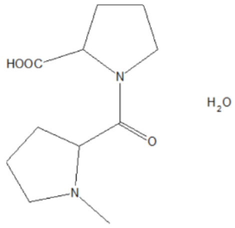
MYPYCA [Baert, 1975]	$C_{11}H_{14}N_2O_6$<br>*D*,*L*-Methylene-bis(*N*-pyrrolid-2-one-5-carboxylic acid)	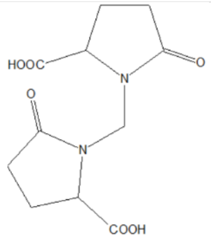
NAHYPL [Hospital, 1979]	$C_7H_{11}NO_4$, H_2O<br>*N*-Acetyl-4-hydroxy-*L*-proline monohydrate	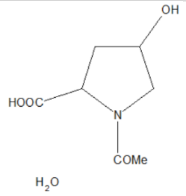
NAMYIJ [Milne, 1997]	$C_{19}H_{26}N_2O_6$, $2(H_2O)$<br>*t*-butyloxycarbonyl-tyrosyl-proline dihydrate	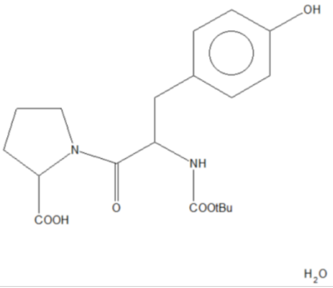
NAMYOP [Milne, 1997]	$C_{19}H_{26}N_2O_5$, $0.7(Na^+)$, $0.7(Cl^-)$<br>*t*-Butyloxycarbonyl-phenylalanyl-proline sodium chloride	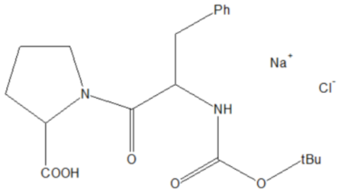

NERNII [Fonari, 2006]	$C_5H_8N_2O_4$<br>(4*R*)-4-Hydroxy-1-nitroso-*L*-pyrrolidine-2-carboxylic acid	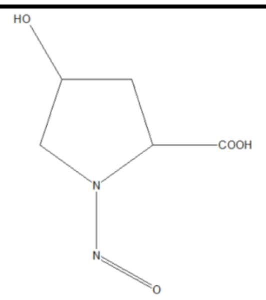
NODZOW [Dai, 2008]	$C_{28}H_{26}N_2O_5$<br>1-benzyl-5-oxo-4-(phenylamino)-3-(([3-phenylprop-2-en-1-yl]oxy)carbonyl)proline	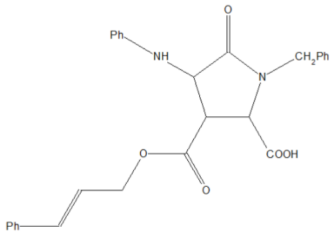
NPRPLN [Kamwaya, 1981]	$C_8H_{13}NO_3$<br>*N*-Propionyl-proline	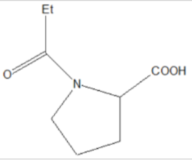
NUKSES [De Poli, 2009]	$C_{14}H_{17}NO_4$<br>1-((benzyloxy)carbonyl)-2-methylproline	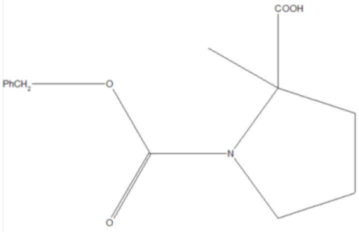
OZUQEH [Kubyshkin, 2012]	$C_{11}H_{15}F_2NO_4$<br>2-(*t*-butoxycarbonyl)-6,6-difluoro-2-azabicyclo[3.1.0]hexane-3-carboxylic acid	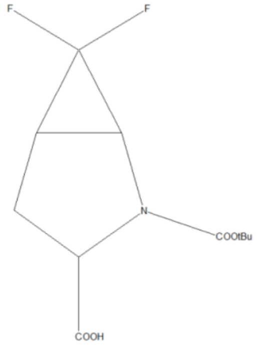
PABSOB [Cheng, 2002]	$C_{18}H_{20}Cl_2N_2O_5$<br>(5*R*,8*S*)-3-(2,6-Dichlorophenyl)-1-oxa-2,7-diazaspiro[4,4]non-2-ene-7-butoxycarbonyl-8-carboxylic acid	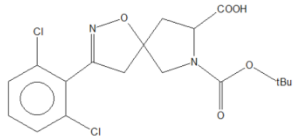
PABSUH [Cheng, 2002]	$C_{29}H_{26}N_2O_6$<br>(5S,8S)-3-(2-Methoxyphenyl)-1-oxa-2,7-diaza-spiro[4,4]non-2-ene-7-(fluorene-9-methoxycarbonyl)-8-carboxylic acid	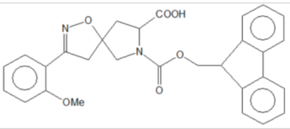
PABTAO [Cheng, 2002]	$C_{19}H_{24}N_2O_6$, CH_4O<br>(5*S*,8*S*)-3-(4-methoxyphenyl)-1-oxa-2,7-diazaspiro[4,4]non-2-ene-7-(t-butoxycarbonyl)-8-carboxylic acid methanol solvate	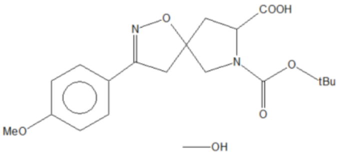
PACWUL [Wasserman, 1992]	$C_{13}H_{20}N_2O_5$, 0.5(CH_4O), 0.5(H_2O)<br>7-(1-Hydroxyethyl)-1-hydroxy-6-methyl-9a-carboxy-8-oxoazetidino(1,2-c)perhydropyrrolo(1,2-a)pyrimidine methanol solvate hemihydrate	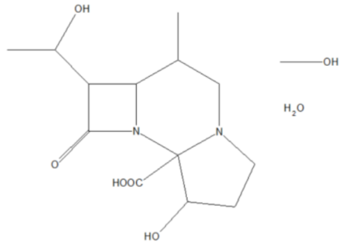

PATZES [Wang, 2017]	$C_{20}H_{20}BrNO_6$<br>(2*S*,3a*R*,4*R*,7*R*,7a*S*)-1-((benzyloxy)carbonyl)-5-bromo-7-ethyl-8-oxo-2,3,3a,4,7,7a-hexahydro-1H-4,7-(epoxymethano)indole-2-carboxylic acid { 3-((benzyloxy)carbonyl)-11-bromo-1-ethyl-9-oxo-8-oxa-3-azatricyclo[5.2.2.02,6]undec-10-ene-4-carboxylic acid}	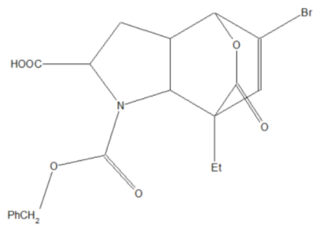
PEFLOB [Baldwin, 1993]	$C_{22}H_{22}N_2O_5$<br>3-Benzyl-4-benzyloxycarbonyl-8-carboxy-1,4-diazabicyclo(3.3.0)octan-2-one	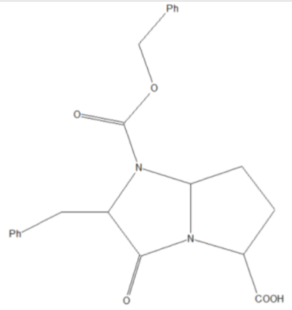
PIGYEL [Hack, 2013]	$C_{12}H_{19}NO_4$<br>1-(*t*-butoxycarbonyl)-4-vinylproline	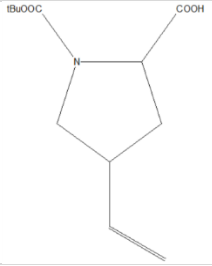
PIZTIE [Venkateshan, 2019]	$C_{14}H_{15}NO_3$<br>1-(3-phenylacryloyl)proline	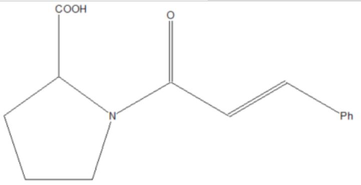
POKKAD(01-16) [Lubben, 2014]	$C_7H_{11}NO_4$, H_2O<br>1-acetyl-4-hydroxyproline monohydrate	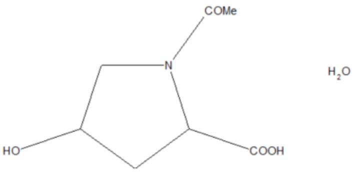
QECWUT [Mykhailiuk, 2017]	$C_{13}H_{12}FNO_3$<br>2-(benzenecarbonyl)-4-fluoro-2-azabicyclo[2.1.1]hexane-1-carboxylic acid	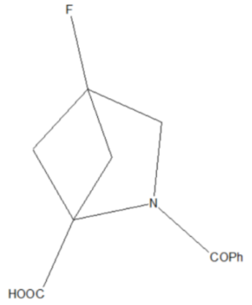
QILCET [Kraatz, 1999]	$C_{16}H_{17}FeNO_3$<br>*N*-ferrocenoylproline	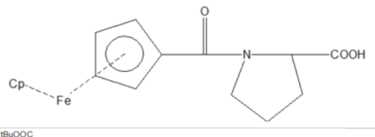
QOMHAE [Poulie, 2019]	$C_{18}H_{23}NO_6$<br>1-(*t*-butoxycarbonyl)-3-[carboxy(phenyl)methyl]-*L*-proline	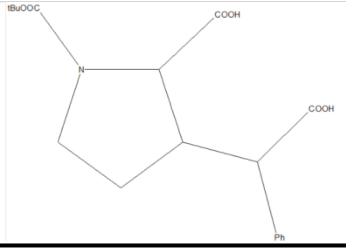

QOMHEI [Poulie, 2019]	$C_{14}H_{23}NO_6$<br>1-(t-butoxycarbonyl)-3-(1-carboxypropyl)proline	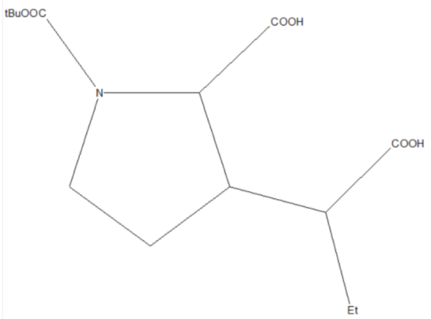
QOQTAR [Camus, 2001]	$C_{18}H_{20}N_2O_6$<br>1,1'-(p-tolyl)-bis(5-oxopyrrolidine-2-carboxylic acid)	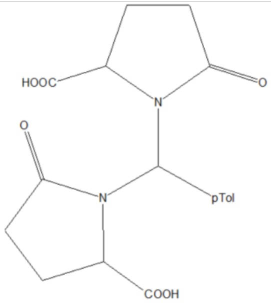
QOQWAU	*See scheme 1, 2*	
QQQARG [Ueki, 1971]	$C_{28}H_{38}BrN_5O_8$<br>(*p*-bromobenzyloxycarbonyl)-glycyl-*L*-prolyl-*L*-leucyl-glycyl-*L*-proline	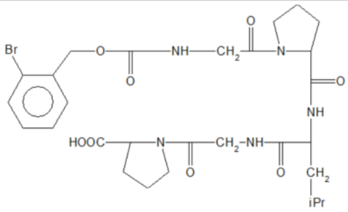
RACSEU [Bunch, 2003]	$C_{13}H_{19}NO_6$<br>(+-)-*N*-*t*-Butoxycarbonyl-2-azanorbornane-3-exo,5-endo-dicarboxylic acid	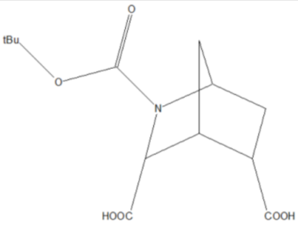
RARNAA [Koskinen, 2005]	$C_{14}H_{25}NO_4$<br>(2*S*)-N-t-butoxycarbonyl-trans-4-t-butyl-*L*-proline	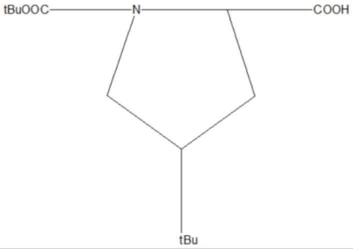
REFYIK [Guenoun, 1997]	$C_{20}H_{24}N_2O_6$<br>3S,8aR-6-benzyl-4,6,7,8a-tetrahydro-7-t-butoxycarbonylpyrrolidino(1,2-a)piperazine-5,8-dione-3-carboxylic acid	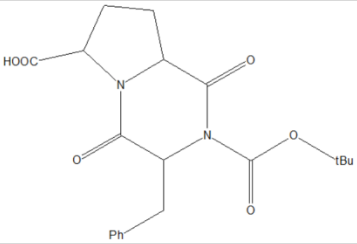
ROKPAI [Ray, 1997]	$C_{15}H_{12}ClNO_3S$<br>1-(*p*-chlorophenyl)-5-oxo-3-(2-thienyl)pyrrolidine-2-carboxylic acid	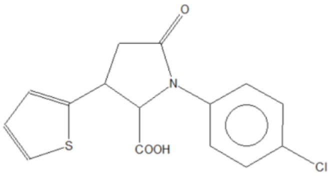
RONCEF [Light, 2019]	$C_{16}H_{24}N_2O_6$, H_2O<br>1-[6-(2-carboxypyrrolidin-1-yl)-6-oxohexanoyl]pyrrolidine-2-carboxylic acid monohydrate	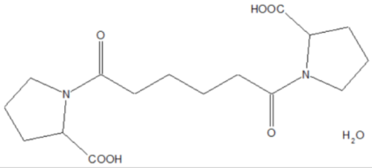
RUWBAM	*See scheme 1, 2*	

RUWVOW [Rayner, 2016]	$C_{10}H_{17}NO_2S$<br>(*S*)-1-(2,2-dimethylpropanethioyl)proline	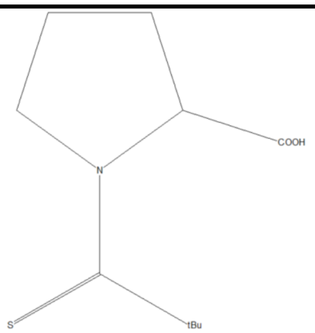
SACDIL [Freabult, 2010]	$C_{15}H_{13}NO_4$<br>rac-3-benzoyl-2-methyl-2-carboxy-3-azabicyclo(3.2.0)hept-6-ene-4-one	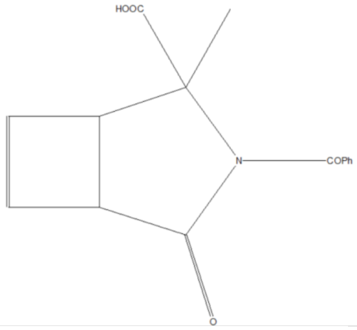
SEJSIJ [Emge, 1990]	$C_{12}H_{16}N_2O_6$<br>1,1'-(1,2-Ethanediyl)-bis(*L*-pyroglutamic acid)	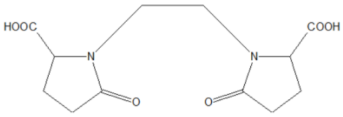
SIWBUV [Pascard, 1991]	$C_{17}H_{28}N_2O_5$, $2(C_2H_6O)$<br>(2*S*,3a*S*,7a*S*)-1-(*N*-(1(*S*)-carboxy-n-butyl)-(*S*)-alanyl)-2-carboxyperhyroindole ethanol solvate	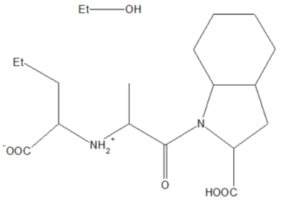
SOWJUL [Wilhelm, 2014]	$C_{37}H_{47}BrN_6O_8$, C_2H_3N<br>*N*-(4-bromobenzoyl)hexaproline acetonitrile solvate	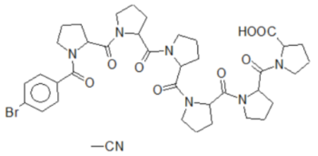
SUGFAE [Stavyskyi, 2020]	$C_{15}H_{12}N_4O_4$<br>3-methyl-2,8-dioxo-7,8-dihydro-2H-pyrrolo[1,2-a][1,2,4]triazino[2,3-c]quinazoline-5a(6H)-carboxylic acid	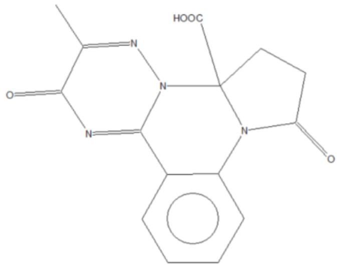
SUMZOP [Sasaki, 1994]	$C_{11}H_{17}NO_6$<br>*N*-*t*-Butoxycarbonyl-pyrrolidine-2,3-dicarboxylic acid	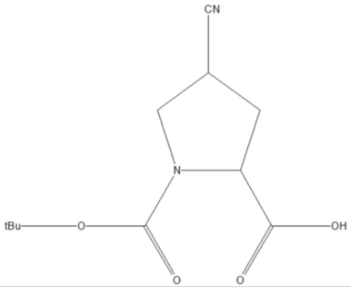
TALBUD [Webb, 1991]	$C_{11}H_{16}N_2O_4$<br>(4*S*)-1-(*t*-butoxycarbonyl)-4-cyano-*L*-proline	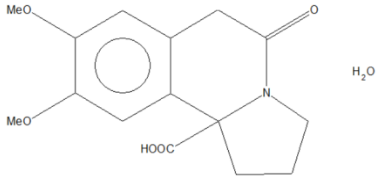

TIWZUV [Kumar, 2008]	$C_{15}H_{17}NO_5$, H_2O<br>8,9-dimethoxy-5-oxo-2,3,5,6-tetrahydropyrrolo[2,1-a]isoquinoline-10b(1H)-carboxylic acid monohydrate	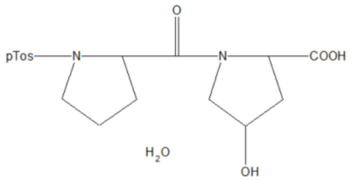
TPHPRO(01) [Fridrichson, 1962]	$C_{17}H_{22}N_2O_6S$, H_2O<br>tosyl-*L*-prolyl-*L*-hydroxyproline monohydrate	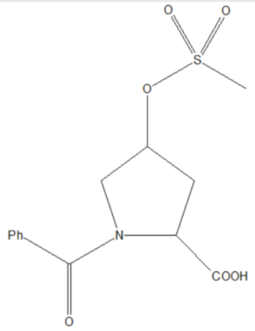
TUHMEO	*See scheme 1, 2*	
TUHMUE	*See scheme 1, 2*	
UHULOA [Reuter, 2015]	$C_{12}H_{19}NO_4$<br>1-(*t*-butoxycarbonyl)-2-vinylproline	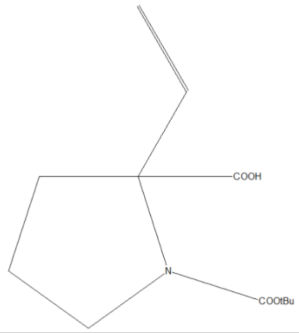
ULUDUA [Hanessian, 2003]	$C_{14}H_{15}NO_4$<br>(2*S*,3a*S*,8a*R*)-1,3a,8,8a-tetrahydro-2H-3-azacyclopenta(a)indene-2,3-dicarboxylic aicd 3-methyl ester	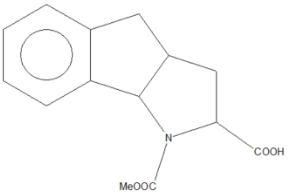
ULUFAI [Hanessian, 2003]	$C_{15}H_{17}NO_4$<br>(2*S*,3a*S*,9b*R*)-2,3,3a,4,5,9b-hexahydrobenzo(g)indole-1,2-dicarboxylic acid 1-methyl ester	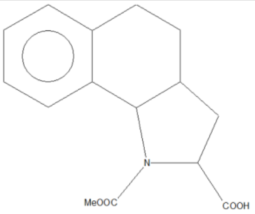
VOBSIR [Marsch, 2019]	$C_{15}H_{24}N_2O_6$, H_2O<br>1-(t-butoxycarbonyl)prolyl-4-hydroxyproline monohydrate	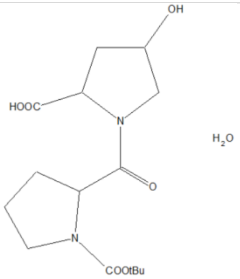
WEMLEI [Wang, 2017]	$C_{20}H_{20}BrNO_6$<br>(2R,3aS,4S,7S,7aR)-1-[(benzyloxy)carbonyl]-5-bromo-7-ethyl-8-oxo-2,3,3a,4,7,7a-hexahydro-1H-4,7-(epoxymethano)indole-2-carboxylic acid	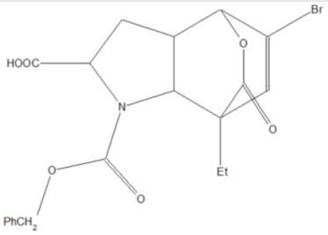

WADVOO [Yuan, 2010]	$C_{11}H_{19}NO_5$<br>(2*R*,4*R*)-1-(*t*-butoxycarbonyl)-4-methoxypyrrolidine-2-carboxylic acid	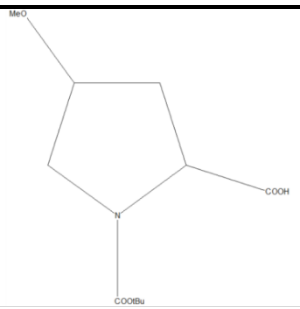
WETTOH [Liu, 2018]	$C_{16}H_{24}FNO_4$<br>6-fluoro-1-(hept-5-en-1-yl)-1-hydroxy-2-methyl-3-oxotetrahydro-1H-pyrrolizine-7a(5H)-carboxylic acid	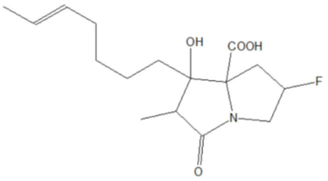
XETSOG [Torbeev, 2012]	$C_{11}H_{19}NO_4$<br>1-(*t*-butoxycarbonyl)-2-methylproline	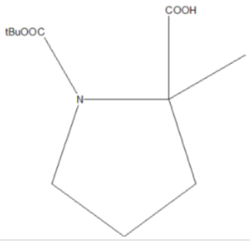
XEYXUX [Foley, 2015]	$C_9H_{13}N_5O_3$, 0.03(H_2O)<br>(3*S**,4a*S**)-3-(azidomethyl)-1-oxohexahydropyrrolo[1,2-c]pyrimidine-4a(5H)-carboxylic acid hydrate	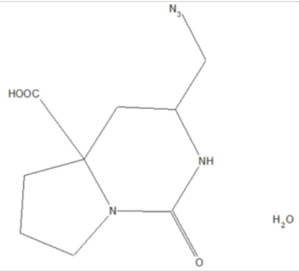
YACQAX [Baumann, 2016]	$C_{20}H_{27}NO_5$<br>1-(2,2-dimethylpropanoyl)-4-(methoxycarbonyl)-4-methyl-5-(2-methylphenyl)proline	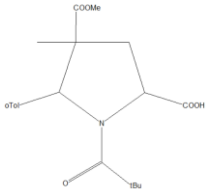
YAXZAZ [Pradelle, 2005]	$C_{22}H_{31}N_3O_6$, CH_4O, H_2O<br>benzyloxycarbonyl-valyl-a-aminoisobutyryl-proline methanol solvate monohydrate	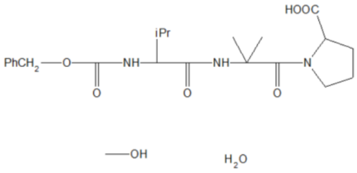
YAYMAO [Chen, 2012]	$C_{13}H_{12}N_2O_3$<br>(*S*)-1-(4-cyanobenzyl)-5-oxoproline	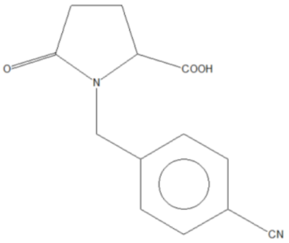
YILTUL [Linden, 2018]	$C_{29}H_{28}N_2O_5$, CH_4O<br>*N*-{[(9H-fluoren-9-yl)methoxy]carbonyl}phenylalanylproline methanol solvate	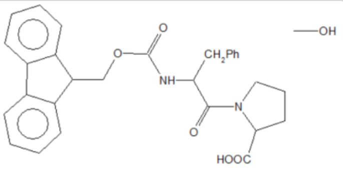
YOZTIS	*See scheme 1, 2*	

YUJLOF [Murkherjee, 2009]	$C_{71}H_{102}CoN_{14}O_{17}PS$, 14($H_2O$)<br>captopril-cobalamin tetradecahydrate	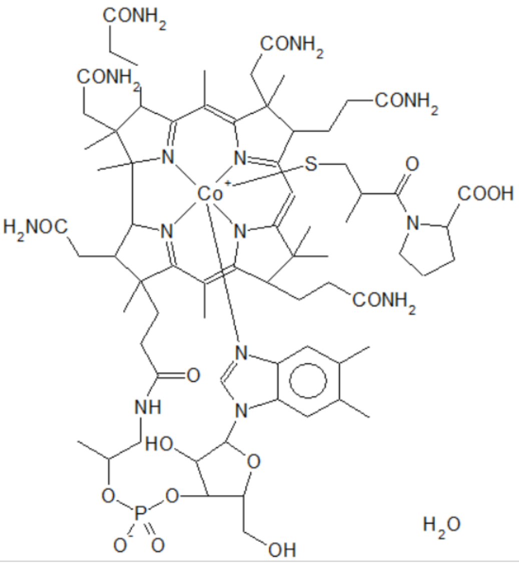
YUJLUL [Murkherjee, 2009]	$C_{71}H_{102}CoN_{14}O_{17}PS$, 12($H_2O$)<br>captopril-cobalamin dodecahydrate	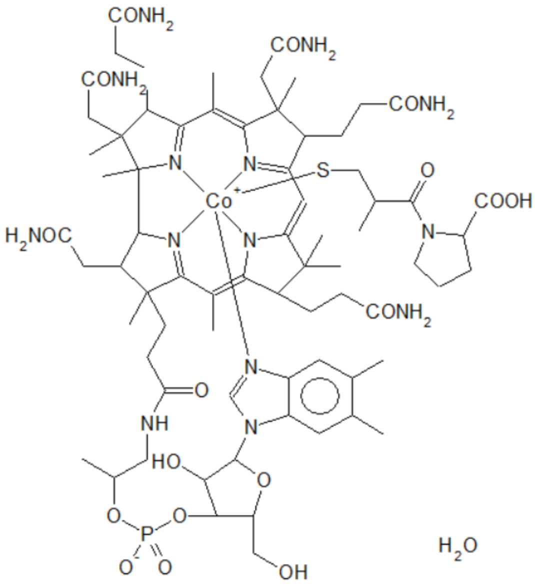
ZAPWUI [Kemp, 1995]	$C_{17}H_{23}N_3O_5S$<br>cyclo-prolyl-prolyl-proline-4-mercaptomethylcarbonyl	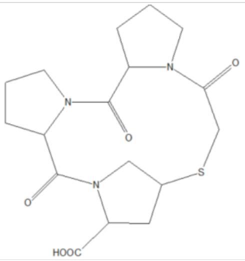
ZIZHAR [Oliver, 1995]	$C_{19}H_{26}N_2O_6$, H_2O<br>*N*-*t*-butoxycarbonyl-tyrosyl-proline monohydrate	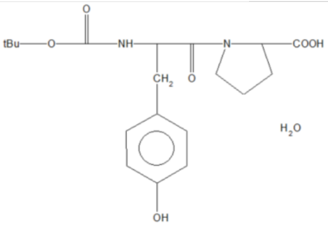
ZOKZUU [Ficker, 1995]	$C_{19}H_{29}NO_4$<br>1-(1-adamantyl)-1-methylethoxycarbonyl-*L*-proline	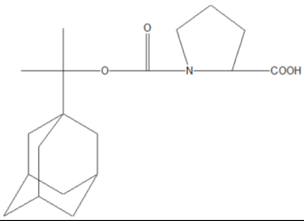

ZUMGIX<br>[Sagnard, 1995]	$C_{11}H_{15}NO_5$<br>*N*-*t*-butoxycarbonyl-3,4-methano-5-oxoproline	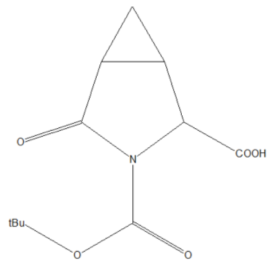
ZZZHKK(01)<br>[Jones, 1953;<br>Williams, 2007]	$C_{11}H_{14}N_2O_6$<br>methylene-bis(*N*-pyrrolidone-2-carboxylic acid)	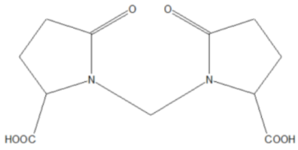
ZZZQNE(01)<br>[Sasada, 1961]	$C_{28}H_{39}N_5O_8$<br>(benzyloxycarbonyl)-glycyl-*L*-prolyl-*L*-leucyl-glycyl-*L*-proline	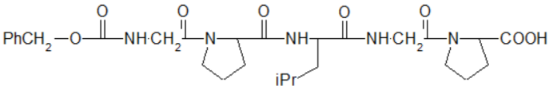

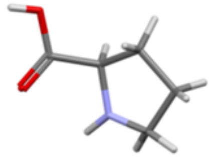

NH - COOH

ACXMPR<br>[Dupont, 1975]	$C_7H_{10}N_2O_5$<br>3*R*-(1'(*S*)-aminocarboxymethyl)-2-pyrrolidone-5(*S*)-carboxylic acid	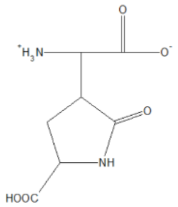
CMPROL [Rao, 1974]	$C_6H_{11}NO_2$<br>*cis*-*d*-methyl-*L*-proline	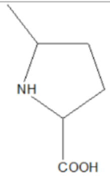
COPFEU<br>[Hammarsttrom, 2014]	$C_{12}H_{19}NO_5$<br>2-(3-t-butoxy-3-oxopropyl)-5-oxoproline solvate	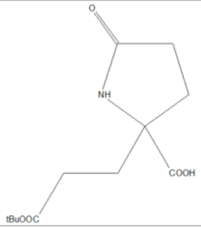
DHKAIN<br>[Flippen, 1976]	$C_{10}H_{17}NO_4$<br>dihydrokainic acid {2-carboxy-4-isopropyl-3-pyrrolidine-acetic acid}	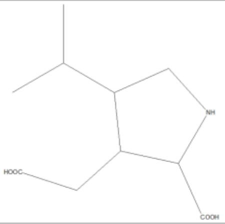
FIZDOH [Baird, 1987]	$C_5H_9NO_4$, H_2O<br>(2*R*,3*S*,4*R*)-3,4-dihydroxyproline monohydrate	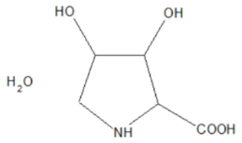

FUHRAC [Arman, 2009]	$C_{10}H_8N_2$, $2(C_5H_7NO_3)$<br>4,4'-bipyridine bis(pyroglutamic acid)	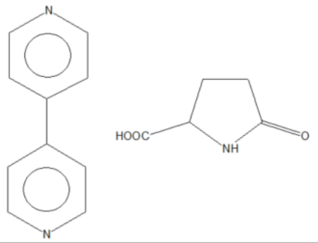
HIBPDC [Kass, 1977]	$C_9H_{15}NO_4$<br>3-hydroxy-3-isobutyl-2-pyrrolidone-5-carboxylic acid	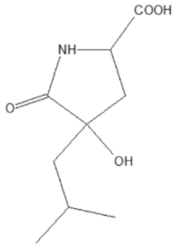
HUDRAC [Marques, 2019]	$C_{14}H_9ClF_3NO_2$, $2(C_5H_9NO_2)$<br>bis(proline) 6-chloro-4-(cyclopropylethynyl)-4-(trifluoromethyl)-1,4-dihydro-2H-3,1-benzoxazin-2-one<br>{bis(proline) (4S)-6-chloro-4-(2-cyclopropylethynyl)-4-(trifluoromethyl)-2,4-dihydro-1H-3,1-benzoxazin-2-one; bis(proline) efavirenz}	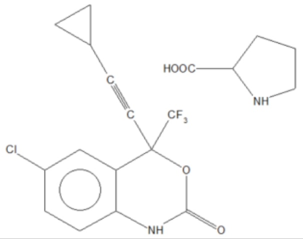
JEPLOF [Voissat, 1990]	$C_7H_{11}ClNO_4PtS$, $C_5H_7NO_3$, K^+, H_2O<br>potassium chloro-dimethylsulfoxide-(5-oxoprolinato(2-)-N,O)-platinum(ii) 5-oxoproline monohydrate	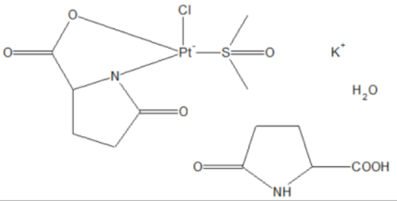
KAINAC [Flippen, 1976]	$C_{10}H_{15}NO_4$<br>kainic acid {2-carboxy-4-isopropenyl-3-pyrrolidine-acetic acid}	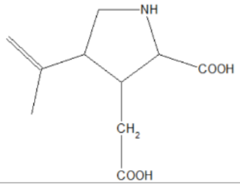
KAINAH [Watase, 1958]	$C_{10}H_{15}NO_4$, H_2O<br>kainic acid monohydrate	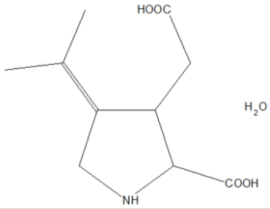
LGPYRG [Taira, 1977]	$C_5H_9NO_4$, $C_5H_7NO_3$, H_2O<br>*L*-glutamic acid *L*-pyroglutamic acid monohydrate	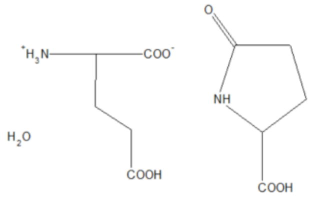
LPYGLU(01..**<u>09</u>**) [Issa, 2019]	$C_5H_7NO_3$<br>*L*-pyroglutamic acid {5-oxoproline}	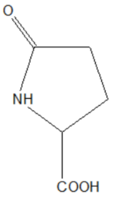
MNPROS (**<u>10</u>**) [Ciunik, 1981]	$(C_5H_{17}MnNO_{62}^{+})_n$, $n(O_4S_2^{-})$<br>catena-tetra-aqua-(m2-*D*,*L*-proline)-manganese(ii) sulfate	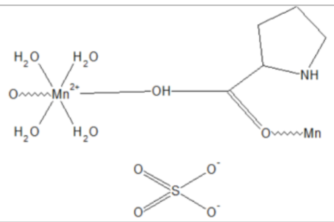

NASTUX<br>[Nomoto, 1992]	$C_{15}H_{21}NO_6$, $2(H_2O)$<br>(2*S*,3*S*,4*S*)-2-carboxy-4-(1-methyl-5(*R*)-carboxy-1(Z),3(E)-hexadienyl)pyrrolidine-3-acetic acid dihydrate<br>{domoic acid dihydrate}	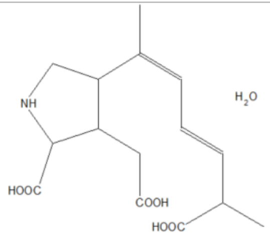
NASVAF<br>[Nomoto, 1992]	$C_{15}H_{21}NO_6$<br>isodomoic acid	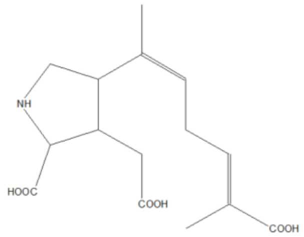
NAZHAZ<br>[Desphande, 2012]	$C_{21}H_{26}O_5$, $C_5H_9NO_2$, $C_5H_9NO_2$<br>(2*S*,3*R*,4*R*,5*S*,6*R*)-2-(3-(4-ethylbenzyl)-phenyl)-6-hydroxymethyl-tetrahydro-2H-pyran-3,4,5-triol bis(*L*-proline)	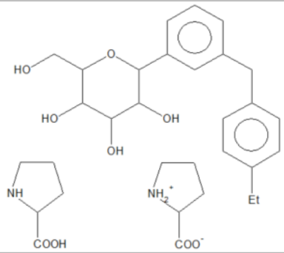
NAZLEG<br>[Fleishhacker, 1996]	$C_{12}H_{13}NO_3$<br>(4*S*,4*S*)-4-methyl-4-phenylpyroglutamic acid	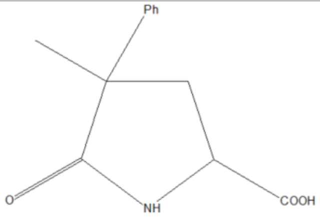
PAJFAH<br>[Klumberger, 1992]	$(C_{10}H_{13}LiN_2O_6)_n$<br>catena-((m3-*L*-pyroglutamato-O,O,O')-(hydrogen L-pyroglutamate-O)-lithium)	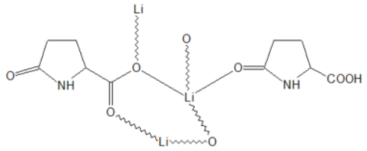
PENDAP<br>[Donohoe, 2012]	$C_{31}H_{43}NO_8Si$<br>3-(4-acetoxy-1-methoxy-3-methylbutyl)-2-(((t-butyl(diphenyl)silyl)oxy)methyl)-3-hydroxy-4-methyl-5-oxoproline	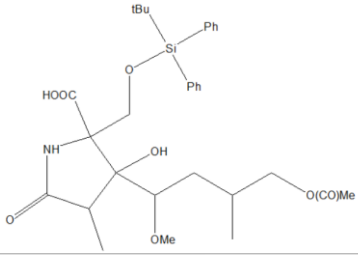
PROLPT<br>[Slyudkin, 1990]	$C_5H_{12}Cl_2N_2O_2Pt$<br>trans-amino-dichloro-(*L*-proline)-platinum(ii)	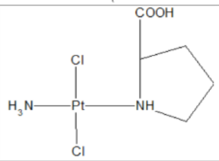
SIGJOK<br>[Tolmachova, 2018]	$C_6H_6F_3NO_3$<br>5-oxo-3-(trifluoromethyl)proline	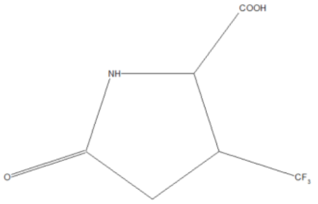
TUBQIQ<br>[Cantrell, 1996]	$C_{15}H_{17}NO_4$<br>(2*S*,*R*,3*S*,*R*,4*S*,*R*)-2-carboxy-4-(1-phenylethenyl)pyrrolidine-3-acetic acid	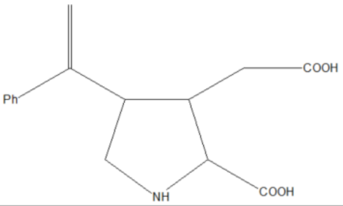

VAPMIK [Mascitti, 2011]	$C_{22}H_{25}ClO_7$, $C_5H_7NO_3$, 0.1(H_2O) 5-(4-chloro-3-(4-ethoxybenzyl)phenyl)-1-(hydroxymethyl)-6,8-dioxabicyclo[3.2.1]octane-2,3,4-triol *L*-pyroglutamic acid hydrate	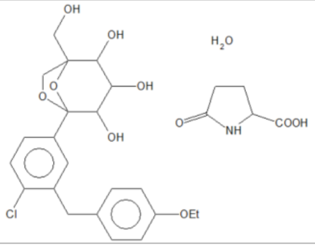
WOHRIV [Chandan, 2008]	$C_9H_{13}NO_3$ 5-(but-3-en-2yl)-2-pyrrolidone-5-carboxylic acid	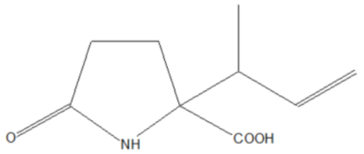
WOHROB [Chandan, 2008]	$C_8H_{11}NO_3$ 5-(prop-2-en-1-yl)-2-pyrrolidone-5-carboxylic acid	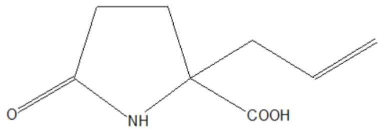
XOJXOL [Panda, 2015]	$C_5H_7NO_3$ 2-pyrrolidinone-5-carboxylic acid	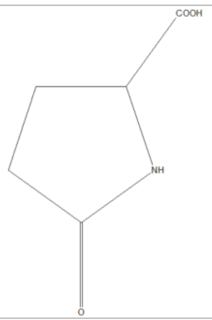
YOFSUK [Reingold, 2019]	$C^5H_9N_2O_3^+$, Cl^- 5-carboxy-2-oxopyrrolidin-3-aminium chloride	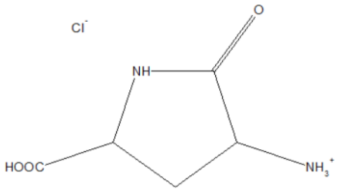
YOYYOD [Dick, 2019]	$C_{13}H_{15}NO_3$, H_2O (2*S*,3a*S*,8a*S*)-8a-hydroxy-3a-methyl-1,2,3,3a,8,8a-hexahydroindeno[2,1-b]pyrrole-2-carboxylic acid monohydrate	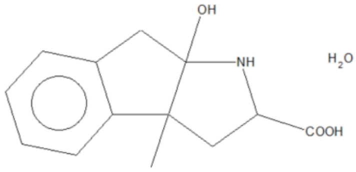

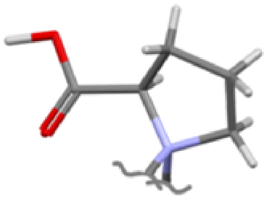

N$^+$- COOH

GEXTIM [Jones, 1988]	$C_7H_{14}NO_2^+$, Cl^- 2-carboxy-1,1-dimethyl-pyrrolidinium chloride	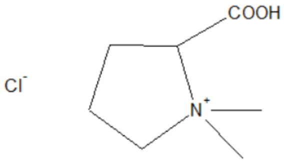
GOJMEX [Jones, 1988]	$C_7H_{14}NO_3^+$, Cl^- trans-*N*,*N*-dimethyl-4-hydroxy-*L*-proline chloride	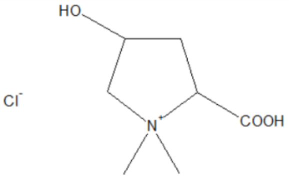

GOJMIB [Jones, 1988]	$C_7H_{14}NO_3^+$, Cl^-<br>*cis*-*N*,*N*-dimethyl-4-hydroxy-*D*-proline chloride	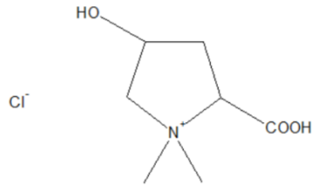

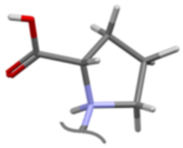

NH⁺ - COOH

DEKNIS [Milunovic, 2012]	$C_{15}H_{20}ClCuN_4O_3S^+$, Cl^-<br>(2-((2-carbamothioylhydrazinylidene)methyl)-6-((*R*)-(2-carboxypyrrolidinium-1-yl)methyl)-4-methylphenolato)-chloro-copper(ii) chloride<br>{chlorido-(3-methyl-*D*-prolinium-5-methyl-2-oxidobenzaldehyde thiosemicarbazone)-copper(ii) chloride}	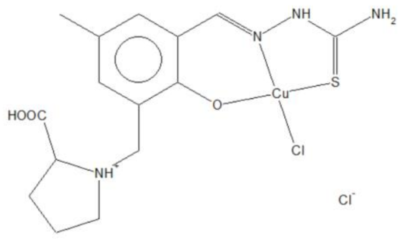
EQIHES [Mykhailiuk, 2011]	$C_{13}H_{17}FNO_2^+$, Cl^-<br>1-benzyl-2-carboxy-5-(fluoromethyl)pyrrolidinium chloride	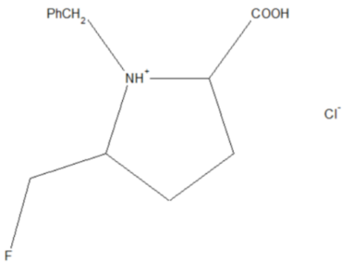
ERURUG [Dobrova, 2016]	$C_{19}H_{26}ClN_4O_3PdS^+$, Cl^-<br>(2-carboxy-1-(2-oxy-5-methyl-3-((2-(pyrrolidin-1-yl(sulfanylidene)methyl)hydrazono)methyl)benzyl)pyrrolidiniumato)-chloro-palladium(ii) chloride	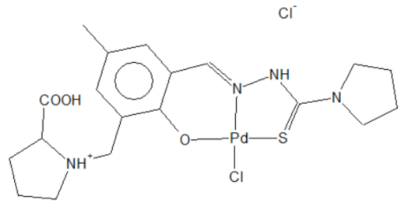
ERUSAN [Dobrova, 2016]	$C_{19}H_{26}ClN_4NiO_3S^+$, Cl^-<br>(2-carboxy-1-(2-oxy-5-methyl-3-((2-(pyrrolidin-1-yl(sulfanylidene)methyl)hydrazono)methyl)benzyl)pyrrolidiniumato)-chloro-nickel chloride	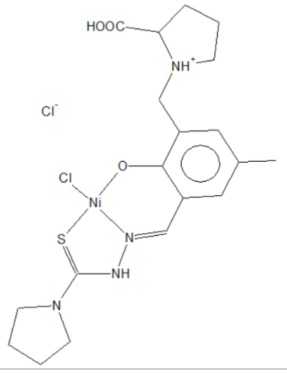
ESIJOH [Albrecht, 2016]	$C_{14}H_{14}F_6NO_2^+$, Cl^-<br>1-(3,5-bis(trifluoromethyl)benzyl)-2-carboxypyrrolidinium chloride	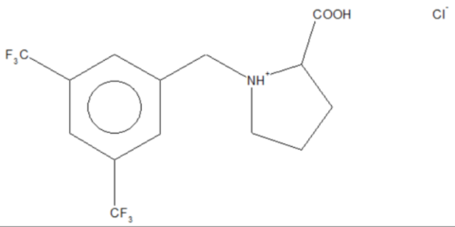
GEXTEI [Jones, 1988]	$C_6H_{12}NO_2^+$, Cl^-<br>1-methyl-*L*-prolinium chloride {hygric acid hydrochloride}	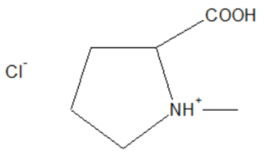

GOJMAT [Jones, 1988]	$C_6H_{12}NO_3^+$, Cl^-<br>*N*-methyl-4-hydroxy-*L*-proline hydrochloride	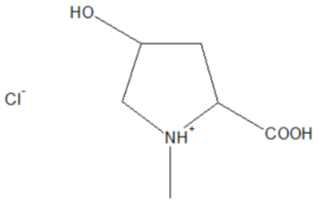
HIBZOH [Jones, 1995]	$C_6H_{12}NO_3^+$, Cl^-<br>trans-3-hydroxy-*N*-methyl-*L*-proline hydrochloride	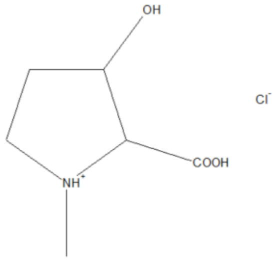
ROFQUZ [Dai, 2008b]	$C_{11}H_{16}N_2O_{22}^+$, 2(Cl^-), 0.5(H_2O)<br>(1*S*,2*S*)-2-carboxy-1-(3-pyridiniomethyl)pyrrolidin-1-ium dichloride hemihydrate<br>{(1*S*,2*S*)-1-((pyridin-3'-yl)methyl)pyrrolidine-2-carboxylic acid-1,1'-ium dichloride hemihydrate}	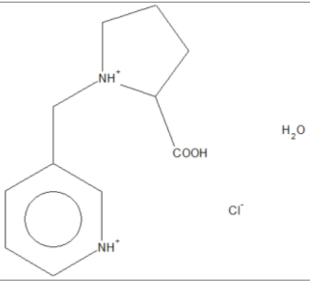
TECSIG [Novitchi, 2017]	$C_{14}H_{18}NO_4^+$, $C_{14}H_{17}NO_4$, Cl^-<br>2-carboxy-1-[(3-formyl-2-hydroxy-5-methylphenyl)methyl]pyrrolidin-1-ium chloride 1-[(3-formyl-2-hydroxy-5-methylphenyl)methyl]proline	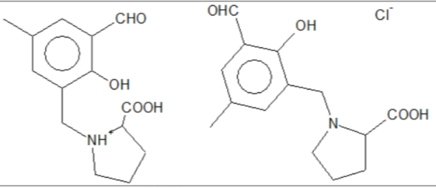
URIHAF [Zhang, 2011]	$C_{52}H_{60}N_4O_{12}$, 11(H_2O)<br>1-((11-((2-carboxylatopyrrolidin-1-yl)methyl)-17-((2-carboxypyrrolidinium-1-yl)methyl)-23-((2-carboxypyrrolidin-1-yl)methyl)-25,26,27,28-tetrahydroxypentacyclo[19.3.1.13,7.19,13.115,19]octacosa-1(25),3(28),4,6,9(27),10,12,15(26),16,18,21,23-dodecaen-5-yl)methyl)pyrrolidinium-2-carboxylate undecahydrate<br>{5,11,17,23-tetrakis(Prolylmethyl)-25,26,27,28-tetrahydroxycalix(4)arene undecahydrate}	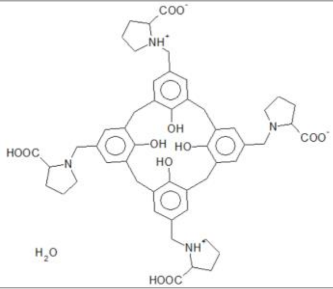
VANBOB [Sawka, 1989]	$C_6H_{12}NO_5P$<br>*N*-phosphonomethyl-*L*-proline	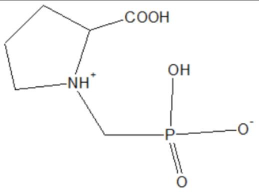

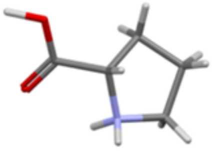

NH_2^+ - COOH

ADALIA [Swamy, 2001]	$C_5H_{10}NO_2^+$, $C_{23}H_{30}O_4P^-$<br>*S*-proline (46,63-di-t-butyl-44,65-dimethyl-4,6(1,2)-dibenzena-1,3,2-dioxaphosphacyclohexaphane 2,2-dioxide)	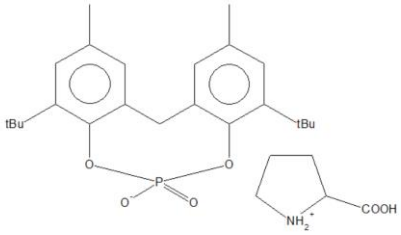

CADKOJ [Gharzaryan, 2011]	$C_5H_{10}NO_2^+$, $C_5H_9NO_2$, BF_4^-<br>*L*-prolinium *L*-proline tetrafluoroborate	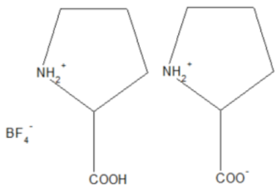
CADKUP* [Gharzaryan, 2011]	$C_5H_9NO_2$, Br^-, $C_5H_{10}NO_2^+$<br>*L*-prolinium *L*-proline bromide	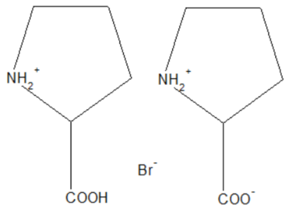
CADLIE [Gharzaryan, 2011]	$C_5H_9NO_2$, Cl^-, $C_5H_{10}NO_2^+$<br>*L*-proline *L*-prolinium chloride	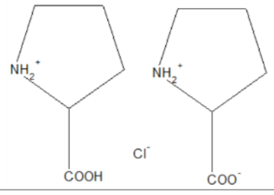
DLPROL [Mitsui, 1969]	$C_5H_{10}NO_2^+$, Cl^-<br>*D*,*L*-proline hydrochloride	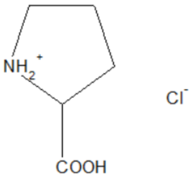
DODWEY [Andersen, 1986]	$C_5H_7NO_2S$<br>5-thioxoproline	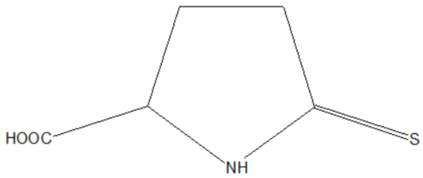
EXIBOC~~(01)~~ [Jin, 2003]	$C_5H_{10}NO_2^+$, $C_6H_2N_3O_7^-$<br>*L*-prolinium picrate	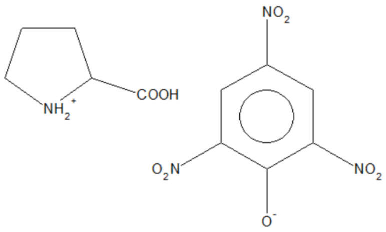
FEWQIK [Kondratov, 2018]	$C_7H_9F_2NO_4$<br>(2-carboxypyrrolidin-1-ium-3-yl)(difluoro)acetate	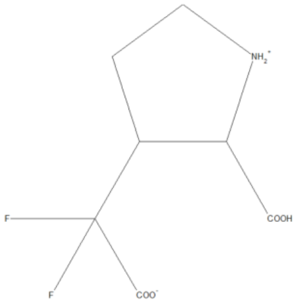
IDIMIT [Hobart, 2013]	$C_{12}H_{14}Cl_2NO_2Pd^-$, $C_{12}H_{16}NO_2^+$<br>(S)-a-benzylprolinium cis-[(*S*)-a-benzylprolinato]dichloridopalladium(II) {2-benzyl-2-carboxypyrrolidinium (2-benzylprolinato)(dichloro)palladate}	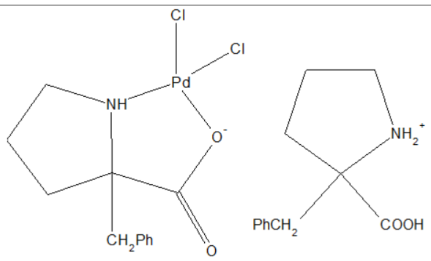
IDINAK* [Pandjarajan, 2002]	$C_5H_9NO_2$, $C_5H_{10}NO_2^+$, ClO_4^-<br>bis(*L*-proline) hydrogen perchlorate	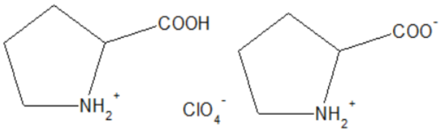

LONBUN*<br>[Ghazaryan, 2014]	$2(C_5H_{10}NO_2^+)$, $C_5H_9NO_2$, $F_6Si_2^-$, H_2O<br>bis(2-carboxypyrrolidinium) pyrrolidinium-2-carboxylate hexafluorosilicate monohydrate<br>{tris(proline) hexafluorosilicate monohydrate}	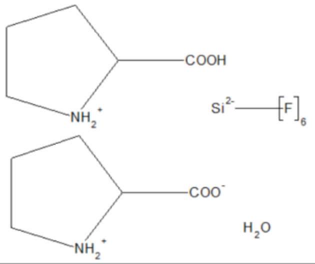
LUDFOF<br>[Pandiarajan, 2002]	$C_5H_9NO_2$, $C_5H_{10}NO_2^+$, NO_3^-<br>bis(*L*-proline) hydrogen nitrate	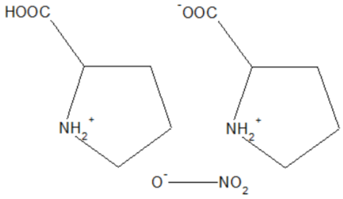
MODHEU<br>[Vasiuta, 2014]	$C_7H_{12}NO_3^+$, $C_7H_{11}NO_3$, Cl^-<br>4-(hydroxymethyl)-2-azabicyclo[2.1.1]hexane-1-carboxylic acid 4-(hydroxymethyl)-2-azabicyclo[2.1.1]hexane-1-carboxylate chloride	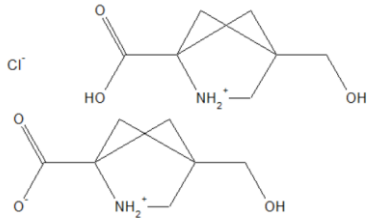
MPROLC<br>[Fuimoto, 1971]	$C_6H_{10}NO_2^+$, Cl^-, H_2O<br>*cis*-3,4-methylene-*L*-proline hydrochloride monohydrate	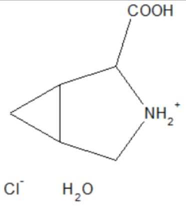
NEKCIP<br>[Wittland, 1997]	$C_8H_9NO_8$<br>(2*R*,*S*,3*R*,*S*,4*R*,*S*,5*R*,*S*)-pyrrolidine-2,3,4,5-tetracarboxylic acid	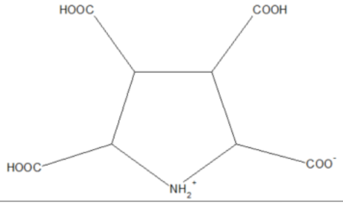
NUXYIO<br>[Hardeis, 1997]	$C_5H_9ClNO_3^+$, Cl^-<br>(2*S*,3*R*,4*R*)-2-carboxy-4-chloro-3-hydroxypyrrolidine hydrochloride<br>{all-trans-4-chloro-3-hydroxy-*L*-proline hydrochloride}	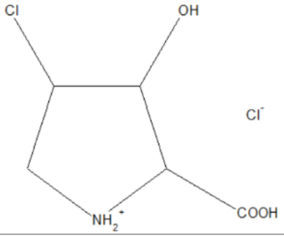
OLIZAL*<br>[Qu, 2011]	$2(C_5H_9NO_2)$, $2(C_5H_{10}NO_2^+)$, $2(C_{12}H_4N_4^-)$, $C_{12}H_4N_4$<br>bis(2-carboxypyrrolidinium) bis(7,7,8,8-tetracyanoquinodimethanide) bis(tetracyanoquinodimethane) pyrrolidinium-2-carboxylate	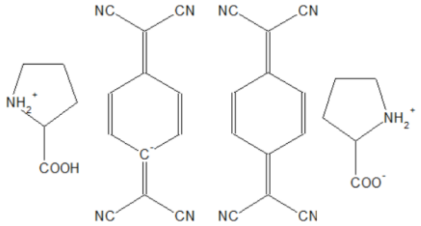
POMFEE<br>[Fleck, 2015]	$C_5H_{10}NO_2^+$, $H_2O_3P^-$<br>2-carboxypyrrolidinium hydrogen phosphonate<br>{*L*-proline hydrogen phosphite}	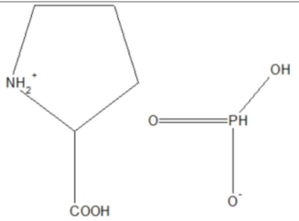
PROLNH*<br>[Swaminathan, 2011]	$C_5H_{10}NO_2^+$, $C_5H_9NO_2$, Cl^-<br>*D*,*L*-proline hemihydrochloride	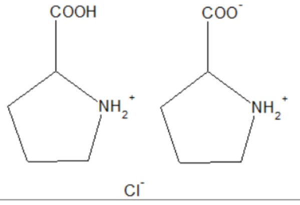

QIJYIR [Nandhini, 2001]	$C_5H_{10}NO_2^+$, $C_4H_5O_6^-$ *L*-prolinium hydrogen tartrate	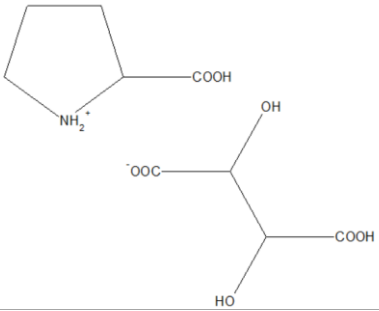
SIGJIE [Tolmachova, 2011]	$C_7H_9F_5NO_2^+$, H_2O, Cl^- 2-carboxy-3-(pentafluoroethyl)pyrrolidin-1-ium chloride monohydrate	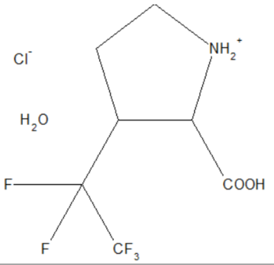
XUYVAO [Rajagopal, 2003]	$C_5H_{10}NO_2^+$, $C_2Cl_3O_2^-$ *L*-prolinium trichloroacetate	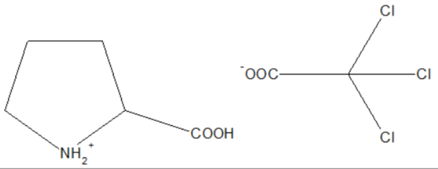

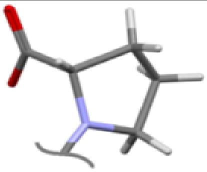

N - COO⁻

AFAFAP [Pilati, 2007]	$C_9H_{14}N_2O_3$ 6-ammonio-5-oxooctahydroindolizine-3-carboxylate	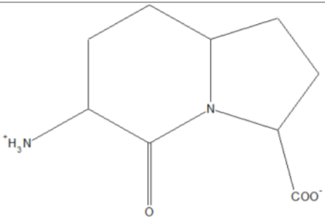
BAHPYC [Kanao, 1978]	$2(C_6H_8NO_4^-)$, Ba_2^+, H_2O barium bis (*N*-hydroxymethylpyrrolid-5-one-1-carboxylate) monohydrate	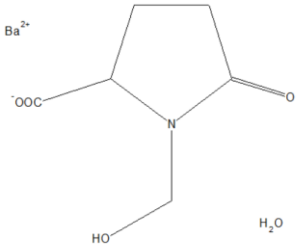
BIRYOQ [Martin, 1999]	Na^+, $C_{10}H_{14}NO_4^-$ sodium (5*S*,6*S*,9*S*)-5-hydroxy-5-methyl-1-azabicyclo[4.3.0]nonan-2-one-9-carboxylate	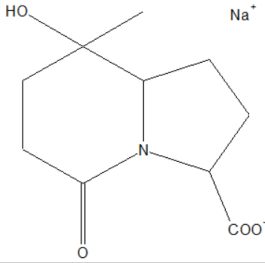
EMOCAL [Bhuyan, 2011]	$C_{12}H_{24}N^+$, $C_9H_{13}BrNO_3^-$ dicyclohexylammonium 1-(3-bromo-2-methylpropanoyl)pyrrolidine-2-carboxylate	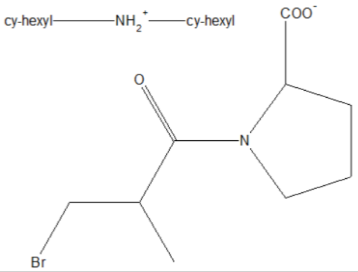

LEYQAK [Tlahuext, 2018]	$C_6H_7NO_2S_{22}^{-}$, $3(H_2O)$, $2(Rb^+)$ bis(rubidium) 1-carbodithioatopyrrolidine-2-carboxylate trihydrate {bis(rubidium) 1-carbodithioatoprolinate trihydrate}	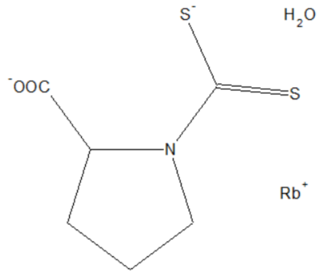
CAFKID [Watson, 1983]	$C_6H_{11}N_3O_3$, H_2O 1-amino(imino)methyl-2-hydroxypyrrolidine-2-carboxylic acid monohydrate	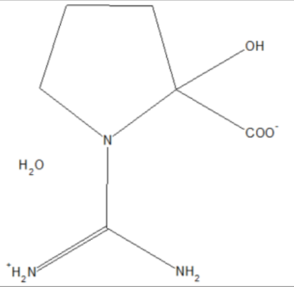
EDALEC	*See scheme 1, 2*	
GAKZEY [Slepokura, 2005]	$C_6H_{11}N_3O_3$, H_2O *N*-Amidino-4-hydroxy-*L*-proline monohydrate	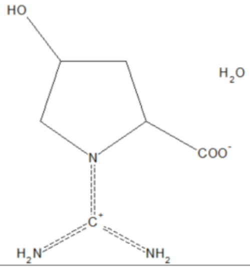
GEHMAI [Dittrich, 2006]	$C_{14}H_{18}N_2O_3$, H_2O *L*-phenylalanyl-*L*-proline monohydrate	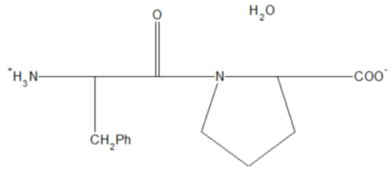
GERWUX(01)	*See scheme 1, 2*	
GERXAE	*See scheme 1, 2*	
GERXEI	*See scheme 1, 2*	
GLHPRA [Garbay, 1980]	$C_7H_{12}N_2O_4$ glycyl-*L*-4-hydroxyproline	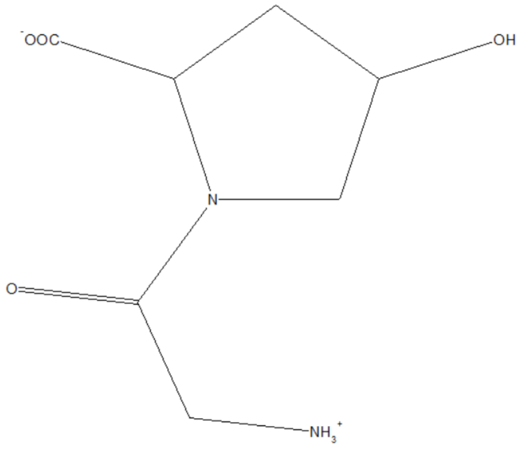
IDAFAX [Roy, 2016]	$C_{24}H_{22}ClN_2O_6^{-}$, $C_{10}H_{18}N^+$, $2(H_2O)$ adamantan-1-aminium 1-((1-(4-chlorobenzoyl)-5-methoxy-2-methyl-1H-indol-3-yl)acetyl)-4-hydroxypyrrolidine-2-carboxylate dihydrate	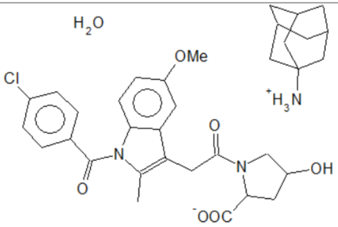
IVEGIA	*See scheme 1, 2*	

JADVAL [Panerselvan, 1989]	$C_{14}H_{18}N_2O_3$, H_2O *L*-phenylalanyl-*L*-proline monohydrate	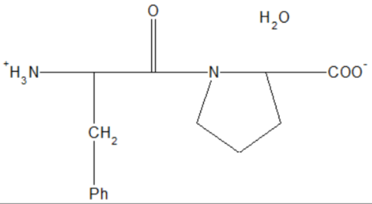
LEYQEO [Tlahuext, 2018]	$C_6H_7NO_2S_2^{2-}$, $2(H_2O)$, $2(Cs^+)$ bis(cesium) 1-carbodithioatopyrrolidine-2-carboxylate dihydrate {bis(cesium) 1-carbodithioatoprolinate dihydrate}	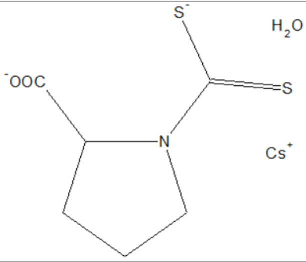
LPROHP20 [Garbay, 1980]	$C_{10}H_{16}N_2O_4$, H_2O *L*-prolyl-*L*-4-hydroxyproline monohydrate	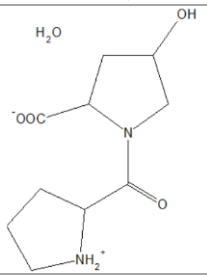
MAZMAE01 [Tlahuext, 2018]	$C_6H_7NO_2S_2^{2-}$, $3(H_2O)$, $2(K^+)$ bis(potassium) 1-carbodithioatopyrrolidine-2-carboxylate trihydrate {bis(potassium) 1-carbodithioato-*L*-prolinate trihydrate}	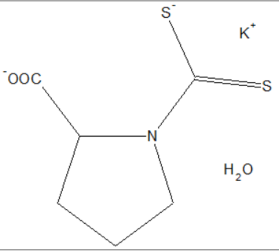
NOXWAA [Wartchow, 2014]	$C_{26}H_{38}N_2O_6S$, H_2O (2*S*,*R*,2'*S*,*R*,3*S*,*R*)-1,2,4,5,6,7,8,9-octahydro-1'-[(4-methoxy-2,3,6-trimethyl phenyl)-sulfonyl]-1-methyl-2-(2-oxopropyl)-spiro[3H-indol-3,3'-pyrrolidin]-2'-carboxylic acid monohydrate	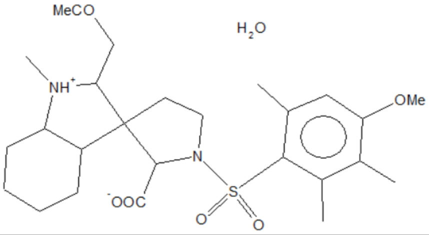
SEHGES [Moggach, 2006]	$C_7H_{12}N_2O_3$, $0.5(H_2O)$ glycyl-*L*-proline hemihydrate	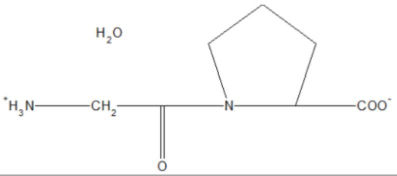
TUHMOY	*See scheme 1, 2*	
TUHMUE	*See scheme 1, 2*	
UGUYUQ [Macias, 2002]	$C_4H_{20}CuN_2O_4^{2+}$, $C_{12}H_{14}CuN_2O_4S_4^{2-}$ (ethylenediamine)-(ethanol)-triaqua-copper(ii) bis(proline-dithiocarbamato)-copper(ii)	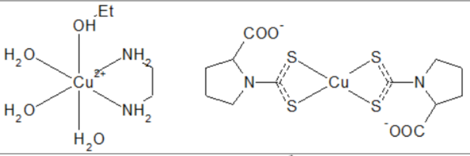
UROLAQ [Bakonyi, 2013]	$C_{11}H_{16}NO_4^-$, $C_8H_{12}NO^+$ 2-hydroxy-1-phenylethanaminium 3-(t-butoxycarbonyl)-3-azabicyclo[3.1.0]hexane-2-carboxylate {(*R*)-phenylglycinolium (+)-trans-(1*R*,2*R*,5*S*)-3-(t-butoxy-carbonyl)-3-azabicyclo[3.1.0]hexane-2-carboxylate}	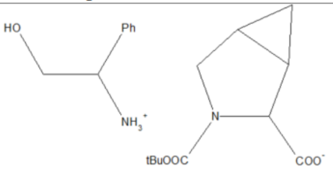
UZOVAH(01,03)	*See scheme 1, 2*	
UZOVIP	*See scheme 1, 2*	
VEDBOW [Bialonska, 2006]	$C_{23}H_{27}N_2O_4^+$, $C_{10}H_{16}NO_4^-$, C_2H_6O, H_2O brucinium *N*-(t-butoxycarbonyl)-*L*-prolinate ethanol solvate monohydrate	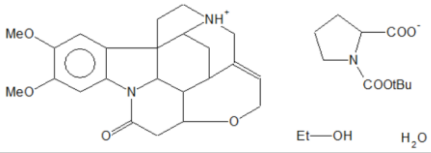

VIRWUO(**01**) [Bowler, 1991]	$C_{18}H_{21}N_2O_7S^-$, $C_{12}H_{24}N^+$ dicyclohexylammonium *t*-butoxycarbonyl-4-(1-methyl-3,3-dioxo-3l6-thiaisoindolinyl)-3-oxoproline	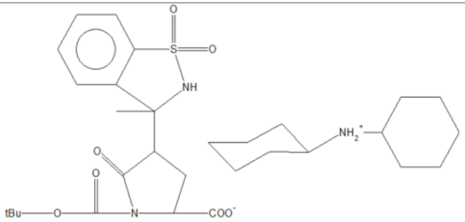
WAPVUF [Eustache, 2005]	$C_{12}H_{18}NO_4^-$, $C_{12}H_{24}N^+$ dicyclohexylammonium (2*S*,5*R*)-*N*-*t*-butoxycarbonyl-5-vinylpyrrolidine-2-carboxylate	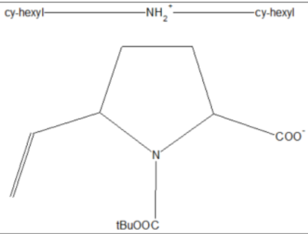
WIPXUO [Nikiforovich, 1996]	$C_{30}H_{37}N_5O_7S_2$, $2(C_2H_6O)$ tyrosyl-cyclo(*D*-b,b-dimethylcysteinyl-glycyl-phenylalanyl-D-trans-3-mercaptoproline) ethanol solvate {cyclic peptide (4 residues): CYS*!-GLY-PHE-PRO*, acyclic peptide (2 residues): TYR-CYS*!}	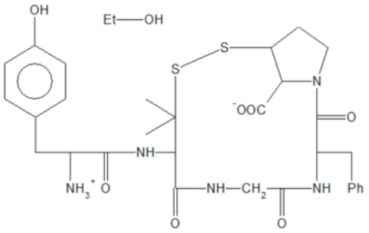

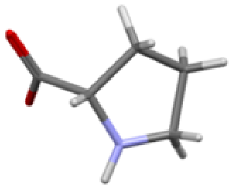

NH - COO$^-$

CPISIN [Eberle, 1980]	$C_{22}H_{20}Cl_2NO^+$, $C_5H_6NO_3^-$ (a*S*,1*S*)-(+)-a,1-bis(4-chlorophenyl)-isoindoline-1-ethanol (*S*)-(-)-5-carboxylato-2-pyrrolidone	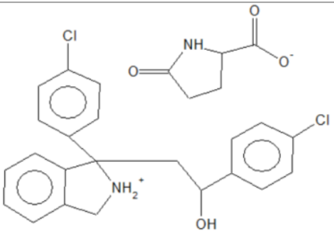
FIRGUL [Babor, 2019]	$C_{10}H_{16}NO^+$, $C_5H_6NO_3^-$, H_2O 1-hydroxy-*N*-methyl-1-phenylpropan-2-aminium 5-oxopyrrolidine-2-carboxylate monohydrate	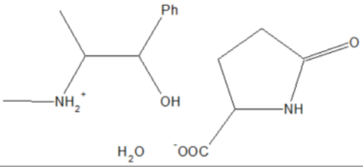
HOGZIN [Kim, 2008]	$C_5H_6NO_5^-$, H_4N^+, H_2O ammonium (3'*S*,4'*S*,5'*S*)-(3',4'-dihydroxypyrrolidin-2'-one-5'-yl)formate monohydrate	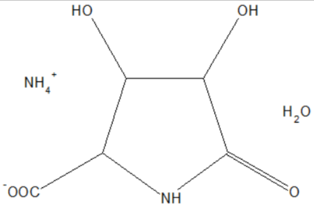
KIBKEL [Minacheva, 1989]	$C_{12}H_{24}N_6O_6PtS_2$, $4(H_2O)$ bis(Hydroxyproline-*N*)-bis(thiourea-*S*)-platinum(ii) tetrahydrate	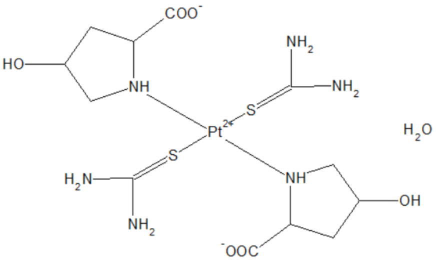

KUTCOS [Kaczmarek, 2010]	$C_5H_8N_2O_3$<br>(2*S*,4*R*)-4-ammonio-5-oxopyrrolidine-2-carboxylate	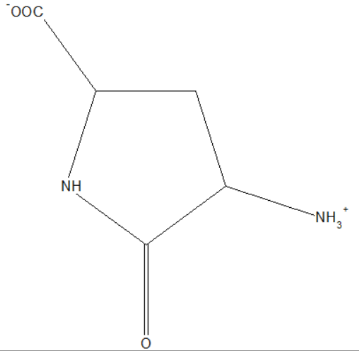
LOQQAJ [Sakai, 1999]	$C_9H_{16}N_2O$<br>dysibetaine {(2*R*8,4*R*8)-2-(trimethylammonium)methyl-4-hydroxy-5-oxoprolinate}	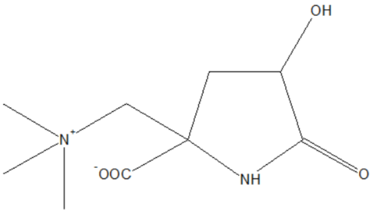
PAMPOL [Kodama, 2014]	$C_{16}H_{28}NO^+$, $C_5H_6NO_3^-$<br>1-(3,5-di-*t*-butyl-2-hydroxyphenyl)ethanaminium 5-oxopyrrolidine-2-carboxylate {1-(3,5-di-t-butyl-2-hydroxyphenyl)ethylammonium *L*-pyroglutamate}	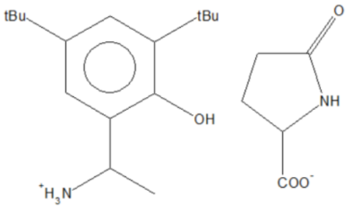
XAGZUB [Zhang, 2004]	$(C_{120}H_{264}ClCu_{30}N_{24}Nd_6O10323+)n$, 11n($HO^-$), 12n($ClO_4^-$), 6n($H_2O$)<br>catena-[triacontakis(m3-Hydroxo)-tetracosakis(m-L-prolinato)-(m-perchlorate)-henicosakis(aqua)-triaconta-copper(ii)-hexa-neodymium(iii) undecahydroxide dodecaperchlorate hexahydrate]	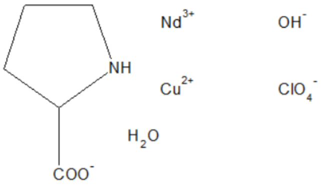
VIRWUQ [Wen, 2013]	$(C_{14}H_{22}N_4O_2Zn_2^+)_n$, n($C_5H_6NO_3^-$), n($ClO_4^-$), 3n($H_2O$)<br>catena-[(m2-*N*,*N*'-bis(pyridin-4-ylmethyl)ethane-1,2-diamine)-diaqua-zinc (*R*)-5-oxopyrrolidine-2-carboxylate perchlorate trihydrate]	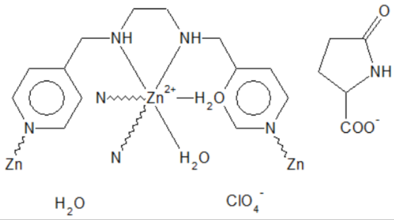
VIWFOW [Kumberger, 1991]	$(C_5H_{10}NNaO_5)n$, n(H_2O)<br>catena-(bis(m2-Aqua-O,O)-(m2-L-pyroglutamate-O,O)-sodium monohydrate) {catena-(bis(m2-aqua)-(m2-2-pyrrolidone-5-carboxylate-O,O)-sodium monohydrate)}	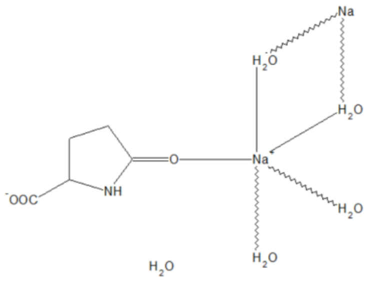
WAWSIW [Khokhar, 1993]	$C_{16}H_{30}N_4O_4Pt$, CH_4O, 2(H_2O)<br>bis(*L*(-)prolinato-*N*)-(trans-*R*,*R*-1,2-diaminocyclohexane)-platinum(ii) methanol solvate dihydrate	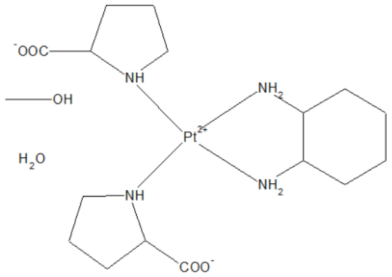

WAWSOC [Khokhar, 1993]	$C_{16}H_{30}N_4O_4Pt$, 6(H_2O) bis(*L*(-)prolinato-*N*)-(*cis*-1,2-diaminocyclohexane)-platinum(ii) hexahydrate	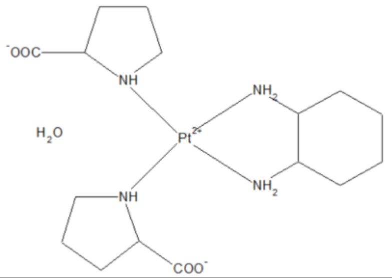

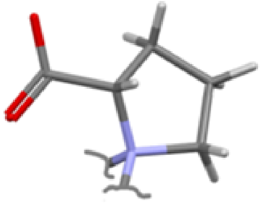

N^{+}- COO^{-}

BIKMUE [Kirbimizeknes, 2004]	$C_{16}H_{19}NO_5$, 2(H_2O) (2*S*,4*R*)-2-carboxy-4-((E)-3-(4-hydroxyphenyl)prop-2-enoyloxy)-1,1-dimethylpyrrolidinium dihydrate {(2*S*,4*R*)-2-carboxy-4-((E)-*p*-coumaroyloxy)-1,1-dimethylpyrrolidinium dihydrate}	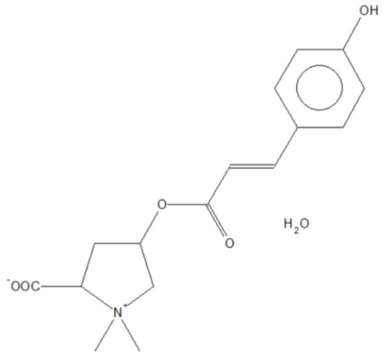
FUPTOB [Turdybekov, 2013]	$C_7H_{13}NO_2$, H_2O 1,1-dimethylpyrrolidinium-2-carboxylate monohydrate	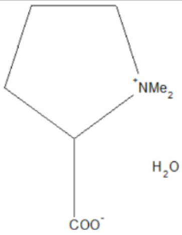
JOKLOM [zhang, 2014]	2($C_{19}H_{20}N_2O_2$), CH_4O, H_2O 4,16-cyclocoryn-19-en-4-ium-17-oate methanol solvate monohydrate	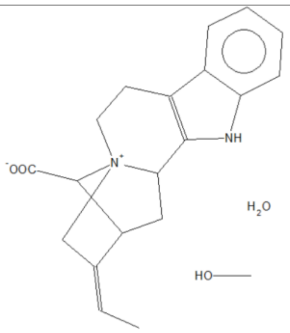
KILKAS [Jenkinson, 2007]	$C_7H_{13}NO_3$, H_2O (2*R*,3*S*)-3-hydroxy-*N*,*N*-dimethylproline monohydrate	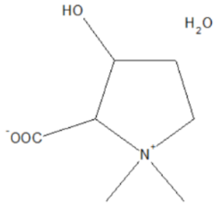
REGKUK [Razzakov, 2005]	$C_7H_{13}NO_3$, CH_4N_2O trans-*N*,*N*-dimethyl-4-hydroxy-*L*-proline urea betonicin urea}	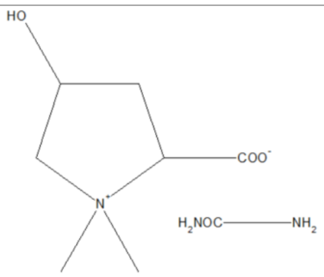

XUHZOR<br>[Wang, 2015]	$C_6H_{11}N_3O_2$, H_2O<br>1-(diaminomethylene)pyrrolidinium-2-carboxylate monohydrate {(*S*)-1-carbamimidoylpyrrolidine-2-carboxylic acid monohydrate}	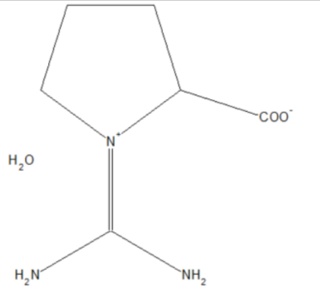
ZATNIR<br>[O`neil, 1995]	$C_{12}H_{15}NO_3$<br>1-benzyl-(1*R*)-oxide-(2*S*)-proline	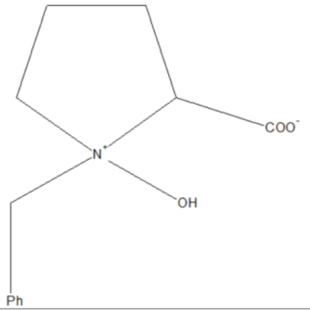

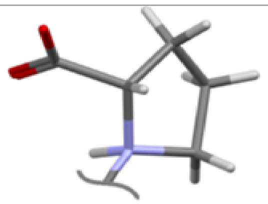

NH⁺- COO⁻

CEFVAM<br>[Edgeley, 2012]	$C_9H_{15}NO_7$<br>1-(2-carboxyethyl)-3,4-dihydroxy-5-(hydroxymethyl)pyrrolidinium-2-carboxylate {*N*-(2-carboxyethyl)-2,5-dideoxy-2,5-imino-*D*-mannonic acid; (3*R*,4*R*,5*R*)-1-(2-carboxyethyl)-3,4-dihydroxy-5-hydroxymethyl-*L*-proline}	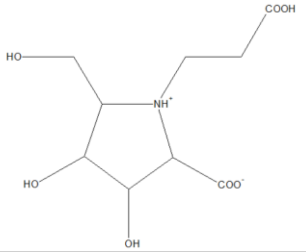
ERUSER<br>[Dobrova, 2016]	$C_{19}H_{26}N_4O_3S$, 1.25(CH_4O)<br>1-(2-hydroxy-5-methyl-3-(((pyrrolidin-1-ylcarbonothioyl)hydrazono)methyl)benzyl)pyrrolidinium-2-carboxylate methanol solvate	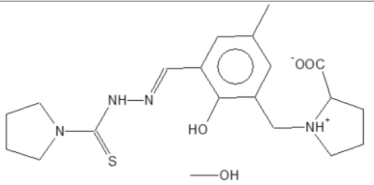
FUMJIH<br>[Renzi, 2010]	$C_{17}H_{27}NO_3$, $C_2H_6O_2$<br>1-(3-butyl-6-oxobicyclo[2.2.2]oct-2-yl)pyrrolidinium-2-carboxylate ethane-1,2-diol solvate	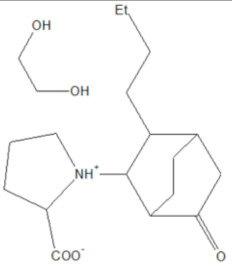
GINPOK<br>[Liautard, 2013]	$C_{10}H_{17}NO_3$, H_2O<br>1-hydroxy-2-methyloctahydroindolizinium-3-carboxylate monohydrate	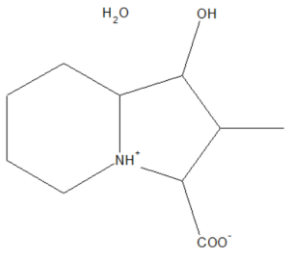

GINPUQ [Liautard, 2013]	$C_{11}H_{19}NO_3$, H_2O<br>1-hydroxy-2,8-dimethyloctahydroindolizinium-3-carboxylate monohydrate	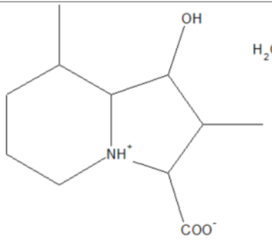
HESHOD [Peifer, 2003]	$C_{17}H_{33}NO_4$, H_2O<br>(4*R*)-4-hydroxy-1-((2*S*)-2-hydroxydodecyl)-*L*-proline monohydrate	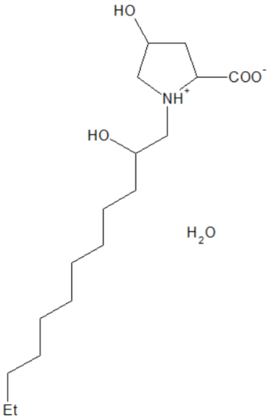
HIDHIM [Tarnavski, 2007]	$C_{11}H_{19}NO_7$, CH_4O<br>*N*-(1-deoxy-b-*D*-fructopyranos-1-yl)-*L*-proline methanol solvate	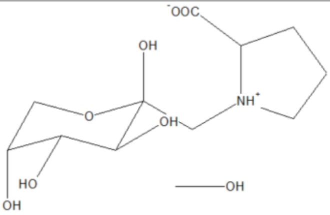
HIDHOS [Tarnavski, 2007]	$C_{11}H_{19}NO_7$, $2(H_2O)$<br>*N*-(1-deoxy-b-*D*-fructopyranos-1-yl)-*L*-proline dihydrate	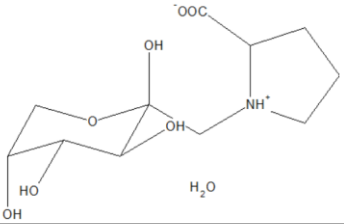
JIMVAD [Mossine, 2007]	$C_{11}H_{19}NO_7$, H_2O<br>*N*-(1-deoxy-b-*D*-fructopyranose-1-yl)-*L*-proline monohydrate	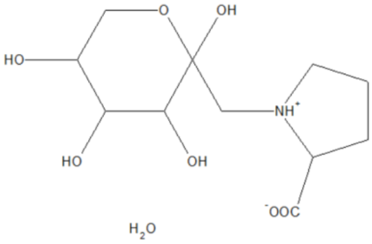
JIMVEH [Mossine, 2007]	$C_{11}H_{19}NO_6$<br>*N*-(1,6-dideoxy-a-*L*-fructofuranose-1-yl)-*L*-proline	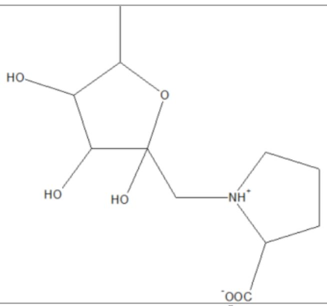
LAKGUA [Dong, 2005]	$C_{16}H_{17}NO_3$<br>*N*-(2-hydroxy-1-naphthylmethyl)-(*S*)-proline {(2*S*)-1-(2-hydroxy-1-naphthylmethyl)pyrrolidinio-2-carboxylate}	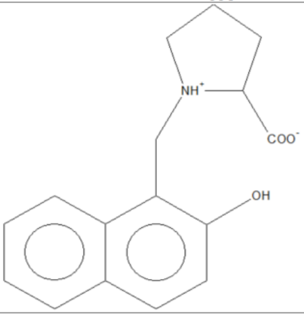

QIJMAZ [Rajalakshmi, 2013]	$C_{12}H_{15}NO_2$<br>1-benzylpyrrolidinium-2-carboxylate {*N*-benzyl-*L*-proline}	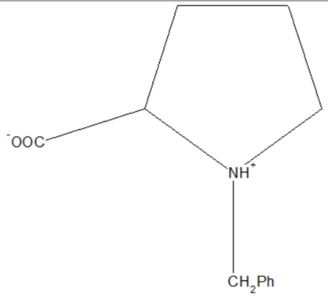
RASQUA [Liu, 2017]	$C_{32}H_{31}N_3O_5$, 0.5(C_2H_3N), 1.5(H_2O)<br>(2*S*)-1-[(1*S*)-1-[8-(9H-fluoren-9-ylmethoxycarbonylamino)-4-methoxy-2-quinolyl]ethyl]pyrrolidinium-2-carboxylate acetonitrile solvate sesquihydrate	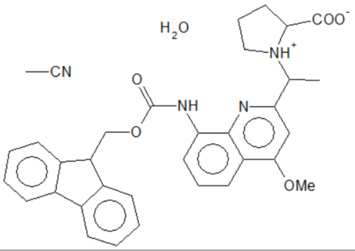
ROKXAQ [Toscano, 1997]	$C_6H_{11}NO_2$, H_2O<br>*L*-1-methylproline monohydrate {hygric acid}	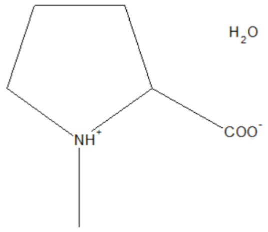
RUSLUO [Barker, 2015]	$C_{52}H_{60}N_4O_{12}$, C_4H_8O<br>(25,26,27,28-tetrahydroxycalix(4)arene-5,11,17,23-tetramethylene)tetrakis(proline) tetrahydrofuran solvate	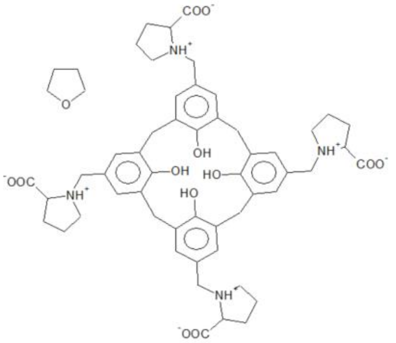
UGUHOT [Yapp, 2002]	$C_6H_{11}NO_3$<br>4-hydroxy-*N*-methylproline	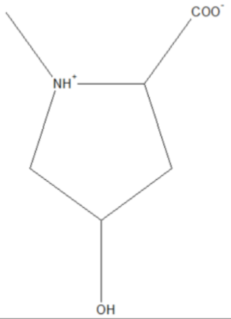
URIHAF [Zhang, 2011]	$C_{52}H_{60}N_4O_{12}$, 11(H_2O)<br>1-((11-((2-Carboxylatopyrrolidin-1-yl)methyl)-17-((2-carboxypyrrolidinium-1-yl)methyl)-23-((2-carboxypyrrolidin-1-yl)methyl)-25,26,27,28-tetrahydroxypentacyclo[19.3.1.13,7.19,13.115,19]octacosa-1(25),3(28),4,6,9(27),10,12,15(26),16,18,21,23-dodecaen-5-yl)methyl)pyrrolidinium-2-carboxylate undecahydrate {5,11,17,23-tetrakis(prolylmethyl)-25,26,27,28-tetrahydroxycalix(4)arene undecahydrate}	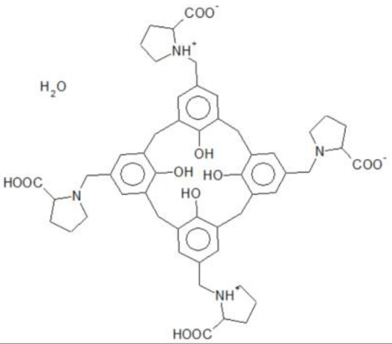

VOTKIY [Pereira, 1991]	$C_8H_{13}NO_5$ (1*R*,2*R*,3*S*,7*S*,7a*R*)-3-carboxy-1,2,7-trihydroxypyrrolizidine {7a-epialexaflorine}	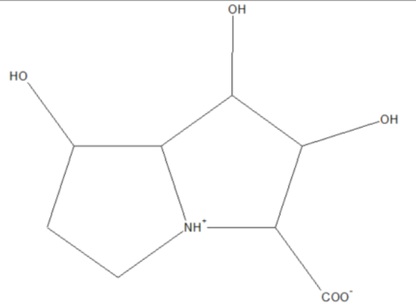
WITZUX [Salonen, 2019]	$C_{20}H_{31}NO_3$, $C_{14}H_{22}O$ 1-[(3,5-di-*t*-butyl-2-hydroxyphenyl)methyl]pyrrolidin-1-ium-2-carboxylate 2,4-di-*t*-butylphenol	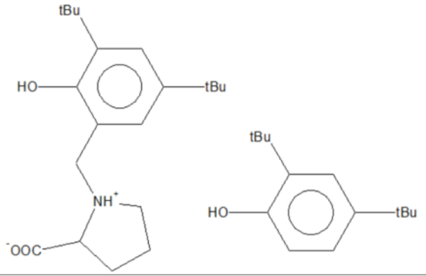
XUFFEM [Dank, 2020]	$C_{16}H_{21}NO_6$, $CHCl_3$, $0.5(H_2O)$ 1-[(3-acetyl-2-hydroxy-4,6-dimethoxyphenyl)methyl]pyrrolidin-1-ium-2-carboxylate chloroform solvate hemihydrate {(-)-monophyllidin chloroform solvate hemihydrate}	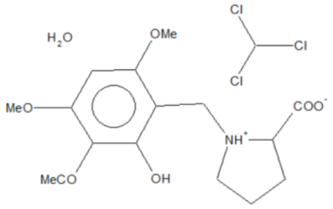
XUFFIQ [Dank, 2020]	$C_{16}H_{21}NO_6$, $2.5(H_2O)$ 1-[(3-acetyl-2-hydroxy-4,6-dimethoxyphenyl)methyl]pyrrolidin-1-ium-2-carboxylate hydrate {(-)-monophyllidin hydrate}	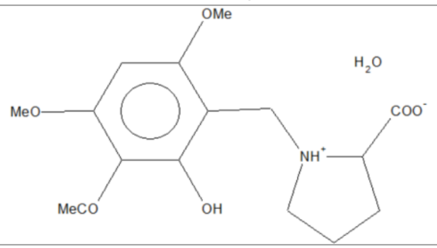
XUFFOW [Dank, 2020]	$C_{16}H_{21}NO_6$, $3(H_2O)$ 1-[(3-acetyl-2-hydroxy-4,6-dimethoxyphenyl)methyl]pyrrolidin-1-ium-2-carboxylate {(-)-monophyllidin trihydrate}	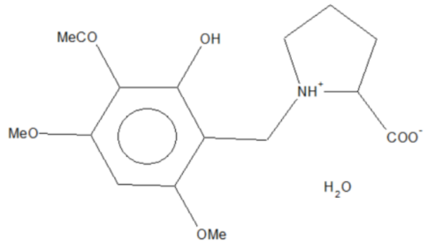
XUFGAJ [Dank, 2020]	$C_{16}H_{21}NO_6$, $0.5(H_2O)$ 1-[(3-acetyl-2-hydroxy-4,6-dimethoxyphenyl)methyl]pyrrolidin-1-ium-2-carboxylate unknown solvate hemihydrate {(-)-monophyllidin unknown solvate hemihydrate}	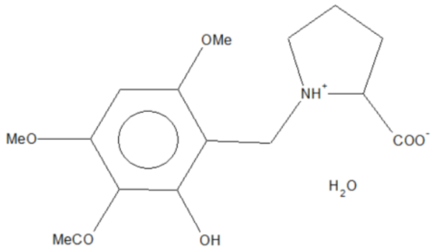
XUFGEN [Dank, 2020]	$C_{16}H_{21}NO_6$, $0.5(C_2H_3N)$, H_2O 1-[(3-acetyl-2-hydroxy-4,6-dimethoxyphenyl)methyl]pyrrolidin-1-ium-2-carboxylate acetonitrile solvate monohydrate {(-)-monophyllidin acetonitrile solvate monohydrate}	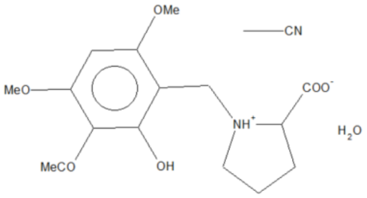
XUFGIR [Dank, 2020]	$C_{16}H_{21}NO_6$, C_2H_3N 1-[(3-acetyl-2-hydroxy-4,6-dimethoxyphenyl)methyl]pyrrolidin-1-ium-2-carboxylate acetonitrile solvate {(-)-monophyllidin acetonitrile solvate}	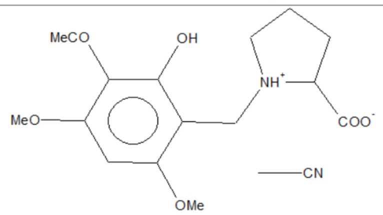

YUCSOE [Mattson, 1994]	$C_{17}H_{24}N_2O_6$ (2*S*,5*R*)-*N*-benzyl-2-carboxy-5-(((2-hydroxy-1,1-bis(hydroxymethyl)ethyl)amino)carbonyl)pyrrolidine	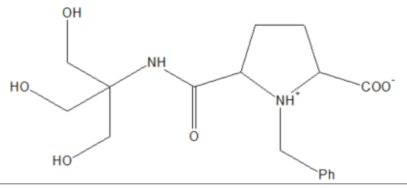
ZEQSUM [Milunovic, 2017]	$C_{15}H_{19}Cl_2FeN_4O_3S$ [1-{[3-{[2-(carbamothioyl)hydrazinylidene]methyl}-2-oxido-5-methylphenyl]methyl}-2-carboxypyrrolidin-1-iumato]-(dichloro)-iron(iii)	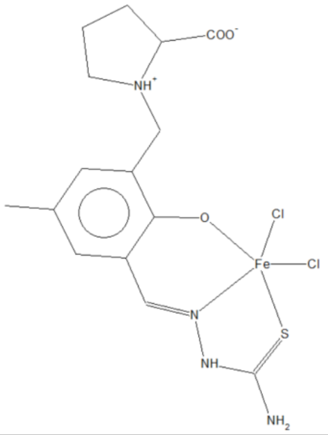
ZEQTAT [Milunovic, 2017]	$C_{19}H_{25}Cl_2FeN_4O_3S$, 0.125($C_4H_{10}O$), 0.2($CH_4O$), 0.063($H_2O$) [2-carboxy-1-{[2-oxido-5-methyl-3-({2-[pyrrolidine-1-(sulfanylidene)methyl]hydrazinylidene}methyl)phenyl]methyl}pyrrolidin-1-iumato]-(dichloro)-iron(iii) diethyl ether methanol solvate hydrate	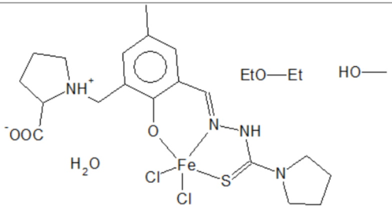
ZEQTEX [Milunovic, 2017]	$C_{22}H_{27}Cl_2FeN_4O_4S$, 0.5($CH_4O$) [2-carboxy-1-{[2-oxido-5-methyl-3-{[2-(phenylcarbamothioyl)hydrazinylidene]methyl}phenyl]methyl}pyrrolidin-1-iumato]-dichloro-methanol-iron(iii) methanol solvate	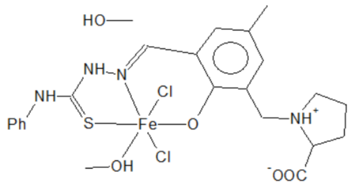
ZEQTIB [Milunovic, 2017]	$C_{28}H_{32}Cl_2FeN_5O_4S$, $C_4H_{10}O$ (2-[(2-carboxylatepyrrolidinium-1-yl)methyl]-4-methyl-6-{[2-(naphthalen-1-ylcarbamothioyl)hydrazinylidene]methyl}phenolato)-dichloro-(*N*,*N*-dimethylformamide)-iron(iii) diethyl ether solvate	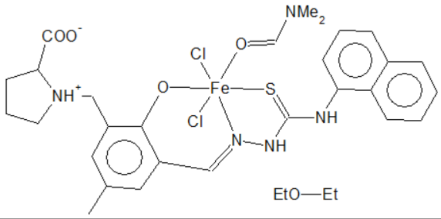

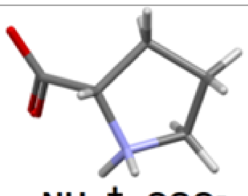

NH_2^+- COO-

AHEMOR [Myasnyanko, 2015]	$C_8H_{13}NO_3$ 4-acetyl-4-methylpyrrolidinium-2-carboxylate {4-acetyl-4-methylproline}	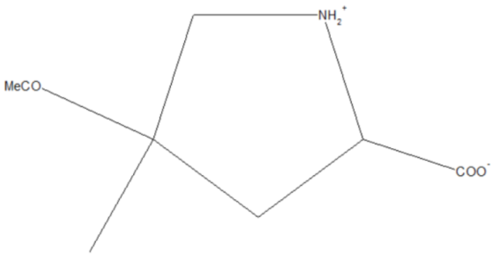

AHLPRO<br>[Shamala, 1976]	$C_5H_9NO_3$, $2(H_2O)$<br>allo-4-hydroxy-*L*-proline dihydrate	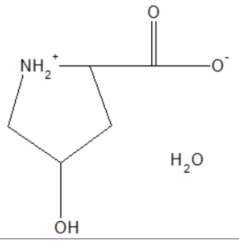
ALKINA**01**)<br>[Cruishank, 1959]	$C_{10}H_{15}NO_4$<br>allokainic acid	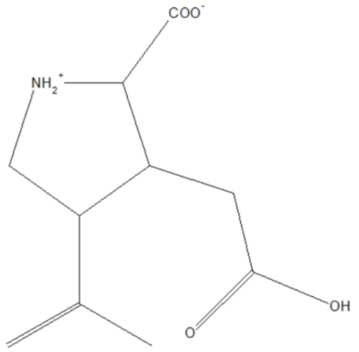
BEJNAI(01)<br>[Almansa, 2017]	$C_{17}H_{14}F_3N_3O_2S$, $2(C_5H_9NO_2)$<br>bis(pyrrolidinium-2-carboxylate) 4-(5-(4-methylphenyl)-3-(trifluoromethyl)-1H-pyrazol-1-yl)benzenesulfonamide {celecoxib *bis*(*L*-proline)}	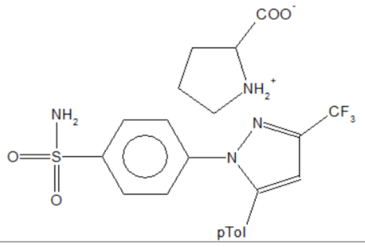
BEXGUI<br>[Tilborg, 2013]	$C_{14}H_{14}O_3$, $C_5H_9NO_2$, H_2O<br>pyrrolidinium-2-carboxylate 2-(6-methoxy-2-naphthyl)propanoic acid monohydrate {*L*-prolinium (*R*,*S*)-naproxen monohydrate}	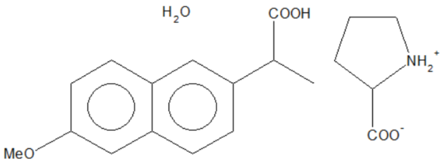
BEYTUW<br>[Tilborg, 2013]	$C_{14}H_{14}O_3$, $C_5H_9NO_2$, H_2O<br>pyrrolidinium-2-carboxylate 2-(6-methoxynaphthalen-2-yl)propanoic acid monohydrate {*D*,*L*-prolinium *R*,*S*-naproxen monohydrate}	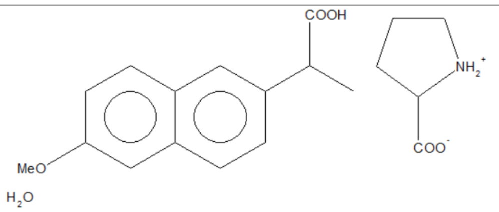
BOCJEL [Foletti, 2019]	$C_{16}H_{17}NO_2$<br>4-[(naphthalen-2-yl)methyl]pyrrolidin-1-ium-2-carboxylate	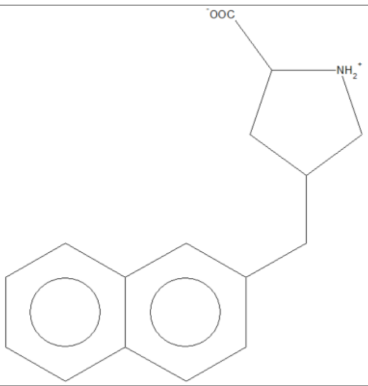
BOYTIS<br>[Oszbach, 1983]	$C_{24}H_{33}NO$<br>2-methyl-9-phenyl-4a-pyrrolidin-1-yl-1,2,3,4,4a,5,6,7,8,9a-decahydroxanthene	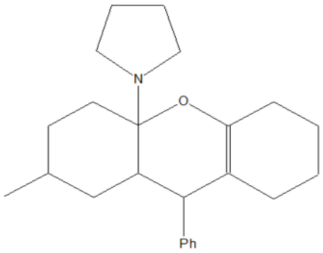

CADKOJ [Gharzaryan, 2011]	$C_5H_{10}NO_2^+$, $C_5H_9NO_2$, BF_4^-<br>*L*-prolinium *L*-proline tetrafluoroborate	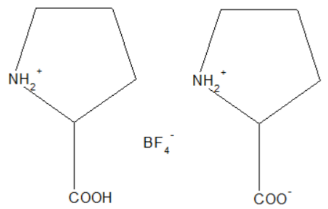
CADLIE [Gharzaryan, 2011]	$C_5H_9NO_2$, Cl^-, $C_5H_{10}NO_2^+$<br>*L*-proline *L*-prolinium chloride	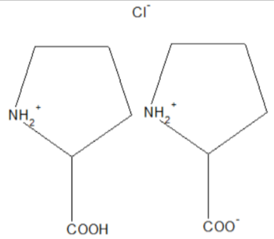
CENZUT [Fujisawa, 2018]	$C_{36}H_{40}O_8$, $C_5H_9NO_2$, $2(CH_4O)$, $3.5(H_2O)$<br>pyrrolidin-1-ium-2-carboxylate 2,8,14,20-tetraethylpentacyclo[19.3.1.13,7.19,13.115,19]octacosa-1(25),3(28),4,6,9(27),10,12,15(26),16,18,21,23-dodecaene-4,6,10,12,16,18,22,24-octol methanol solvate hydrate {C-ethyl-calix(4)resorcinarene *D*,*L*-proline clathrate methanol solvate hydrate}	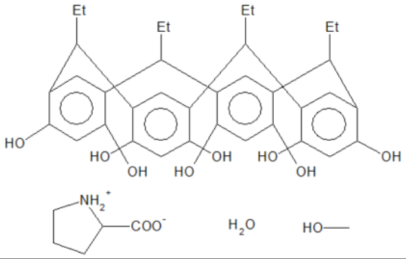
CEPBAD [Fujisawa, 2018]	$C_{36}H_{40}O_{12}$, $C_5H_9NO_2$, C_2H_6O, $5(H_2O)$<br>pyrrolidin-1-ium-2-carboxylate 2,8,14,20-tetraethylpentacyclo[19.3.1.13,7.19,13.115,19]octacosa-1(25),3(28),4,6,9(27),10,12,15(26),16,18,21,23-dodecaene-4,5,6,10,11,12,16,17,18,22,23,24-dodecol ethanol solvate pentahydrate {*C*-ethyl-calix(4)pyrogallolarene *L*-proline clathrate ethanol solvate decahydrate; 2,8,14,20-tetraethyl-4,5,6,10,11,12,16,17,18,22,23,24-dodecahydroxycalix(4)arene proline ethanol solvate pentahydrate}	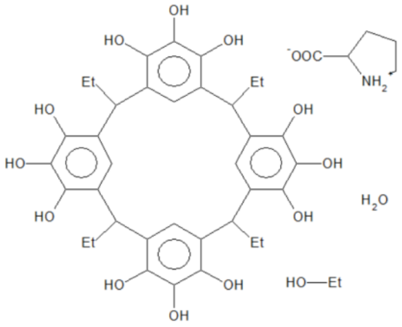
CIDBOH [Athimoolam, 2007]	$C_7H_7NO_2$, $2(C_5H_9NO_2)$, H_2O<br>4-aminobenzoic acid bis(*L*-proline) monohydrate	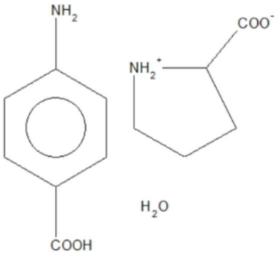
CIPVUU [Bien, 2009]	$C_{21}H_{25}ClO_6$, $2(C_5H_9NO_2)$<br>bis(2-carboxypyrrolidinium) 2-(4-chloro-3-(4-ethoxybenzyl)phenyl)-6-(hydroxymethyl)tetrahydro-2H-pyran-3,4,5-triol {dapagliflozin bis(proline)}	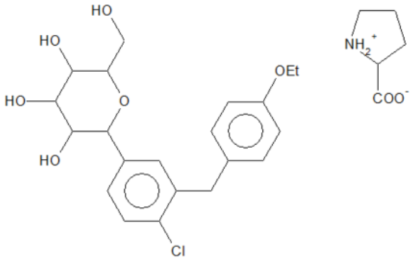
CIPZIM [Bien, 2009]	$C_{21}H_{25}ClO_6$, $C_5H_9NO_2$<br>2-(4-chloro-3-(4-ethoxybenzyl)phenyl)-6-(hydroxymethyl)tetrahydro-2H-pyran-3,4,5-triol L-proline {dapagliflozin *L*-proline}	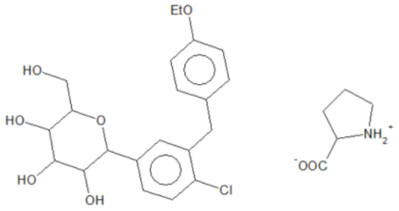
CIRFEQ [Bien, 2009]	$C_{21}H_{25}ClO_6$, $C_5H_9NO_2$, $0.5(H_2O)$<br>2-(4-chloro-3-(4-ethoxybenzyl)phenyl)-6-(hydroxymethyl)tetrahydro-2H-pyran-3,4,5-triol L-proline hemihydrate {dapagliflozin *L*-proline hemihydrate}	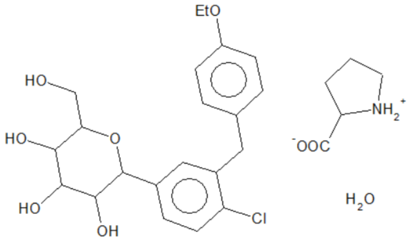

COGKOA [Mohana, 2014]	$C_5H_9NO_3$, $C_4H_6O_6$<br>4-hydroxypyrrolidinium-2-carboxylate 2,3-dihydroxysuccinate {4-hydroxy-*L*-proline *L*-tartaric acid}	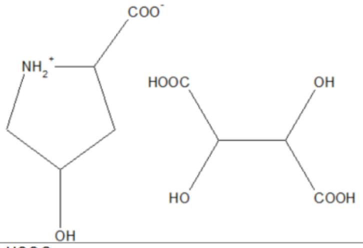
CXPROL [Dupont, 1978]	$C_6H_9NO_4$<br>*trans*-4-carboxy-*L*-proline	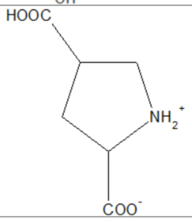
CUDCAG [Best, 2009]	$C_6H_{11}NO_5$<br>(2*S*,3*R*,4*R*,5*R*)-3,4-dihydroxy-5-(hydroxymethyl)pyrrolidine-2-carboxylic acid	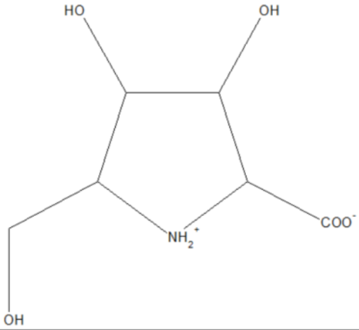
DHPROL10 [Karle, 1970]	$C_5H_9NO_4$<br>2,3-cis-3,4-trans-3,4-dihydroxy-*L*-proline	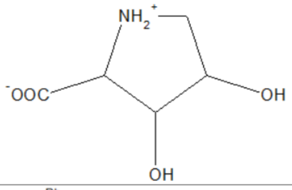
DIDKXIA [Meesakul, 2020]	$C_{13}H_{10}O_2$<br>6-(2-phenylethenyl)-2H-pyran-2-one {(E)-6-styrylpyran-2-one}	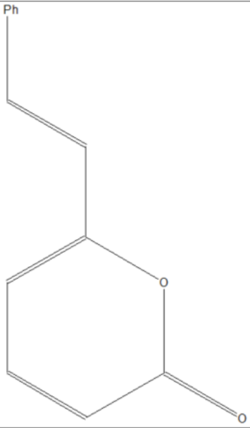
DIDXOG [Davies, 2018]	$C_5H_9NO_3$, H_2O<br>3-hydroxypyrrolidin-1-ium-2-carboxylate monohydrate	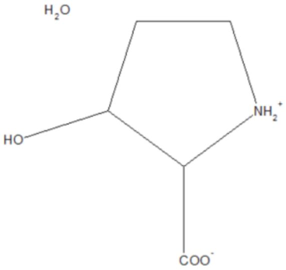
DIZXOB [Pandey, 2014]	$C_{12}H_{12}N_2O_6$, H_2O<br>4-((4-nitrobenzoyl)oxy)pyrrolidinium-2-carboxylate monohydrate	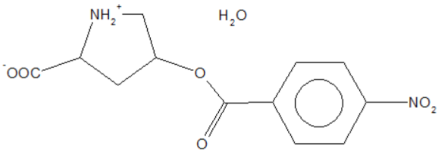
DLPROM(01, **02**, 03) [Fraig, 2002]	$C_5H_9NO_2$, H_2O<br>*D*,*L*-proline monohydrate	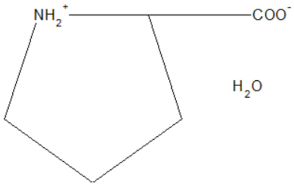

DUKJUP [Muramulla, 2009]	$C_{29}H_{28}F_6N_4OS$, $C_5H_9NO_2$, CH_4O<br>(*S*,*R*,*R*,*S*,*S*)-1-[3,5-bis(trifluoromethyl)phenyl]-3-[(5-ethenyl-1-azabicyclo[2.2.2]octan-2-yl)(6-methoxyquinolin-4-yl)methyl]thiourea *L*-proline methanol solvate	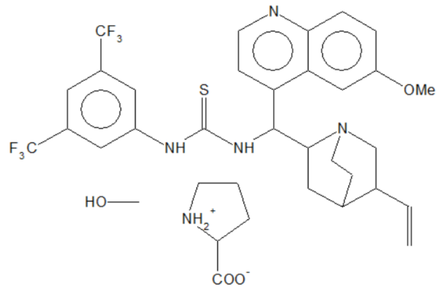
DUMBUK(**01**) [Pinalli, 2016]	$C_{36}H_{36}O_{12}P_4$, $C_5H_9NO_3$, $3.55(C_2H_3F_3O)$<br>5,11,17,23-tetramethyl-4,24:6,10:12,16:18,22-O,O'-tetrakis(methyl(oxo)phosphanidene)calix[4]resorcinarene 4-hydroxypyrrolidinium-2-carboxylate 2,2,2-trifluoroethanol solvate	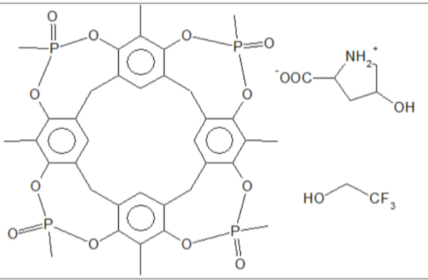
EJEPOA [He, 2016]	$C_{15}H_{10}O_6$, $2(C_5H_9NO_2)$<br>3,5,7-trihydroxy-2-(4-hydroxyphenyl)-4H-chromen-4-one bis(pyrrolidinium-2-carboxylate) {kaempferol bis(*L*-proline)}	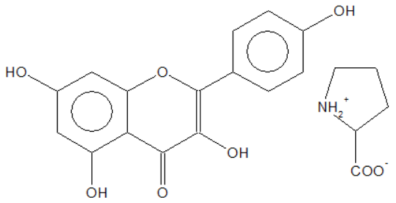
EJEPUG [He, 2016]	$C_{15}H_{10}O_6$, $C_5H_9NO_2$<br>2-(3,4-dihydroxyphenyl)-5,7-dihydroxy-4H-chromen-4-one pyrrolidinium-2-carboxylate {luteolin *L*-proline}	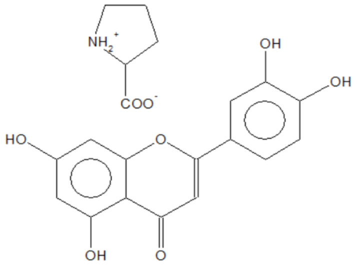
EJEQAN [Hongyan, 2016]	$C_{15}H_{10}O_4$, $C_5H_9NO_2$<br>5,7-dihydroxy-2-phenyl-4H-chromen-4-one pyrrolidinium-2-carboxylate {chrysin *L*-proline}	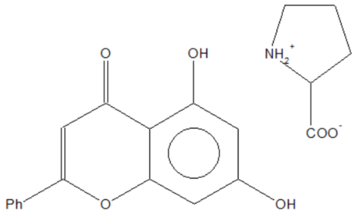
EJEQER [He, 2016]	$C_{15}H_{10}O_5$, $2(C_5H_9NO_2)$<br>5,7-dihydroxy-3-(4-hydroxyphenyl)-4H-chromen-4-one bis(pyrrolidinium-2-carboxylate) {genistein bis(*L*-proline)}	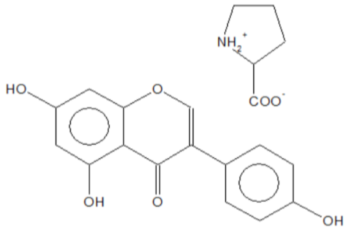
EJEQIV [He, 2016]	$C_{15}H_{10}O_6$, $C_5H_9NO_2$<br>pyrrolidinium-2-carboxylate 2-(3,4-dihydroxyphenyl)-5,7-dihydroxy-4H-chromen-4-one {luteolin *D*-proline}	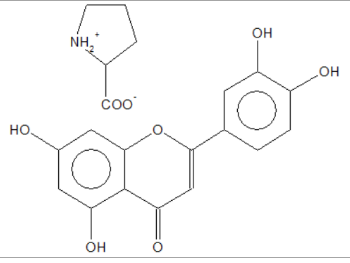
EJEQOB [He, 2016]	$C_{15}H_{10}O_4$, $C_5H_9NO_2$<br>pyrrolidinium-2-carboxylate 5,7-dihydroxy-2-phenyl-4H-chromen-4-one {chrysin *D*-proline}	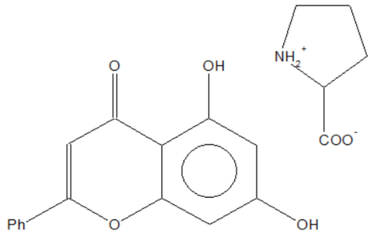

EJEQUH [He, 2016]	$C_{15}H_{10}O_5$, $2(C_5H_9NO_2)$ 5,7-dihydroxy-3-(4-hydroxyphenyl)-4H-chromen-4-one bis(pyrrolidinium-2-carboxylate) {genistein bis (*D*-proline)}	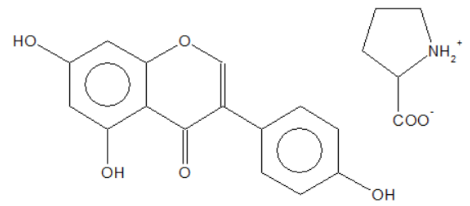
EJERAO [He, 2016]	$C_{15}H_{10}O_7$, $2(C_5H_9NO_2)$ 2-(3,4-dihydroxyphenyl)-3,5,7-trihydroxy-4H-chromen-4-one bis(pyrrolidinium-2-carboxylate) {quercetin *bis*(*D*-proline)}	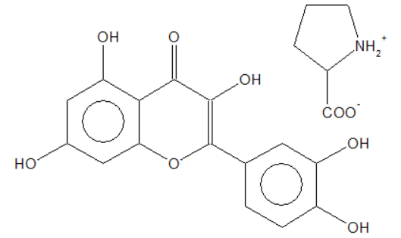
EJERES [He, 2016]	$C_{15}H_{10}O_7$, $2(C_5H_9NO_2)$ 2-(3,4-dihydroxyphenyl)-3,5,7-trihydroxy-4H-chromen-4-one bis(pyrrolidinium-2-carboxylate) {quercetin bis(*L*-proline)}	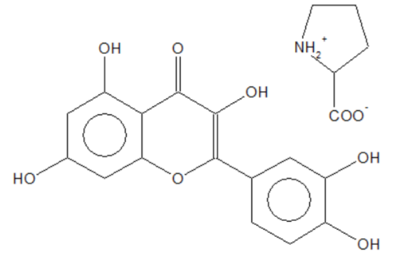
EJERIW [He, 2016]	$C_{15}H_{10}O_6$, $2(C_5H_9NO_2)$ 3,5,7-trihydroxy-2-(4-hydroxyphenyl)-4H-chromen-4-one bis(pyrrolidinium-2-carboxylate) {kaempferol bis(*D*-proline)}	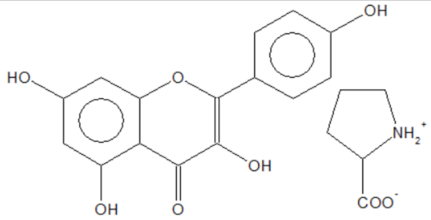
EQIHUI [Mykhailiuk, 2011]	C6H10FNO2, H2O 5-(fluoromethyl)pyrrolidinio-2-carboxylate monohydrate	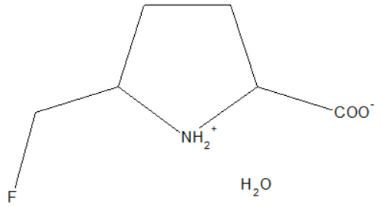
FEVZOX [Tilborg, 2013]	$C_{14}H_{14}O_3$, $C_5H_9NO_2$ (2*S*)-2-(6-methoxynaphthalen-2-yl)propanoic acid (*R*)-pyrrolidinium-2-carboxylate {*D*-proline *S*-naproxen}	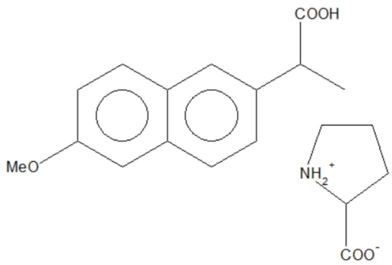
FEVZUD [Tilborg, 2013]	$C_{14}H_{14}O_3$, $C_5H_9NO_2$ (2*S*)-2-(6-methoxynaphthalen-2-yl)propanoic acid (*S*)-pyrrolidinium-2-carboxylate {(*L*)-proline (*S*)-naproxen}	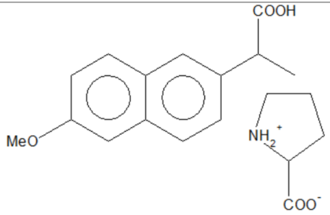
GAYCEO [Krapcho, 1988]	$C_{11}H_{13}NO_2$ trans-4-phenyl-*L*-proline	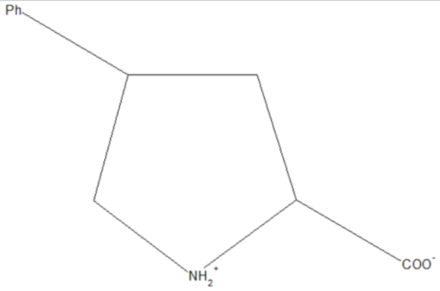

GEVMOK<br>[Hanessian, 2006]	$C_6H_9NO_2$<br>4,5-methano-*L*-proline	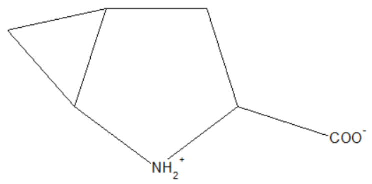
GIHSAT<br>[Tilborg, 2013]	$2(C_5H_9NO_2)$, $C_4H_4O_4$<br>bis(rac-pyrrolidinium-2-carboxylate) (2E)-but-2-enedioic acid {bis(D,L-proline) fumaric acid	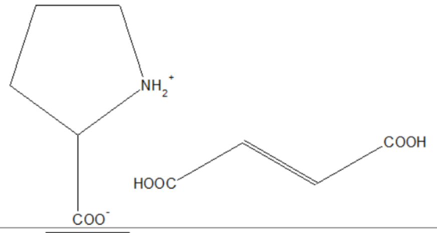
GIHSOH<br>[Tilborg, 2013]	$2(C_5H_9NO_2)$, $C_4H_4O_4$<br>bis((*S*)-pyrrolidinium-2-carboxylate) (2E)-but-2-enedioic acid { bis(*L*-proline) fumaric acid}	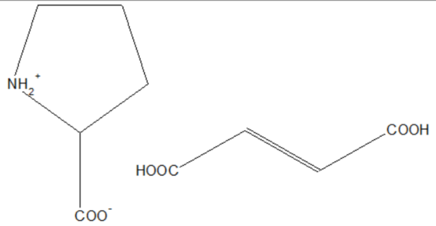
GIHSUN<br>[Tilborg, 2013]	$2(C_5H_9NO_2)$, $C_4H_4O_4$<br>bis((*R*)-pyrrolidinium-2-carboxylate) (2E)-but-2-enedioic acid { bis(*D*-proline) fumaric acid}	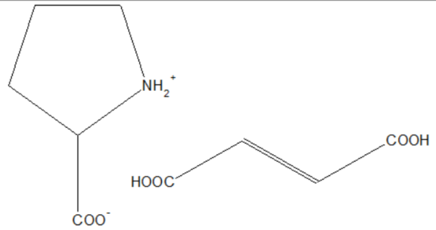
GIVROS<br>[Ramanathan, 1998]	$C_{18}H_{14}O_4$, $C_5H_9NO_2$<br>(11*R*,12*R*)-(+)-9,10-dihydro-9,10-ethanoanthracene-11,12-dicarboxylic acid (*S*)-proline	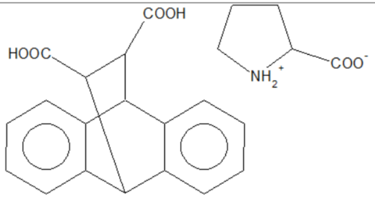
GULCUM [Cini, 2009]	$C_{10}H_{19}NO_2$<br>(5*R*)-*cis*-5-(3-methylbutyl)pyrrolidinium-2-carboxylate	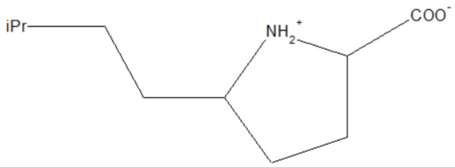
HOPROL(01,**12)**<br>[Koetzke, 1973]	$C_5H_9NO_3$<br>4-hydroxy-L-proline	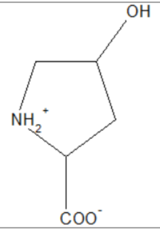
HXMPRO<br>[Koyama, 1974]	$C_6H_{11}NO_3$<br>3-hydroxy-4-methyl-proline	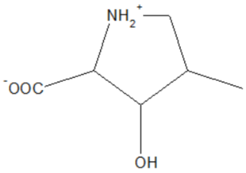
IDINAK<br>[Pandiarajan, 2002]	$C_5H_9NO_2$, $C_5H_{10}NO_2^+$, ClO_4^-<br>bis(*L*-proline) hydrogen perchlorate	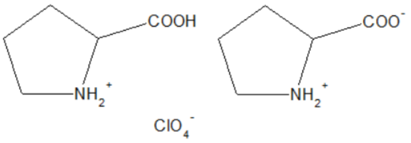

IHUMAZ [Timofeeva, 2003]	$C_{10}H_6N_2O$, $C_5H_9NO_2$ 1,1-dicyano-2-(4-hydroxyphenyl)ethene *L*-proline	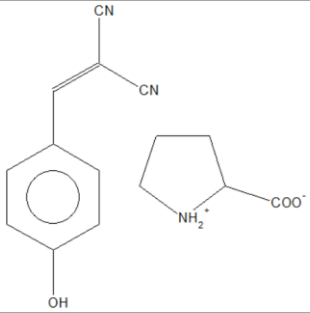
JOFGOD [Song, 2019]	$C_4H_6N_4O_3S_2$, $C_5H_9NO_2$ pyrrolidin-1-ium-2-carboxylate N-(5-sulfamoyl-1,3,4-thiadiazol-2-yl)acetamide	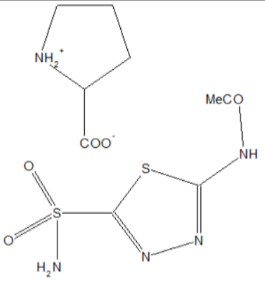
JOKLEC [Bedekar, 2014]	$C_{20}H_{14}O_4$, $2(C_5H_9NO_2)$ pyrrolidinium-2-carboxylate 1,1'-binaphthalene-2,2',7,7'-tetrol	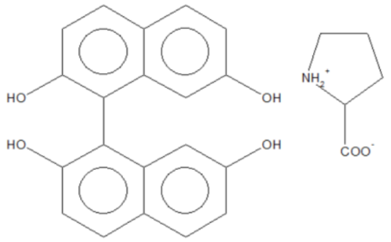
KECJIM [Rogowska, 2006]	$C_8H_{11}BO_3$, $C_5H_9NO_2$ *L*-proline 4-ethoxyphenylboronic acid	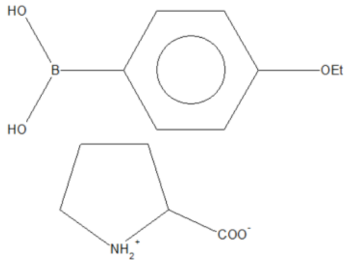
KEFWAU [Maverick, 1989]	$C_{20}H_{20}N_2NiO_2$ trans-bis(3-amino-1-phenylbut-2-en-1-onato-N,O)-nickel(ii)	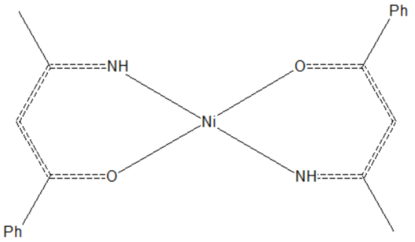
KIJFOB [Liang, 2018]	$C_{10}H_{15}NO_4$, H_2O 3-(carboxymethyl)-4-(prop-1-en-2-yl)pyrrolidin-1-ium-2-carboxylate monohydrate	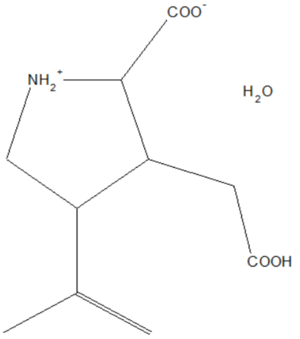
LABZUJ [Sridhar, 1993]	$C_5H_9NO_2$, $0.5(C_4H_6O_4)$ *D,L*-proline hemisuccinic acid	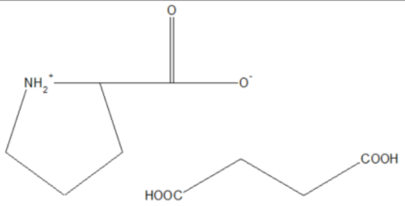

LEGBEG [Hobart, 2012]	$C_5H_8FNO_2$<br>(2*S*,4*R*)-4-Fluoropyrrolidinium-2-carboxylate {trans-4-fluoroproline}	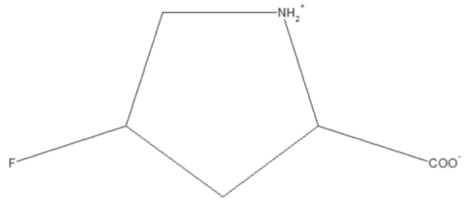
LIRSEN [Surov, 2018]	$C_{13}H_9F_3N_2O_2$, $C_5H_9NO_2$<br>2-((3-(trifluoromethyl)phenyl)amino)pyridine-3-carboxylic acid pyrrolidin-1-ium-2-carboxylate {niflumic acid *L*-proline}	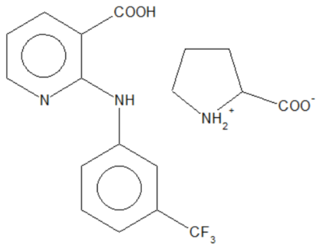
LOXCOS [Hernandez, 2015]	$C_6H_8B_2O_4$, $2(C_5H_9NO_2)$<br>bis(pyrrolidinium-2-carboxylate) 1,4-phenylenediboronic acid	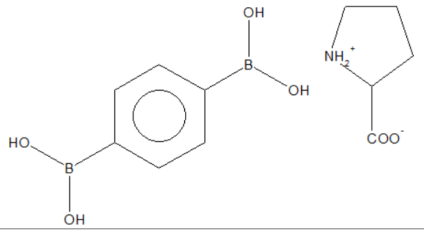
LOXDIN [Hernandez, 2015]	$C_5H_9NO_2$, $C_6H_7BO_2$, H_2O<br>pyrrolidinium-2-carboxylate phenylboronic acid monohydrate	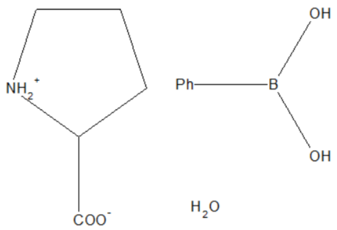
LOXHAJ(01) [Hernandez, 2015]	$C_5H_9NO_2$, $C_6H_6BIO_2$<br>*L*-proline (4-iodophenyl)boronic acid	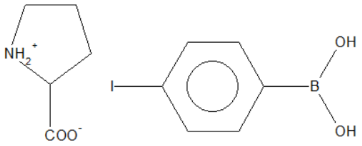
LOXHIR [Hernandez, 2015]	$C_5H_9NO_2$, $C_6H_7BO_2$<br>*L*-proline phenylboronic acid	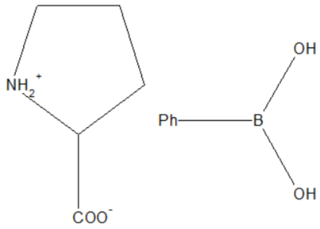
LUDFOF [Pandiarajan, 2002]	$C_5H_9NO_2$, $C_5H_{10}NO_2^+$, NO_3^-<br>bis(*L*-proline) hydrogen nitrate	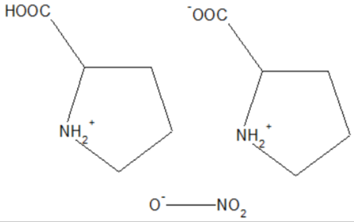
MEPROL [Bell, 1980]	$C_6H_9NO_2$, H_2O<br>2,4-methanoproline monohydrate {2-carboxy-2,4-methanopyrrolidine monohydrate}	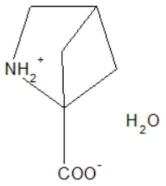
MPROLT [Fujimoto, 1971]	$C_6H_9NO_2$, H_2O<br>trans-3,4-methylene-*L*-proline monohydrate	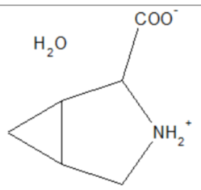

NAZGIG [Desphande, 2012]	$C_{21}H_{26}O_5$, $4(H_2O)$, $2(C_5H_9NO_2)$ (2*S*,3*R*,4*R*,5*S*,6*R*)-2-(3-(4-ethylbenzyl)-phenyl)-6-hydroxymethyl-tetrahydro-2H-pyran-3,4,5-triol bis(*L*-proline) tetrahydrate	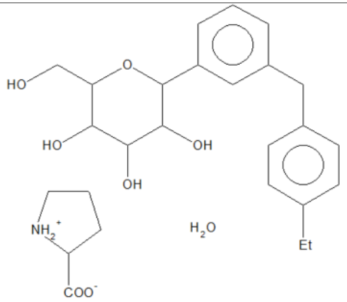
NAZGOM [Desphande, 2012]	$C_{21}H_{26}O_5$, H_2O, $2(C_5H_9NO_2)$, C_2H_6O (2*S*,3*R*,4*R*,5*S*,6*R*)-2-(3-(4-ethylbenzyl)-phenyl)-6-hydroxymethyl-tetrahydro-2H-pyran-3,4,5-triol bis(*L*-proline) ethanol solvate monohydrate	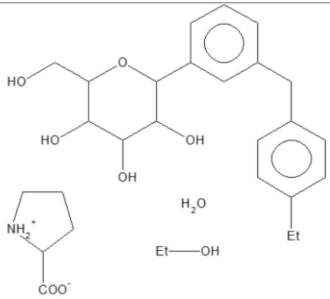
NAZGUS [Desphande, 2012]	$C_{21}H_{26}O_5$, $C_5H_9NO_2$ (2*S*,3*R*,4*R*,5*S*,6*R*)-2-(3-(4-ethylbenzyl)-phenyl)-6-hydroxymethyl-tetrahydro-2H-pyran-3,4,5-triol *L*-proline	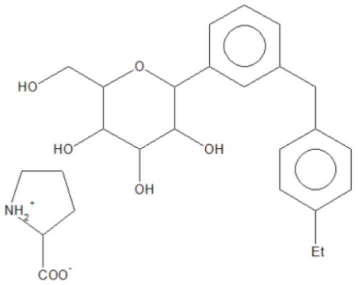
NEGBUW [Baldwin, 1997]	$C_{14}H_{17}NO_5$ (2*S*,3*S*,4*S*)-3-carboxymethyl-4-(2-methoxyphenyl)pyrrolidine-2-carboxylic acid	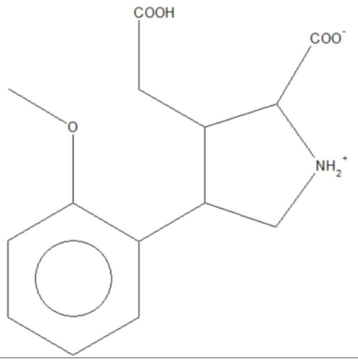
NISVOA(**<u>01</u>**) [Hu, 2012]	$2(C_{20}H_{14}O_2)$, $C_5H_9NO_2$ (*S*)-(-)-bis(1,1'-binaphth-2,2'-diol) (*S*)-proline	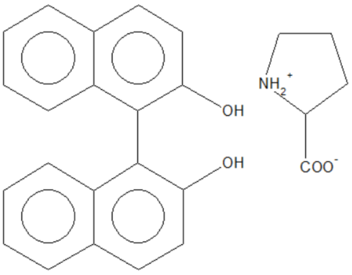
NOHTIP [Pangerl, 2014]	$C_{14}H_{19}NO_3$ 4-((benzyloxy)methyl)-5-methylpyrrolidinium-2-carboxylate	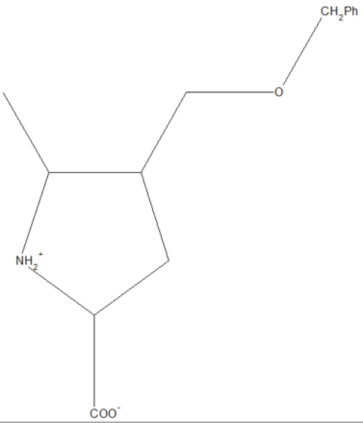

OLIZAL [Qu, 2011]	$2(C_5H_9NO_2)$, $2(C_5H_{10}NO_2^+)$, $2(C_{12}H_4N_4^-)$, $C_{12}H_4N_4$ bis(2-carboxypyrrolidinium) bis(7,7,8,8-tetracyanoquinodimethanide) bis(tetracyanoquinodimethane) pyrrolidinium-2-carboxylate	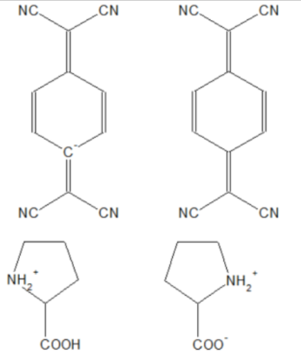
PEBZOO [He, 2017]	$C_{14}H_{12}O_3$, $C_5H_9NO_2$ pyrrolidinium-2-carboxylate 5-(2-(4-hydroxyphenyl)vinyl)benzene-1,3-diol {*L*-proline resveratrol}	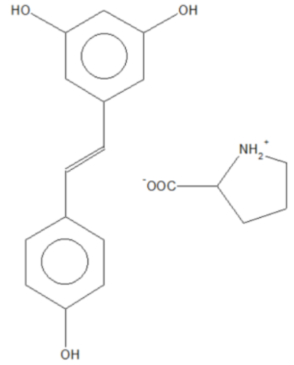
PEBZUU [He, 2017]	$C_{14}H_{12}O_3$, $2(C_5H_9NO_2)$ bis(pyrrolidinium-2-carboxylate) 5-(2-(4-hydroxyphenyl)vinyl)benzene-1,3-diol {bis(*L*-proline) resveratrol}	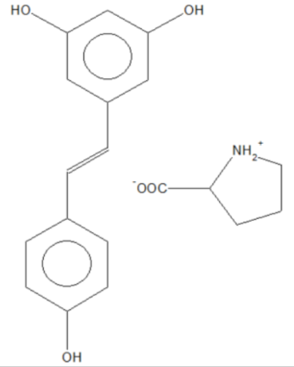
PEGZAE [Venkatesan, 2013]	$C_{10}H_{18}Br_2N_2O_4Zn$ dibromo-(bis(2-(carboxy)pyrrolidiniumato))-zinc(ii)	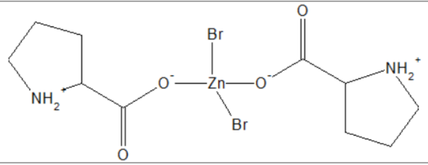
POKHAY10 [Fu, 1997]	$C_{23}H_{28}O_3$, $C_5H_9NO_2$ 4-(2,4,6-tri-isopropylbenzoyl)benzoic acid proline	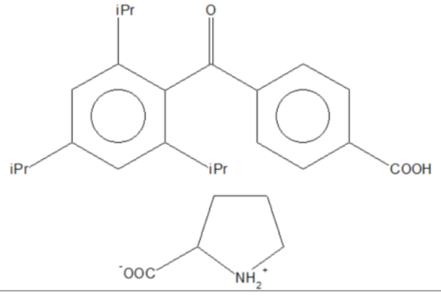
PROLIN(01-05)	*See scheme 1, 2*	
QANRUT(01)	*See scheme 1, 2*	
RUWGEV [Janczak, 1997]	$C_5H_9NO_2$, H_2O *L*-proline monohydrate	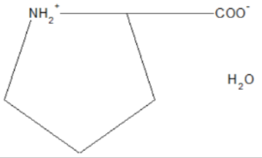
QILZET [Tumanova, 2018]	$C_{14}H_{14}O_3$, $2(C_5H_9NO_2)$ (2*S*)-2-(6-methoxynaphthalen-2-yl)propanoic acid (2*R*)-pyrrolidin-1-ium-2-carboxylate (2*S*)-pyrrolidin-1-ium-2-carboxylate {(*S*)-naproxen bis(*D*,*L*-proline)}	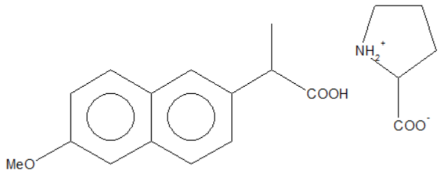

QILZIX [Tumanova, 2018]	$C_{14}H_{14}O_3$, 2($C_5H_9NO_2$) (2*S*)-2-(6-methoxynaphthalen-2-yl)propanoic acid bis((2S)-pyrrolidin-1-ium-2-carboxylate) { (S)-naproxen bis(*L*-proline)}	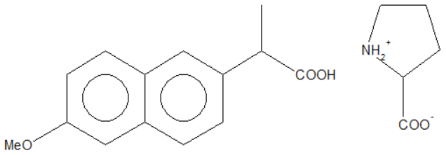
QILZOD [Tumanova, 2018]	$C_{14}H_{14}O_3$, $C_5H_9NO_2$ bis((2*S*)-2-(6-methoxynaphthalen-2-yl)propanoic acid) (2R)-pyrrolidin-1-ium-2-carboxylate (2*S*)-pyrrolidin-1-ium-2-carboxylate {(*S*)-naproxen (*D*,*L*)-proline}	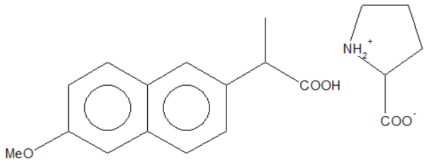
QILZUJ [Tumanova, 2018]	$C_{14}H_{14}O_3$, $C_5H_9NO_2$, 0.5(CH_4O) (2*R*)-2-(6-methoxynaphthalen-2-yl)propanoic acid (2*S*)-2-(6-methoxynaphthalen-2-yl)propanoic acid bis((2*R*)-pyrrolidine-2-carboxylic acid) methanol solvate {(*RS*)-naproxen (*D*)-proline methanol solvate}	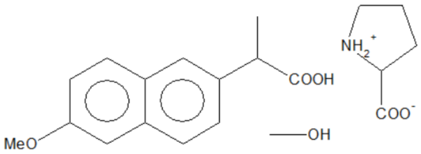
QIMBAS(01) [Tumanova, 2018]	$C_{14}H_{14}O_3$, $C_5H_9NO_2$ (2*R*)-2-(6-methoxynaphthalen-2-yl)propanoic acid (2*S*)-2-(6-methoxynaphthalen-2-yl)propanoic acid bis((2*R*)-pyrrolidin-1-ium-2-carboxylate) {(*R*,*S*)-naproxen (*D*)-proline}	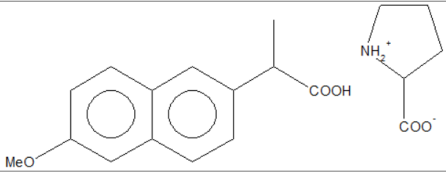
QIMBEW(01) [Tumanova, 2018]	$C_{14}H_{14}O_3$, $C_5H_9NO_2$ (2*R*)-2-(6-methoxynaphthalen-2-yl)propanoic acid (2*S*)-2-(6-methoxynaphthalen-2-yl)propanoic acid (2R)-pyrrolidin-1-ium-2-carboxylate (2*S*)-pyrrolidin-1-ium-2-carboxylate {(*RS*)-naproxen (*DL*)-proline}	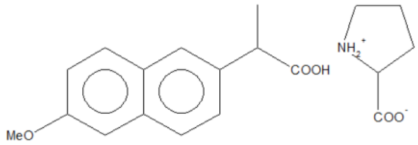
QIMBIA [Tumanova, 2018]	$C_{14}H_{14}O_3$, $C_5H_9NO_2$, H_2O bis((2*S*)-2-(6-methoxynaphthalen-2-yl)propanoic acid) (2*R*)-pyrrolidin-1-ium-2-carboxylate (2*S*)-pyrrolidin-1-ium-2-carboxylate monohydrate {(S)-naproxen (*DL*)-proline monohydrate}	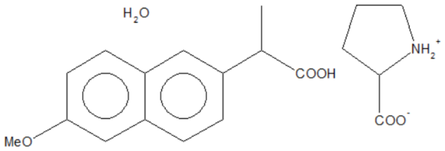
QIMBOG [Tumanova, 2018]	$C_{14}H_{14}O_3$, 2($C_5H_9NO_2$) (2*R*)-2-(6-methoxynaphthalen-2-yl)propanoic acid (2*S*)-2-(6-methoxynaphthalen-2-yl)propanoic acid tetrakis((2*S*)-pyrrolidine-2-carboxylic acid) {(*RS*)-naproxen bis(*L*-proline)}	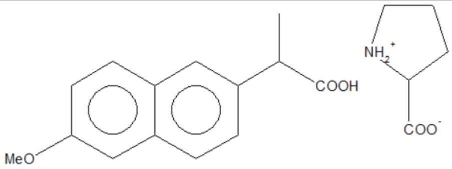
QIMCOH [Tumanova, 2018]	$C_{14}H_{14}O_3$, $C_5H_9NO_2$ (2*R*)-2-(6-methoxynaphthalen-2-yl)propanoic acid (2*S*)-2-(6-methoxynaphthalen-2-yl)propanoic acid bis((2S)-pyrrolidin-1-ium-2-carboxylate) {(*RS*)-naproxen (*L*)-proline}	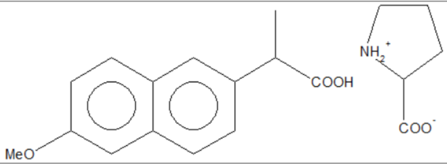
QIMCUN [Tumanova, 2018]	2($C_{14}H_{14}O_3$), 3($C_5H_9NO_2$) bis((2*S*)-2-(6-methoxynaphthalen-2-yl)propanoic acid) tris((2*S*)-pyrrolidin-1-ium-2-carboxylate) {bis((*S*)-naproxen) tris((*L*)-proline)}	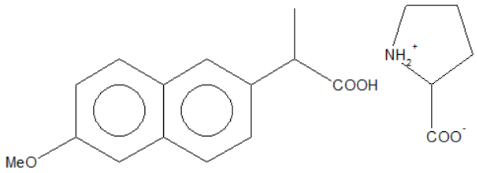
QIRNUC [Sowmya, 2013]	$C_5H_9NO_2$, 2($C_6H_5NO_3$) pyrrolidinium-2-carboxylate bis(4-nitrophenol)	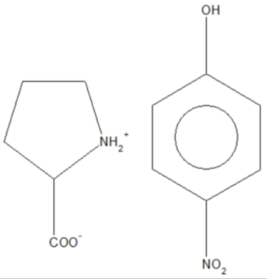
RETNEM(01) [Nugrahani, 2018]	$C_{14}H_{11}Cl_2NO_2$, $C_5H_9NO_2$ 2-(2-(2,6-dichloroanilino)phenyl)acetic acid (2S)-pyrrolidinium-2-carboxylate {diclofenac *L*-proline}	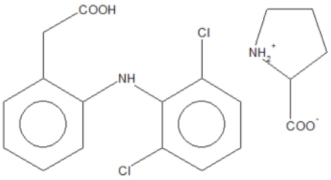

RUWGEV [Janczak, 1997]	$C_5H_9NO_2$, H_2O<br>*L*-proline monohydrate	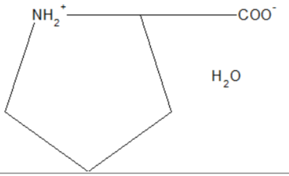
TIWZUV [Kumar, 2008]	$C_{15}H_{17}NO_5$, H_2O<br>8,9-dimethoxy-5-oxo-2,3,5,6-tetrahydropyrrolo[2,1-a]isoquinoline-10b(1H)-carboxylic acid monohydrate	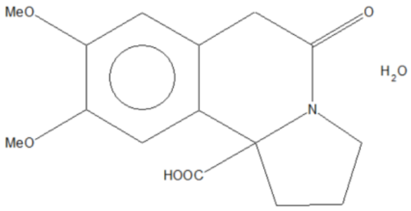
TOSKOD [Priya, 2015]	$C_{20}H_{21}N_3O_6S$<br>1-((2-((2-acetamidobenzoyl)amino)phenyl)sulfonyl)proline	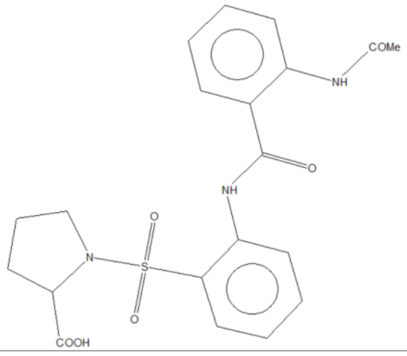
TPHPRO13 [Lu, 2018]	$C_{17}H_{22}N_2O_6S$, H_2O<br>tosyl-*L*-prolyl-*L*-hydroxyproline monohydrate	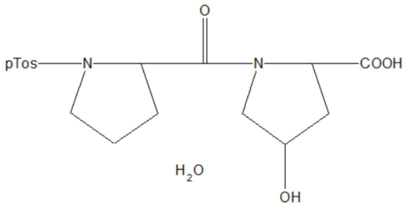
TUGWAV(01) [Pinalli, 2016]	$C_{36}H_{36}O_{12}P_4$, $C_5H_9NO_2$, 2.6($C_2H_3F_3O$)<br>pyrrolidinium-2-carboxylate 5,11,17,23-tetramethyl-6,10:12,16:18,22:24,4-tetrakis(methylphosphonato-O,O')calix(4)arene 2,2,2-trifluoroethanol solvate {pyrrolidinium-2-carboxylate 5,11,17,23-tetramethyl-6,10:12,16:18,22:24,4-O,O'-tetrakis(methylphosphoryl)calix(4)resorcinarene 2,2,2-trifluoroethanol solvate}	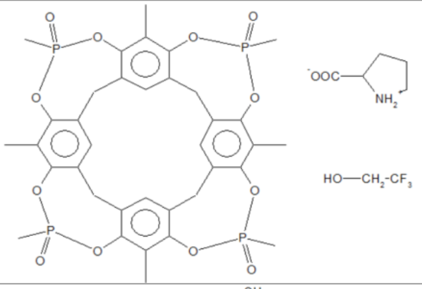
UPIKUB [Scholmeyer, 2016]	$C_5H_9NO_3$<br>4-hydroxypyrrolidinium-2-carboxylate {4-hydroxyproline}	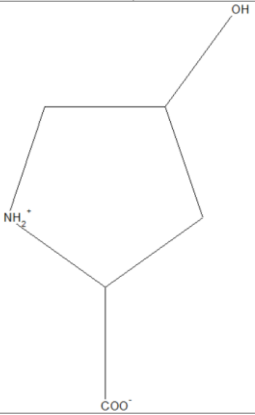
VESCUS [Marchand, 2006]	$C_{12}H_{12}O_4$, $C_5H_9NO_2$<br>pentacyclo(5.3.0.02,5.03,9.04,8)decane-2,5-dicarboxylic acid proline	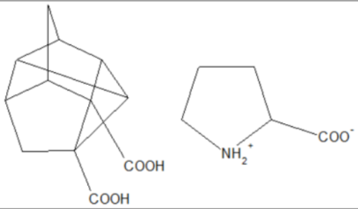

VEVKEP(01) [Tumanova, 2018]	$C_{15}H_{13}FO_2$, $C_5H_9NO_2$<br>(2*R*)-2-(2-fluoro[1,1'-biphenyl]-4-yl)propanoic acid (2*S*)-pyrrolidin-1-ium-2-carboxylate {(*R*)-flurbiprofen *L*-proline}	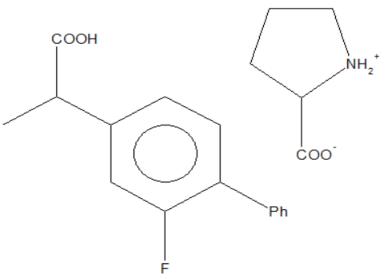
VEVKOZ [Tumanova, 2018]	$2(C_{15}H_{13}FO_2)$, $C_5H_9NO_2$<br>bis((2*R*)-2-(2-fluoro-[1,1'-biphenyl]-4-yl)propanoic acid) (2*S*)-pyrrolidin-1-ium-2-carboxylate { bis((*R*)-flurbiprofen) *L*-proline}	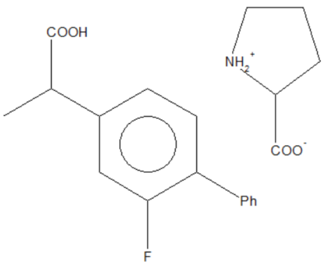
VEVKUF [Tumanova, 2018]	$C_{15}H_{13}FO_2$, $3(C_5H_9NO_2)$<br>(2*R*)-2-(2-fluoro-[1,1'-biphenyl]-4-yl)propanoic acid tris((2*S*)-pyrrolidin-1-ium-2-carboxylate) {(R)-flurbiprofen tris(*L*-proline)}	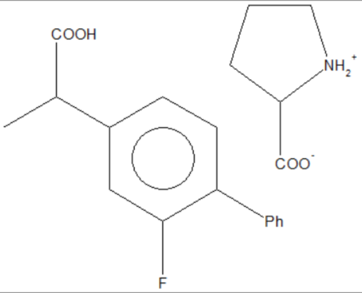
VEVLAM [Tumanova, 2018]	$C_{19}H_{13}FO_2$, $2(C_5H_9NO_2)$<br>(2*R*)-2-(2-fluoro[1,1'-biphenyl]-4-yl)propanoic acid bis((2*S*)-pyrrolidin-1-ium-2-carboxylate) {(*R*)-flurbiprofen bis((*L*)-proline)}	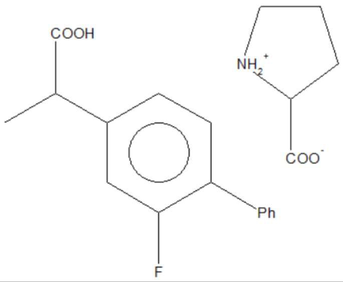
VEVLEQ(01) [Tumanova, 2018]	$C_{15}H_{13}FO_2$, $C_5H_9NO_2$, $0.5(CH_4O)$<br>(2*R*)-2-(2-fluoro[1,1'-biphenyl]-4-yl)propanoic acid (2*S*)-pyrrolidin-1-ium-2-carboxylate methanol solvate {(*R*)-flurbiprofen *L*-proline methanol solvate}	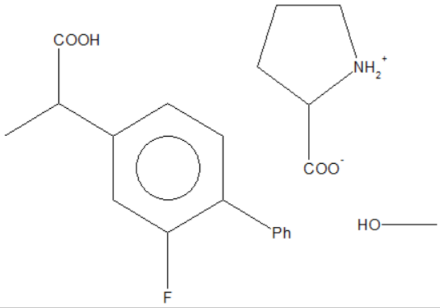
VEVLOA [Tumanova, 2018]	$C_{15}H_{13}FO_2$, $C_5H_9NO_2$, $0.5(C_2H_6O)$<br>(2*R*)-2-(2-fluoro[1,1'-biphenyl]-4-yl)propanoic acid (2*S*)-pyrrolidin-1-ium-2-carboxylate ethanol solvate {(*R*)-flurbiprofen *L*-proline ethanol solvate}	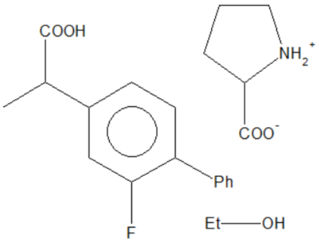
VEVLUG [Tumanova, 2018]	$2(C_{15}H_{13}FO_2)$, $C_5H_9NO_2$<br>bis((2*R*)-2-(2-fluoro-[1,1'-biphenyl]-4-yl)propanoic acid) (2*R*)-pyrrolidin-1-ium-2-carboxylate {bis((*R*)-flurbiprofen) *D*-proline}	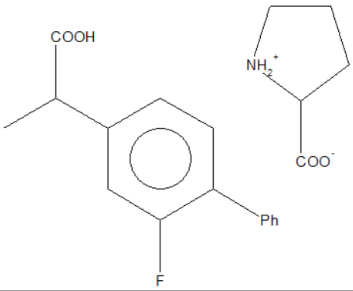

VEVMAN [Tumanova, 2018]	$C_{15}H_{13}FO_2$, $C_5H_9NO_2$ (2*R*)-2-(2-fluoro[1,1'-biphenyl]-4-yl)propanoic acid (2*R*)-pyrrolidin-1-ium-2-carboxylate {(R)-flurbiprofen *D*-proline}	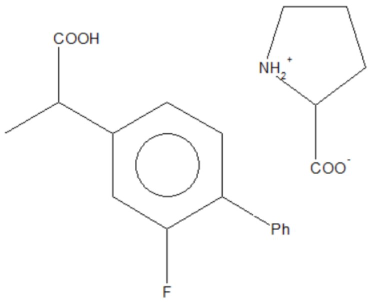
VEVMER [Tumanova, 2018]	$2(C_{15}H_{13}FO_2)$, $C_5H_9NO_2$ bis((2*R*)-2-(2-fluoro[1,1'-biphenyl]-4-yl)propanoic acid) rac-pyrrolidin-1-ium-2-carboxylate { bis((R)-flurbiprofen) *D*,*L*-proline}	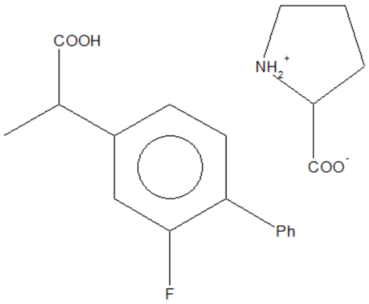
VEVMIV [Tumanova, 2018]	$C_{15}H_{13}FO_2$, $C_5H_9NO_2$ (2*R*)-2-(2-fluoro-[1,1'-biphenyl]-4-yl)propanoic acid rac-pyrrolidin-1-ium-2-carboxylate {(*R*)-flurbiprofen *DL*-proline}	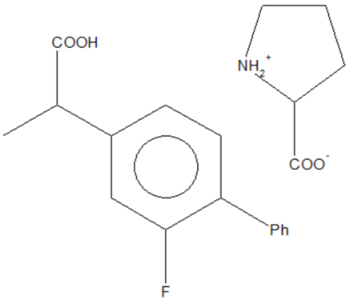
VEVMOB(01) [Tumanova, 2018]	$C_{15}H_{13}FO_2$, $C_5H_9NO_2$ rac-2-(2-fluoro-[1,1'-biphenyl]-4-yl)propanoic acid (2*R*)-pyrrolidin-1-ium-2-carboxylate {rac-flurbiprofen *D*-proline}	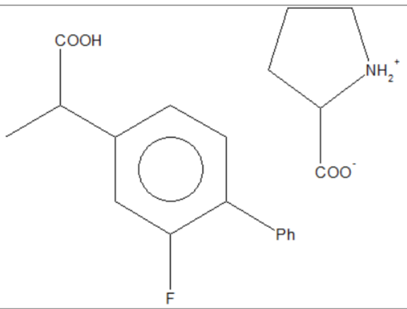
VEVMUH(01) [Tumanova, 2018]	$C_{15}H_{13}FO_2$, $C_5H_9NO_2$ rac-2-(2-fluoro[1,1'-biphenyl]-4-yl)propanoic acid (2*S*)-pyrrolidin-1-ium-2-carboxylate {rac-flurbiprofen *L*-proline}	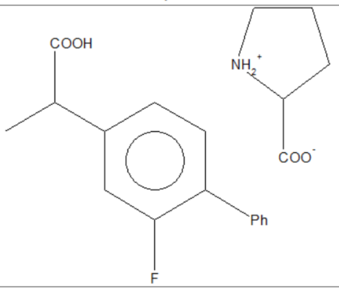
VEVNIW [Tumanova, 2018]	$2(C_{15}H_{13}FO_2)$, $C_5H_9NO_2$ bis(rac-2-(2-fluoro-[1,1'-biphenyl]-4-yl)propanoic acid) (2*S*)-pyrrolidin-1-ium-2-carboxylate {bis(rac-flurbiprofen) *L*-proline}	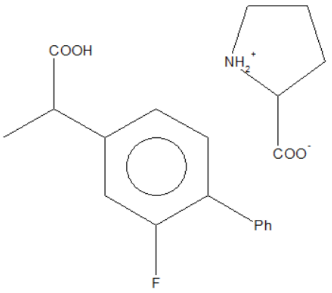

VEVNOC [Tumanova, 2018]	$C_{15}H_{13}FO_2$, $C_5H_9NO_2$ rac-2-(2-fluoro-[1,1'-biphenyl]-4-yl)propanoic acid rac-pyrrolidin-1-ium-2-carboxylate { rac-flurbiprofen *DL*-proline}	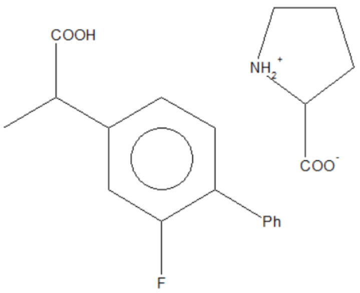
VIOABR10 [Coggon, 1970]	$C_8H_{15}N_5O_{22}^{+}$, 2(Br^-), 3(H_2O) viocidic acid dihydrobromide trihydrate	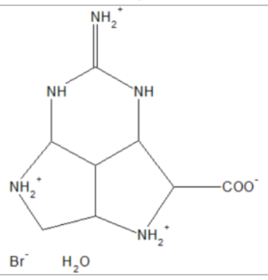
WERMIQ [Klussman, 2006]	$C_5H_9NO_2$, 0.5($CHCl_3$) pyrrolidinium-2-carboxylate chloroform solvate {*DL*-proline chloroform solvate}	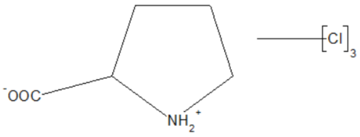
XEGKIG [Ambika, 2017]	$C_5H_9NO_2$, 2($C_7H_4Cl_2O_2$), 0.5(H_2O) *L*-proline bis(2,4-dichlorobenzoic acid) hemihydrate	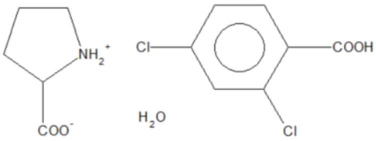
YEBLUO [Fuijsawa, 2012]	$C_{36}H_{40}O_{12}$, $C_5H_9NO_2$, C_2H_6O, 3(H_2O) 2,8,14,20-tetraethylpentacyclo[19.3.1.13,7.19,13.115,19]octacosa-1(25),3(28),4,6,9(27),10,12,15(26),16,18,21,23-dodecaene-4,5,6,10,11,12,16,17,18,22,23,24-dodecol *DL*-proline ethanol solvate trihydrate {C-ethylpyrogallo(4)arene *DL*-proline ethanol solvate trihydrate}	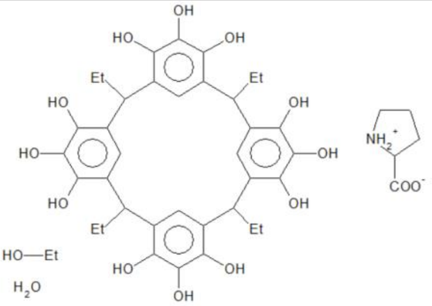
YEBMAV [Fuijsawa, 2012]	$C_{36}H_{40}O_{12}$, 2($C_5H_9NO_2$), 2(H_2O) 2,8,14,20-tetraethylpentacyclo[19.3.1.13,7.19,13.115,19]-octacosa-1(25),3(28),4,6,9(27),10,12,15(26),16,18,21,23-dodecaene-4,5,6,10,11,12,16,17,18,22,23,24-dodecol bis(*DL*-proline) dihydrate {C-ethylpyrogallo(4)arene bis(*DL*-proline) dihydrate}	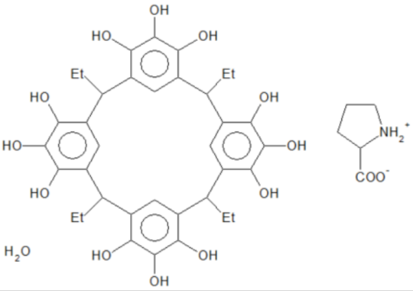
YEPJEL [Yadav, 2018]	$C_8H_5F_3N_2OS$, $C_5H_9NO_2$ pyrrolidin-1-ium-2-carboxylate 6-(trifluoromethoxy)-1,3-benzothiazol-2-amine {riluzole proline}	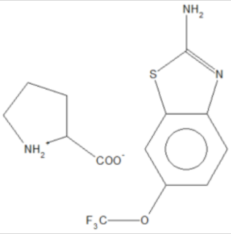
ZAPDAY [Chesna, 2017]	$C_5H_9NO_2$, $C_7H_6O_2$ pyrrolidin-1-ium-2-carboxylate benzoic acid	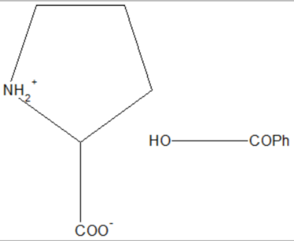

ZEZHIV [Aakeroy, 1995]	$C_5H_9NO_2$, $C_7H_6O_4$<br>*L*-proline 2,5-dihydroxybenzoic acid	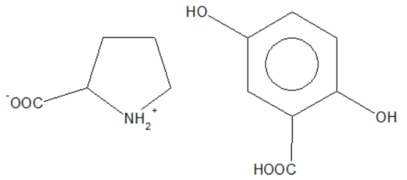
ZINPEU(**01**) [Surov, 2018]	$C_{13}H_{11}ClN_2O_2$, $C_5H_9NO_2$<br>pyrrolidin-1-ium-2-carboxylate 2-[(3-chloro-2-methylphenyl)amino]pyridine-3-carboxylic acid {clonixin proline}	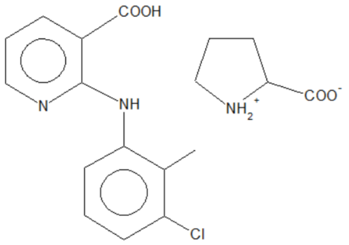
ZINPIY [Li, 2018]	$C_5H_9NO_2$, $C_4H_4FN_3O$<br>pyrrolidin-1-ium-2-carboxylate 4-amino-5-fluoropyrimidin-2(1H)-one {flucytosine proline}	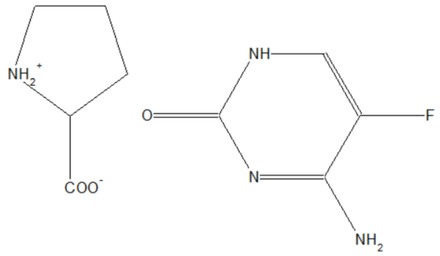
ZINPUK [Duanxiu, 2018]	$C_9H_{10}N_4O_2S_2$, $C_5H_9NO_2$<br>pyrrolidin-1-ium-2-carboxylate 4-amino-*N*-(5-methyl-1,3,4-thiadiazol-2(3H)-ylidene)benzene-1-sulfonamide {sulfamethizole proline}	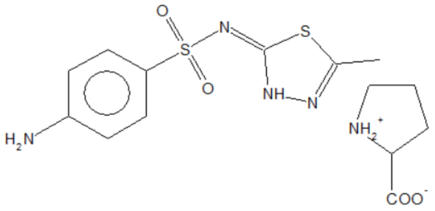
ZIZHAR [Oliver, 1995]	$C_{19}H_{26}N_2O_6$, H_2O<br>*N*-*t*-butoxycarbonyl-tyrosyl-proline monohydrate	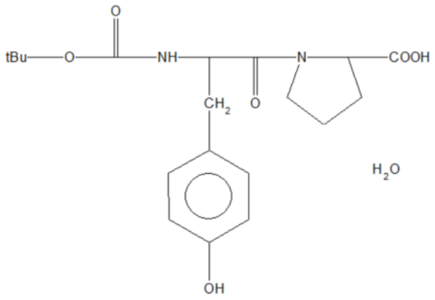

*see references below

Table S2. Chemical names of analyzed compounds.

CSD ref. code	*Name*
	Proline structures
PROLIN03	*L*-proline
QANRUT	*D,L*-proline
NELSEC	*R*-thioproline
	Proline-based ACEI
CIYNIH	1-(*N*-(1*S*)-carboxy-3-phenylpropyl)-*L*-alanyl)-*L*-proline trihydrate
DIVHOF01	*N*-(1-ethoxycarbonyl-3-phenylpropyl)-*L*-alanylium-*L*-proline hydrogen maleate
GERWUX01	1-(6-ammonio-2-((1-carboxylato-3-phenylpropyl)ammonio)hexanoyl)pyrrolidine-2-carboxylate dihydrate {synonyme: *N*2-[(1*S*)-1-carboxy-3-phenylpropyl]-*L*-lysyl-*L*-proline dihydrate}
GERXAE	1-(6-ammonio-*N*-(1-carboxy-3-phenylpropyl)norleucyl)pyrrolidine-2-carboxylate monohydrate
GERXEI	1-(6-ammonio-*N*-(1-carboxy-3-phenylpropyl)norleucyl)pyrrolidine-2-carboxylate
TUHMOY	sodium 4-cyclohexyl-1-(((2-methyl-1-(propionyloxy)propoxy)(4-phenylbutyl)phosphoryl)acetyl)pyrrolidine-2-carboxylate
TUHMUE	sodium hemi zofenopril

	Perindopril-derived compounds
IVEGIA	(2*S*,3a*S*,7a*S*)-1[(2*S*)-2-[[(2*S*)-1-ethoxy-1-oxopentan-2-yl]amino)propanoyl]2,3,3a,4,5,6,7,7a-octahydroindole-2-carboxylate-t-butylammonium dihydrate
UZOVAH03	(2*S*,3a*S*,7a*S*)-1[(2*S*)-2-[[(2*S*)-1-ethoxy-1-oxopentan-2-yl]amino)propanoyl]2,3,3a,4,5,6,7,7a-octahydroindole-2-carboxylate-t-butylaminum
BECWIR	perindoprilat dimethylsulfoxide solvate
FEFKEI	(1*S*)-2-((*S*)-(1-[2*S*,3a*S*,7a*S*)-(2-carboxyoctahydro-1H-indol-1-yl]-1-oxopropan-2yl)ammonio)pentanoate monohydrate
BILNAN	ethyl (2*S*)-2-[(3*S*,5a*S*,9a*S*,10a*S*)-3-methyl-1,4-dioxo-5a,6,7,8,9a,10,10a-octahydro-3H-pyrazino[1,2-alpha]indol-2-yl]pentanoate
BILNAN01	ethyl (2*S*)-2-[(3*S*,5a*S*,9a*S*,10a*S*)-3-methyl-1,4-dioxo-5a,6,7,8,9a,10,10a-octahydro-3H-pyrazino[1,2-alpha]indol-2-yl]pentanoate
	Other modified proline-based ACEI
EDALEC	1,3-dihydroxy-2-(hydroxymethyl)propan-2-aminium-1-(*N*-(ethoxy-1-oxo-4-phenylbutan-2-yl)alanyl)octahydrocyclopenta)[b]pyrrole-2-carboxylate<br>{synonyme: 1,3-dihydroxy-2-(hydroxymethyl)propan-2-aminium ramiprilate}
FIFGEG	2-(*N*-((*S*)-1-carboxy-3-phenylpropyl)-L-alanyl)-(1*S*,3*S*,5*S*)-2-azabicyclo(3.3.0)octane-3-caerboxylic acid methanol clathrtae
IQISAE	(2*S*,3a*S*,7a*S*)-1-[(2*S*)-2-[[(2*S*)-1-ethoxy-1oxo-4-phenylbutan-2-yl]amino]propanoyl]-2,3,3a,4,5,6,7,7a-octahydroindole-2-carboxylic acid
MCPRL01	1-(2-methyl-3-sulfanylpropanoyl)proline
YOZTIS	1,1`-(disulfanediylbis(2-methyl-1-oxopropane-3,1-diyl))dipyrrolidine-2-carboxylic acid
QOQWAU	1-(2-(1-ethoxycarbonyl-3-phenylpropylamino)propionyl)-octahydrocyclopenta(b)-pyrrole-2-carboxylic acid
RUWBAM	(8*S*-(7(*R**(*R**)),8*R**))-7-(2-((1-(ethoxycarbonyl)-3-phenylpropyl)amino)-1-oxopropyl)-2,4-dithia-7-azaspiro(4,4)nonane-8-carboxylic acid hydrochloride monohydrate

Table S3. Crystal data of proline, proline-based and modified-proline-based ACEI structures and their derivatives.

Proline structures and its analogue

compound name	***L*-proline**	***D,L*-proline**	***R*-thioproline**				
CCDC ref. code	**PROLIN03** [38], see the Article	**QANRUT** [176]	**NELSEC** [177]				
formula	$C_5H_9NO_2$	$C_5H_9N_1O_2$	$C_4H_7NO_2S$				
Crystal system	orthorhombic	monoclinic	orthorhombic				
Space group	$P2_12_12_1$	$P2_1/c$	$P2_12_12_1$				
Z	4	4	4				
Unit cell parameters [A, o]	*a*=5.28018(12),<br>*b*=8.8794(2),<br>*c*=11.5307(2)<br>*α*=90<br>*β*=90<br>*γ*=90	a=8.9906(6)<br>*b*=5.2987(4)<br>*c*=11.4786(8)<br>*α*=90<br>*β*=97.041(2)<br>*γ*=90	*a*=5.6733(3)<br>*b*=9.9375(6)<br>*c*=9.9407(6)<br>*α*=90 *β*=90<br>*γ*=90				
V [Å^3]	540.615	544.699	560.441				
R_1 *[I>2σ(I)]*	3.39	3.95	2.9				
T [K]	100	120	room				

Proline-based ACEI

compound name	**enalapril maleate**	**enalaprilat**	**lisinopril**	**lisinopril monohydrate**	**lisinopril dihydrate**	**captopril**	**Captopril disulfide**
CCDC ref. code	**DIVHOF01** [193]	**CIYNIH** [192]	**GERXEI** [194]	**GERXAE** [194]	**GERWUX01** [195]	**MCPRPL01** [B., 2015]	**YOZTIS** [188]
formula	$C_{20}H_{29}N_2O_5^+ \cdot C_4H_3O_4^-$	$C_{18}H_{24}N_2O_5 \cdot 3H_2O$	$C_{21}H_{31}N_3O_5$	$C_{21}H_{31}N_3O_5 \cdot H_2O$	$C_{21}H_{31}N_3O_5 \cdot 2(H_2O)$	$C_{19}H_{15}NO_3S$	$C_{18}H_{28}N_2O_6S_2$
Crystal system	monoclinic	orthorhombic	monoclinic	monoclinic	monoclinic	orthorhombic	monoclinic
Space group	$P2_1$	$P2_12_12_1$	$P2_1$	$P2_1$	$P2_1$	$P2_12_12_1$	$P2_1$
Z	2	4	2	2	2	4	2
Unit cell parameters [A, o]	*a*=11.224(4)<br>*b*=6.645(2)<br>*c*=17.824(5)<br>*α*=90<br>*β*=105.52(3)<br>*γ*=90	*a*=9.798(2)<br>*b*=10.452(2)<br>*c*=19.819(5)<br>*α*=90<br>*β*=90<br>*γ*=90	*a*=15.17734(29)<br>*b*=5.94528(8)<br>*c*=14.22942(26)<br>*α*=90<br>*β*=120.6221(9)<br>*γ*=90	*a*=14.66805(34)<br>*b*=5.91244(11)<br>*c*=14.22565(30)<br>*α*=90<br>*β*=112.8806(13)<br>*γ*=90	*a*=14.5491(18)<br>*b*=5.8917(8)<br>*c*=14.238(2)<br>*α*=90<br>*β*=112.832(3)<br>*γ*=90	*a*=6.8001(1)<br>*b*=8.8015(2)<br>*c*=17.4805(3)<br>*α*=90<br>*β*=90<br>*γ*=90	*a*= 6.6678(4)<br>*b*= 11.0680(6)<br>*c*= 14.4219(8)<br>*α*=90<br>*β*= 91.925(2)<br>*γ*=90
V [Å^3]	1280.903	2029.638	1104.916	1136.633	1124.838	1046.227	1063.724
R_1 *[I>2σ(I)]*	8.9	4.6	6.64	4.64	5.3	1.95	4.11

T [K]	room	room	room	room	173	100	100

Modifie proline-based ACEI

compound name	**perindoprilat hydrate**	**Perindoprilat DMSO solvate**	**perindopril erbumine**	**perindopril erbumine dihydrate**	**diketapiperazine of perindopril**	**diketapiperazine of perindopril**	**trandolapril**
CCDC ref. code	**FEFKEI** [184]	**BECWIR** [185]	**UZOVAH03** [189]	**IVEGIA** [189]	**BILNAN** [187]	**BILNAN01** [186, 187]	**IQISAE** [200]
formula	$C_{17}H_{28}N_2O_5$ $\cdot$ H_2O	$C_{17}H_{28}N_2O_5$ $\cdot$ 0.5 (C_2H_6OS)	$C_{19}H_{31}N_2O_5^-$ $\cdot$ $C_4H_{12}N^+$	$C_{19}H_{31}N_2O_5^-$ $\cdot$ $C_4H_{12}N^+$ $\cdot$ 2(H_2O)	$C_{19}H_{30}N_2O_4$	$C_{19}H_{30}N_2O_4$	$C_{24}H_{34}N_2O_5$
Crystal system	orthorhombic	orthorhombic	monoclinic	triclinic	orthorhombic	tetragonal	orthorhombic
Space group	$P2_12_12_1$	$P2_12_12_1$	$P2_1$	*P1*	$P2_12_12_1$	$P4_12_12_1$	$P2_12_12_1$
Z	4	8	2	2	8	8	4
Unit cell parameters [A, o]	*a*=8.1645(2)<br>*b*=10.0136(2)<br>*c*=23.2429(5)<br>*α*=90<br>*β*=90<br>*γ*=90	*a*=10.3504(7)<br>*b*=16.0908(11)<br>*c*=24.4828(16)<br>*α*=90<br>*β*=90<br>*γ*=90	*a*=12.400(3)<br>*b*=6.4313(17)<br>*c*=16.656(4)<br>*α*=90<br>*β*=97.013(3)<br>*γ*=90	*a*=6.5746(3)<br>*b*=12.1652(5)<br>*c*=16.9885(8)<br>*α*=97.153(4)<br>*β*=94.417(4)<br>*γ*=90.349(3)	*a*=9.2089(10)<br>*b*=17.9875(17)<br>*c*=23.697(2)<br>*α*=90<br>*β*=90<br>*γ*=90	*a*=9.2606(8)<br>*b*=9.2606(8)<br>*c*=44.847(4)<br>*α*=90<br>*β*=90<br>*γ*=90	*a*=19.7685(4)<br>*b*=15.0697(4)<br>*c*=7.67036(17)<br>*α*=90<br>*β*=90<br>*γ*=90
V [Å³]	1900.247	4077.518	1318.347	1344.015	3925.292	3846.021	2285.041
R_1 [I>2σ(I)]	2.69	2.68	7.59	3.73	5.67	3.91	1.84
T [K]	100	100	room	room	100	100	room

compound name	**ramipril**	**ramiprilat**	**ramiprilat methanol clathrate**	**spirapril hydrochloride monohydrate**	**sodium fosinopril**	**sodium hemi zofenopril**	
CCDC ref. code	**QOQWAU** [198]	**EDALEC** [197]	**FIFGEG** [199]	**RUWBAM** [201]	**TUHMOY** [196]	**TUHMUE** [196]	
formula	$C_{23}H_{32}N_2O_5$	$C_{23}H_{31}N_2O_5^-$ $\cdot$ $C_4H_{12}NO_3^+$	$C_{21}H_{28}N_2O_5$ $\cdot$ 4.7(CH_4O)	$C_{22}H_{31}N_2O_5S_2^+$ $\cdot$ Cl^- $\cdot$ H_2O	$C_{30}H_{45}NO_7P^-$ $\cdot$ Na^+	$C_{22}H_{22}NO_4S_2^-$ $\cdot$ $C_{22}H_{23}NO_4S_2$ $\cdot Na^+$ ($C_{44}H_{45}N_2Na_1O_8S_4$)	
Crystal system	orthorhombic	monoclinic	orthorhombic	orthorhombic	monoclinic	monoclinic	
Space group	$P2_12_12_1$	$P2_1$	$P2_12_12_1$	$P2_12_12_1$	$P2_1$	$P2_1$	
Z	4	2	4	4	2	2	
Unit cell parameters [A, o]	*a*=7.4845(11)<br>*b*=13.937(2)<br>*c*=22.012(3)<br>*α*=90<br>*β*=90<br>*γ*=90	*a*=24.3341(15)<br>*b*=6.4645(5)<br>*c*=9.5357(7)<br>*α*=90<br>*β*=96.9165(34)<br>*γ*=90	*a*=10.529(1)<br>*b*=12.147(2)<br>*c*=22.240(3)<br>*α*=90<br>*β*=90<br>*γ*=90	*a*=10.5075(15)<br>*b*=10.6251(15)<br>*c*=23.407(3)<br>*α*=90<br>*β*=90<br>*γ*=90	*a*=14.258(2)<br>*b*=5.951(1)<br>*c*=18.930(5)<br>*α*=90<br>*β*=93.01(2)<br>*γ*=90	*a*=5.461(1)<br>*b*=26.427(3)<br>*c*=15.239(1)<br>*α*=90<br>*β*=97.52(1)<br>*γ*=90	
V [Å³]	2296.104	1489.124	2844.402	2613.233	1603.982	2180.344	
R_1 [I>2σ(I)]	6.43	5.92	4.2	4.0	6.6	7.1	
T [K]	room	room	room	room	room	room	

Table S4. Conformations and puckering parameters of pyrrolidine ring in investigated structures [202].

	Group of proline type	*Puckering parameters*			
		Q [Å]	*φ [°]*		
Proline structures					
NELSEC	III	0.5223(13)	12.51(15)	*envelope*	*endo*
PROLIN03	III	0.410(3)	77.6(4)	*dist. envelope*	*endo*
QANRUT	III				*endo*
Proline-based ACEI structures					
EDALEC	II	0.204(13)	124(4)	*half-chair*	
FIFGEG	II	0.236(5)	70.4(11)	*envelope*	*endo*
IQISAE	I	0.422(11)	82.0(13)	*dist. half-chair*	
QOQWAU	I	0.275(5)	61.6(10)	*dist. half-chair*	
RUWBAM	I	0.416(4)	281.4(5)	*dist. envelope*	*exo*
Perindopril-derived structures					
IVEGIA	II	mol. 1: 0.384(3)<br>mol. 2: 0.392(3)	mol. 1: 291.5(4)<br>mol. 2: 293.0(4)	mol. 1: *dist. envelope*<br>mol. 2: *dist. envelope*	*exo*<br>*exo*
UZOVAH	II	0.411(8)	250.9(10)	*envelope*	*exo*
UZOVAH03	II	-	-	*envelope*	*exo*
BECWIR	I	mol. 1: 0.3776(14)<br>mol. 2: 0.3928(15)	mol. 1: 285.5(2)<br>mol. 2: 287.8(2)	mol. 1: *envelope*<br>mol. 2: *envelope*	*exo*<br>*exo*
FEFKEI	I	0.3973(14)	284.50(19)	*envelope*	*exo*
BILNAN		mol. 1: 0.381(4)<br>mol. 2: 0.413(4)	mol. 1: 284.9(5)<br>mol. 2: 284.7(5)	mol. 1: *envelope*<br>mol. 2: *envelope*	*exo*

BILNAN01		0.404(2)	275.7(3)	*half-chair*	
Other modified-proline-based ACEI structures					
CIYNIH	I	0.378(5)	77.2(7)	*envelope*	*endo*
DIVHOF01	II	0.357(16)	271(2)	*half-chair*	
GERXAE	II	0.129(9)	288(3)	*envelope*	*exo*
GERXEI	II	0.187(12)	309(4)	*half-chair*	
GERWUX01	II	0.320(5)	250.9(8)	*envelope*	*exo*
MCPRL01	I	0.3693(19)	291.6(3)	*dist. envelope*	*exo*
TUHMOY		0.362(11)	85.8(16)	*dist. half-chair*	
TUHMUE		mol. 1: 0.351(14)<br>mol. 2: 0.471(16)	mol. 1: 314(2)<br>mol. 2: 273.0(19)	mol. 1: *dist. half-chair*<br>mol. 2: *half-chair*	
YOZTIS	I	mol. 1: 0.385(5)<br>mol. 2: 0.401(6)	mol. 1: 83.7(7)<br>mol. 2: 70.1(7)	mol. 1: *dist. half-chair*<br>mol. 2: *envelope*	 *endo*

Table S5. Conformation of COOH/COO$^-$ group of proline ring in investigated structures.

CSD ref. code	*Type of tecton*	*conformation*
NELSEC	III	+ *syn*-periplanar
PROLIN03	III	- *syn*-periplanar
QANRUT	III	- *syn*-periplanar
IVEGIA	II	- *syn*-periplanar<br>- *syn*-periplanar
UZOVAH	II	- *syn*-periplanar
UZOVAH03	II	- *syn*-periplanar
BECWIR	I	- *syn*-periplanar<br>- *syn*-periplanar
FEFKEI	I	- *syn*-periplanar
BILNAN		- *syn*-periplanar
BILNAN01		
EDALEC	II	- *syn*-clinal<br>+ anti-clinal
FIFGEG	II	- *syn*-periplanar
IQISAE	I	- *syn*-periplanar
QOQWAU	I	- *syn*-periplanar
RUWBAM	I	- *syn*-clinal
CIYNIH	I	- *syn*-periplanar
DIVHOF01	II	- *syn*-clinal
GERXAE	II	- *syn*-periplanar
GERXEI	II	- *syn*-periplanar
GERWUX01	II	- *syn*-periplanar
MCPRL01	I	- *syn*-periplanar
TUHMOY		
TUHMUE		
YOZTIS	I	- *syn*-periplanar<br>- *syn*-periplanar

Table S6. Relevant atom charges, heteroatom-hydrogen bonds, d(H-X), relative DFT, ΔE_{DFT}, and Gibbs free energies at room temperature, ΔG_{298}, of stable structures of *L*-proline (**1**) obtained at M06/6-311++G(d,p)/CPCM(H_2O) level of theory.

**Structure**		**1a**	**1b**	**1c**
Heteroatom X charges				
O1		-0.719	-0.707	-0.658
O2		-0.672	-0.665	-0.705
N1		-0.746	-0.714	-0.715
Relevant hydrogen atoms				
H_A	location	N1	N1	N1
	d(H-X) [Å]	1.013	1.013	1.017
	charge	0.400	0.382	0.380
H_B	location	N1/O1	O1	O2
	d(H-X) [Å]	1.821/0.995	0.969	0.969
	charge	0.519	0.510	0.510
ΔE_{DFT} [kJ/mol]		0.0	24.2	19.1
ΔG_{298} [kJ/mol]		0.0	22.7	16.6
Rel. Gibbs free energy at 298 K (kJ/mol)		0.0	22.7	16.6

Table S7. Relevant atom charges, heteroatom-hydrogen bonds, d(H-X), relative DFT, ΔE_{DFT}, and Gibbs free energies at room temperature, ΔG_{298}, of stable structures of FEFKEI (**2**) obtained at M06/6-311++G(d,p)/CPCM(H_2O) level of theory.

Structure		2a	2b	2c	2d	2e	2f	2g
Heteroatom X charges								
O1		-0.693	-0.733	-0.709	-0.685	-0.710	-0.721	-0.558
O2		-0.812	-0.707	-0.710	-0.811	-0.697	-0.577	-0.820
O3		-0.780	-0.652	-0.607	-0.803	-0.654	-0.592	-0.827
O4		-0.642	-0.718	-0.819	-0.655	-0.649	-0.825	-0.730
O5		-0.700	-0.640	-0.813	-0.660	-0.702	-0.837	-0.714
N1		-0.514	-0.514	-0.500	-0.496	-0.529	-0.523	-0.443
N2		-0.610	-0.730	-0.602	-0.697	-0.696	-0.698	-0.682
Relevant hydrogen atoms								
H_A	location	N2	N2	N2	N2	N2	N2	N2
	d(H-X) [Å]	1.028	1.017	1.026	1.015	1.018	1.017	1.018
	charge	0.449	0.394	0.459	0.378	0.378	0.388	0.370
H_B	location	N2/O2	O2/N2	N2/O1	O1/O4	O2	O2	O4 [a)]
	d(H-X) [Å]	1.042/1.874	0.986/1.918	1.038/1.985	0.992/1.656	0.970	0.975	0.967
	charge	0.473	0.519	0.473	0.526	0.508	0.546	0.514
H_C	location	O5	O4/O1	O2	O5	O5	O3	O5[a)]
	d(H-X) [Å]	0.969	0.990/1.689	0.970	0.972	0.969	0.974	0.966
	charge	0.512	0.520	0.530	0.524	0.510	0.547	0.512
ΔE_{DFT} [kJ/mol]		13.8	0.0	62.1	106.3	15.7	190.9	178.9
ΔG_{298} [kJ/mol]		10.8	0.0	57.3	107.0	9.9	180.8	174.5
Rel. Gibbs free energy at 298 K (kJ/mol)		10.8	0.0	57.3	107.0	9.9	180.8	174.5

[a)]additional O1-C15 bond, d(O1-C15) = 1.534 Å

Table S8. Relevant atom charges, heteroatom-hydrogen bonds, d(H-X), relative DFT , ΔE_{DFT}, and Gibbs free energies at room temperature, ΔG_{298}, of stable structures of protonated IVEGIA (**3**) obtained at M06/6-311++G(d,p)/CPCM(H_2O) level of theory.

**Structure**		**3a**	**3b**	**3c**	**3d**	**3e**	**3f**
Heteroatom X charges							
O1		-0.710	-0.725	-0.695	-0.696	-0.705	-0.706
O2		-0.641	-0.654	-0.655	-0.655	-0.601	-0.488
O3		-0.556	-0.584	-0.585	-0.585	-0.484	-0.607
O4		-0.819	-0.719	-0.704	-0.649	-0.823	-0.823
O5		-0.831	-0.643	-0.652	-0.702	-0.836	-0.836
N1		-0.500	-0.517	-0.531	-0.529	-0.523	-0.523
N2		-0.600	-0.696	-0.697	-0.697	-0.689	-0.678
Relevant hydrogen atoms							
H_A	location	N2	N2	N2	N2	N2	N2
	d(H-X) [Å]	1.029	1.018	1.019	1.019	1.016	1.017
	charge	0.462	0.378	0.376	0.376	0.386	0.385
H_B	location	N2/O1	O1/O4	O4	O5	O2	O3
	d(H-X) [Å]	1.036/2.032	1.667/0.992	0.969	0.969	0.974	0.976
	charge	0.202	0.519	0.511	0.510	0.546	0.577
ΔE_{DFT} [kJ/mol]		42.0	-3.3	1.6	0.0	173.2	234.5
ΔG_{298} [kJ/mol]		41.4	1.9	5.5	0.0	172.9	227.8
Rel. Gibbs free energy at 298 K (kJ/mol)		41.4	1.9	5.5	0.0	172.9	227.8

Table S9. H-bond geometries of all investigated crystal structures.

**D-H···A**	*Symmetry code*	**D-H**	**H···A**	**D···A**	**D-H···A [°]**
Proline structures					
**PROLIN03**					
N1-H6···O1	*1+x, y, z*	0.94(4)	1.78(4)	2.707(3)	167(3)
*N1-H7···O1		0.98(3)	2.23(3)	2.656(3)	105(2)
N1-H7···O2	*1-x, -1/2+y, 3/2-z*	0.98(3)	1.92(4)	2.763(3)	143(3)

**NELSEC**					
N1-H1···O1	*1+x, y, z*	0.79	1.89	2.6557(16)	163
*N1-H2···O2		0.81	2.12	2.6389(17)	122
N1-H2···O2	*1/+x, ½-y, -z*	0.81	2.17	2.7819(19)	133
*C3-H5···O1		0.97	2.51	2.888(2)	103
C4-H6···O1	*-x, ½+y, ½-z*	0.97	2.44	3.1806(19)	133
**QANRUT**					
N1-H1···O1	*x, -1+y, z*	0.92(2)	1.80(2)	2.7131(14)	171.8(16)
NI-H2···O1	*-x, 1-y, -z*	0.906(19)	2.093(18)	2.8392(16)	138.9(17)
C2-H3···O1	*-x, -1/2+y, ½-z*	1.00	2.57	3.4857(17)	151

Proline-based ACEI structures

**CIYNIH**					
*N2-H3···O4		1.02(4)	2.29(4)	2.671(4)	100(2)
N2-H3···O6		1.02(4)	1.85(4)	2.832(5)	161(3)
N2-H4···O4	*½+x, ½-y, -z*	0.87(3)	1.99(3)	2.802(5)	154(4)
O2-H6···O5	*-x, -1/2+y, ½-z*	0.82(3)	1.81(4)	2.578(5)	156(4)
O6-H25···O3	*-1/2+x, ½-y, -z*	0.86(10)	2.10(8)	2.890(5)	154(5)
O6-H26···O7	*½+x, ½-y, -z*	0.82(4)	2.09(4)	2.808(5)	146(4)
O7-H28···O5		0.96(5)	1.85(5)	2.742(5)	153(4)
*C4-H2···O4		1.06(4)	2.42(4)	3.080(6)	119(3)
C17-H23···O2	*½-x, 1-y, -1/2+z*	1.08(4)	2.40(4)	3.404(7)	154(3)
**DIVHOF01**					
N1-H6···O7		0.99	1.78	2.753(15)	165
N1-H7···O5	*1-x, -1/2+y, 2-z*	0.98	2.42	3.137(16)	129
C20-H14···O3	*x, 1+y, x*	1.02	2.54	3.285(17)	129
C4-H15···O9	*-1+x, y, z*	0.99	2.46	3.369(16)	145
C5-H16···O1	*X, 1+y, z*	1.00	2.53	3.435(17)	166
C2-H20···O9	*-1+x, -1+y, z*	0.96	2.53	3.43(2)	156
C15-H23···O9	*-1+x, y, z*	1.01	2.55	3.445(17)	148
**GERWUX01**					
*N1-H1···O1		0.92	2.24	2.686(4)	109
N1-H1···O6	*1-x, ½+y, 2-z*	0.92	2.05	2.786(4)	136
N1-H2···O2	*x, 1+y, z*	0.92	1.81	2.726(4)	174
N2-H3···O4	*-x, ½+y, 1-z*	0.91	1.89	2.790(4)	172
N2-H4···O5	*-x, 3/2+y, 1-z*	0.91	1.85	2.721(4)	158

N2-H5···O4	*x, 1+y, z*	0.91	1.89	2.781(4)	167
O6-H32···O1	*1-x, ½+y, 2-z*	0.95(2)	1.83(2)	2.778(3)	179(5)
O6-H33···O1		0.947(19)	1.94(2)	2.857(3)	162(3)
O7-H34···O2		0.95(2)	1.89(2)	2.817(4)	165(3)
O7-H35···O7	*1-x, ½+y, 1-z*	0.95(2)	2.17(2)	3.102(5)	169(4)
C17-H25···O5	*x, 1+y, z*	0.99	2.41	3.366(5)	161
C17-H26···O6	*1-x, ½+y, 2-z*	0.99	2.45	3.432(5)	170
**GERXAE**					
O1-H11···O6	*1-x, ½+y, 2-z*	0.920(13)	1.81(2)	2.71(2)	166.6(9)
N1-H12···O2	*x, 1+y, z*	0.850(11)	2.075(12)	2.911(11)	167.8(8)
N2-H22···O4	*-x, ½+y, 1-z*	0.86(2)	2.048(15)	2.799(14)	145.3(10)
N2-H23···O4	*x, 1+y, z*	0.86(3)	2.08(3)	2.826(14)	145(2)
N2-H31···O5	*-x, 3/2+y, 1-z*	0.86(2)	1.9692)	2.732(15)	148(2)
O6-H32···N1	*1-x, -1/2+y, 2-z*	0.95(8)	1.94(7)	2.750(16)	142(10)
O6-H33···O1		0.95(3)	2.03(2)	2.905(16)	154(4)
*C12-H14···O3		0.980(14)	2.508(12)	2.888(12)	102.8(8)
C17-H24···O5	*x, 1+y, z*	0.980(16)	2.496(19)	3.421(12)	157.3(13)
C17-H25···O6	*1-x, ½+y, 2-z*	0.980(18)	2.51(3)	3.46(2)	165.3(14)
**GERXEI**					
O2-H11···O2	*-1-x, -1/2+y, -z*	0.992(15)	2.557(14)	3.472(14)	171.6(8)
O2-H11···N1	*-1-x, -1/2+y, -z*	0.992(15)	2.527(10)	3.011(10)	113.1(8)
N1-H12···O1	*x, 1+y, z*	0.850(16)	2.360(16)	3.127(15)	150.4(12)
N2-H22···O4	*-x, ½+y, 1-z*	0.98(2)	1.80(2)	2.632(16)	141(2)
N2-H23···O4	*x, 1+y, z*	0.98(3)	1.82(3)	2.737(15)	155(2)
N2-H31···O5	*-x, 3/2+y, 1-z*	0.86(3)	2.32(3)	3.121(16)	156(2)
C4-H3···O5	*-1+x, 1+y, z*	0.951(13)	2.599(14)	3.274(12)	128.2(10)
C8-H8···O1	*x, 1+y, z*	0.981(19)	2.52(2)	3.217(15)	127.9(11)
C13-H16···O3	*x, 1+y, z*	0.98(2)	2.47(2)	3.289(15)	141(2)
**MCPRL01**					
O1-H1···O3	*1-x, ½+y, ½-z*	0.80(3)	1.80(3)	2.5881(17)	169(3)
S1-H15···O2	*-1+x, y, z*	1.26(3)	2.40(3)	3.5165(12)	146.2(18)
C5-H7···O2	*-1/2+x, 3/2-y, 1-z*	0.98(2)	2.58(2)	3.408(2)	142.4(15)
C8-H11···O1	*1-x, -1/2+y, ½-z*	1.00(2)	2.53(2)	3.300(2)	133.8(19)
**TUHMOY**					
*C8-H10···O3		0.98	2.59	3.149(12)	116
*C18-H22···O7		0.96	2.30	2.657(13)	101

C19-H23···O4		0.99	2.46	3.142(13)	126
**TUHMUE**					
*O6-H45···O2		1.09	1.47	2.502(12)	156
*C8-H8···O4		0.95	2.37	2.88(2)	113
*C9-H10···O4	*-1+x, y, z*	0.95	2.57	3.31(3)	135
*C12-H13···S1		0.95	2.57	3.025(17)	110
*C26-H21···O8		0.94	2.50	3.428(19)	168
*C30-H25···O8		0.96	2.47	2.852(16)	104
*C31-H26···S3		0.91	2.87	3.227(16)	105
*C34-H29···S3		0.97	2.66	3.066(16)	105
**YOZTIS**					
O1-H1···O4	*2-x, -1/2+y, -z*	0.84	1.82	2.644(5)	167
O2-H2···O5	*-x, ½+y, 1-z*	0.84	1.85	2.677(5)	168
C2-H3···S2	*1+x, y, z*	1.00	2.81	3.709(4)	149
C5-H6···O3	*2-x, ½+y, -z*	0.99	2.44	3.307(7)	146
C6-H8···S1	*-1+x, y, z*	0.99	2.87	3.707(4)	143
*C7-H9···S1		1.00	2.84	3.389(4)	115
C11-H11···O3	*2-x, ½+y, -z*	0.98	2.52	3.405(7)	151
C16-H23···O3	*-1+x, y, z*	0.99	2.28	3.255(7)	170
C18-H27···O6	*1+x, -1+y, z*	0.99	2.57	3.179(6)	120

Perindopril structures

**UZOVAH01**					
N3-H32···O2	*1-x, ½+y, 1-z*	0.89	1.85	2.738(9)	175
N3-H33···O1	*-1+x, 1+y, z*	0.89	1.88	2.765(9)	179
N3-H34···O2	*-1+x, y, z*	0.89	1.91	2.788(10)	169
N3-H32···O2	*1-x, ½+y, 1-z*	0.89	1.85	2.738(9)	175
**UZOVAH03**					
N3-H32···O4	*-1+x, y, z*	0.89	1.91	2.80(2)	174
N3-H33···O5	*1-x, ½+y, 1-z*	0.89	1.89	2.782(13)	177
N3-H34···O5	*-1+x, 1+y, z*	0.89	1.90	2.79(3)	174
*C8-H6···O6		0.98	2.47	3.38(3)	154
*C10-H7···O2		0.98	2.59	3.28(3)	128
*C12-H10···O3		0.97	2.04	2.51(5)	108
*C14-H14···O3		0.96	2.33	3.14(7)	142
*C14-H14···O7		0.96	2.37	3.30(11)	163

*C19-H22···O6	*1-x, ½+y, 1-z*	0.96	1.09	1.67(4)	109
*C19-H23···O2	*1-x, ½+y, 1-z*	0.96	2.17	2.62(5)	107
*C12-H44···O3		1.16	1.86	2.51(5)	111
*C14-H47···O7		1.39	2.40	3.30(11)	118
*C19-H52···O2		1.28	2.20	3.46(4)	167
*C19-H52···O3		1.28	1.90	2.60(4)	108
*C19-H52···O7		1.28	1.79	2.83(8)	134
**IVEGIA**					
N2-H2···O12		0.86	2.53	3.022(4)	117
N5-H63···O5	*-1+x, y, z*	0.89	1.94	2.813(3)	168
N5-H64···O4		0.89	1.90	2.794(3)	166
N5-HH65···O10	*x, -1+y, 1+z*	0.89	1.94	2.811(3)	166
N6-H79···O9	*1+x, -1+y, z*	0.89	1.90	2.791(3)	179
N6-H80···O5	*x, y, -1+z*	0.89	1.90	2.784(3)	169
C12-H18···O12	*1+x, y, z*	0.97	2.53	3.500(5)	175
C13-H20···O13	*-1+x, y, z*	0.97	2.45	3.254(13)	140
C14-H23···O13		0.96	2.28	3.100(13)	144
C19-H29···O2	*1+x, y, z*	0.96	2.57	3.513(5)	166
C37-H59···O2		0.97	2.48	3.383(4)	155

Perindoprilat structures

**BECWIR**					
N2-H1···O11		0.905(19)	2.017(19)	2.8912(15)	161.9(16)
N2-H3···O8		0.898(17)	1.981(17)	2.7204(14)	138.5(15)
O5-H6···O2	*½+x, ½-y, -z*	0.93(2)	1.70(2)	2.5972(14)	162.4(19)
N4-H33···O3	*2-x, -1/2+y, ½-z*	0.856(19)	1.990(19)	2.8157(15)	161.8(16)
N4-H36···O11		0.887(19)	1.989(19)	2.8710(16)	172.2(17)
O10-H39···O7	*-1/2+x, ½-y, 1-z*	0.79(2)	1.79(2)	2.5773(15)	170(2)
C3-H7···O4	*-1/2+x, ½-y, -z*	1.00	2.56	3.3805(17)	140
*C10-H17···O3		1.00	2.42	3.0143(16)	117
C12-H19···O5	*-1/2+x, ½-y, -z*	0.99	2.54	3.3191(18)	135
C18-H29···O9	*½+x, ½-y, 1-z*	1.00	2.53	3.2571(17)	129
C21-H35···O5	*2-x, ½+y, ½-z*	0.99	2.57	3.5169(17)	161
*C27-H45···O8		1.00	2.50	3.0519(16)	115
C29-H47···O2	*2-x, -1/2+y, ½-z*	0.99	2.58	3.4845(18)	151
C35-H59···O3	*2-x, -1/2+y, ½-z*	0.98	2.46	3.420(2)	168

C36-H61···O7	*2-x, -1/2+y, ½-z*	0.98	2.29	3.218(2)	159
**FEFKEI**					
N2-H2···O2	*½+x, 3/2-y, 2-z*	0.884(19)	1.954(17)	2.7063(15)	142.1(15)
N2-H3···O6		0.959(16)	1.850(16)	2.7923(16)	167.0(15)
O5-H11···O3	*2-x, ½+y, 3/2-z*	0.890(19)	1.73(2)	2.5929(14)	163.7(19)
O6-H29···O3	*1+x, y, z*	0.90(2)	1.89(2)	2.7725(15)	167(2)
O6-H30···O1		0.81(2)	2.29(2)	2.8307(15)	124(2)
C1-H1···O4	*2-x, -1/2+y, 3/2-z*	1.00	2.45	3.2777(16)	140
C4-H7···O1	*-1+x, y, z*	0.99	2.56	3.3926(17)	142
*C10-H17···O2		1.00	2.33	2.9600(16)	120
C11-H18···O5	*2-x, -1/2+y, 3/2-z*	1.00	2.50	3.1808(16)	125

DKP perindopril structures

**BILNAN**					
C10-H14···O8		1.00	2.22	3.184(4)	161
C11-H15···O6	*1-x, ½+y, 3/2-z*	1.00	2.45	3.297(4)	142
*C12-H17···O4		0.99	2.51	3.062(4)	115
C29-H44···O4	*1+x, y, z*	1.00	2.36	3.341(4)	165
*C31-H46···O8		0.99	2.53	3.082(4)	115
C38-H59···O5	*-1+x, y, z*	0.98	2.54	3.408(5)	148
**BILNAN01**					
C10-H12···O4	*3/2-x, -1/2+y, ¼-z*	0.98	2.30	3.218(2)	155
*C18-H24···O2		0.97	2.39	2.725(8)	100
C19-H27···O1	*x, 1+y, z*	0.96	2.40	3.172(5)	138
*C12-H29···O4		0.97	2.52	3.050(3)	115
C1-H30···O4	*3/2-x, -1/2+y, ¼-z*	0.98	2.58	3.403(2)	141

Other modified-proline-based ACEI structures

**EDALEC**					
*N1-H31···O3		0.83	2.24	2.797(18)	124
O6-H38···O8	*x, -1+y, z*	0.84	2.42	2.723(14)	102
*O7-H39···O8		0.84	1.94	2.620(14)	138
O8-H40···O6	*x, 1+y, z*	0.84	2.07	2.723(14)	134
*O8-H40···N3		0.84	2.18	2.480(10)	101
*N3-H42···O6		0.84	2.42	2.829(12)	111

C3-H3···O4	*1-x, -1/2+y, -z*	0.92	2.41	3.179(14)	141
*C8-H8···O1		0.92	2.21	2.760(15)	118
C11-H12···N1	*x, -1+y, z*	0.92	2.22	3.043(19)	149
C13-H16···O1	*x, 1+y, z*	0.92	2.56	3.060(16)	115
C14-H18···O1	*x, 1+y, z*	0.92	2.47	2.846(13)	105
C18-H23···O7	*x, -1+y, -1+z*	0.92	2.37	2.976(12)	124
C18-H23···O8	*x, -1+y, -1+z*	0.92	2.21	2.889(13)	130
C18-H24···O7	*2-x, -1/2+y, 1-z*	0.92	2.31	3.217(11)	171
C19-H25···O7	*x, y, -1+z*	0.92	2.44	3.277(15)	152
*C19-H26···O5		0.92	2.34	2.898(16)	119
**FIFGEG**					
*N2-H24···O1		0.74(2)	2.58(3)	2.803(3)	100(2)
N2-H24···O6		0.74(2)	2.10(2)	2.783(4)	155(3)
C11-H13···O4	*½+x, ½-y, -z*	0.960(6)	2.566(5)	3.442(5)	151.8(4)
**IQISAE**					
*O3-H30···N1		0.95	2.27	2.745(14)	110
N2-H31···O4	*½-x, 1-y, -1/2+z*	1.03	1.59	2.596(16)	167
C4-H5···O5	*-1/2+x, 3/2-y, 1-z*	1.07	2.59	3.383(18)	131
*C6-H9···O1		1.05	2.53	3.217(18)	122
*C6-H10···O5		1.09	2.50	3.375(19)	137
C9-H12···O1	*x, y, 1+z*	1.03	2.55	3.428(17)	144
*C19-H27···O5		1.09	2.30	2.939(16)	115
C24-H33···O4	*½-x, 1-y, -1/2+z*	1.09	2.59	3.337(18)	125
**QOQWAU**					
O1-H1···N2	*1/2-+x, ½-y, -z*	1.04(5)	1.58(5)	2.601(6)	168(5)
*N2-H2···O3		0.78(3)	2.43(3)	2.829(5)	114(3)
C3-H6···O5	*3/2-x, -y, 1/1+z*	0.98	2.45	3.388(6)	161
*C14-H20···O5		0.97	2.35	2.690(6)	100
**RUWBAM**					
N1-H13···Cl1		0.96(5)	2.42(4)	3.212(3)	140(4)
N1-H14···O4	*2-x, ½+y, ½-z*	0.81(4)	2.11(4)	2.866(4)	155(4)
O5-H15···Cl1	*2-x, -1/2+y, ½-z*	0.73(5)	2.26(5)	2.990(3)	175(6)
O6-H32···S1	*1-x, -1/2+y, ½-z*	0.98(5)	2.51(5)	3.473(5)	169(4)
O6-H33···Cl1	*-1+x, y, z*	1.05(12)	2.32(12)	3.327(5)	161(10)
*C3-H3···O1		1.01(4)	2.60(3)	3.043(4)	107(3)
*C2-H11···O3		0.92(4)	2.46(4)	2.879(5)	108(3)

C9-H19···Cl1	*2-x, ½+y, ½-z*	0.93(4)	2.77(4)	3.674(4)	167(3)
C12-H24···O2	*2-x, -1/2+y, ½-z*	1.05(4)	2.48(4)	3.246(5)	130(3)
C12-H26···O1	*2-x, ½+y, ½-z*	1.00(5)	2.51(5)	3.497(5)	170(4)

Table S10. Geometrical parameters (Å and º) for the π-stacking moieties involved in the π···π interactions for studied compounds.

CgI-CgJ[a,b]	*symmetry*	*Cg···Cg*[c]	*Cg(I)-perp*[d]	*Cg(J)-perp*[e]	*α*[f]	*β*[g]	*γ*[f]
Proline-based ACEI structures							
CIYNIH							
Cg2···Cg2	*-x, -1/2+y, -1/2-z*	5.535(3)	3.561(2)	-4.003(2)	87.3(3)	43.7	49.9
DIVHOF01							
Cg2···Cg2	*1-x, -1/2+y, 1-z*	4.800(13)	1.667(8)	4.473(8)	50	21.3	69.7
GERWUX01							
Cg2···Cg2	*2-x, ½+y, 2-z*	5.196(2)	1.8039(16)	4.2058(16)	48.12(18)	36.0	69.7
GERXAE							
Cg2···Cg1	*1-x, ½+y, 2-z*	5.113(4)	0.194(3)	4.639(3)	63.6(4)	24.9	87.8
Cg2···Cg2	*2-x, ½+y, 2-z*	5.420(3)	1.745(2)	4.126(2)	47.5(2)	40.4	71.2
TUHMOY							
Cg2···Cg2	*2-x, -1/2+y, -z*	4.836(8)	1.870(5)	4.679(5)	56.3(6)	14.6	67.3
TUHMUE							
Cg3···Cg3	*-1+x, y, z*	5.461(10)	3.033(7)	-3.033(7)	0.0(8)	56.3	56.3
Cg5···Cg3	*-x, -1/2+y, -z*	5.979(10)	-1.345(6)	-3.628(7)	73.9(8)	52.7	77.0
Cg5···Cg5	*-1+x, y, z*	5.461(9)	3.015(7)	-3.015(7)	0.0(8)	56.5	56.5
Cg5···Cg6	*-1+x, y, z*	5.080(10)	1.608(7)	-4.866(7)	75.4(8)	16.7	71.6
Cg6···Cg6	*1+x, y, z*	5.461(10)	2.924(7)	-2.924(7)	0.0(8)	57.6	57.6
Perindopril –derived compounds							
UZOVAH03							
Cg18···Cg18	*1-x, ½+y, 1-z*	4.88(4)	-1.96(4)	4.08(4)	41	33.4	66.4
Other modified-proline-based ACEI structures							
EDALEC							
Cg3···Cg3	*1-x, -1/2+y, -z*	4.888(7)	2.388(4)	-3.413(4)	52.4(5)	45.7	60.8
IQISAE							
Cg2···Cg2	*½-x, 2-y, -1/2+z*	5.156(7)	1.994(6)	4.287(6)	34.8(7)	33.7	67.2
RUWBAM							
Cg4···Cg4	*½+x, 3/2-y, 1-z*	5.972(3)	0.2921(19)	4.2006(19)	43.7(2)	45.3	87.2

Table S11. Geometrical parameters (in Å and in º) for the other π-stacking moieties for studied compounds.

Y(X)-X(H)···Cg	*symmetry*	*X(H)···Cg*	*Y(X)-X(H)···Cg*	*Y(X)···Cg*
Proline-based structures				
CIYNIH				
C8-H9···Cg2	*-1/2-x, 1-y, ½+z*	2.77(4)	139(3)	3.614(6)
C9-H12···Cg2		2.91(4)	130(3)	3.610(6)
DIVHOF01				
C10-H2···Cg2	*1-x, ½+y, 1-z*	2.71	144	3.56(2)
C2-H21···Cg2	*X, -1+y, z*	2.88	167	3.91(2)
GERWUX01				
C15-H24···Cg2	*1-x, ½+y, 1-z*	2.77	173	3.753(4)
GERXAE				
C15-H20···Cg2	*1-x, ½+y, 1-z*	2.727(19)	167.9(15)	3.691(12)
GERXEI				
C15-H20···Cg2	*-1-x, ½+y, 1-z*	2.72(2)	152(2)	3.611(15)
TUHMOY				
C14-H18···Cg2	*2-x, ½+y, -z*	2.84	137	3.604(13)
TUHMUE				
C32-O8···Cg6	*x, y, z*	3.665(13)	150.9(10)	4.766(18)
Other modified-proline-based ACEI structures				
EDALEC				
C23-O5···Cg2	*x, y, z*	2.825(13)	99.8(11)	3.280(16)
FIFGEG				
C5-H7···Cg3	*1-x, -1/2+y, ½-z*	2.875(5)	167.9(5)	3.817(5)
RUWBAM				
C11-H23···Cg4	*3/2-x, 2-y, -1/2+z*	2.68(5)	167(4)	3.682(5)
C22-H29···Cg4	*X, 1+y, z*	2.88	142	3.688(9)

Table S12. HS analysis (percent contribution of various types of the intermolecular interactions) of ACEI and their derivatives available in the CSD (above 0.5 %).

	H···H	O···H/ H···O	N···H /H···N	C···H /H···C	C···C	C···O /O···C	O···O	N···O /O···N	O···S /S···O	S···H /H···S [S···S]	Cl···H /H···Cl	O···Na /Na···O	H···Na /Na···H
Proline structures													
PROLIN03	52.2	45.9		1.9									
NELSEC	31.4	43.4		2.9					3.4	18.2 [0.7]	0.7		
QANRUT	51.9	45.6		2.5									
Proline-based ACEI structures													
CIYNIH	46.5	39.1		12.9									
DIVHOF01	31.9	38.9		20	2	1.2	2.5						
GERXAE	59.2	29.1		10.3									
GERXEI	55.2	30.1	0.7	10.7									
GERWUX	59	30.3	0.5	10.2									
GERWUX01	57.6	32		10.4									
MCPRL01	54.6	33.3		0.6						10.9			
TUHMOY	69	14.8		7.5								6.5	1.5
TUHMUE	49.8	12.6		19.9		0.9				10		4	0.6
YOZTIS	54.4	34.5		1.4					0.7	8.7			
Perindoprilat structures													
FEFKEI	62.9	36.1		0.9			0.2						
BECWIR mol. 1	60.8	36		0.9			0.3		0.2	1.4			
BECWIR mol. 2	64.2	34.2		0.8			0.4						
Perindopril structures													
IVEGIAm.1	70.5	28.3		0.4			0.8						
IVEGIAm.2	73.8	23.2		0.4			2.6	0.1					
UZOWAH	74.1	24.4	0.3				0.4						
UZOWAH1	76.8	22.7		0.3									
UZOWAH3	76.6	22.6	0.1	0.6		0.1							
UZOVIPm1	70.2	28.7		0.4			0.7						
UZOVIPm2	73.8	23.2		0.4			2.5	0.1					
DKP perindopril structures													
BILNAN mol. 1	77.3	20	0.8	0.7	0.1	0.2	0.1						
BILNAN mol. 2	74.5	22.7	0.9	0.8	0.1	0.2	0.1						
BILNAN01	77.9	20.7	0.7	0.7									
Other modieif-proline-based ACEI structures													
EDALEC	58.5	27.2		4.3		1.6	0.7						
FIFGEG	37.5	34.1		16.7		4.1	5.2	0.6					
IQISAE	68.2	21.3	0.7	8.4		0.7							
IQISAE01	68.2	21.2	0.8	8.4		0.7							
QOQWAU	68.1	19	1.8	9.7		0.6	0.7						
RUWBAM	58.1	17.5		8.5		0.5	1.4		1	7.9	5		

Table S13. HS parameters for analyzed structures.

	Volume V [Å^3]	*Globularity G*	*Surface Area A [Å^2] (A/V [Å^{-1}])]*	*Asphericity Ω*
Proline structures				
PROLIN03	131.38	0.883	141.47 (1.07)	0.031
NELSEC	136.13	0.887	144.26 (1.06)	0.03

QANRUT	131.90	0.886	141.39 (1.07)	0.028
Proline-based ACEI structures				
CIYNIH	424.13	0.719	379.55 (0.89)	0.337
DIVHOF01	631.7	0.702	506.89 (0.80)	0.100
GERWUX01	500.59	0.693	439.80 (0.88)	0.185
GERXAE	535.75	0.700	455.64 (0.85)	0.167
GERXEI	544.81	0.711	453.84(0.83)	0.181
MCPRPL01	255.96	0.797	244.73 (0.96)	0.135
TUHMOY	789.2	0.649	636.65 (0.81)	0.231
TUHMUE	1075.52	0.604	840.94 (0.78)	0.172
Perindoprilat structures				
BECWIR mol. 1	453.29	0.749	381.19 (0.84)	0.123
BECWIR mol. 2	444.07	0.733	384.22 (0.86)	0.135
FEFKEI	441.21	0.738	379.54 (0.86)	0.140
Perindopril structures				
IVEGIA	485.94	0.767	389.82 (0.80)	0.046
UZOVAH	484.07	0.780	382.09 (0.79)	0.057
UZOVAH03	514.95	0.728	426.52 (0.83)	0.115
DKP perindopril structures				
BILNAN	487.5	0.788	380.13 (0.78)	0.084
BILNAN02	480.08	0.788	376.22 (0.78)	0.093
Other modified-proline-based ACEI structures				
QOQWAU	566.30	0.737	448.93 (0.79)	0.084
EDALEC	575.15	0.726	460.95 (0.80)	0.178
FIFGEG	526.06	0.716	440.01 (0.83)	0.280
IQISAE	562.91	0.719	458.42 (0.81)	0.083
RUWBAM	585.61	0.658	514.14 (0.88)	0.147

Table S14. HS interaction surfaces, random contatcs and enrichment ratios for all analyzed structures. Values in italics are derived from the HS analysis by CrystalExplorer. ER were calculated for contacts larger than 0.9 %.

Proline structures:

PROLIN03

	H	O	C
H	*52.2*	*HS contacts [%]*	
O	*45.9*		
C	*1.9*		
Surface (%)	76.1	22.95	0.95
H	57.91	Random contatcs [%]	
O	34.92		

C	1.45		
H	**0.901**	**Enrichment ratio**	
O	**1.314**		
C	**1.31**		

QANRUT

	H	O	C
H	*51.9*		
O	*45.6*		
C	*2.5*		
Surface (%)	75.95	22.8	1.25
H	57.68		
O	34.6		
C	1.89		
H	**0.9**		
O	**1.32**		
C	**1.32**		

NELSEC

	H	O	C	S
H	*31.4*			
O	*43.4*			
C	*2.9*			
S	*18.2*	*3.4*		
Surface (%)	63.65	23.4	1.45	11.5
H	40.51			
O	29.78			
C	1.85			
S	14.63	5.38		
H	**0.78**			
O	**1.46**			
C	**1.57**			
S	**1.24**	**0.63**		

Perindopril-derived structures:

FEFKEI

	H	O	C
H	*62.9*		
O	*36.1*		
C	*0.9*		
Surface (%)	81.4	18.05	0.45
H	66.25		
O	29.38		
C	0.73		
H	**0.95**		
O	**1.23**		
C	**1.23**		

BECWIR

	~~H~~	~~O~~	~~C~~	~~S~~
H	*60.8*			
O	*36*			
C	*0.9*			
S	*1.4*			
Surface (%)	61.95	18	0.45	0.7
H	38.37			
O	22.3			
C	0.56			
S	0.87			
H	**1.58**			
O	**1.61**			
C	**1.61**			
S	**1.61**			

BILNAN

	H	O	N
H	*74.5*		
O	*22.7*		
N	*0.9*		
Surface (%)	86.3	11.35	0.45
H	74.47		

O	19.6		
N	0.78		
H	**1.0**		
O	**1.16**		
N	**1.15**		

BILNAN01

	H	O
H	*77.9*	
O	*20.7*	
Surface (%)	88.25	10.35
H	77.88	
O	18.26	
H	**1.0**	
O	**1.1**	

IVEGIA

	H	O
H	*73.8*	
O	*23.2*	*2.6*
Surface (%)	85.4	14.2
H	73	
O	24.25	2
H	**1**	
O	**0.96**	1.3

UZOWAH

	H	O
H	*74.1*	
O	*24.4*	
Surface (%)	86.3	12.2
H	74.4	
O	21.06	
H	**0.96**	
O	**1.16**	

UZOWAH03

	H	O
H	*76.6*	
O	*22.6*	
Surface (%)	87.9	11.3
H	77.26	
O	19.86	
H	**1.0**	
O	**1.14**	

Other proline-based ACEI structures:

CIYNIH

	H	O	C
H	*46.5*		
O	*39.1*		
C	*12.9*		
Surface (%)	72.5	19.55	6.45
H	52.56		
O	28.34		
C	9.35		
H	**0.88**		
O	**1.38**		
C	**1.38**		

DIVHOF01

	H	O	C
H	*31.9*		
O	*38.9*	*2.5*	
C	*20*	*1.2*	*2*
Surface (%)	61.35	22.55	12.6
H	37.64		
O	28.48	5.08	5.67
C	15.91		
H	**0.85**		

O	**1.37**	**2.03**	
C	**1.25**	**0.21**	**1.25**

EDALEC

	H	O	C
H	*58.5*		
O	*27.2*		
C	*4.3*	*1.6*	
Surface (%)	74.25	14.4	2.95
H	55.1		
O	21.4		
C	4.39	0.85	
H	**1.06**		
O	**1.27**		
C	**0.98**	**1.88**	

FIFGEG

	H	O	C
H	*37.5*		
O	*34.1*		
C	*16.7*		
Surface (%)	62.9	24.3	10.4
H	39.56		
O	30.56	5.9	5.05
C	13.1		
H	**0.95**		
O	**1.1**	**0.88**	**0.81**
C	**1.27**		

GERXAE

	H	O	C
H	*59.2*		
O	*29.1*		
C	*10.3*		
Surface (%)	78.9	14.55	5.15
H	62.25		
O	22.95		
C	8.12		
H	**0.95**		
O	**1.27**		
C	**1.27**		

GERXEI

	H	O	C
H	*55.2*		
O	*30.1*		
C	*10.7*		
Surface (%)	75.6	15.05	5.35
H	57.15		
O	22.75		
C	8.08		
H	**1.03**		
O	**1.28**		
C	**1.27**		

GERWUX01

	H	O	C
H	*57.6*		
O	*32*		
C	*10.4*		
Surface (%)	73.6	16	5.2
H	54.17		
O	23.55		
C	7.65		
H	**1.06**		
O	**1.36**		
C	**1.36**		

IQISAE

	H	O	C
H	*68.2*		
O	*21.3*		
C	*8.4*		
Surface (%)	83.05	10.65	4.2

H	68.97		
O	17.68		
C	6.97		
H	**0.98**		
O	**1.2**		
C	**1.2**		

QOQWAU

	H	O	N	C
H	*68.1*			
O	*19*			
N	*1.8*			
C	*9.7*			
Surface (%)	83.35	9.5	0.9	4.85
H	69.47			
O	15.84			
N	1.5			
C	8.08			
H	**0.71**			
O	**1.2**			
N	**1.2**			
C	**1.2**			

RUWBAM

	H	O	C	S	Cl
H	*58.1*				
O	*17.5*		*1.4*		
C	*8.5*				
S	*7.9*	*1*			
Cl	*5*				
Surface (%)	77.55	9.45	4.95	3.95	2.5
H	60.14				
O	14.65				
C	7.67				
S	6.12	0.75			
Cl	3.87				
H	**0.96**				
O	**1.19**				
C	**1.10**	**1.5**			
S	**1.28**	**1.33**			
Cl	**1.29**				

TUHMOY

	H	O	C	Na
H	*69*			
O	*14.8*			
C	*7.5*			
Na	*1.5*	*6.5*		
Surface (%)	80.9	10.65	3.75	4
H	65.45			
O	17.23			
C	6.06			
Na	6.47	0.852		
H	**1.05**			
O	**0.86**			
C	**1.24**			
Na	**0.23**	**7.62**		

TUHMUE

	H	O	C	S	Na
H	*49.8*				
O	*12.6*				
C	*19.9*	*0.9*			
S	*10*				
Na		*4*			
Surface (%)	71.05	8.75	10.4	5	2
H	50.48				
O	12.43				
C	14.77	1.82			
S	7.1				
Na		0.35			
H	**1**				
O	**1.01**				
C	**1.35**				
S	**1.41**	**0.5**			

Na		**11.43**			

MCPRL01

	H	O	S
H	*54.6*		
O	*33.3*		
S	*10.9*		
Surface (%)	76.7	16.65	5.45
H	57.91		
O	25.54		
S	8.36		
H	**0.94**		
O	**1.30**		
S	**0.77**		

YOZTIS

	H	O	C	S
H	*54.4*			
O	*34.5*			
C	*1.4*			
S	*8.7*			
Surface (%)	76.7	17.25	0.7	4.35
H	58.82			
O	26.46			
C	1.07			
S	6.67			
H	**0.93**			
O	**1.3**			
C	**1.3**			
S	**1.3**			

Table S15. Supramolecular synthons resulting from three types of proline-based tectons observed in ACEI structures.

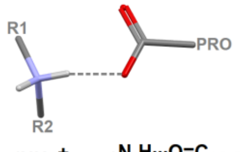 $(NH_2^+{}_{PRO})^{N-H\cdots O=C}(COO^-{}_{PRO})$	PROLIN03 (III):<br>**$C(5)$**, $C^2_2(6)$, **$C^2_2(9)$**, **$C^2_2(10)$**, **$C^3_4(14)$**, $C^4_4(16)$, **$R^4_4(16)$**<br>QANRUT (III):<br>**$C(5)$**, **$C^2_2(9)$**, **$C^2_2(10)$**, **$C^2_2(11)$**, **$C^3_4(14)$**, **$C^4_4(18)$**, $R^2_4(8)$, $R^3_4(14)$, **$R^4_4(16)$**, $R^4_4(18)$, $R^4_4(20)$, $R^4_5(19)$, $R^4_6(18)$<br>NELSEC (III):<br>**$C(5)$**, $C^2_2(8)$, **$C^2_2(9)$**, **$C^2_2(10)$**, **$C^2_2(11)$**, $C^3_4(16)$, $C^4_4(17)$, **$C^4_4(18)$**, $R^3_3(11)$, $R^3_4(16)$, **$R^4_4(16)$**, $R^4_4(17)$
$(NH_2^+)^{N-H\cdots O=C}(COO^-{}_{PRO})$	DIVHOF01 (II):<br>$C(8)$, $C^2_2(11)$, $C^2_2(13)$, $C^2_2(14)$, $C^2_2(15)$, $R^3_3(19)$, $D^3_3(15)$, $D^3_3(16)$, $D^3_3(17)$, $D^3_3(19)$
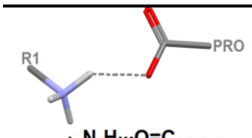 $(NH_3^+)^{N-H\cdots O=C}(COO^-{}_{PRO})$	IVEGIA (II):<br>**$D(2)$**, $D^2_2(5)$, $D^2_2(12)$, $D^2_2(14)$, $D^3_3(20)$<br>UZOVAH03 (II): **$D(2)$**<br>GERXAE (II): **$C(12)$**, $C^1_2(4)$, **$C^2_2(10)$**, **$C^2_2(12)$**, **$C^2_2(18)$**, **$C^2_2(19)$**, **$C^2_2(20)$**, **$R^2_2(10)$**, **$R^2_2(12)$**, **$R^2_2(19)$**, $R^2_3(16)$, $D^3_3(17)$, **$D^3_3(19)$**<br>GERXEI (II): **$C(12)$**, **$C^2_2(12)$**, $C^2_2(17)$, **$C^2_2(18)$**, **$C^2_2(19)$**, **$R^2_2(12)$**, **$R^2_2(19)$**<br>GERWUX01 (II): **$C(12)$**, **$C^2_2(10)$**, **$C^2_2(18)$**, **$C^2_2(19)$**, **$C^2_2(20)$**, **$R^2_2(10)$**, **$R^2_2(19)$**, **$D^3_3(19)$**
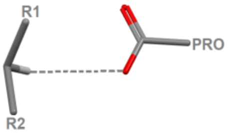 $(C_{cycl}\ PRO)^{C-H\cdots O=C}(COO^-{}_{PRO})$	PROLIN03 (III): **$C(4)$**, **$C^2_2(9)$**, **$C^3_4(14)$**<br>NELSEC (III): **$C(6)$**, $C^2_2(7)$, **$C^2_2(10)$**, **$C^2_2(11)$**, $C^3_4(16)$, $C^4_4(17)$, **$C^4_4(18)$**, $R^3_4(16)$<br>QANRUT (III):**$C(4)$**, **$C(5)$**, **$C(6)$**, **$C^2_2(9)$**, **$C^2_2(10)$**, **$C^2_2(11)$**, **$C^3_4(14)$**, $C^3_4(17)$, **$C^4_4(18)$**, $R^3_4(14)$, $R^3_4(17)$, $R^4_4(16)$, $R^4_4(18)$, $R^4_4(20)$, $R^4_5(19)$, $R^4_6(18)$<br>FIFGEG (II): **$C(5)$**, **$C^2_2(14)$**, $D^3_3(18)$<br>GERXAE (II): **$C(6)$**, $C(9)$, **$C^2_2(14)$**, **$C^2_2(16)$**, **$C^2_2(17)$**, **$C^2_2(18)$**, **$C^2_2(19)$**, **$R^2_2(14)$**, **$R^2_2(16)$**, **$R^2_2(17)$**, **$R^2_2(19)$**, **$D^3_3(11)$**, $D^3_3(15)$<br>GERXEI (II): $C^1_2(8)$, $C^1_2(10)$, $C^1_2(11)$, $C^2_2(13)$, **$C^2_2(14)$**, $C^2_2(15)$, **$C^2_2(16)$**, **$C^2_2(17)$**, **$C^2_2(18)$**, **$C^2_2(19)$**, $C^2_2(20)$, $R^2_2(13)$, **$R^2_2(14)$**, **$R^2_2(17)$**, **$R^2_2(19)$**, $R^2_2(19)$, $R^2_2(20)$, $R^2_3(17)$, $R^2_3(19)$, $R^2_3(20)$<br>GERWUX01 (II):<br>**$C(6)$**, **$C^2_2(16)$**, **$C^2_2(17)$**, **$C^2_2(18)$**, **$R^2_2(16)$**, **$R^2_2(17)$**, **$D^3_3(11)$**, $D^3_3(17)$
$(C_{cycl})^{C-H\cdots O=C}(COO^-{}_{PRO})$	EDALEC (II): **$C(15)$**, **$C^2_2(19)$**<br>GERXEI (II): **$C(15)$**, $C^2_2(16)$, $C^2_2(17)$, $C^2_2(18)$,**$C^2_2(19)$**, $C^2_2(20)$
$(C)^{C-H\cdots O=C(COO^-{}_{PRO})}$	FIFGEG (II): $C(9)$, $C^2_2(14)$, **$C^2_2(17)$**, $D^3_3(14)$, $D^3_3(16)$<br>GERWUX01 (II): $C(10)$, $C^2_2(16)$, **$C^2_2(17)$**, $R^2_3(16)$, $R^3_3(20)$, $D^3_3(17)$, $D^3_3(19)$
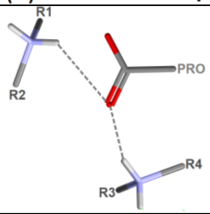	PROLIN03 (III): $C^1_2(4)$, **$C^3_4(14)$**, **$R^3_4(14)$**<br>QANRUT (III): **$C^3_4(14)$**, **$R^3_4(14)$**, $R^4_6(18)$

[(NH$_2^+$ $_{PRO}$)N-H]$_2$··· O=C(COO$^-$ $_{PRO}$)	
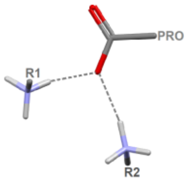<br>[(NH$_3^+$)N-H]$_2$···O=C(COO$^-$ $_{PRO}$)	IVEGIA (II): C$^2_2$(6), D$^1_2$(3)<br>UZOVAH03 (II): **C$^1_2$(4), C$^2_2$(6)**<br>GERXEI (II): **C$^1_2$(4), R$^2_3$(16)**<br>GERWUX01 (II): **C$^1_2$(4), R$^2_3$(16)**
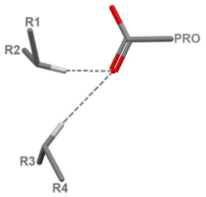<br>[(C$_{cycl}$ PRO)C-H]$_2$···O=C(COO$^-$ $_{PRO}$)	QANRUT (III): C$^1_2$(6), C$^3_4$(17), R$^3_4$(17)
(Ccycl PRO)C-H, (C$_{cycl}$)C-H ···> O=C(COO$^-$ $_{PRO}$)	GERXEI (II): C$^1_2$(15)
(Ccycl PRO)C-H, (C)C-H ···> O=C(COO$^-$ $_{PRO}$)	FIFGEG (II): R$^1_2$(10)
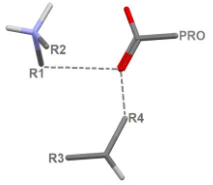<br>(NH$_2^+$ $_{PRO}$)N-H, (C$_{cycl}$)C-H ···> O=C(COO$^-$ $_{PRO}$)	PROLIN03 (III): C$^1_2$(5), R$^1_2$(5), R$^3_4$(14)<br>QANRUT (III): C$^1_2$(5), C$^2_2$(7), R$^2_3$(9)<br>NELSEC (III): C$^1_2$(5), C$^3_4$(16), R$^3_4$(16)
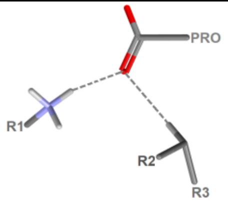<br>(NH$_3^+$)N-H, (C$_{cycl}$ PRO)C-H ···> O=C(COO$^-$ $_{PRO}$)	GERWUX01 (II): C$^1_2$(12)
(NH$_3^+$)N-H, (C$_{cycl}$)C-H ···> O=C(COO$^-$ $_{PRO}$)	GERXEI (II): C$^1_2$(17)
(NH$_3^+$)N-H, (C)C-H ···> O=C(COO$^-$ $_{PRO}$)	GERWUX01 (II): R$^1_2$(6)
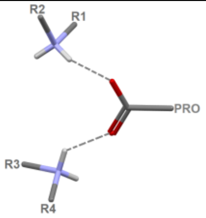<br>[(NH$_2^+$ $_{cycl}$)N-H]$_2$···O=C(COO$^-$ $_{PRO}$)	PROLIN03 (III): R$^2_1$(4)<br>NELSEC (III): C$^2_2$(6), R$^3_3$(11), R$^4_4$(16)
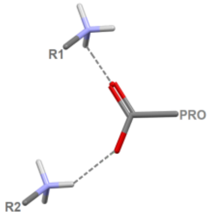<br>[(NH$_3^+$)N-H]$_2$···O=C(COO$^-$ $_{PRO}$)	GERXAE, GERXEI, GERWUX01 (II): C$^2_2$(6)
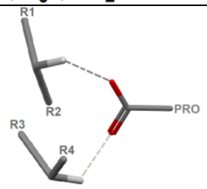<br>[(Ccycl PRO)C-H]$_2$···O=C(COO$^-$ $_{PRO}$)	QANRUT (III): R$^3_3$(11), R$^4_4$(16)
(Ccycl PRO)C-H, (-C)C-H ···> O=C(COO$^-$ $_{PRO}$)	GERWUX01 (II): C$^2_2$(12)

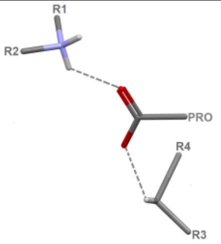<br>(NH$_2^+$cycl PRO)N-H<br>(C$_{cycl}$ PRO)C-H ⋯> O=C(COO$^-$$_{PRO}$)	PROLIN03 (III): $R^2_2(7)$<br>QANRUT (III): $C^1_2(5)$, $C^2_2(7)$, **$C^4_4(18)$**, $R^2_2(8)$, $R^4_4(16)$<br>NELSEC (III): **$C^4_4(18)$**
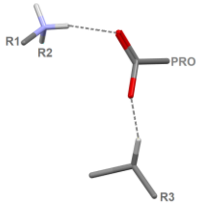<br>(NH$_3^+$)N-H<br>(C$_{cycl}$ PRO)C-H ⋯> O=C(COO$^-$$_{PRO}$)	GERXAE (II): $C^1_2(12)$, **$C^2_2(14)$, $R^2_2(14)$**<br>GERXEI (II): **$R^2_2(14)$**<br>GERWUX01 (II): **$C^2_2(14)$, $R^2_2(14)$**
(NH$_3^+$)N-H<br>(-C)C-H ⋯> O=C(COO$^-$$_{PRO}$)	GERWUX01 (II): $C^1_2(6)$, $C^2_2(8)$
(NH$_3^+$)N-H<br>(C$_{cycl}$)C-H ⋯> O=C(COO$^-$$_{PRO}$)	GERXEI (II): $C^2_2(19)$
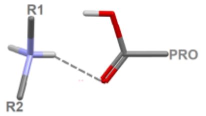<br>(NH$_2^+$)N-H⋯O=C(COOH$_{PRO}$)	RUWBAM (I): C(8), $C^2_2(13)$, $C^2_2(15)$, $C^2_2(19)$, $R^1_2(7)$, $R^2_2(11)$, $R^2_2(13)$, $D^3_3(13)$, $D^3_3(15)$, $D^3_3(16)$
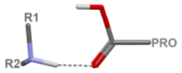<br>(-N)N-H⋯O=C(COOH$_{PRO}$)	IQISAE (I): C(8), $C^2_2(13)$, $C^2_2(14)$, $C^2_2(16)$, $C^2_2(18)$, $C^2_2(19)$
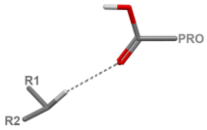<br>(C$_{cycl}$ PRO)C-H⋯O=C(COOH$_{PRO}$)	BECWIR (I): **C(4), C(6), $C^2_2(10)$**, **$C^2_2(14)$**, **$C^2_2(15)$**, $C^2_2(16)$, **$C^2_2(17)$**, $D^3_3(9)$, **$D^3_3(13)$**, **$D^3_3(17)$**, $D^3_3(18)$<br>FEFKEI (I): **C(4)**, **$C^2_2(10)$**, $C^2_2(12)$, **$C^2_2(13)$**, **$C^2_2(15)$**, **$C^2_2(17)$**, $R^2_2(10)$, **$R^2_2(12)$**, **$D^3_3(13)$**, **$D^3_3(17)$**<br>MCPRL01 (I): **C(6)**<br>YOZTIS (I): $C^2_2(9)$, **$C^2_2(13)$**, **$C^2_2(14)$**, $C^2_2(18)$, **$R^2_2(12)$**, $R^2_2(18)$
(C$_{cycl}$)C-H⋯O=C(COOH$_{PRO}$)	BECWIR (I): $C^2_2(15)$, $C^2_2(20)$, $R^2_2(14)$, $R^2_2(20)$, D(2), $D^2_2(10)$, $D^2_2(12)$, $D^2_2(14)$, $D^2_3(7)$, $D^2_3(9)$, $D^3_3(16)$, $D^3_3(17)$
(-C)C-H⋯O=C(COOH$_{PRO}$)	IQISAE (I): C(8), **$C^2_2(15)$, $C^2_2(16)$, $C^2_2(18)$,** $C^2_2(19)$<br>YOZTIS (I): $C^2_2(8)$, $C^2_2(10)$, $C^2_2(14)$, **$C^2_2(15)$, $C^2_2(16)$, $C^2_2(18)$**, $C^2_2(20)$<br>QOQWAU: S(10)
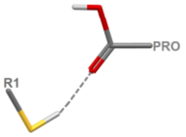<br>(-S)S-H⋯O=C(COOH$_{PRO}$)	MCPRL01 (I): C(9), $C^2_2(6)$, $C^2_2(10)$, $C^2_2(15)$, $C^2_2(16)$, $C^2_2(17)$
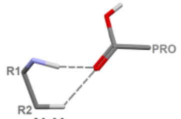<br>(-N)N-H<br>(-C)C-H ⋯> O=C(COOH$_{PRO}$)	IQISAE (I): $R^1_2(6)$
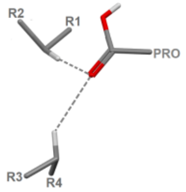<br>[(C$_{cycl}$ PRO)C-H]$_2$ ⋯O=C(COOH$_{PRO}$)	BECWIR (I): $R^1_2(6)$<br>MCPRL01 (I): $C^1_2(4)$, $R^2_3(10)$
(C$_{cycl}$ PRO)C-H<br>(C$_{cycl}$)C-H ⋯> O=C(COOH$_{PRO}$)	BECWIR (I): $D^2_3(7)$, $D^2_3(9)$
[(-C)C-H]$_2$⋯O=C(COOH$_{PRO}$)	YOZTIS (I): $C^1_2(8)$, $R^1_2(6)$

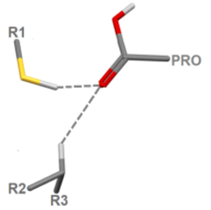<br>(C$_{cycl}$ PRO)C-H···O=C(COOH$_{PRO}$)<br>(-S)S-H	MCPRL01 (I): $C^{1}_{2}(9)$, $R^{1}_{2}(9)$, $R^{2}_{3}(15)$
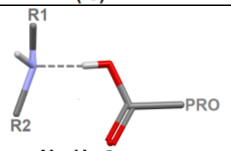<br>(-N)N···H=O(COOH$_{PRO}$)	QOQWAU (I): C(8), $C^{2}_{2}(11)$, $C^{2}_{2}(16)$, $C^{2}_{2}(18)$
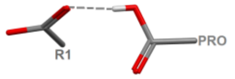<br>(COO-)C=O···H=O(COOH$_{PRO}$)	BECWIR (I): **C(11), $C^{2}_{2}(15)$, $C^{2}_{2}(17)$, $D^{2}_{3}(14)$, $D^{3}_{3}(16)$, $D^{3}_{3}(18)$, $D^{3}_{3}(20)$**<br>FEFKEI (I): **C(11), $C^{2}_{2}(12)$**, $C^{2}_{2}(13)$, **$C^{2}_{2}(15)$, $C^{2}_{2}(16)$, $C^{2}_{2}(17)$**, $C^{2}_{2}(18)$, **$C^{2}_{2}(20)$**, $R^{2}_{2}(19)$, **$D^{2}_{3}(14)$, $D^{3}_{3}(16)$, $D^{3}_{3}(18)$**<br>CIYNIH (I): $C^{1}_{2}(16)$, $C^{2}_{2}(11)$, **$C^{2}_{2}(12)$, $C^{2}_{2}(15)$, $C^{2}_{2}(16)$, $C^{2}_{2}(17)$, $C^{2}_{2}(20)$, $D^{3}_{3}(16)$, $D^{3}_{3}(18)$, $D^{3}_{3}(20)$**
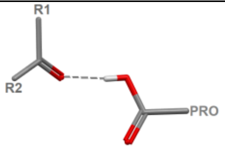<br>(C=O)O ···H=O(COOH$_{PRO}$)	CIYNIH (I): C(11)<br>MCPRL01 (I): $C^{2}_{2}(9)$, $C^{2}_{2}(10)$, **$C^{2}_{2}(13)$**, $C^{2}_{2}(14)$, **$C^{2}_{2}(15)$**, $C^{2}_{2}(16)$<br>YOZTIS (I): C(7), $C^{1}_{2}(8)$, $C^{1}_{2}(15)$, $C^{2}_{2}(9)$, $C^{2}_{2}(11)$, **$C^{2}_{2}(13)$, $C^{2}_{2}(15)$**, $C^{2}_{2}(17)$, $C^{2}_{2}(19)$
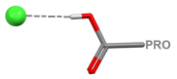<br>Cl^{-} ···H=O(COOH$_{PRO}$)	RUWBAM (I): $C^{1}_{2}(8)$, $C^{1}_{2}(9)$, $C^{1}_{2}(10)$, D(2), $D^{1}_{2}(3)$, $D^{2}_{2}(8)$, $D^{2}_{2}(9)$, $D^{2}_{2}(10)$, $D^{3}_{3}(15)$, $D^{3}_{3}(18)$
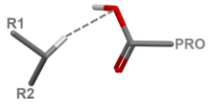<br>(C$_{cycl}$)C-H···O(OH$_{COOHPRO}$)	FEFKEI (I): C(8), **$C^{2}_{2}(11)$, $C^{2}_{2}(12)$**, $C^{2}_{2}(13)$, $C^{2}_{2}(14)$, **$C^{2}_{2}(19)$**, $R^{2}_{2}(12)$, $R^{2}_{2}(13)$, $R^{2}_{2}(17)$, $R^{2}_{2}(19)$, **$D^{3}_{3}(15)$, $D^{3}_{3}(19)$**<br>BECWIR (I): **$C^{2}_{2}(15)$, $C^{2}_{2}(19)$**, D(2), $D^{2}_{2}(10)$, $D^{2}_{2}(11)$, $D^{2}_{2}(12)$, $D^{2}_{2}(13)$, $D^{3}_{3}(9)$, $D^{3}_{3}(13)$, $D^{3}_{3}(14)$, **$D^{3}_{3}(15)$**<br>CIYNIH (I): C(14), **$C^{2}_{2}(11)$, $C^{2}_{2}(12)$, $C^{2}_{2}(15)$**, $C^{2}_{2}(17)$, **$C^{2}_{2}(19)$, $D^{3}_{3}(19)$**
(C$_{cycl}$ PRO)C-H···O(OH$_{COOHPRO}$)	TUHMUE: C(14), C(15)
(-C)C-H···O(OH$_{COOHPRO}$)	BECWIR (I): C(10), **$C^{2}_{2}(14)$**, $C^{2}_{2}(16)$, $D^{3}_{3}(15)$, $D^{3}_{3}(17)$<br>FEFKEI (I):C(9), $C^{2}_{2}(12)$, **$C^{2}_{2}(13)$, $C^{2}_{2}(14)$, $C^{2}_{2}(15)$, $C^{2}_{2}(16)$**, $C^{2}_{2}(20)$, $R^{2}_{2}(11)$, $R^{2}_{2}(17)$, $D^{3}_{3}(14)$, $D^{3}_{3}(16)$<br>IQISAE (I): S(10)<br>YOZTIS (I): **C(8)**<br>MCPRL01(I):**C(8)**, $C^{2}_{2}(7)$, **$C^{2}_{2}(14)$, $C^{2}_{2}(15)$**, $C^{2}_{2}(17)$<br>QOQWAU (I): **C(8), $C^{2}_{2}(13)$, $C^{2}_{2}(16)$**, $C^{2}_{2}(18)$
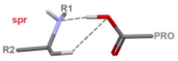<br>(-N)N···H-O(OH$_{COOHPRO}$)<br>(-C)C-H	QOQWAU (I):$R^{2}_{2}(6)$
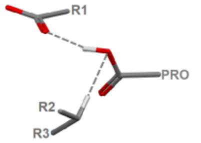<br>(COO-)C=O···H-O (OH$_{COOHPRO}$)<br>(C$_{cycl}$)C-H	BECWIR (I): $D^{3}_{3}(14)$
(COO-)C=O···H-O (OH$_{COOHPRO}$)<br>(-C)C-H	BECWIR (I): $R^{2}_{2}(7)$<br>FEFKEI (I): $R^{2}_{2}(6)$
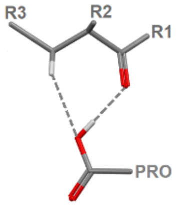<br>(C=O)O···H-O(OH$_{COOHPRO}$)<br>(-C)C-H	MCPRL01 (I): $R^{2}_{2}(7)$
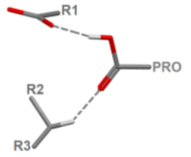	BECWIR (I): $R^{2}_{2}(13)$, $R^{2}_{2}(14)$, $D^{2}_{3}(14)$<br>FEFKEI (I):$R^{2}_{2}(13)$

$(COO^{-})O\cdots H\text{-}O(OH_{COOH\,PRO})$<br>$(C_{cycl\,PRO})C\text{-}H\cdots O(C{=}O_{COOH\,PRO})$	
$(COO^{-})O\cdots H\text{-}O(OH_{COOH\,PRO})$<br>$(C_{cycl})C\text{-}H\cdots O(C{=}O_{COOH\,PRO})$	BECWIR (I): D^{3}_{3}(16)
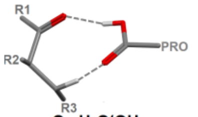<br><br>$(C{=}O)O\cdots H\text{-}O(OH_{COOH\,PRO})$<br>$(\text{-}C)C\text{-}H\cdots O(C{=}O_{COOH\,PRO})$	YOZTIS (I): R^{2}_{2}(9)
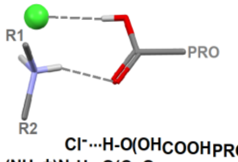<br><br>$Cl^{-}\cdots H\text{-}O(OH_{COOH\,PRO})$<br>$(NH_2^{+})N\text{-}H\cdots O(C{=}O_{COOH\,PRO})$	RUWBAM (I): D^{3}_{3}(15)
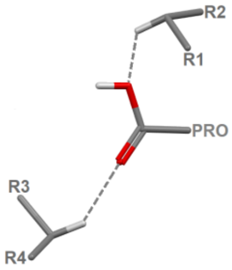<br><br>$(C_{cycl\,PRO})C\text{-}H\cdots O{=}C(COOH_{PRO})$<br>$(\text{-}C)C\text{-}H\cdots O(OH_{COOH\,PRO})$	BECWIR (I): R^{2}_{2}(12), R^{2}_{2}(13)<br>FEFKEI (I): R^{2}_{2}(11)<br>MCPRL01 (I): C^{2}_{2}(10)
$(C_{cycl\,PRO})C\text{-}H\cdots O{=}C(COOH_{PRO})$<br>$(\text{-}C)C\text{-}H\cdots O(OH_{COOH\,PRO})$	FEFKEI (I): C^{2}_{2}(10)
$(C_{cycl\,PRO})C\text{-}H\cdots O{=}C(COOH_{PRO})$<br>$(C_{cycl})C\text{-}H\cdots O(OH_{COOH\,PRO})$	BECWIR (I): D^{3}_{3}(11)
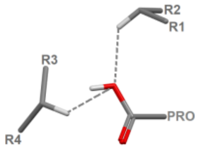<br><br>$(\text{-}C)C\text{-}H\cdots O{=}C(COOH_{PRO})$<br>$(C_{cycl})C\text{-}H\cdots O(OH_{COOH\,PRO})$	BECWIR (I):D^{2}_{3}(11)<br>FEFKEI (I): C^{1}_{2}(11)
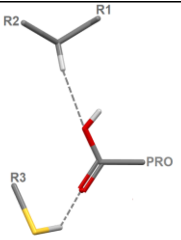<br><br>$(\text{-}S)S\text{-}H\cdots O{=}C(COOH_{PRO})$<br>$(\text{-}C)C\text{-}H\cdots O(OH_{COOH\,PRO})$	MCPRL01 (I):C^{2}_{2}(7)
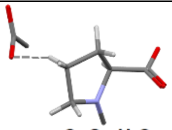<br>$(COO^{-})C{=}O\cdots H\text{-}C(C_{cycl\,PRO})$	DIVHOF01 (II): C^{2}_{2}(11), C^{2}_{2}(12), C^{2}_{2}(14), C^{2}_{2}(15), R^{2}_{2}(14), R^{2}_{2}(17), D(2), D^{2}_{3}(8), D^{3}_{3}(11), D^{3}_{3}(12)
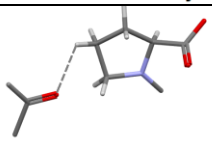<br>$(C{=}O)O\cdots H\text{-}C(C_{cycl\,PRO})$	TUHMUE: S(9)<br>DIVHOF01 (II): C(5), **C(6)**, **C^{2}_{2}(11)**, **C^{2}_{2}(13)**, **C^{2}_{2}(14)**, C^{2}_{2}(16), **C^{2}_{2}(17)**, R^{1}_{2}(5), R^{2}_{2}(16), R^{2}_{2}(17), R^{3}_{3}(19), D^{3}_{3}(14), **D^{3}_{3}(15)**, D^{3}_{3}(16), D^{3}_{3}(17), D^{3}_{3}(18), **D^{3}_{3}(19)**, D^{3}_{3}(20)<br>MCPRL01 (I): C^{2}_{2}(9), **C^{2}_{2}(11)**, **C^{2}_{2}(12)**, **C^{2}_{2}(13)**, **C^{2}_{2}(14)**, C^{2}_{2}(15), R^{2}_{3}(10), R^{2}_{3}(15), R^{3}_{4}(16)<br>YOZTIS (I): **C(6)**, C^{1}_{2}(8), C^{2}_{2}(9), **C^{2}_{2}(10)**, **C^{2}_{2}(13)**, **C^{2}_{2}(14)**, C^{2}_{2}(18), C^{2}_{2}(20), R^{2}_{2}(14), R^{2}_{2}(20)<br>FEFKEI (I): **C(6)**, C^{1}_{2}(6), **C^{2}_{2}(10)**, **C^{2}_{2}(12)**, C^{2}_{2}(15), **C^{2}_{2}(17)**, C^{3}_{4}(19), R^{2}_{2}(10), R^{2}_{2}(11), R^{2}_{2}(13), R^{3}_{4}(19), D^{2}_{3}(9), **D^{3}_{3}(15)**, **D^{3}_{3}(19)**
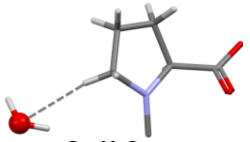<br>$(H_2O)O\cdots H\text{-}C(C_{cycl\,PRO})$	GERXAE (II): C^{1}_{2}(11), **C^{2}_{2}(11)**, R^{2}_{2}(8), **D(2)**, **D^{3}_{3}(11)**, D^{3}_{3}(15), **D^{3}_{3}(17)**, D^{3}_{3}(18), **D^{3}_{3}(19)**, D^{3}_{3}(20)<br>GERWUX01 (II): **C^{2}_{2}(11)**, **D(2)**, D^{2}_{2}(10), D^{2}_{2}(11), **D^{3}_{3}(11)**, **D^{3}_{3}(17)**, **D^{3}_{3}(19)**

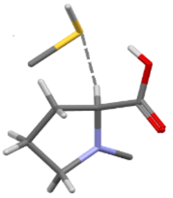 (-S/Scycl)S···H-C(Ccycl PRO)	NELSEC (III): C(4), **$C^2_2(7)$**, **$C^2_2(8)$**, **$C^2_2(9)$**, **$C^2_2(10)$**, $C^4_4(17)$, $R^4_4(17)$<br>MCPRL01 (I): C(7), $C^2_2(6)$, **$C^2_2(7)$**, **$C^2_2(10)$**, **$C^2_2(11)$**, $C^2_2(13)$, **$C^2_2(14)$**, **$C^2_2(15)$**, **$C^2_2(16)$**, $R^2_2(20)$<br>YOZTIS (I): C(8), $C^1_2(16)$, **$C^2_2(8)$**, **$C^2_2(9)$**, **$C^2_2(10)$**, **$C^2_2(11)$**, $C^2_2(12)$, **$C^2_2(14)$**, **$C^2_2(15)$**, **$C^2_2(16)$**, $C^2_2(18)$, $C^2_2(20)$, $R^2_2(10)$, $R^2_2(12)$
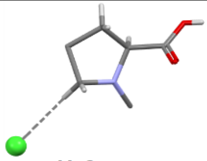 Cl⁻···H-C(Ccycl PRO)	RUWBAM (I): $C^1_2(8)$, $D^1_2(3)$, $D^2_2(7)$, $D^2_2(8)$, $D^3_3(14)$, $D^3_3(18)$
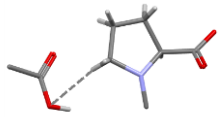 (COOH OH)O···H-C(Ccycl PRO)	GERXEI (II): C(9), $C^1_2(11)$, $C^2_2(10)$, $C^2_2(12)$, $C^2_2(13)$, $C^2_2(14)$, $C^2_2(15)$, $C^2_2(16)$, $C^2_2(17)$, $C^2_2(18)$, $R^2_2(11)$, $R^3_3(19)$
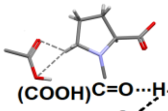 (COOH)C=O···H-C(Ccycl PRO) (COOH OH)O	GERXEI (II): $R^2_1(4)$

Table S16. Supramolecular synthon patterns involving COOH/COO- groups of proline ring, in relations to three types of proline-based tectons, observed in the ACEI crystal structures.

	Tecton I	*Tecton II*	*Tecton III*
C(4)	BECWIR, FEFKEI		PROLIN03
C(5)		FIFGEG,	NELSEC, PROLIN03
C(6)	BECWIR, MCPRL01, YOZTIS	GERWUX01, GERXAE, GERXEI,	NELSEC
C(7)	MCPRL01, YOZTIS		
C(8)	FEFKEI, MCPRL01, YOZTIS, IQISAE, QOQWAU, RUWBAM	DIVHOF01	
C(9)	BECWIR, FEFKEI, MCPRL01	FIFGEG	
C(11)	BECWIR, CIYNIH		
C(12)		GERWUX01, GERXAE, GERXEI,	
C(14)	CIYNIH		
C(15)		GERXEI, EDALEC	
$C^1_2(4)$	MCPRL01	UZOVAH, UZOVAH03, GERWUX01, GERXAE, GERXEI	PROLIN03
$C^1_2(5)$			NELSEC, PROLIN03
$C^1_2(6)$		UZOVAH	
$C^1_2(8)$	YOZTIS, RUWBAM		
$C^1_2(9)$	RUWBAM		
$C^1_2(10)$	RUWBAM		
$C^1_2(11)$	BECWIR, FEFKEI	GERXAE?	
$C^1_2(12)$		GERWUX01, GERXAE, GERXEI	
$C^1_2(15)$	YOZTIS	GERXEI	
$C^1_2(16)$	CIYNIH		
$C^1_2(17)$		GERXEI	
$C^2_2(6)$	MCPRL01	IVEGIA, UZOVAH, UZOVAH03, GERWUX01, GERXAE, GERXEI,	NELSEC, PROLIN03
$C^2_2(7)$	MCPRL01	IVEGIA, FIFGEG	NELSEC
$C^2_2(8)$	YOZTIS	IVEGIA, UZOVAH	NELSEC
$C^2_2(9)$	MCPRL01, YOZTIS	EDALEC	NELSEC, PROLIN03
$C^2_2(10)$	BECWIR, FEFKEI, MCPRL01, YOZTIS	GERWUX01, GERXAE,	NELSEC, PROLIN03
$C^2_2(11)$	BECWIR, FEFKEI, CIYNIH, MCPRL01, YOZTIS, QOQWAU	DIVHOF01,	NELSEC
$C^2_2(12)$	BECWIR, FEFKEI, CIYNIH, MCPRL01, YOZTIS	GERWUX01, GERXAE, GERXEI,	
$C^2_2(13)$	BECWIR, FEFKEI, MCPRL01, YOZTIS, IQISAE, QOQWAU, RUWBAM	DIVHOF01, GERXEI,	
$C^2_2(14)$	BECWIR, FEFKEI, IQISAE, MCPRL01, YOZTIS	DIVHOF01, GERWUX01, GERXAE, GERXEI, FIFGEG	
$C^2_2(15)$	BECWIR, FEFKEI, CIYNIH, IQISAE, YOZTIS, MCPRL01, RUWBAM	DIVHOF01, GERXAE, GERXEI,	
$C^2_2(16)$	BECWIR, FEFKEI, CIYNIH, IQISAE, YOZTIS, MCPRL01, QOQWAU	GERWUX01, GERXAE, GERXEI,	
$C^2_2(17)$	BECWIR, FEFKEI, CIYNIH, MCPRL01, YOZTIS	GERWUX01, GERXAE, GERXEI, FIFGEG	
$C^2_2(18)$	BECWIR, FEFKEI, YOZTIS, IQISAE, QOQWAU	GERWUX01, GERXAE, GERXEI	
$C^2_2(19)$	BECWIR, FEFKEI, CIYNIH, YOZTIS, IQISAE, RUWBAM	GERWUX01, GERXAE, GERXEI,	
$C^2_2(20)$	BECWIR, FEFKEI, CIYNIH, YOZTIS,	GERWUX01, GERXAE, GERXEI	
$C^3_4(14)$			PROLIN03
$C^3_4(16)$			NELSEC
$C^4_4(16)$			PROLIN03
$C^4_4(17)$			NELSEC

$C^{4}_{4}(18)$			NELSEC
$R^{1}_{2}(5)$			PROLIN03
$R^{1}_{2}(6)$	BECWIR, IQISAE, YOZTIS	UZOVAH	
$R^{1}_{2}(9)$	MCPRL01		
$R^{1}_{2}(10)$		FIFGEG	
$R^{2}_{1}(4)$			PROLIN03
$R^{2}_{2}(6)$	FEFKEI, QOQWAU		
$R^{2}_{2}(7)$	BECWIR, MCPRL01		PROLIN03
$R^{2}_{2}(8)$		UZOVAH	
$R^{2}_{2}(9)$	YOZTIS		
$R^{2}_{2}(10)$	FEFKEI, YOZTIS	GERWUX01, GERXAE	
$R^{2}_{2}(11)$	FEFKEI, RUWBAM		
$R^{2}_{2}(12)$	BECWIR, FEFKEI, YOZTIS	GERWUX01, GERXAE, GERXEI	
$R^{2}_{2}(13)$	BECWIR, FEFKEI, RUWBAM	GERXEI	
$R^{2}_{2}(14)$	BECWIR	GERWUX01, GERXAE, GERXEI	
$R^{2}_{2}(16)$		GERWUX01, GERXAE	
$R^{2}_{2}(17)$	FEFKEI	GERWUX01, GERXAE, GERXEI	
$R^{2}_{2}(18)$	YOZTIS		
$R^{2}_{2}(19)$	FEFKEI	GERWUX01, GERXAE, GERXEI	
$R^{2}_{2}(20)$	BECWIR, FEFKEI,	GERXEI	
$R^{2}_{3}(10)$	MCPRL01		
$R^{2}_{3}(15)$	MCPRL01		
$R^{2}_{3}(16)$		GERWUX01, GERXEI	
$R^{3}_{3}(19)$		DIVHOF01	
$R^{3}_{4}(14)$	BECWIR		PROLIN03
$R^{3}_{4}(16)$	MCPRL01		NELSEC
$R^{4}_{4}(14)$	BECWIR		NELSEC
$R^{4}_{4}(17)$			NELSEC
D(2)	BECWIR, RUWBAM	IVEGIA, UZOVAH, UZOVAH03	
$D^{1}_{2}(3)$	RUWBAM	IVEGIA	
$D^{2}_{2}(5)$		IVEGIA	
$D^{2}_{2}(8)$	RUWBAM		
$D^{2}_{2}(9)$	RUWBAM		
$D^{2}_{2}(10)$	BECWIR, RUWBAM		
$D^{2}_{2}(12)$	BECWIR	IVEGIA	
$D^{2}_{2}(14)$	BECWIR	IVEGIA	
$D^{2}_{3}(7)$	BECWIR		
$D^{2}_{3}(13)$	BECWIR		
$D^{2}_{3}(14)$	FEFKEI, CIYNIH		
$D^{3}_{3}(9)$	BECWIR		
$D^{3}_{3}(11)$	BECWIR	GERWUX01, GERXAE	
$D^{3}_{3}(13)$	FEFKEI, RUWBAM	DIVHOF01	
$D^{3}_{3}(14)$	BECWIR, FEFKEI	UZOVAH, FIFGEG	
$D^{3}_{3}(15)$	BECWIR, FEFKEI, RUWBAM	UZOVAH, DIVHOF01, GERWUX01, GERXAE	
$D^{3}_{3}(16)$	BECWIR, FEFKEI, CIYNIH, RUWBAM	UZOVAH, DIVHOF01, FIFGEG	
$D^{3}_{3}(17)$	BECWIR, FEFKEI,	UZOVAH, DIVHOF01, GERWUX01, GERXAE	
$D^{3}_{3}(18)$	BECWIR, FEFKEI, CIYNIH, RUWBAM	UZOVAH, DIVHOF01, FIFGEG	
$D^{3}_{3}(19)$	FEFKEI, CIYNIH	DIVHOF01, GERWUX01, GERXAE	
$D^{3}_{3}(20)$	BECWIR, CIYNIH	IVEGIA	
S(10)	QOQWAU		

Table S17. Supramolecular synthon patterns formed by **COOH(COO^{-})** of pyrrolidine ring of proline in investigated crystals.

	*Tecton **I***
C(4)	BECWIR, FEFKEI<br>$*_{(CH)}C\text{-}H\cdots O_{(C=O/COO-)}$
C(6)	BECWIR, FEFKEI, YOZTIS, MCPRL01<br>$*_{(CH)}C\text{-}H\cdots O_{(C=O/COO-)}$
C(7)	MCPRL01, YOZTIS<br>$*_{(OH)}O\text{-}H\cdots O_{(C=O/COO-)}$
C(8)	FEFKEI, MCPRL01, QOQWAU<br>$_{(CH)}C\text{-}H\cdots O_{(OH)}$
C(11)	BECWIR, CIYNIH<br>$*_{(OH)}O\text{-}H\cdots O_{(C=O/COO-)}$
$C^{2}_{2}(9)$	MCPRL01, YOZTIS<br>$*_{(OH)}O\text{-}H\cdots O_{(C=O/COO-)}$ & $_{(CH)}C\text{-}H\cdots O_{(C=O/COO-)}$
$C^{2}_{2}(10)$	BECWIR, FEFKEI, MCPRL01<br>$*_{(NH)}N\text{-}H\cdots O_{(C=O/COO-)}$ & $_{(CH)}C\text{-}H\cdots O_{(C=O/COO-)}$<br>$*_{(NH)}N\text{-}H\cdots O_{(C=O/COO-)}$ & $_{(NH)}N\text{-}H\cdots O_{(C=O/COO-)}$<br>$*_{(CH)}C\text{-}H\cdots O_{(C=O/COO-)}$ & $_{(CH)}C\text{-}H\cdots O_{(C=O/COO-)}$<br>$*_{(CH)}C\text{-}H\cdots O_{(C=O)}$ & $_{(CH)}C\text{-}H\cdots O_{(OH)}$
$C^{2}_{2}(12)$	FEFKEI, CIYNIH, MCPRL01,<br>$*_{(OH)}O\text{-}H\cdots O_{(C=O/COO-)}$ & $_{(NH)}N\text{-}H\cdots O_{(C=O/COO-)}$<br>$*_{(NH)}N\text{-}H\cdots O_{(C=O/COO-)}$ & $_{(CH)}C\text{-}H\cdots O_{(OH)}$<br>$*_{(CH)}C\text{-}H\cdots O_{(C=O/COO-)}$ & $_{(CH)}C\text{-}H\cdots O_{(C=O/COO-)}$<br>$*_{(NH)}N\text{-}H\cdots O_{(C=O/COO-)}$ & $_{(CH)}C\text{-}H\cdots O_{(C=O/COO-)}$
$C^{2}_{2}(13)$	MCPRL01, YOZTIS<br>$*_{(OH)}O\text{-}H\cdots O_{(C=O/COO-)}$ & $_{(CH)}C\text{-}H\cdots O_{(C=O/COO-)}$
$C^{2}_{2}(14)$	BECWIR, FEFKEI, MCPRL01, CIYNIH, IQISAE<br>$*_{(CH)}C\text{-}H\cdots O_{(C=O/COO-)}$ & $_{(CH)}C\text{-}H\cdots O_{(OH)}$<br>$*_{(NH)}N\text{-}H\cdots O_{(C=O/COO-)}$ & $_{(CH)}C\text{-}H\cdots O_{(OH)}$<br>$*_{(NH)}N\text{-}H\cdots O_{(C=O/COO-))}$ & $_{(CH)}C\text{-}H\cdots O_{(C=O/COO-)}$

$C^2_2(15)$	BECWIR, FEFKEI, YOZTIS, CIYNIH<br>*$_{(OH)}$O-H···O$_{(C=O/COO-)}$ & $_{(CH)}$C-H···O$_{(C=O/COO-)}$<br>*($_{(CH)}$C-H···O$_{(C=O/COO-)}$)$_2$ & $_{(CH)}$C-H···O$_{(OH)}$
$C^2_2(16)$	BECWIR, FEFKEI, CIYNIH<br>*$_{(OH)}$O-H···O$_{(C=O/COO-)}$ & $_{(NH)}$N-H···O$_{(C=O/COO-)}$<br>*$_{(CH)}$C-H···O$_{(C=O/COO-)}$ & $_{(CH)}$C-H···O$_{(OH)}$
$C^2_2(17)$	BECWIR, FEFKEI, YOZTIS, CIYNIH<br>*$_{(OH)}$O-H···O$_{(C=O/COO-)}$ & $_{(CH)}$C-H···O$_{(C=O/COO-)}$<br>*N-H···O$_{(C=O/COO-)}$ & $_{(CH)}$C-H···O$_{(C=O/COO-)}$<br>*$_{(NH)}$N-H···O$_{(C=O/COO-)}$ & $_{(CH)}$C-H···O$_{(OH)}$
$C^2_2(19)$	BECWIR, FEFKEI, CIYNIH<br>$_{(NH)}$N-H···O$_{(C=O/COO-)}$ & $_{(CH)}$C-H···O$_{(OH)}$
$C^2_2(20)$	FEFKEI, CIYNIH<br>$_{(OH)}$O-H···O$_{(C=O/COO-)}$ & $_{(CH)}$C-H···O$_{(OH)}$<br>$_{(OH)}$O-H···O$_{(C=O/COO-)}$ & $_{(CH)}$C-H···O$_{(C=O/COO-)}$
$R^1_2(6)$	BECWIR, YOZTIS<br>*$_{(CH)}$C-H···O$_{(C=O/COO-)}$ & $_{(CH)}$C-H···O$_{(C=O/COO-)}$
$R^2_2(7)$	BECWIR, MCPRL01<br>*$_{(OH)}$O-H···O$_{(C=O/COO-)}$ & $_{(CH)}$C-H···O$_{(OH)}$
$R^2_2(12)$	BECWIR, FEFKEI<br>*$_{(CH)}$C-H···O$_{(C=O/COO-)}$ & $_{(CH)}$C-H···O$_{(OH)}$
$D(2)$	BECWIR, CIYNIH<br>*$_{(CH)}$C-H···O$_{(C=O_COOH)}$<br>*$_{(CH)}$C-H···O$_{(OH)}$<br>*$_{(NH)}$N-H···O$_{(OH)}$
$D^2_3(14)$	FEFKEI, CIYNIH<br>$_{(OH)}$O-H···O$_{(C=O/COO-)}$ & ($_{(OH)}$O-H···O$_{(C=O/COO-)}$)$_2$
$D^3_3(14)$	BECWIR, FEFKEI, CIYNIH<br>*$_{(OH)}$O-H···O$_{(C=O/COO-)}$ & ($_{(CH)}$C-H···O$_{(OH)}$)$_2$<br>*$_{(NH)}$N-H···O$_{(C=O/COO-)}$ & ($_{(CH)}$C-H···O$_{(OH)}$)$_2$
$D^3_3(16)$	FEFKEI, CIYNIH<br>*$_{(OH)}$O-H···O$_{(C=O/COO-)}$ & ($_{(NH)}$N-H···O$_{(OH)}$)$_2$<br>*$_{(OH)}$O-H···O$_{(C=O/COO-)}$ & ($_{(OH)}$O-H···O$_{(C=O/COO-)}$)$_2$<br>*($_{(NH)}$N-H···O$_{(OH)}$)$_2$ & $_{(CH)}$C-H···O$_{(C=O/COO-)}$
$D^3_3(18)$	FEFKEI, CIYNIH<br>$_{(OH)}$O-H···O$_{(C=O/COO-)}$ & ($_{(CH)}$C-H···O$_{(OH)}$)$_2$
$D^3_3(19)$	FEFKEI, CIYNIH<br>*$_{(CH)}$C-H···O$_{(OH)}$ & ($_{(CH)}$C-H···O$_{(OH)}$)$_2$
$D^3_3(20)$	BECWIR, CIYNIH<br>*$_{(OH)}$O-H···O$_{(C=O/COO-)}$ & ($_{(CH)}$C-H···O$_{(OH)}$)$_2$<br>*($_{(OH)}$O-H···O$_{(C=O/COO-)}$)$_2$ & $_{(CH)}$C-H···O$_{(C=O/COO-)}$

	Tecton ***II***
$C(5)$	DIFHOF01, FIFGEG<br>*$_{(CH)}$C-H···O$_{(C=O/COO-)}$
$C(6)$	DIFHOF01, GERXAE, GERXEI, GERWUX01<br>*$_{(CH)}$C-H···O$_{(C=O/COO-)}$
$C(12)$	GERXAE, GERXEI, GERWUX01<br>*$_{(NH)}$N-H···O$_{(C=O/COO-)}$
$C^1_2(4)$	GERXAE, GERXEI, GERWUX01, UZOVAH03<br>$_{(NH)}$N-H···O$_{(C=O/COO-)}$ & $_{(NH)}$N-H···O$_{(C=O/COO-)}$
$C^1_2(11)$	GERXAE, GERXEI<br>$_{(OH)}$O-H···O$_{(OH)}$ & $_{(CH)}$C-H···O$_{(OH)}$<br>$_{(OH)}$O-H···O$_{(OH)}$ & $_{(COO-)}$C-H···O$_{(OH)}$
$C^1_2(12)$	GERXAE, GERXEI, GERWUX01<br>$_{(NH)}$N-H···O$_{(C=O/COO-)}$ & $_{(CH)}$C-H···O$_{(C=O/COO-)}$
$C^2_2(6)$	IVEGIA, GERXAE, GERXEI, GERWUX01<br>[$_{(NH)}$N-H···O5$_{(C=O/COO-)}$]$_2$
$C^2_2(10)$	GERXEI, GERWUX01<br>*$_{(NH)}$N-H···O$_{(C=O/COO-)}$ & $_{(CH)}$C-H···O$_{(C=O/COO-)}$
$C^2_2(11)$	DIFHOF01, GERXEI,<br>*$_{(NH)}$N-H···O$_{(C=O/COO-)}$ & $_{(CH)}$C-H···O$_{(C=O/COO-)}$<br>*$_{(CH)}$C-H···O$_{(C=O/COO-)}$ & $_{(CH)}$C-H···O$_{(C=O/COO-)}$
$C^2_2(12)$	GERXAE, GERXEI, GERWUX01<br>*$_{(NH)}$N-H···O$_{(C=O/COO-)}$ & $_{(CH)}$C-H···O$_{(C=O/COO-)}$
$C^2_2(14)$	DIFHOF01, GERXAE, GERXEI, GERWUX01, FIFGEG<br>*$_{(NH)}$N-H···O$_{(C=O/COO-)}$ & $_{(CH)}$C-H···O$_{(C=O/COO-)}$<br>*$_{(CH)}$C-H···O$_{(C=O/COO-)}$ & $_{(CH)}$C-H···O$_{(C=O/COO-)}$
$C^2_2(15)$	DIFHOF01, GERXAE, GERXEI<br>*$_{(NH)}$N-H···O$_{(C=O/COO-)}$ & $_{(CH)}$C-H···O$_{(C=O/COO-)}$<br>*$_{(CH)}$C-H···O$_{(C=O/COO-)}$ & $_{(CH)}$C-H···O$_{(C=O/COO-)}$
$C^2_2(16)$	DIFHOF01, GERXAE, GERXEI, GERWUX01<br>*$_{(CH)}$C-H···O$_{(C=O/COO-)}$ & $_{(CH)}$C-H···O$_{(C=O/COO-)}$
$C^2_2(17)$	DIFHOF01, GERXAE, GERXEI, GERWUX01, FIFGEG<br>*$_{(CH)}$C-H···O$_{(C=O/COO-)}$ & $_{(CH)}$C-H···O$_{(C=O/COO-)}$<br>*$_{(NH)}$N-H···O$_{(C=O/COO-)}$ & $_{(CH)}$C-H···O$_{(C=O/COO-)}$
$C^2_2(18)$	GERXAE, GERXEI, GERWUX01<br>*$_{(NH)}$N-H···O$_{(C=O/COO-)}$ & $_{(CH)}$C-H···O$_{(C=O/COO-)}$

$C^2_2(19)$	GERXAE, GERXEI, GERWUX01<br>*$_{(NH)}$N-H···O$_{(C=O/COO-)}$ & $_{(NH)}$N-H···O$_{(C=O/COO-)}$<br>*$_{(CH)}$C-H···O$_{(C=O/COO-)}$ & $_{(CH)}$C-H···O$_{(C=O/COO-)}$
$C^2_2(20)$	GERXAE, GERXEI, GERWUX01<br>*$_{(NH)}$N-H···O$_{(C=O/COO-)}$ & $_{(CH)}$C-H···O$_{(C=O/COO-)}$
$R^2_2(10)$	GERXAE, GERWUX01<br>$_{(NH)}$N-H···O$_{(C=O/COO-)}$ & $_{(CH)}$C-H···O$_{(C=O/COO-)}$
$R^2_2(12)$	GERXAE, GERXEI, GERWUX01<br>$_{(NH)}$N-H···O$_{(C=O/COO-)}$ & $_{(CH)}$C-H···O$_{(C=O/COO-)}$
$R^2_2(14)$	DIFHOF01, GERXAE, GERXEI, GERWUX01<br>$_{(NH)}$N-H···O$_{(C=O/COO-)}$ & $_{(CH)}$C-H···O$_{(C=O/COO-)}$<br>$_{(CH)}$C-H···O$_{(C=O/COO-)}$ & $_{(CH)}$C-H···O$_{(C=O/COO-)}$
$R^2_2(16)$	DIFHOF01, GERXAE, GERWUX01<br>$_{(CH)}$C-H···O$_{(C=O/COO-)}$ & $_{(CH)}$C-H···O$_{(C=O/COO-)}$
$R^2_2(17)$	DIFHOF01, GERXAE, GERXEI, GERWUX01<br>*$_{(CH)}$C-H···O$_{(C=O/COO-)}$ & $_{(CH)}$C-H···O$_{(C=O/COO-)}$<br>*$_{(NH)}$N-H···O$_{(C=O/COO-)}$ & $_{(CH)}$C-H···O$_{(C=O/COO-)}$
$R^2_2(19)$	GERXAE, GERXEI, GERWUX01<br>*$_{(NH)}$N-H···O$_{(C=O/COO-)}$ & $_{(NH)}$N-H···O$_{(C=O/COO-)}$<br>*$_{(CH)}$C-H···O$_{(C=O/COO-)}$ & $_{(CH)}$C-H···O$_{(C=O/COO-)}$
$R^2_3(16)$	GERXEI, GERWUX01<br>($_{(NH)}$N-H···O$_{(C=O/COO-)}$)$_2$ & $_{(NH)}$N-H···O$_{(C=O/COO-)}$
$R^3_3(19)$	DIFHOF01, GERXEI<br>*($_{(NH)}$N-H···O$_{(C=O/COO-)}$)$_2$ & $_{(CH2)}$C-H···O$_{(C=O/COO-)}$
$D(2)$	IVEGIA, DIFHOF01, GERXAE<br>*$_{(NH)}$N-H···O$_{(C=O/COO-)}$<br>*$_{(CH)}$C-H···O$_{(OH)}$<br>*$_{(CH)}$C-H···O$_{(C=O/COO-)}$
$D^3_3(11)$	DIFHOF01, GERXAE, GERWUX01<br>*($_{(CH)}$C-H···O$_{(C=O)}$)$_2$ & $_{(CH)}$C-H···O$_{(C=O)}$<br>*$_{(CH)}$C-H···O$_{(C=O/COO-)}$ & ($_{(CH)}$C-H···O$_{(OH)}$)$_2$
$D^3_3(15)$	GERXAE, GERWUX01<br>*$_{(CH)}$C-H···O$_{(C=O/COO-)}$ & $_{(CH)}$C-H···O$_{(OH)}$
$D^3_3(17)$	GERXAE, GERWUX01<br>*($_{(OH)}$O-H···N$_{(NH)}$)$_2$ & $_{(NH)}$N-H···O$_{(C=O/COO-)}$
$D^3_3(18)$	GERXAE, GERWUX01<br>$_{(NH)}$N-H···O$_{(C=O/COO-)}$ & $_{(CH)}$C-H···O$_{(OH)}$
$D^3_3(19)$	GERXAE, GERWUX01<br>*$_{(NH)}$N-H···O$_{(C=O/COO-)}$ & ($_{(CH)}$C-H···O$_{(OH)}$)$_2$

Tecton ***III***	
$C(5)$	PROLIN03, NELSEC, QANRUT<br>$_{(NH)}$N-H···O$_{(C=O/COO-)}$
$C(6)$	$_{CH2cycl)}$C4-H6···O1$_{(COO}$ nelsec ……..
$C^1_2(5)$	PROLIN03, NELSEC, QANRUT<br>$_{(NH)}$N-H···O$_{(C=O/COO-)}$ & $_{(CH)}$C-H···O$_{(C=O/COO-)}$
$C^2_2(6)$	NELSEC, NELSEC, QANRUT<br>[$_{(NH)}$N-H···O$_{(C=O/COO-)}$]$_2$
$C^2_2(10)$	PROLIN03, NELSEC, QANRUT<br>$_{(NH2)}$N-H···O$_{(C=O/COO-)}$ & $_{(NH)}$N-H···O$_{(C=O/COO-)}$
$R^4_4(14)$	PROLIN03, NELSEC, QANRUT<br>($_{(NH)}$N-H···O$_{(C=O/COO-)}$)$_2$ & ($_{(NH)}$N-H···O$_{(C=O/COO-)}$)$_2$

Table S18. Supramolecular synthon patterns forming by COOH(COO^-) in chain of proline in investigated crystals.

	Tecton ***I***	*Tecton* ***II***
C(2)		GERXEI
C(5)	FEFKEI	GERXAE, GERXEI
C(8)		FIFGEG
C(6)		GERXEI
C(9)	CIYNIH	GERXEI
C(11)	BECWIR, FEFKEI, CIYNIH	
$C^1_2(8)$		GERXEI
$C^1_2(10)$		GERXEI
$C^1_2(11)$		GERWUX01, GERXAE, GERXEI
$C^1_2(16)$	CIYNIH	
$C^2_2(4)$		GERWUX01, GERXAE
$C^2_2(7)$	FEFKEI	GERWUX01, GERXAE, FIFGEG
$C^2_2(9)$	FEFKEI	GERXEI
$C^2_2(10)$	BECWIR, FEFKEI	GERWUX01, GERXAE, GERXEI
$C^2_2(11)$	CIYNIH	GERXAE, GERXEI
$C^2_2(12)$	FEFKEI, CIYNIH	GERXEI

$C^2_2(13)$	FEFKEI	GERWUX01, GERXAE, GERXEI
$C^2_2(14)$	FEFKEI, CIYNIH	GERXEI
$C^2_2(15)$	BECWIR, FEFKEI, CIYNIH	GERWUX01, GERXAE, GERXEI
$C^2_2(16)$	FEFKEI, CIYNIH	GERXEI
$C^2_2(17)$	BECWIR, FEFKEI, CIYNIH	GERWUX01, GERXAE, GERXEI, FIFGEG
$C^2_2(18)$	FEFKEI	GERXEI
$C^2_2(19)$	BECWIR, FEFKEI	GERWUX01, GERXAE, GERXEI
$C^2_2(20)$	BECWIR, FEFKEI, CIYNIH	GERXEI
$R^1_2(5)$		GERXEI
$R^1_2(6)$		GERWUX01, GERXAE, GERXEI
$R^1_2(7)$	BECWIR	GERXEI
$R^2_1(4)$		GERXEI
$R^2_2(6)$	FEFKEI	
$R^2_2(7)$	BECWIR	
$R^2_2(11)$		GERXEI
$R^2_2(13)$	BECWIR, FEFKEI	GERWUX01, GERXAE, GERXEI
$R^2_2(15)$		GERXAE, GERXEI
$R^2_2(14)$	BECWIR	
$R^2_2(15)$		GERWUX01
$R^2_2(16)$		GERXEI
$R^2_2(17)$		GERWUX01, GERXAE, GERXEI
$R^2_2(19)$	FEFKEI	GERWUX01, GERXAE, GERXEI
$R^2_2(20)$	BECWIR	GERXEI
$R^2_3(17)$		GERXEI
$R^2_3(19)$		GERXEI
$R^2_3(20)$		GERXEI
$R^3_3(11)$		GERXEI
$R^3_3(12)$		GERXEI
$R^3_3(15)$		GERXEI
$R^4_4(18)$		GERXEI
$R^4_4(20)$		GERXEI
D(2)	BECWIR, FEFKEI, CIYNIH	GERWUX01, GERXAE
$D^1_2(3)$	BECWIR	
$D^2_1(5)$	BECWIR	
$D^2_2(4)$	CIYNIH	
$D^2_2(5)$	BECWIR	
$D^2_2(6)$	BECWIR	
$D^2_2(7)$	BECWIR, CIYNIH	
$D^2_2(8)$	BECWIR, CIYNIH	
$D^2_2(9)$	CIYNIH	
$D^2_2(12)$	BECWIR	
$D^2_2(14)$	BECWIR	
$D^2_3(14)$	FEFKEI, CIYNIH	
$D^3_3(8)$		GERWUX01, GERXAE
$D^3_3(10)$	FEFKEI, CIYNIH	GERWUX01, GERXAE
$D^3_3(12)$	CIYNIH	GERWUX01, GERXAE
$D^3_3(14)$	BECWIR, FEFKEI, CIYNIH	
$D^3_3(15)$	BECWIR	FIFGEG
$D^3_3(16)$	BECWIR, FEFKEI, CIYNIH	
$D^3_3(17)$	BECWIR	GERXAE
$D^3_3(18)$	BECWIR, FEFKEI, CIYNIH	GERWUX01, GERXAE
$D^3_3(19)$	FEFKEI	GERWUX01, GERXAE, FIFGEG
$D^3_3(20)$	BECWIR, FEFKEI, CIYNIH	GERWUX01, GERXAE
S(6)	FEFKEI	

Table S19. Supramolecular synthon **patterns** formed by **(NH_2^+)** in proline chain of investigated crystal structures.

	Tecton I	*Tecton II*
C(5)	FEFKEI, CIYNIH	GERXEI
C(6)		EDALEC
C(8)	RUWBAM	DIVHOF01, FIFGEG
$C^1_2(5)$	RUWBAM	
$C^1_2(6)$	CIYNIH	
$C^1_2(8)$	RUWBAM	GERXEI
$C^1_2(10)$	RUWBAM	DIVHOF01
$C^2_2(7)$	FEFKEI, CIYNIH	GERWUX01, GERXAE
$C^2_2(9)$	BECWIR	GERXEI
$C^2_2(10)$	BECWIR	DIVHOF01, GERWUX01, GERXEI
$C^2_2(11)$	QOQWAU	DIVHOF01, GERXEI
$C^2_2(12)$	FEFKEI, CIYNIH	
$C^2_2(13)$	IQISAE, RUWBAM	DIVHOF01, GERWUX01, GERXAE, GERXEI,
$C^2_2(14)$	FEFKEI, CIYNIH, IQISAE	DIVHOF01, GERXEI,
$C^2_2(15)$	FEFKEI, RUWBAM	DIVHOF01, GERWUX01, GERXAE

$C^2_2(16)$	FEFKEI, CIYNIH, IQISAE, QOQWAU	
$C^2_2(17)$	CIYNIH	GERWUX01, GERXAE, GERXEI
$C^2_2(18)$	IQISAE, QOQWAU	GERXEI
$C^2_2(19)$	BECWIR, FEFKEI, CIYNIH, IQISAE, RUWBAM	GERWUX01, GERXAE, GERXEI, EDALEC
$C^2_2(20)$	BECWIR	GERXEI
$R^1_2(6)$	BECWIR, FEFKEI, IQISAE	GERWUX01, GERXAE, GERXEI
$R^1_2(7)$	BECWIR, CIYNIH	DIVHOF01, GERXEI
$R^2_2(7)$	FEFKEI	
$R^2_2(6)$	QOQWAU	
$R^2_2(8)$		GERXAE
$R^2_2(9)$		DIVHOF01
R22(11)	RUWBAM	
$R^2_2(13)$	RUWBAM	GERWUX01, GERXAE, GERXEI
$R^2_2(14)$		DIVHOF01
$R^2_2(15)$		GERWUX01, GERXAE
R22(17)		GERWUX01, GERXAE, GERXEI
R22(19)		GERWUX01, GERXAE, GERXEI
$R^2_2(20)$	BECWIR	
R23(17)		GERXEI
$R^3_3(19)$		DIVHOF01, GERXEI
R44(18)		GERXEI
D(2)	BECWIR, FEFKEI, CIYNIH, RUWBAM	DIVHOF01, GERWUX01, GERXAE, FIFGEG
$D^1_2(3)$	BECWIR, RUWBAM	
$D^2_2(4)$	CIYNIH	
$D^2_2(5)$	BECWIR	
D22(6)	RUWBAM	
$D^2_2(7)$	BECWIR, CIYNIH	FIFGEG
$D^2_2(8)$	BECWIR	
$D^2_2(10)$	BECWIR, RUWBAM	
$D^2_2(13)$	BECWIR	
$D^3_3(8)$		GERWUX01, GERXAE
$D^3_3(10)$	FEFKEI, CIYNIH	GERWUX01, GERXAE
$D^3_3(11)$		GERWUX01, GERXAE
$D^3_3(12)$	CIYNIH, RUWBAM	DIVHOF01, GERWUX01, GERXAE
D33(13)	RUWBAM	DIVHOF01, GERWUX01, GERXAE
$D^3_3(14)$	FEFKEI, CIYNIH	DIVHOF01, FIFGEG
$D^3_3(15)$	BECWIR, FEFKEI, RUWBAM	DIVHOF01, GERWUX01, GERXAE, FIFGEG
$D^3_3(16)$	BECWIR, FEFKEI, CIYNIH, RUWBAM	DIVHOF01
$D^3_3(17)$	BECWIR, FEFKEI	DIVHOF01, GERWUX01, GERXAE
$D^3_3(18)$	BECWIR	GERWUX01, GERXAE, FIFGEG
$D^3_3(19)$	FEFKEI	DIVHOF01

Table S20. Library of all supramolecular synthon patterns (below 20-membered motifs) in all investigated crystals (proline atoms are black, other atoms – grey, but atoms of COOH(COO^-) of proline ring are in blue color, COOH (COO^-) in the chain – green, while NH(NH_2^+) in the chain of proline – pink).

CSD ref. code	*Motif*	*Interaction (functional group)*
PROLINE structures		
**NELSEC**	*C*(4)	$_{(CHcycl)}$C2-H3···S1$_{cycl}$
	C(5)	$_{(NH2cycl)}$N1-H1···**O1**$_{(COO-)}$<br>$_{(NH2cycl)}$N1-H2···**O2**$_{(COO-)}$
	C(6)	$_{(CH2cycl)}$C4-H6···**O1**$_{(COO-)}$
level 2	$C^1_2(5)$	$_{(NH2cycl)}$N1-H1···**O1**$_{(COO-)}$ & $_{(CH2cycl)}$C4-H6···**O1**$_{(COO-)}$
	$C^2_2(6)$	$_{(NH2cycl)}$N1-H1···**O1**$_{(COO-)}$ & $_{(NH2cycl)}$N1-H2···**O2**$_{(COO-)}$
	$C^2_2(7)$	$_{(NH2cycl)}$N1-H2···**O2**$_{(COO-)}$ & $_{(CH2cycl)}$C4-H6···**O1**$_{(COO-)}$<br>$_{(CHcycl)}$C2-H3···S1 $_{cycl}$ & $_{(CH2cycl)}$C4-H6···**O1**$_{(COO-)}$
	$C^2_2(8)$	*$_{(NH2cycl)}$N1-H1···**O1**$_{(COO-)}$ & $_{(CHcycl)}$C2-H3···S1 $_{cycl}$<br>*$_{(NH2cycl)}$N1-H2···**O2**$_{(COO-)}$ & $_{(CHcycl)}$C2-H3···S1 $_{cycl}$
	$C^2_2(9)$	*$_{(NH2cycl)}$N1-H1···**O1**$_{(COO-)}$ & $_{(CHcycl)}$C2-H3···S1 $_{cycl}$

		*$_{\text{(NH2cycl)}}$N1-H2···**O2**$_{\text{(COO-)}}$ & $_{\text{(CHcycl)}}$C2-H3···S1 $_{\text{cycl}}$
	$C^{2}_{2}(10)$	*$_{\text{(NH2cycl)}}$N1-H1···**O1**$_{\text{(COO-)}}$ & $_{\text{(NH2cycl)}}$N1-H2···**O2**$_{\text{(COO-)}}$<br>*$_{\text{(CH2cycl)}}$C4-H6···**O1**$_{\text{(COO-)}}$ & $_{\text{(CHcycl)}}$C2-H3···S1 $_{\text{cycl}}$
	$C^{2}_{2}(11)$	*$_{\text{(NH2cycl)}}$N1-H1···**O1**$_{\text{(COO-)}}$ & $_{\text{(CH2cycl)}}$C4-H6···**O1**$_{\text{(COO-)}}$<br>*$_{\text{(NH2cycl)}}$N1-H2···**O2**$_{\text{(COO-)}}$ & $_{\text{(CH2cycl)}}$C4-H6···**O1**$_{\text{(COO-)}}$
	$C^{3}_{4}(16)$	($_{\text{(NH2cycl)}}$N1-H1···**O1**$_{\text{(COO-)}}$)$_2$ & ($_{\text{(CH2cycl)}}$C4-H6···**O1**$_{\text{(COO-)}}$)$_2$
	$C^{4}_{4}(17)$	*($_{\text{(NH2cycl)}}$N1-H1···**O1**$_{\text{(COO-)}}$)$_2$ & ($_{\text{(CHcycl)}}$C2-H3···S1 $_{\text{cycl}}$)$_2$<br>*($_{\text{(NH2cycl)}}$N1-H2···**O2**$_{\text{(COO-)}}$)$_2$ & ($_{\text{(CHcycl)}}$C2-H3···S1 $_{\text{cycl}}$)$_2$<br>*($_{\text{(CH2cycl)}}$C4-H6···**O1**$_{\text{(COO-)}}$)$_2$ & $_{\text{(CHcycl)}}$C2-H3···S1 $_{\text{cycl}}$
	$C^{4}_{4}(18)$	($_{\text{(NH2cycl)}}$N1-H2···**O2**$_{\text{(COO-)}}$)$_2$ & ($_{\text{(CH2cycl)}}$C4-H6···**O1**$_{\text{(COO-)}}$)$_2$
	$R^{3}_{3}(11)$	$_{\text{(NH2cycl)}}$N1-H1···**O1**$_{\text{(COO-)}}$ & ($_{\text{(NH2cycl)}}$N1-H2···**O2**$_{\text{(COO-)}}$)$_2$
	$R^{3}_{4}(16)$	($_{\text{(NH2cycl)}}$N1-H1···**O1**$_{\text{(COO-)}}$)2 & ($_{\text{(CH2cycl)}}$C4-H6···**O1**$_{\text{(COO-)}}$)$_2$
	$R^{4}_{4}(14)$	($_{\text{(NH2cycl)}}$N1-H1···**O1**$_{\text{(COO-)}}$)2 & ($_{\text{(NH2cycl)}}$N1-H2···**O2**$_{\text{(COO-)}}$)$_2$
	$R^{4}_{4}(17)$	($_{\text{(NH2cycl)}}$N1-H1···**O1**$_{\text{(COO-)}}$)2 & ($_{\text{(CHcycl)}}$C2-H3···S1 $_{\text{cycl}}$)$_2$
**PROLIN03**	$C(4)$	$_{\text{(CHcycl)}}$C2-H1···**O2**$_{\text{(COO-)}}$
	$C(5)$	*$_{\text{(NH2cycl)}}$N1-H6···**O1**$_{\text{(COO-)}}$<br>*$_{\text{(NH2cycl)}}$N1-H6···**O2**$_{\text{(COO-)}}$<br>*$_{\text{(NH2cycl)}}$N1-H7···**O2**$_{\text{(COO-)}}$
level 2	$C^{1}_{2}(4)$	$_{\text{(NH2cycl)}}$N1-H6···**O1**$_{\text{(COO-)}}$ & $_{\text{(NH2cycl)}}$N1-H7···**O2**$_{\text{(COO-)}}$
	$C^{1}_{2}(5)$	$_{\text{(NH2cycl)}}$N1-H7···**O2**$_{\text{(COO-)}}$ & $_{\text{(CHcycl)}}$C2-H1···**O2**$_{\text{(COO-)}}$
	$C^{2}_{2}(6)$	$_{\text{(NH2cycl)}}$N1-H6···**O1**$_{\text{(COO-)}}$ & $_{\text{(NH2cycl)}}$N1-H7···**O2**$_{\text{(COO-)}}$
	$C^{2}_{2}(9)$	*$_{\text{(NH2cycl)}}$N1-H6···**O1**$_{\text{(COO-)}}$ & $_{\text{(CHcycl)}}$C2-H1···**O2**$_{\text{(COO-)}}$<br>*$_{\text{(NH2cycl)}}$N1-H6···**O2**$_{\text{(COO-)}}$ & $_{\text{(CHcycl)}}$C2-H1···**O2**$_{\text{(COO-)}}$<br>*$_{\text{(NH2cycl)}}$N1-H7···**O2**$_{\text{(COO-)}}$ & $_{\text{(CHcycl)}}$C2-H1···**O2**$_{\text{(COO-)}}$
	$C^{2}_{2}(10)$	*$_{\text{(NH2cycl)}}$N1-H6···**O1**$_{\text{(COO-)}}$ & $_{\text{(NH2cycl)}}$N1-H6···**O2**$_{\text{(COO-)}}$<br>*$_{\text{(NH2cycl)}}$N1-H6···**O1**$_{\text{(COO-)}}$ & $_{\text{(NH2cycl)}}$N1-H7···**O2**$_{\text{(COO-)}}$
	$C^{3}_{4}(14)$	*($_{\text{(NH2cycl)}}$N1-H6···**O2**$_{\text{(COO-)}}$)$_2$ & ($_{\text{(NH2cycl)}}$N1-H7···**O2**$_{\text{(COO-)}}$)$_2$<br>*($_{\text{(NH2cycl)}}$N1-H7···**O2**$_{\text{(COO-)}}$)$_2$ & ($_{\text{(CHcycl)}}$C2-H1···**O2**$_{\text{(COO-)}}$)$_2$
	$C^{4}_{4}(16)$	($_{\text{(NH2cycl)}}$N1-H6···**O1**$_{\text{(COO-)}}$)$_2$ & ($_{\text{(NH2cycl)}}$N1-H7···**O2**$_{\text{(COO-)}}$)$_2$
	$R^{1}_{2}(5)$	$_{\text{(NH2cycl)}}$N1-H6···**O2**$_{\text{(COO-)}}$ & $_{\text{(CHcycl)}}$C2-H1···**O2**$_{\text{(COO-)}}$
	$R^{2}_{1}(4)$	$_{\text{(NH2cycl)}}$N1-H6···**O1**$_{\text{(COO-)}}$ & $_{\text{(NH2cycl)}}$N1-H6···**O2**$_{\text{(COO-)}}$
	$R^{2}_{2}(7)$	$_{\text{(NH2cycl)}}$N1-H6···**O1**$_{\text{(COO-)}}$ & $_{\text{(CHcycl)}}$C2-H1···**O2**$_{\text{(COO-)}}$
	$R^{3}_{4}(14)$	*($_{\text{(NH2cycl)}}$N1-H6···O2$_{\text{(COO-)}}$)$_2$ & ($_{\text{(NH2cycl)}}$N1-H7···O2$_{\text{(COO-)}}$)$_2$<br>*($_{\text{(NH2cycl)}}$N1-H7···**O2**$_{\text{(COO-)}}$)$_2$ & ($_{\text{(CHcycl)}}$C2-H1···**O2**$_{\text{(COO-)}}$)$_2$
	$R^{4}_{4}(14)$	($_{\text{(NH2cycl)}}$N1-H7···**O2**$_{\text{(COO-)}}$)$_2$ & ($_{\text{(NH2cycl)}}$N1-H6···**O1**$_{\text{(COO-)}}$)$_2$
**QANRUT**	C(4)	$_{\text{(CH)}}$C2-H3···O1$_{\text{(C=O_COO-)}}$
	C(5)	*$_{\text{(NH2+)}}$N1-H1···O1$_{\text{(C=O_COOH)}}$

		*$_{(CH2)}$C3-H4···O2$_{(C=O_COO-)}$
	$C(6)$	$_{(CH2)}$C5-H8···O2$_{(C=O_COO-)}$
	$C^{1}_{2}(5)$	*$_{(NH2)}$N1-H1···O1$_{(C=O_COO-)}$ & $_{(CH)}$C2-H3···O1$_{(C=O_COO-)}$<br>*$_{(NH2)}$N1-H1···O1$_{(C=O_COO-)}$ & $_{(CH)}$C2-H3···O1$_{(C=O_COO-)}$
	$C^{1}_{2}(6)$	*$_{(CH2)}$C3-H4···O2$_{(C=O_COO-)}$ & $_{(CH2)}$C5-H8···O2$_{(COO-)}$
	$C^{2}_{2}(7)$	*$_{(NH2)}$N1-H1···O1$_{(C=O_COO-)}$ & $_{(CH2)}$C5-H8···O2$_{(COO-)}$<br>*$_{(NH2)}$N1-H1···O1$_{(C=O_COO-)}$ & $_{(CH2)}$C5-H8···O2$_{(COO-)}$<br>*$_{(CH2)}$C3-H4···O2$_{(C=O_COO-)}$ & $_{(CH)}$C2-H3···O1$_{(C=O_COO-)}$
	$C^{2}_{2}(9)$	$_{(NH2)}$N1-H1···O1$_{(C=O_COO-)}$ & $_{(CH)}$C2-H3···O1$_{(C=O_COO-)}$<br>*$_{(NH2)}$N1-H1···O1$_{(C=O_COO-)}$ & $_{(CH)}$C2-H3···O1$_{(C=O_COO-)}$<br>*$_{(CH2)}$C3-H4···O2$_{(C=O_COO-)}$ & $_{(CH)}$C2-H3···O1$_{(C=O_COO-)}$
	$C^{2}_{2}(10)$	$_{(NH2)}$N1-H1···O1$_{(C=O_COO-)}$ & $_{(CH2)}$C3-H4···O2$_{(COO-)}$
	$C^{2}_{2}(11)$	*$_{(NH2)}$N1-H1···O1$_{(C=O_COO-)}$ & $_{(CH2)}$C5-H8···O2$_{(COO-)}$<br>*$_{(NH2)}$N1-H1···O1$_{(C=O_COO-)}$ & $_{(CH2)}$C5-H8···O2$_{(COO-)}$<br>*$_{(CH2)}$C3-H4···O2$_{(COO-)}$ & $_{(CH2)}$C5-H8···O2$_{(COO-)}$
	$C^{3}_{4}(14)$	*$_{[(NH2)}$N1-H1···O1$_{(C=O_COO-)]2}$ & $_{[(NH2)}$N1-H2···O1$_{(C=O_COO-)]2}$<br>*$_{(NH2)}$N1-H1···O1$_{(C=O_COO-)}$ & $_{(CH)}$C2-H3···O1$_{(C=O_COO-)}$
	$C^{3}_{4}(17)$	$_{[(CH2)}$C3-H4···O2$_{(COO-)]2}$ & $_{[(CH2)}$C5-H8···O2$_{(COO-)]2}$
	$C^{4}_{4}(18)$	*$_{[(NH2)}$N1-H1···O1$_{(C=O_COO-)]2}$ & $_{[(NH2)}$N1-H2···O1$_{(C=O_COO-)]2}$<br>*$_{[(NH2)}$N1-H1···O1$_{(C=O_COO-)]2}$ & $_{[(CH2)}$C3-H4···O2$_{(COO-)]2}$<br>*$_{(NH2)}$N1-H1···O1$_{(C=O_COO-)}$ & $_{(CH2)}$C5-H8···O2$_{(COO-)}$<br>*$_{(CH)}$C2-H3···O1$_{(C=O_COO-)}$ & $_{(CH2)}$C5-H8···O2$_{(COO-)}$
	$R^{2}_{2}(8)$	$_{(NH2)}$N1-H1···O1$_{(C=O_COO-)}$ & $_{(CH2)}$C3-H4···O2$_{(COO-)}$
	$R^{2}_{2}(10)$	$_{[(NH2+)}$N1-H1···O1$_{(C=O_COOH)]2}$
	$R^{2}_{3}(9)$	$_{(NH2)}$N1-H1···O1$_{(C=O_COO-)}$ & $_{[(CH)}$C2-H3···O1$_{(C=O_COO-)]2}$
	$R^{2}_{4}(8)$	$_{[(NH2)}$N1-H1···O1$_{(C=O_COO-)]2}$ & $_{[(NH2)}$N1-H2···O1$_{(C=O_COO-)]2}$
	$R^{3}_{3}(11)$	*$_{(CH2)}$C3-H4···O2$_{(C=O_COO-)}$ & $_{(CH)}$C2-H3···O1$_{(C=O_COO-)}$
	$R^{3}_{4}(14)$	*$_{[(NH2)}$N1-H1···O1$_{(C=O_COO-)]2}$ & $_{[(NH2)}$N1-H2···O1$_{(C=O_COO-)]2}$<br>*$_{[(NH2)}$N1-H1···O1$_{(C=O_COO-)]2}$ & $_{[(CH)}$C2-H3···O1$_{(C=O_COO-)]2}$
	$R^{3}_{4}(17)$	*$_{(CH2)}$C3-H4···O2$_{(C=O_COO-)}$ & $_{[(CH2)}$C5-H8···O2$_{(COO-)]2}$
	$R^{4}_{4}(16)$	*$_{(CH)}$C2-H3···O1$_{(C=O_COO-)}$ & $_{[(CH2)}$C3-H4···O2$_{(COO-)]2}$<br>*$_{(CH)}$C2-H3···O1$_{(C=O_COO-)}$ & $_{(CH2)}$C5-H8···O2$_{(COO-)}$
	$R^{4}_{4}(18)$	$_{[(NH2)}$N1-H1···O1$_{(C=O_COO-)]2}$ & $_{[(CH2)}$C5-H8···O2$_{(COO-)]2}$<br>*$_{[(NH2)}$N1-H1···O1$_{(C=O_COO-)]2}$ & $_{[(CH2)}$C3-H4···O2$_{(COO-)]2}$
	$R^{4}_{4}(16)$	$_{[(NH2)}$N1-H1···O1$_{(C=O_COO-)]2}$ & $_{[(CH2)}$C3-H4···O2$_{(COO-)]2}$
	$R^{4}_{4}(20)$	*$_{[(NH2)}$N1-H1···O1$_{(C=O_COO-)]2}$ & $_{[(NH2)}$N1-H2···O1$_{(C=O_COO-)]2}$<br>*$_{[(NH2)}$N1-H1···O1$_{(C=O_COO-)]2}$ & $_{[(CH2)}$C3-H4···O2$_{(COO-)]2}$

		*(CH)C2-H3···O1(C=O_COO-) & (CH2)C5-H8···O2(COO-)
	$R^4_6(18)$	[(NH2)N1-H1···O1(C=O_COO-)]4 & [(NH2)N1-H2···O1(C=O_COO-)]2<br>*(NH2)N1-H1···O1(C=O_COO-) & (CH)C2-H3···O1(C=O_COO-)
	$R^4_5(19)$	[(NH2)N1-H1···O1(C=O_COO-)]3 & [(CH)C2-H3···O1(C=O_COO-)]2
PERINDOPRILAT structures		
**BECWIR**	*C*(11)	*(OH-COOH)**O5**-H6···**O2**(COO-)<br>*(OH-COOH)**O10**-H39···**O7**(COO-)
	D(2)	*(NH2+)**N2**-H1···O11(C=O)<br>*(NH2+)**N2**-H1···**O8**(COO-)<br>*(NH2+)**N4**-H33···**O3**(COO-)<br>*(CH2cycl)C5-H11···**O9**(C=O-COOH)<br>*(CH2)C12-H20···O11(C=O)<br>*(CH2)C13-H22···**O8**(COO-)<br>*(CH2)C29-H47···**O2**(COO-)<br>*(CH2)C29-H47···**O3**(COO-)<br>*(CH2cycl)C21-H35···**O5**(OH-COOH)<br>*(CH3)C35-H58···**O8**(COO-)<br>*(CH3)C35-H59···**O3**(COO-)<br>*(CH3)C36-H61···**O7**(COO-)
level 2	$D^1_2(3)$	(NH2+)**N2**-H1···O11(C=O) & (NH2+)**N4**-H36···O11(C=O)
	$D^2_2(5)$	*(NH2+)**N2**-H1···O11(C=O) & (NH2+)**N2**-H3···**O8**(COO-)<br>*(NH2+)**N4**-H33···**O3**(COO-) & (NH2+)**N4**-H36···O11(C=O)
	$D^2_2(7)$	*(NH2+)**N2**-H1···O11(C=O) & (NH2+)**N4**-H33···**O3**(COO-)<br>*(NH2+)**N2**-H3···**O8**(COO-) & (NH2+)**N4**-H36···O11(C=O)
	$D^2_2(12)$	(NH2+)**N2**-H1···O11(C=O) & (CH2cycl)C5-H11···**O9**(C=O-COOH)
	$D^3_3(15)$	((NH2+)**N2**-H1···O11(C=O))2 & (CH2cycl)C5-H11···**O9**(C=O-COOH)
	$D^3_3(16)$	*(OH-COOH)O5-H6···**O2**(COO-) & ((NH2+)**N2**-H1···O11(C=O))2<br>*(OH-COOH)O10-H39···**O7**(COO-) & ((NH2+)**N2**-H3···**O8**(COO-))2<br>*(OH-COOH)**O10**-H39···**O7**(COO-) & ((NH2+)**N4**-H33···**O3**(COO-))2<br>*((NH2+)**N2**-H1···O11(C=O))2 & (OH-COOH)**O5**-H6···**O2**(COO-)<br>*((NH2+)**N2**-H3···**O8**(COO-))2 & (OH-COOH)**O5**-H6···**O2**(COO-)<br>*((NH2+)**N4**-H33···**O3**(COO-))2 & (OH-COOH)**O5**-H6···**O2**(COO-)<br>*((NH2+)**N4**-H36···O11(C=O))2 & (NH2+)**N4**-H36···O11(C=O)
	C(4)	*(CHcycl)C1-H2···**O4**(C=O-COOH)<br>*(CHcycl)C18-H29···**O9**(C=O-COOH)
	C(6)	*(CHcycl)C3-H7···**O4**(C=O-COOH)<br>*(CHcycl)C20-H32···**O9**(C=O-COOH)

	$C^{2}_{2}(9)$	(NH2+)N2-H1···O11(C=O) & (CH3)C35-H59···O3(COO-)
	$C^{2}_{2}(10)$	*(NH2+)N2-H3···O8(COO-) & (CH2)C29-H47···O2(COO-)<br>*(NH2+)N2-H3···O8(COO-) & (NH2+)N4-H33···O3(COO-)<br>*(NH2+)N2-H3···O8(COO-) & (CH2)C29-H47···O3(COO-)<br>*(CHcycl)C1-H2···O4(C=O-COOH) & (CHcycl)C3-H7···O4(C=O-COOH)
	$C^{2}_{2}(14)$	(CHcycl)C1-H2···O4(C=O-COOH) & (CH2)C12-H19···O5(OH-COOH)
	$C^{2}_{2}(15)$	*(OH-COOH)O5-H6···O2(COO-) & (CHcycl)C1-H2···O4(C=O-COOH)<br>*((CH2cycl)C5-H11···O9)2 & (CH2cycl)C21-H35···O5(OH-COOH)
	$C^{2}_{2}(16)$	(CHcycl)C3-H7···O4(C=O-COOH) & (CH2)C12-H19···O5(OH-COOH)
	$C^{2}_{2}(17)$	(OH-COOH)O5-H6···O2(COO-) & (CHcycl)C3-H7···O4(C=O-COOH)
	$C^{2}_{2}(19)$	(NH2+)N2-H3···O8(COO-) & (CH2cycl)C21-H35···O5(OH-COOH)
	$C^{2}_{2}(20)$	(NH2+)N2-H3···O8(COO-) & (CH2cycl)C5-H11···O9(C=O-COOH)
	$R^{1}_{2}(6)$	*(NH2+)N2-H1···O11(C=O) & (CH2)C12-H20···O11(C=O)<br>*(CHcycl)C1-H2···O4(C=O-COOH) & (CHcycl)C3-H7···O4(C=O-COOH)
	$R^{1}_{2}(7)$	(NH2+)N2-H3···O8(COO-) & (CH2)C13-H22···O8(COO-)
	$R^{2}_{2}(7)$	(OH-COOH)O5-H6···O2(COO-) & (CH2)C12-H19···O5(OH-COOH)
	$R^{2}_{2}(12)$	(CHcycl)C1-H2···O4(C=O-COOH) & (CH2)C12-H19···O5(OH-COOH)
	$R^{2}_{2}(13)$	*(OH-COOH)O5-H6···O2(COO-) & (CHcycl)C1-H2···O4(C=O-COOH)<br>*(CHcycl)C3-H7···O4(C=O-COOH) & (CH2)C12-H19···O5(OH-COOH)
	$R^{2}_{2}(14)$	(OH-COOH)O5-H6···O2(COO-) & (CHcycl)C3-H7···O4(C=O-COOH)
	$R^{2}_{2}(20)$	(NH2+)N4-H33···O3(COO-) & (CH2cycl)C5-H11···O9(C=O-COOH)
	$D^{1}_{2}(3)$	(NH2+)N2-H3···O8(COO-) & (CH3)C35-H58···O8(COO-)
	$D^{2}_{2}(5)$	(NH2+)N2-H3···O8(COO-) & (CH3)C36-H61···O7(COO-)
	$D^{2}_{2}(6)$	*(cycl)N1-H1···O11(C=O) & (CH3)C35-H58···O8(COO-)<br>*(cycl)N1-H1···O11(C=O) & (CH3)C36-H61···O7(COO-)
	$D^{2}_{2}(7)$	*(cycl)N1-H1···O11(C=O) & (CH2)C29-H47···O2(COO-)<br>*(cycl)N1-H1···O11(C=O) & (CH2)C29-H47···O3(COO-)<br>*(NH2+)N2-H3···O8(COO-) & (CH2)C12-H20···O11(C=O)<br>*(NH2+)N2-H3···O8(COO-) & (CH3)C35-H59···O3(COO-)
	$D^{2}_{2}(8)$	(NH2+)N2-H1···O11(C=O) & (CH2)C13-H22···O8(COO-)
	$D^{2}_{2}(10)$	*(NH2+)N2-H1···O11(C=O) & (CH2cycl)C21-H35···O5(OH-COOH)<br>*(NH2+)N4-H36···O11(C=O) & (CH2cycl)C5-H11···O9(C=O-COOH)
	$D^{2}_{2}(12)$	*(CH2cycl)C5-H11···O9(C=O-COOH) & (CH3)C35-H58···O8(COO-)<br>*(CH2cycl)C5-H11···O9(C=O-COOH) & (CH3)C36-H61···O7(COO-)
	$D^{2}_{2}(14)$	(CH2cycl)C5-H11···O9(C=O-COOH) & (CH2)C12-H20···O11(C=O)<br>(CH2cycl)C5-H11···O9(C=O-COOH) & (CH3)C35-H59···O3(COO-)
	$D^{2}_{3}(7)$	((CH2cycl)C5-H11···O9(C=O-COOH))2 & (CHcycl)C18-H23···O9(C=O-COOH)

	$D^{2}_{3}(13)$	$_{(CH2)}$C12-H19···O5$_{(OH-COOH)}$ & ($_{(CH2cycl)}$C21-H35···O5$_{(OH-COOH)}$)$_2$
	$D^{3}_{3}(9)$	*($_{(CH2cycl)}$C5-H11···O9$_{(C=O-COOH)}$)$_2$ & $_{(CHcycl)}$C20-H37···O9$_{(C=O-COOH)}$<br>*($_{(CH2cycl)}$C21-H35···O5$_{(OH-COOH)}$)$_2$
	$D^{3}_{3}(11)$	$_{(CHcycl)}$C3-H7···O4$_{(C=O-COOH)}$ & ($_{(CH2cycl)}$C21-H35···O5$_{(OH-COOH)}$)$_2$
	$D^{3}_{3}(14)$	*$_{(OH-COOH)}$O5-H6···O2$_{(COO-)}$ & ($_{(CH2cycl)}$C21-H35···O5$_{(OH-COOH)}$)$_2$<br>*$_{(OH-COOH)}$O5-H6···O2$_{(COO-)}$ & ($_{(CH2)}$C29-H47···O2$_{(COO-)}$)$_2$<br>*$_{(OH-COOH)}$O5-H6···O2$_{(COO-)}$ & ($_{(CH3)}$C35-H59···O3$_{(COO-)}$)$_2$
	$D^{3}_{3}(15)$	*($_{(NH2+)}$N2-H3···O8$_{(COO-)}$)$_2$ & $_{(CH2)}$C12-H19···O5$_{(OH-COOH)}$<br>*$_{(CHcycl)}$C3-H7···O4$_{(C=O-COOH)}$ & ($_{(acycl)}$C15-H11···O9$_{(C=O-COOH)}$)$_2$<br>*$_{(CH2)}$C12-H19···O5$_{(OH-COOH)}$ & ($_{(CH2)}$C12-H20···O11$_{(C=O)}$)$_2$
	$D^{3}_{3}(16)$	*$_{(OH-COOH)}$O5-H6···O2$_{(COO-)}$ & ($_{(CH2)}$C29-H47···O3$_{(COO-)}$)$_2$<br>*$_{(CH2cycl)}$C5-H11···O9$_{(C=O-COOH)}$
	$D^{3}_{3}(17)$	*($_{(NH2+)}$N2-H1···O11$_{(C=O)}$)$_2$ & $_{(CHcycl)}$C1-H2···O4$_{(C=O-COOH)}$<br>*($_{(NH2+)}$N2-H3···O8$_{(COO-)}$)$_2$ & $_{(CHcycl)}$C1-H2···O4$_{(C=O-COOH)}$<br>*$_{(NH2+)}$N4-H33···O3$_{(COO-)}$ & $_{(CH2)}$C12-H19···O5$_{(OH-COOH)}$<br>*$_{(CHcycl)}$C1-H2···O4$_{(C=O-COOH)}$ & ($_{(CH2cycl)}$C5-H11···O9$_{(C=O-COOH)}$)$_2$<br>*$_{(CH2)}$C12-H19···O5$_{(OH-COOH)}$ & ($_{(CH2)}$C13-H22···O8$_{(COO-)}$)$_2$<br>*($_{(CH2)}$C29-H47···O2$_{(COO-)}$)$_3$
	$D^{3}_{3}(18)$	*$_{(OH-COOH)}$O5-H6···O2$_{(COO-)}$ & ($_{(CH2)}$C12-H20···O11$_{(C=O)}$)$_2$<br>*($_{(NH2+)}$N2-H1···O11$_{(C=O)}$)$_2$ & $_{(CHcycl)}$C3-H7···O4$_{(C=O-COOH)}$<br>*($_{(NH2+)}$N2-H3···O8$_{(COO-)}$)$_2$ & $_{(CHcycl)}$C3-H7···O4$_{(C=O-COOH)}$
	$D^{3}_{3}(20)$	$_{(OH-COOH)}$O5-H6···O2$_{(COO-)}$ & ($_{(CH2)}$C13-H22···O8$_{(COO-)}$)$_2$
**FEFKEI**	$S(6)$	$_{(CH)}$C10-H17···O2$_{(COO-)}$
	$C(4)$	$_{(CHcycl)}$C1-H1···O4$_{(C=O_COOH)}$
	$C(5)$	$_{(NH2+)}$N2-H2···O2$_{(COO-)}$
	$C(6)$	$_{(CH2cycl)}$C2-H4···O1$_{(C=O)}$
	$C(7)$	$_{(CH2cycl)}$C4-H7···O1$_{(C=O)}$
	$C(8)$	$_{(CH2cycl)}$C6-H13···O5$_{(OH_COOH)}$
	$C(9)$	$_{(CH)}$C11-H18···O5$_{(OH_COOH)}$
	$C(11)$	$_{(CH2cycl)}$C5-H11···O3$_{(COO-)}$
	$D(2)$	*$_{(OH2)}$O6-H29···O3$_{(COO-)}$<br>*$_{(OH2)}$O6-H30···O1$_{(C=O)}$<br>*$_{(NH2+)}$N2-H3···O6$_{(OH2)}$<br>*$_{(CH3)}$C16-H26···O6$_{(OH2)}$
level 2	$C^{1}_{2}(6)$	$_{(CH2cycl)}$C2-H4···O1$_{(C=O)}$ & $_{(CH2cycl)}$C4-H7···O1$_{(C=O)}$
	$C^{1}_{2}(11)$	$_{(CH2cycl)}$C6-H13···O5$_{(OH_COOH)}$ & $_{(CH)}$C11-H18···O5$_{(OH_COOH)}$

	$C^2_2(7)$	(OH2)O6-H29···O3(COO-) & (NH2+)N2-H3···O6(OH2)
	$C^2_2(9)$	(OH2)O6-H29···O3(COO-) & (CH3)C16-H26···O6(OH2)
	$C^2_2(10)$	*(OH2)O6-H29···O3(COO-) & (OH2)O6-H30···O1(C=O)<br>*(CHcycl)C1-H1···O4(C=O_COOH) & (CH2cycl)C2-H4···O1(C=O)<br>*(CHcycl)C1-H1···O4(C=O_COOH) & (CH2cycl)C6-H13···O5(OH_COOH)
	$C^2_2(11)$	(CH2cycl)C4-H7···O1(C=O) & (CH2cycl)C6-H13···O5(OH_COOH)
	$C^2_2(12)$	*(OH_COOH)O5-H11···O3(COO-) & (NH2+)N2-H2···O2(COO-)<br>*(NH2+)N2-H2···O2(COO-) & (CH)C11-H18···O5(OH_COOH)<br>*(CHcycl)C1-H1···O4(C=O_COOH) & (CH2cycl)C4-H7···O1(C=O)<br>*(CH2cycl)C2-H4···O1(C=O) & (CH2cycl)C6-H13···O5(OH_COOH)
	$C^2_2(13)$	*(OH_COOH)O5-H11···O3(COO-) & (CH2cycl)C6-H13···O5(OH_COOH)<br>*(CHcycl)C1-H1···O4(C=O_COOH) & (CH)C11-H18···O5(OH_COOH)<br>*(CH2cycl)C2-H4···O1(C=O) & (CH2cycl)C4-H7···O1(C=O)<br>*(CH2cycl)C4-H7···O1(C=O) & (CH)C11-H18···O5(OH_COOH)
	$C^2_2(14)$	*(NH2+)N2-H2···O2(COO-) & (CH)C11-H18···O5(OH_COOH)<br>*(CH2cycl)C4-H7···O1(C=O) & (CH2cycl)C6-H13···O5(OH_COOH)
	$C^2_2(15)$	*(OH_COOH)O5-H11···O3(COO-) & (CHcycl)C1-H1···O4(C=O_COOH)<br>*(OH_COOH)O5-H11···O3(COO-) & (CH2cycl)C4-H7···O1(C=O)<br>*(NH2+)N2-H2···O2(COO-) & (CH2cycl)C2-H4···O1(C=O)<br>*(CH2cycl)C2-H4···O1(C=O) & (CH)C11-H18···O5(OH_COOH)
	$C^2_2(16)$	*(OH_COOH)O5-H11···O3(COO-) & (NH2+)N2-H2···O2(COO-)<br>*(NH2+)N2-H2···O2(COO-) & (CH2cycl)C4-H7···O1(C=O)<br>*(CH2cycl)C4-H7···O1(C=O) & (CH)C11-H18···O5(OH_COOH)
	$C^2_2(17)$	*(OH_COOH)O5-H11···O3(COO-) & (CH2cycl)C2-H4···O1(C=O)<br>*(cycl)N1-H2···O2(COO-) & (CHcycl)C1-H1···O4(C=O_COOH)
	$C^2_2(18)$	(OH_COOH)O5-H11···O3(COO-) & (CH2cycl)C4-H7···O1(C=O)
	$C^2_2(19)$	(NH2+)N2-H2···O2(COO-) & (CH2cycl)C6-H13···O5(OH_COOH)
	$C^2_2(20)$	(OH_COOH)O5-H11···O3(COO-) & (CH)C11-H18···O5(OH_COOH)
	$C^3_4(19)$	((CH2cycl)C2-H4···O1)$_2$ & (CH2cycl)C4-H7···O1(C=O)
	$R^1_2(6)$	(NH2+)N2-H3···O6(OH2) & (CH3)C16-H26···O6(OH2)
	$R^2_2(6)$	(OH_COOH)O5-H11···O3(COO-) & (CH)C11-H18···O5(OH_COOH)
	$R^2_2(7)$	*(OH2)O6-H30···O1(C=O) & (NH2+)N2-H3···O6(OH2)<br>*(OH2)O6-H30···O1(C=O) & (CH3)C16-H26···O6(OH2)
	$R^2_2(10)$	(CHcycl)C1-H1···O4(C=O_COOH) & (CH2cycl)C2-H4···O1(C=O)
	$R^2_2(11)$	*(CHcycl)C1-H1···O4(C=O_COOH) & (CH)C11-H18···O5(OH_COOH)<br>*(CH2cycl)C2-H4···O1(C=O) & (CH)C11-H18···O5(OH_COOH)
	$R^2_2(12)$	(CHcycl)C1-H1···O4(C=O_COOH) & (CH2cycl)C6-H13···O5(OH_COOH)

	$R^2_2(13)$	*$_{(OH_COOH)}$O5-H11···O3$_{(COO-)}$ & $_{(CHcycl)}$C1-H1···O4$_{(C=O_COOH)}$<br>*$_{(OH_COOH)}$O5-H11···O3$_{(COO-)}$ & $_{(CH2cycl)}$C2-H4···O1$_{(C=O)}$<br>*$_{(CH2cycl)}$C2-H4···O1$_{(C=O)}$ & $_{(CH2cycl)}$C6-H13···O5$_{(OH_COOH)}$
	$R^2_2(17)$	$_{(CH2cycl)}$C6-H13···O5$_{(OH_COOH)}$ & $_{(CH)}$C11-H18···O5$_{(OH_COOH)}$
	$R^2_2(19)$	$_{(OH_COOH)}$O5-H11···O3$_{(COO-)}$ & $_{(CH2cycl)}$C6-H13···O5$_{(OH_COOH)}$
	$R^3_4(19)$	($_{(CH2cycl)}$C2-H4···O1$_{(C=O)}$)$_2$ & $_{(CH2cycl)}$C4-H7···O1$_{(C=O)}$
	$D^2_3(9)$	($_{(OH2)}$O6-H30···O1$_{(C=O)}$)$_2$ & $_{(CH2cycl)}$C2-H4···O1$_{(C=O)}$
	$D^2_3(10)$	($_{(OH2)}$O6-H30···O1$_{(C=O)}$)$_2$ & $_{(CH2cycl)}$C4-H7···O1$_{(C=O)}$
	$D^2_3(14)$	$_{(OH_COOH)}$O5-H11···O3$_{(COO-)}$ & ($_{(OH2)}$O6-H29···O3$_{(COO-)}$)$_2$
	$D^3_3(10)$	*($_{(OH2)}$O6-H29···O3$_{(COO-)}$)$_2$ & $_{(NH2+)}$N2-H2···O2$_{(COO-)}$<br>*$_{(NH2+)}$N2-H2···O2$_{(COO-)}$ & ($_{(NH2+)}$N2-H3···O6$_{(OH2)}$)$_2$
	$D^3_3(13)$	$_{(CHcycl)}$C1-H1···O4$_{(C=O_COOH)}$ & ($_{(OH2)}$O6-H30···O1$_{(C=O)}$)$_2$
	$D^3_3(14)$	*($_{(OH2)}$O6-H30···O1$_{(C=O)}$)$_2$ & $_{(NH2+)}$N2-H2···O2$_{(COO-)}$<br>*($_{(OH2)}$O6-H30···O1$_{(C=O)}$)$_2$ & $_{(CH)}$C11-H18···O5$_{(OH_COOH)}$<br>*$_{(NH2+)}$N2-H2···O2$_{(COO-)}$ & ($_{(CH3)}$C16-H26···O6$_{(OH2)}$)$_2$<br>*($_{(NH2+)}$N2-H3···O6$_{(OH2)}$)$_2$ & $_{(CH)}$C11-H18···O5$_{(OH_COOH)}$
	$D^3_3(15)$	*($_{(OH2)}$O6-H30···O1$_{(C=O)}$)$_2$ & $_{(CH2cycl)}$C6-H13···O5$_{(OH_COOH)}$<br>*($_{(NH2+)}$N2-H3···O6$_{(OH2)}$)$_2$ & $_{(CH2cycl)}$C2-H4···O1$_{(C=O)}$<br>*$_{(CH2cycl)}$C2-H4···O1$_{(C=O)}$ & ($_{(CH3)}$C16-H26···O6$_{(OH2)}$)$_2$
	$D^3_3(16)$	*$_{(OH_COOH)}$O5-H11···O3$_{(COO-)}$ & ($_{(NH2+)}$N2-H3···O6$_{(OH2)}$)$_2$<br>*$_{(OH_COOH)}$O5-H11···O3$_{(COO-)}$ & ($_{(OH2)}$O6-H30···O1$_{(C=O)}$)$_2$<br>*($_{(OH2)}$O6-H29···O3$_{(COO-)}$)$_2$ & $_{(CH)}$C11-H18···O5$_{(OH_COOH)}$<br>*($_{(NH2+)}$N2-H3···O6$_{(OH2)}$)$_2$ & $_{(CH2cycl)}$C4-H7···O1$_{(C=O)}$<br>*$_{(CH2cycl)}$C4-H7···O1$_{(C=O)}$ & ($_{(CH3)}$C16-H26···O6$_{(OH2)}$)$_2$<br>*$_{(CH)}$C11-H18···O5$_{(OH_COOH)}$ & ($_{(CH3)}$C16-H26···O6$_{(OH2)}$)$_2$
	$D^3_3(17)$	*($_{(NH2+)}$N2-H3···O6)$_2$ & $_{(CHcycl)}$C1-H1···O4$_{(C=O_COOH)}$<br>*$_{(CHcycl)}$C1-H1···O4$_{(C=O_COOH)}$ & ($_{(CH3)}$C16-H26···O6$_{(OH2)}$)$_2$
	$D^3_3(18)$	$_{(OH_COOH)}$O5-H11···O3$_{(COO-)}$ & ($_{(CH3)}$C16-H26···O6$_{(OH2)}$)$_2$
	$D^3_3(19)$	*($_{(OH2)}$O6-H29···O3$_{(COO-)}$)$_2$ & $_{(CH2cycl)}$C2-H4···O1$_{(C=O)}$<br>*($_{(NH2+)}$N2-H3···O6$_{(OH2)}$)$_2$ & $_{(CH2cycl)}$C6-H13···O5$_{(OH_COOH)}$<br>*$_{(CH2cycl)}$C6-H13···O5$_{(OH_COOH)}$ & ($_{(CH3)}$C16-H26···O6$_{(OH2)}$)$_2$
	$D^3_3(20)$	($_{(OH2)}$O6-H29···O3$_{(COO-)}$)$_2$ & $_{(CH2cycl)}$C4-H7···O1$_{(C=O)}$
DKP PERINDOPRIL structures		
**BILNAN**	*D*(2)	$_{(CHcycl)}$C1-H1···O8$_{(C=O)}$<br>$_{(CHcycl)}$C10-H14···O8$_{(C=O)}$<br>$_{(CH)}$C11-H15···O6$_{(C=O)}$

		(CHcycl)C20-H31···O2(C=O)<br>(CH2cycl)C24-H38···O1(C=O)<br>(CHcycl)C29-H44···O4(C=O)<br>(CH)C30-H45···O2(C=O)
	C(10)	(CH3)C38-H59···O5(C=O)
	$C^1_2(7)$	(CHcycl)C20-H31···O2(C=O) & (CH)C30-H45···O2(C=O)
	$C^2_2(9)$	(CHcycl)C1-H1···O8(C=O) & (CHcycl)C29-H44···O4(C=O)
	$C^2_2(10)$	*(CHcycl)C10-H14···O8(C=O) & (CHcycl)C20-H31···O2(C=O)<br>*(CHcycl)C10-H14···O8(C=O) & (CHcycl)C29-H44···O4(C=O)
	$C^2_2(11)$	*(CHcycl)C1-H1···O8(C=O) & (CHcycl)C20-H31···O2(C=O)<br>*(CHcycl)C10-H14···O8(C=O) & (CH)C11-H15···O6(C=O)<br>*(CHcycl)C10-H14···O8(C=O) & (CH)C30-H45···O2(C=O)<br>*(CH)C11-H15···O6(C=O) & (CHcycl)C20-H31···O2(C=O)<br>*(CH)C11-H15···O6(C=O) & (CHcycl)C29-H44···O4(C=O)<br>*(CHcycl)C29-H44···O4(C=O) & (CH)C30-H45···O2(C=O)
	$C^2_2(12)$	*(CHcycl)C1-H1···O8(C=O) & (CH)C11-H15···O6(C=O)<br>*(CHcycl)C1-H1···O8(C=O) & (CH)C30-H45···O2(C=O)<br>*(CHcycl)C10-H14···O8(C=O) & (CH2cycl)C24-H38···O1(C=O)
	$C^2_2(13)$	(CHcycl)C1-H1···O8(C=O) & (CH2cycl)C24-H38···O1(C=O)
	$C^2_2(14)$	(CHcycl)C20-H31···O2(C=O) & (CH2cycl)C24-H38···O1(C=O)
	$C^2_2(15)$	(CH2cycl)C24-H38···O1(C=O) & (CHcycl)C29-H44···O4(C=O)
	$C^2_2(17)$	*(CH)C11-H15···O6(C=O) & (CH2cycl)C24-H38···O1(C=O)<br>*(CH2cycl)C4-H38···O1(C=O) & (CH)C30-H45···O2(C=O)
	$R^1_2(7)$	(CHcycl)C1-H1···O8(C=O) & (CHcycl)C10-H14···O8(C=O)
	$R^2_2(8)$	*(CH)C11-H15···O6(C=O) & (CH)C30-H45···O2(C=O)
	$R^2_2(12)$	(CHcycl)C20-H31···O2(C=O) & (CHcycl)C29-H44···O4(C=O)
	$D^3_3(15)$	*(CH)C11-H15···O6(C=O) & (CH3)C38-H59···O5(C=O)<br>*((CHcycl)C29-H44···O4(C=O))$_2$ & (CH3)C38-H59···O5(C=O)<br>*((CH)C30-H45···O2(C=O))$_2$ & (CH3)C38-H59···O5(C=O)
	$D^3_3(17)$	*(CHcycl)C1-H1···O8(C=O) & (CH3)C38-H59···O5(C=O)<br>*((CHcycl)C10-H14···O8(C=O))$_2$ & (CH3)C38-H59···O5(C=O)<br>*((CHcycl)C20-H31···O2(C=O))$_2$ & (CH3)C38-H59···O5(C=O)
PERINDOPRIL structures		
**IVEGIA**	*D*(2)	*(NH3+)N5-H63···**O5**(COO-)<br>*(NH3+)N5-H64···**O4**(COO-)<br>*(NH3+)N5-H65···**O10**(COO-)<br>*(NH3+)N6-H78···**O10**(COO-)

		*$_{(NH3+)}$N6-H79···**O9**$_{(COO-)}$<br>*$_{(NH3+)}$N6-H80···**O5**$_{(COO-)}$<br>*$_{(CH2)}$C12-H18···O12$_{(OH2)}$<br>*$_{(CH2)}$C37-H59···O2$_{(C=O)}$
	$C(6)$	*$_{(CH3)}$C19-H29···O2 $_{(C=O)}$<br>*$_{(CH3)}$C38-H60···O7$_{(C=O)}$
	$C(7)$	$_{(CH3)}$C16-H25···O3$_{(C-O-C)}$
level 2	$C^2_2(6)$	*$_{(NH3+)}$N5-H63···**O5**$_{(COO-)}$ & $_{(NH3+)}$N5-H64···**O4**$_{(COO-)}$<br>*$_{(NH3+)}$N6-H78···**O10**$_{(COO-)}$ & $_{(NH3+)}$N6-H79···**O9**$_{(COO-)}$
	$C^2_2(13)$	$_{(CH3)}$C16-H25···O3$_{(C-O-C)}$ & $_{(CH3)}$C19-H29···O2$_{(C=O)}$
	$R^2_2(11)$	$_{(CH3)}$C16-H25···O3$_{(C-O-C)}$ & $_{(CH3)}$C19-H29···O2$_{(C=O)}$
	$D^1_2(3)$	*$_{(NH3+)}$N5-H63···**O5**$_{(COO-)}$ & $_{(NH3+)}$N6-H80···**O5**$_{(COO-)}$<br>*$_{(NH3+)}$N5-H65···**O10**$_{(COO-)}$ & $_{(NH3+)}$N6-H78···**O10**$_{(COO-)}$
	$D^2_2(5)$	*$_{(NH3+)}$N5-H63···**O5**$_{(COO-)}$ & $_{(NH3+)}$N5-H65···**O10**$_{(COO-)}$<br>*$_{(NH3+)}$N5-H64···**O4**$_{(COO-)}$ & $_{(NH3+)}$N5-H65···**O10**$_{(COO-)}$<br>*$_{(NH3+)}$N5-H64···**O4**$_{(COO-)}$ & $_{(NH3+)}$N6-H80···**O5**$_{(COO-)}$<br>*$_{(NH3+)}$N5-H65···**O10**$_{(COO-)}$ & $_{(NH3+)}$N6-H79···**O9**$_{(COO-)}$<br>*$_{(NH3+)}$N6-H78···**O10**$_{(COO-)}$ & $_{(NH3+)}$N6-H80···**O5**$_{(COO-)}$<br>*$_{(NH3+)}$N6-H79···**O9**$_{(COO-)}$ & $_{(NH3+)}$N6-H80···**O5**$_{(COO-)}$
	$D^2_2(7)$	$_{(CH2)}$C12-H18···O12$_{(OH2)}$ & $_{(CH2)}$C37-H59···O2$_{(C=O)}$
	$D^2_2(12)$	*$_{(NH3+)}$N5-H63···**O5**$_{(COO-)}$ & $_{(CH2)}$C12-H18···O12$_{(OH2)}$<br>*$_{(NH3+)}$N5-H64···**O4**$_{(COO-)}$ & $_{(CH2)}$C12-H18···O12$_{(OH2)}$<br>*$_{(NH3+)}$N5-H63···**O5**$_{(COO-)}$ & $_{(CH2)}$C37-H59···O2$_{(C=O)}$<br>*$_{(NH3+)}$N5-H64···**O4**$_{(COO-)}$ & $_{(CH2)}$C37-H59···O2$_{(C=O)}$<br>*$_{(NH3+)}$N6-H80···**O5**$_{(COO-)}$ & $_{(CH2)}$C12-H18···O12$_{(OH2)}$<br>*$_{(NH3+)}$N6-H80···**O5**$_{(COO-)}$ & $_{(CH2)}$C37-H59···O2$_{(C=O)}$
	$D^2_2(14)$	$_{(NH3+)}$N5-H65···**O10**$_{(COO-)}$ & $_{(CH2)}$C37-H59···O2$_{(C=O)}$<br>*$_{(NH3+)}$N6-H78···**O10**$_{(COO-)}$ & $_{(CH2)}$C37-H59···O2$_{(C=O)}$<br>*$_{(NH3+)}$N6-H79···**O9**$_{(COO-)}$ & $_{(CH2)}$C37-H59···O2$_{(C=O)}$
	$D^2_3(9)$	$_{(CH3)}$C19-H29···O2$_{(C=O)}$ & ($_{(CH2)}$C37-H59···O2$_{(C=O)}$)$_2$
	$D^3_3(11)$	($_{(CH2)}$C37-H59···O2$_{(C=O)}$)$_2$ & $_{(CH3)}$C38-H60···O7$_{(C=O)}$
	$D^3_3(12)$	$_{(CH3)}$C16-H25···O3$_{(C-O-C)}$ & ($_{(CH2)}$C37-H59···O2$_{(C=O)}$)$_2$
	$D^3_3(14)$	($_{(CH2)}$C12-H18···O12$_{(OH2)}$)$_2$ & $_{(CH3)}$C16-H25···O3$_{(C-O-C)}$
	$D^3_3(15)$	$_{(CH2)}$C12-H18···O12$_{(OH2)}$)$_2$ & $_{(CH3)}$C19-H29···O2$_{(C=O)}$
	$D^3_3(20)$	*($_{(NH3+)}$N5-H63···**O5**$_{(COO-)}$)$_2$ & $_{(CH3)}$C16-H25···O3$_{(C-O-C)}$<br>*($_{(NH3+)}$N5-H64···**O4**$_{(COO-)}$)$_2$ & $_{(CH3)}$C16-H25···O3$_{(C-O-C)}$<br>*($_{(NH3+)}$N6-H80···**O5**$_{(COO-)}$)$_2$ & $_{(CH3)}$C16-H25···O3$_{(C-O-C)}$

**UZOVAH03**	$C(7)$	$_{(CH3)}$C26-H48···O3$_{(C-O-C)}$
	$R^2_2(12)$	$_{(CHcycl)}$C8-H6···O6$_{(C-O-C)}$
	$D(2)$	*$_{(NH3+)}$N3-H32···**O4**$_{(COO-)}$<br>*$_{(NH3+)}$N3-H33···**O5**$_{(COO-)}$<br>*$_{(NH3+)}$N3-H34···**O5**$_{(COO-)}$
level 2	$C^1_2(4)$	$_{(NH3+)}$N3-H33···**O5**$_{(COO-)}$ & $_{(NH3+)}$N3-H34···**O5**$_{(COO-)}$
	$C^2_2(6)$	*$_{(NH3+)}$N3-H32···**O4**$_{(COO-)}$ & $_{(NH3+)}$N3-H33···**O5**$_{(COO-)}$<br>*$_{(NH3+)}$N3-H32···**O4**$_{(COO-)}$ & $_{(NH3+)}$N3-H34···**O5**$_{(COO-)}$

Proline-based ACEI		
**CIYNIH**	$D(2)$	*$_{(OH2)}$O6-H25···O3$_{(C=O)}$<br>*$_{(OH2)}$O6-H26···O7$_{(OH2)}$<br>*$_{(OH2)}$O7-H28···**O5**$_{(COO-)}$<br>*$_{(NH2+)}$**N2**-H3···O6$_{(OH2)}$<br>*$_{(CH2)}$C11-H16···O6$_{(OH2)}$<br>*$_{(CH2)}$C12-H19···O6$_{(OH2)}$
	$C(5)$	$_{(NH2+)}$**N2**-H3···O6$_{(OH2)}$
	$C(9)$	$_{(CHcycl)}$C15-H21···**O5**$_{(COO-)}$
	$C(11)$	$_{(OH\_COOH)}$**O2**-H6···**O5**$_{(COO-)}$
	$C(14)$	$_{(CHcycl)}$C17-H23···**O2**$_{(OH\_COOH)}$
level 2	$C^1_2(5)$	$_{(CH2)}$C11-H16···O6$_{(OH2)}$ & $_{(CH2)}$C12-H19···O6$_{(OH2)}$
	$C^1_2(6)$	*$_{(NH2+)}$**N2**-H3···O6$_{(OH2)}$ & $_{(CH2)}$C11-H16···O6$_{(OH2)}$
	$C^1_2(16)$	$_{(OH\_COOH)}$**O2**-H6···**O5**$_{(COO-)}$ & $_{(CHcycl)}$C15-H21···**O5**$_{(COO-)}$
	$C^2_2(7)$	$_{(NH2+)}$**N2**-H3···O6$_{(OH2)}$ & $_{(OH2)}$O6-H25···O3$_{(C=O)}$
	$C^2_2(10)$	$_{(OH2)}$O6-H25···O3$_{(C=O)}$ & $_{(CH2)}$C12-H19···O6$_{(OH2)}$
	$C^2_2(11)$	$_{(OH\_COOH)}$**O2**-H6···**O5**$_{(COO-)}$ & $_{(CHcycl)}$C17-H23···**O2**$_{(OH\_COOH)}$
	$C^2_2(12)$	*$_{(OH\_COOH)}$**O2**-H6···**O5**$_{(COO-)}$ & $_{(NH2+)}$**N2**-H4···**O4**$_{(COO-)}$<br>*$_{(NH2+)}$**N2**-H4···**O4**$_{(COO-)}$ & $_{(CHcycl)}$C15-H21···**O5**$_{(COO-)}$
	$C^2_2(14)$	$_{(NH2+)}$**N2**-H4···**O4**$_{(COO-)}$ & $_{(CHcycl)}$C15-H21···**O5**$_{(COO-)}$
	$C^2_2(15)$	$_{(CHcycl)}$C15-H21···**O5**$_{(COO-)}$ & $_{(CHcycl)}$C17-H23···**O2**$_{(OH\_COOH)}$
	$C^2_2(16)$	$_{(OH\_COOH)}$**O2**-H6···**O5**$_{(COO-)}$ & $_{(NH2+)}$**N2**-H4···**O4**$_{(COO-)}$
	$C^2_2(17)$	$_{(NH2+)}$**N2**-H4···**O4**$_{(COO-)}$ & $_{(CHcycl)}$C17-H23···**O2**$_{(OH\_COOH)}$
	$C^2_2(19)$	$_{(NH2+)}$**N2**-H4···O4$_{(COO-)}$ & $_{(CHcycl)}$C17-H23···**O2**$_{(OH\_COOH)}$
	$C^2_2(20)$	$_{(OH\_COOH)}$**O2**-H6···**O5**$_{(COO-)}$ & $_{(CHcycl)}$C15-H21···**O5**$_{(COO-)}$
	$R^1_2(7)$	$_{(NH2+)}$**N2**-H3···O6$_{(OH2)}$ & $_{(CH2)}$C12-H19···O6$_{(OH2)}$
	$R^2_2(9)$	$_{(OH2)}$O6-H25···O3$_{(C=O)}$ & $_{(CH2)}$C11-H16···O6$_{(OH2)}$
	$D^2_2(4)$	*$_{(OH2)}$O6-H26···O7$_{(OH2)}$ & $_{(NH2+)}$**N2**-H3···O6$_{(OH2)}$<br>*$_{(OH2)}$O6-H26···O7$_{(OH2)}$ & ($_{(CH2)}$C11-H16···O6$_{(OH2)}$)$_2$<br>*$_{(OH2)}$O6-H26···O7$_{(OH2)}$ & $_{(CH2)}$C12-H19···O6$_{(OH2)}$<br>*$_{(OH2)}$O7-H28···**O5**$_{(COO-)}$ & $_{(OH2)}$O6-H27···O7$_{(OH2)}$
	$D^2_2(5)$	$_{(OH2)}$O6-H25···O3$_{(C=O)}$ & $_{(OH2)}$O6-H26···O7 $_{(OH2)}$
	$D^2_2(7)$	*$_{(OH2)}$O7-H28···**O5**$_{(COO-)}$ & $_{(NH2+)}$**N2**-H3···O6$_{(OH2)}$<br>*$_{(OH2)}$O7-H28···**O5**$_{(COO-)}$ & $_{(CH2)}$C11-H16···O6$_{(OH2)}$
	$D^2_2(8)$	$_{(OH2)}$O7-H28···**O5**$_{(COO-)}$ & $_{(CH2)}$C12-H19···O6$_{(OH2)}$
	$D^2_2(9)$	$_{(OH2)}$O7-H28···**O5**$_{(COO-)}$ & $_{(OH2)}$O6-H25···O3$_{(C=O)}$
	$D^2_3(14)$	$_{(OH\_COOH)}$**O2**-H6···**O5**$_{(COO-)}$ & $_{(OH2)}$O7-H28···**O5**$_{(COO-)}$
	$D^3_3(10)$	($_{(OH2)}$O7-H28···**O5**$_{(COO-)}$)$_2$ & $_{(NH2+)}$**N2**-H4···**O4**$_{(COO-)}$
	$D^3_3(12)$	*$_{(OH2)}$O7-H28···**O5**$_{(COO-)}$ & $_{(CHcycl)}$C15-H21···**O5**$_{(COO-)}$<br>*$_{(NH2+)}$**N2**-H4···**O4**$_{(COO-)}$ & $_{(CH2)}$C11-H16···O6$_{(OH2)}$
	$D^3_3(14)$	*$_{(NH2+)}$**N2**-H4···**O4**$_{(COO-)}$ & $_{(CH2)}$C12-H19···O6$_{(OH2)}$<br>*$_{(OH2)}$O6-H25···O3$_{(C=O)}$ & $_{(NH2+)}$**N2**-H4···O4$_{(COO-)}$<br>*($_{(CH2)}$C11-H16···O6)$_2$ & $_{(CHcycl)}$C15-H21···**O5**$_{(COO-)}$<br>*($_{(CH2)}$C12-H19···O6)$_2$ & $_{(CHcycl)}$C15-H21···**O5**$_{(COO-)}$

	$D^3_3(16)$	*$_{(OH_COOH)}$O2-H6···O5$_{(COO-)}$ & $(_{(NH2+)}$N2-H3···O6$_{(OH2)})_2$<br>*$_{(OH_COOH)}$O2-H6···O5$_{(COO-)}$ & $(_{(OH2)}$O6-H25···O3$_{(C=O)})_2$<br>*$(_{(NH2+)}$N2-H3···O6$_{(OH2)})_2$ & $_{(CHcycl)}$C15-H21···O5$_{(COO-)}$<br>*$(_{(NH2+)}$N2-H3···O6$_{(OH2)})_2$ & $_{(CHcycl)}$C17-H23···O2$_{(OH_COOH)}$
	$D^3_3(18)$	$_{(OH_COOH)}$O2-H6···O5$_{(COO-)}$ & $(_{(CH2)}$C11-H16···O6$_{(OH2)})_2$
	$D^3_3(19)$	*$(_{(OH2)}$O6-H25···O3$_{(C=O)})_2$ & $_{(CHcycl)}$C17-H23···O2$_{(OH_COOH)}$<br>*$_{(CH2)}$C11-H16···O6$_{(OH2)}$ & $_{(CHcycl)}$C17-H23···O2$_{(OH_COOH)}$<br>*$(_{(CH2)}$C12-H19···O6$_{(OH2)})_2$ & $_{(CHcycl)}$C17-H23···O2$_{(OH_COOH)}$
	$D^3_3(20)$	*$_{(OH_COOH)}$O2-H6···O5$_{(COO-)}$ & $(_{(CH2)}$C12-H19···O6$_{(OH2)})_2$<br>*$(_{(OH2)}$O6-H25···O3$_{(C=O)})_2$ & $_{(CHcycl)}$C15-H21···O5$_{(COO-)}$
**DIVHOF01**	$C(5)$	*$_{(CH2)}$C5-H16···O1$_{(C=O)}$<br>*$_{(CH2cycl)}$C20-H14···O3$_{(C=O)}$<br>*$_{(CH)}$C21-H26···O8$_{(COO-)}$
	$C(6)$	$_{(CH2cycl)}$C19-H12···O3$_{(C=O)}$
	$C(8)$	$_{(NH2+)}$N1-H7···O5$_{(COO-)}$
	$D(2)$	*$_{(NH2+)}$N1-H6···O7$_{(COO-)}$<br>*$_{(CH2)}$C2-H20···O9$_{(COO-)}$<br>*$_{(CH)}$C4-H15···O9$_{(COO-)}$<br>*$_{(CH2)}$C6-H19···O6$_{(COO-)}$<br>*$_{(CH2)}$C6-H19···O7$_{(COO-)}$<br>*$_{(CH3)}$C15-H23···O9$_{(COO-)}$<br>*$_{(CH2cycl)}$C19-H11···O8$_{(COO-)}$<br>*$_{(CH)}$C23-H27···O2$_{(C-O-C)}$
level 2	$C^1_2(7)$	*$_{(CH)}$C4-H15···O9$_{(COO-)}$<br>*$_{(CH2cycl)}$C20-H20···O9$_{(COO-)}$
	$C^1_2(10)$	*$_{(NH2+)}$N1-H6···O7$_{(COO-)}$ & $_{(CH)}$C4-H15···O9$_{(COO-)}$<br>*$(_{(CH2)}$C2-H20···O9$_{(COO-)})_2$ & $_{(CH3)}$C15-H23···O9$_{(COO-)}$
	$C^2_2(7)$	$_{(CH2)}$C2-H20···O9$_{(COO-)}$ & $_{(CH)}$C23-H27···O2$_{(C-O-C)}$
	$C^2_2(10)$	$_{(NH2+)}$N1-H6···O7$_{(COO-)}$ & $_{(CH)}$C23-H27···O2$_{(C-O-C)}$
	$C^2_2(11)$	*$_{(NH2+)}$N1-H6···O7$_{(COO-)}$ & $_{(CH3)}$C15-H23···O9$_{(COO-)}$<br>*$_{(NH2+)}$N1-H7···O5$_{(COO-)}$ & $_{(CH2cycl)}$C19-H12···O3$_{(C=O)}$<br>*$_{(NH2+)}$N1-H7···O5$_{(COO-)}$ & $_{(CH2cycl)}$C20-H14···O3$_{(C=O)}$<br>*$_{(CH)}$C4-H15···O9$_{(COO-)}$ & $_{(CH2)}$C6-H19···O6$_{(COO-)}$<br>*$_{(CH)}$C4-H15···O9$_{(COO-)}$ & $_{(CH2)}$C6-H19···O7$_{(COO-)}$<br>*$_{(CH2)}$C6-H19···O6$_{(COO-)}$ & $_{(CH)}$C23-H27···O2$_{(C-O-C)}$<br>*$_{(CH2)}$C6-H19···O7$_{(COO-)}$ & $_{(CH)}$C23-H27···O2$_{(C-O-C)}$<br>*$_{(CH3)}$C15-H23···O9$_{(COO-)}$ & $_{(CH2cycl)}$C19-H11···O8$_{(COO-)}$<br>*$_{(CH2cycl)}$C19-H12···O3$_{(C=O)}$ & $_{(CH2cycl)}$C20-H14···O3$_{(C=O)}$
	$C^2_2(12)$	$_{(CH)}$C4-H15···O9$_{(COO-)}$ & $_{(CH2cycl)}$C19-H11···O8$_{(COO-)}$
	$C^2_2(13)$	*$_{(NH2+)}$N1-H6···O7$_{(COO-)}$ & $_{(CH2)}$C2-H20···O9$_{(COO-)}$<br>*$_{(NH2+)}$N1-H7···O5$_{(COO-)}$ & $_{(CH2cycl)}$C20-H14···O3$_{(C=O)}$
	$C^2_2(14)$	*$_{(NH2+)}$N1-H7···O5$_{(COO-)}$ & $_{(CH2cycl)}$C19-H12···O3$_{(C=O)}$<br>*$_{(CH2)}$C2-H20···O9$_{(COO-)}$ & $_{(CH2)}$C6-H19···O6$_{(COO-)}$<br>*$_{(CH2)}$C2-H20···O9$_{(COO-)}$ & $_{(CH2)}$C6-H19···O7$_{(COO-)}$<br>*$_{(CH2)}$C5-H23···O9$_{(COO-)}$ & $_{(CH2)}$C16-H19···O7$_{(COO-)}$<br>*$(_{(CH2)}$C6-H19···O7$_{(COO-)})_2$ & $_{(CH3)}$C15-H23···O9$_{(COO-)}$<br>*$_{(CH2cycl)}$C19-H11···O8$_{(COO-)}$ & C23-H27···O2$_{(C-O-C)}$
	$C^2_2(15)$	*$_{(NH2+)(NH2+)}$N1-H7···O5$_{(COO-)}$ & $_{(CH2)}$C5-H16···O1$_{(C=O)}$<br>*$_{(CH2)}$C2-H20···O9$_{(COO-)}$ & $_{(CH2cycl)}$C19-H11···O8$_{(COO-)}$
	$C^2_2(16)$	$_{(CH2)}$C5-H16···O1$_{(C=O)}$ & $_{(CH2cycl)}$C20-H14···O3$_{(C=O)}$
	$C^2_2(17)$	$_{(CH2)}$C5-H16···O1$_{(C=O)}$ & $_{(CH2cycl)}$C19-H12···O3$_{(C=O)}$
	$R^1_2(5)$	$_{(CH2cycl)}$C19-H12···O3$_{(C=O)}$ & $_{(CH2cycl)}$C20-H14···O3$_{(C=O)}$
	$R^1_2(7)$	*$_{(NH2+)}$N1-H6···O7$_{(COO-)}$ & $_{(CH2)}$C6-H19···O7$_{(COO-)}$<br>*$_{(CH)}$C4-H15···O9$_{(COO-)}$ & $_{(CH3)}$C15-H23···O9$_{(COO-)}$
	$R^2_1(4)$	$_{(CH2)}$C6-H19···O6$_{(COO-)})$ & $_{(CH2)}$C6-H19···O7$_{(COO-)}$
	$R^2_2(8)$	*$(_{(CH)}$C4-H15···O9$_{(COO-)})_2$ & $_{(CH)}$C23-H27···O2$_{(C-O-C)}$
	$R^2_2(9)$	$_{(NH2+)}$N1-H6···O7$_{(COO-)}$ & $(_{(CH2)}$C6-H19···O6$_{(COO-)}))_2$
	$R^2_2(11)$	$_{(CH3)}$C15-H23···O9$_{(COO-)}$ & $_{(CH)}$C23-H27···O2$_{(C-O-C)}$
	$R^2_2(14)$	$_{(NH2+)}$N1-H6···O7$_{(COO-)}$ & $_{(CH2cycl)}$C19-H11···O8$_{(COO-)})$
	$R^2_2(16)$	$_{(CH2)}$C5-H16···O1$_{(C=O)}$ & $_{(CH2cycl)}$C20-H14···O3$_{(C=O)}$
	$R^2_2(17)$	*$_{(CH2)}$C5-H16···O1$_{(C=O)}$ & $_{(CH2cycl)}$C19-H12···O3$_{(C=O)}$<br>*$_{(CH2)}$C6-H19···O7$_{(COO-)}$ & $_{(CH2cycl)}$C19-H11···O8$_{(COO-)}$

	$R^{3}_{3}(19)$	*($_{(NH2+)}$N1-H7···O5$_{(COO-)}$)$_{2}$ & $_{(CH2cycl)}$C19-H12···O3$_{(C=O)}$<br>*($_{(NH2+)}$N1-H7···O5$_{(COO-)}$)$_{2}$ & $_{(CH2cycl)}$C20-H14···O3$_{(C=O)}$
	$D^{2}_{3}(8)$	($_{(CH2cycl)}$C19-H11···O8$_{(COO-)}$)$_{2}$ & $_{(CH)}$C21-H26···O8$_{(COO-)}$
	$D^{3}_{3}(10)$	*($_{(CH2)}$C2-H20···O9$_{(COO-)}$)$_{2}$ & $_{(CH)}$C21-H26···O8$_{(COO-)}$<br>*($_{(CH)}$C4-H15···O9$_{(COO-)}$)$_{2}$ & $_{(CH2)}$C5-H16···O1$_{(C=O)}$<br>*($_{(CH)}$C4-H15···O9$_{(COO-)}$))$_{2}$ & $_{(CH)}$C21-H26···O8$_{(COO-)}$<br>*$_{(CH2)}$C5-H16···O1$_{(C=O)}$ & ($_{(CH)}$C23-H27···O2$_{(C-O-C)}$)$_{2}$<br>*($_{(CH3)}$C15-H23···O9$_{(COO-)}$)$_{2}$ & $_{(CH)}$C21-H26···O8$_{(COO-)}$<br>*$_{(CH)}$C21-H26···O8$_{(COO-)}$ & ($_{(CH)}$C23-H27···O2$_{(C-O-C)}$)$_{2}$
	$D^{3}_{3}(11)$	$_{(CH2cycl)}$C19-H12···O3$_{(C=O)}$ & ($_{(CH2cycl)}$C19-H11···O8$_{(COO-)}$)$_{2}$
	$D^{3}_{3}(12)$	*($_{(NH2+)}$N1-H6···O7$_{(COO-)}$)$_{2}$ & $_{(CH2)}$C5-H16···O1$_{(C=O)}$<br>*($_{(NH2+)}$N1-H6···O7$_{(COO-)}$)$_{2}$ & $_{(CH)}$C21-H26···O8$_{(COO-)}$<br>*$_{(CH2)}$C5-H16···O1$_{(C=O)}$ & ($_{(CH2)}$C6-H19···O6$_{(COO-)}$)$_{2}$<br>*$_{(CH2)}$C5-H16···O1$_{(C=O)}$ & ($_{(CH2)}$C6-H19···O7$_{(COO-)}$)$_{2}$<br>*($_{(CH2)}$C6-H19···O6$_{(COO-)}$)$_{2}$ & $_{(CH)}$C21-H26···O8$_{(COO-)}$<br>*($_{(CH2)}$C6-H19···O7$_{(COO-)}$)$_{2}$ & $_{(CH)}$C21-H26···O8$_{(COO-)}$<br>*($_{(CH2cycl)}$C19-H11···O8$_{(COO-)}$)$_{2}$ & $_{(CH2cycl)}$C20-H14···O3$_{(C=O)}$
	$D^{3}_{3}(13)$	($_{(NH2+)}$N1-H6···O7$_{(COO-)}$)$_{2}$ & $_{(NH2+)}$N1-H7···O5$_{(COO-)}$
	$D^{3}_{3}(14)$	*($_{(NH2+)}$N1-H6···O7$_{(COO-)}$)$_{2}$ & $_{(CH2cycl)}$C20-H14···O3$_{(C=O)}$<br>*($_{(CH2)}$C2-H20···O9$_{(COO-)}$)$_{2}$ & $_{(CH2)}$C5-H16···O1$_{(C=O)}$<br>*($_{(CH3)}$C15-H23···O9$_{(COO-)}$)$_{2}$ & $_{(CH2cycl)}$C20-H14···O3$_{(C=O)}$
	$D^{3}_{3}(15)$	*($_{(NH2+)}$N1-H6···O7)$_{2}$ & $_{(CH2cycl)}$C19-H12···O3$_{(C=O)}$<br>*$_{(NH2+)}$N1-H7···O5$_{(COO-)}$ & $_{(CH)}$C4-H15···O9$_{(COO-)}$<br>*$_{(NH2+)}$N1-H7···O5$_{(COO-)}$ & $_{(CH3)}$C15-H23···O9$_{(COO-)}$<br>*($_{(CH3)}$C15-H23···O9$_{(COO-)}$)$_{2}$ & $_{(CH2cycl)}$C19-H12···O3 $_{(C=O)}$
	$D^{3}_{3}(16)$	*$_{(NH2+)}$N1-H7···O5$_{(COO-)}$ & ($_{(CH2cycl)}$C19-H11···O8$_{(COO-)}$)$_{2}$<br>*($_{(CH)}$C4-H15···O9$_{(COO-)}$)$_{2}$ & $_{(CH2cycl)}$C20-H14···O3$_{(C=O)}$<br>*$_{(CH2)}$C5-H16···O1$_{(C=O)}$ & ($_{(CH3)}$C15-H23···O9$_{(COO-)}$)$_{2}$
	$D^{3}_{3}(17)$	*$_{(NH2+)}$N1-H7···O5$_{(COO-)}$ & ($_{(CH)}$C23-H27···O2$_{(C-O-C)}$)$_{2}$<br>*($_{(CH)}$C4-H15···O9$_{(COO-)}$)$_{2}$ & $_{(CH2cycl)}$C19-H12···O3$_{(C=O)}$
	$D^{3}_{3}(18)$	$_{(CH2cycl)}$C20-H14···O3$_{(C=O)}$ & ($_{(CH)}$C23-H27···O2$_{(C-O-C)}$)$_{2}$
	$D^{3}_{3}(19)$	*$_{(NH2+)}$N1-H7···O5$_{(COO-)}$ & ($_{(CH2)}$C6-H19···O6$_{(COO-)}$)$_{2}$<br>*$_{(NH2+)}$N1-H7···O5$_{(COO-)}$ & ($_{(CH2)}$C6-H19···O7$_{(COO-)}$)$_{2}$<br>*$_{(CH2cycl)}$C19-H12···O3$_{(C=O)}$ & ($_{(CH)}$C23-H27···O2$_{(C-O-C)}$))$_{2}$
	$D^{3}_{3}(20)$	*$_{(CH2)}$C6-H19···O6$_{(COO-)}$ & $_{(CH2cycl)}$C20-H14···O3$_{(C=O)}$<br>*($_{(CH2)}$C6-H19···O7$_{(COO-)}$)$_{2}$ & $_{(CH2cycl)}$C20-H14···O3$_{(C=O)}$
**GERWUX01**		$_{(NH2+)}$N1-H2···O2$_{(COO-)}$
	$C(6)$	$_{(CH2cycl)}$C17-H25···O5$_{(COO-)}$
	$C(8)$	$_{(CH2)}$C15-H23···O3$_{(C=O)}$
	$C(12)$	*$_{(NH3+)}$N2-H3···O4$_{(COO-)}$<br>*$_{(NH3+)}$N2-H4···O5$_{(COO-)}$<br>*$_{(NH3+)}$N2-H5···O4$_{(COO-)}$
	$D(2)$	*$_{(OH2)}$O6-H32···O1$_{(COO-)}$<br>*$_{(OH2)}$O6-H33···O1$_{(COO-)}$<br>*$_{(NH2+)}$N1-H1···O6$_{(OH2)}$<br>*$_{(CH)}$C9-H15···O7$_{(OH2)}$)<br>*$_{(CH2cycl)}$C17-H26···O6$_{(OH2)}$
	$D^{3}_{3}(12)$	($_{(COO-)}$O1-H11···O6$_{(OH2)}$)$_{2}$ & $_{(CH2)}$C8-H9···O2$_{(COO-)}$
level 2	$R^{1}_{2}(6)$	*$_{(NH2+)}$N1-H12···O2$_{(COO-)}$ & $_{(CH2)}$C8-H9···O2$_{(COO-)}$<br>*$_{(CH2)}$C13-H16···O3$_{(C=O)}$ & $_{(CH2)}$C15-H21···O3$_{(C=O)}$
	$R^{2}_{2}(8)$	$_{(OH2)}$O6-H32···N1$_{(NH2+)}$ & $_{(CH2cycl)}$C17-H25···O6$_{(OH2)}$
	$R^{2}_{2}(10)$	$_{(NH3+)}$N2-H23···O4$_{(COO-)}$ & $_{(CH2)}$C15-H21···O3$_{(C=O)}$
	$R^{2}_{2}(12)$	$_{(NH3+)}$N2-H23···O4$_{(COO-)}$ & $_{(CH2)}$C13-H16···O3$_{(C=O)}$
	$R^{2}_{2}(13)$	$_{(NH2+)}$N1-H12···O2$_{(COO-)}$ & $_{(CH2)}$C13-H16···O3$_{(C=O)}$
	$R^{2}_{2}(14)$	*$_{(NH3+)}$N2-H23···O4$_{(COO-)}$ & $_{(CH2cycl)}$C17-H24···O5$_{(COO-)}$<br>*$_{(CH2)}$C13-H16···O3$_{(C=O)}$ & $_{(CH2cycl)}$C17-H24···O5$_{(COO-)}$
	$R^{2}_{2}(15)$	*$_{(NH2+)}$N1-H12···O2$_{(COO-)}$ & $_{(CH2)}$C15-H21···O3$_{(C=O)}$<br>*$_{(CH2)}$C8-H9···O2$_{(COO-)}$ & $_{(CH2)}$C13-H16···O3$_{(C=O)}$
	$R^{2}_{2}(16)$	$_{(CH2)}$C15-H21···O3$_{(C=O)}$ & $_{(CH2cycl)}$C17-H24···O5$_{(COO-)}$
	$R^{2}_{2}(17)$	*$_{(NH2+)}$N1-H12···O2$_{(COO-)}$ & $_{(CH2cycl)}$C17-H24···O5$_{(COO-)}$<br>*$_{(CH2)}$C8-H9···O2$_{(COO-)}$ & $_{(CH2)}$C15-H21···O3$_{(C=O)}$
	$R^{2}_{2}(19)$	*$_{(NH2+)}$N1-H12···O2$_{(COO-)}$ & $_{(NH3+)}$N2-H23···O4$_{(COO-)}$

		*(CH2)C8-H9···O2(COO-) & (CH2cycl)C17-H24···O5(COO-)
	$R^2_3(16)$	((NH3+)N2-H22···O4(COO-))$_2$ & (NH3+)N2-H23···O4(COO-)
	$C^1_2(4)$	(NH3+)N2-H22···O4(COO-) & (NH3+)N2-H23···O4(COO-)
	$C^1_2(11)$	(COO-)O1-H11···O6(OH2) & (CH2cycl)C17-H25···O6(OH2)
	$C^1_2(12)$	(NH3+)N2-H31···O5(COO-) & (CH2cycl)C17-H24···O5(COO-)
	$C^2_2(4)$	(COO-)O1-H11···O6(OH2) & (OH2)O6-H33···O1(COO-)
	$C^2_2(6)$	*(NH3+)N2-H22···O4(COO-) & (NH3+)N2-H31···O5(COO-)<br>*(NH3+)N2-H23···O4(COO-) & (NH3+)N2-H31···O5(COO-)
	$C^2_2(7)$	*(COO-)O1-H11···O6(OH2) & (OH2)O6-H32···N1(NH2+)<br>*(OH2)O6-H32···N1(NH2+) & (OH2)O6-H33···O1(COO-)
	$C^2_2(10)$	*(NH2+)N1-H12···O2(COO-) & (CH2)C8-H9···O2(COO-)<br>*(NH3+)N2-H22···O4(COO-) & (CH2)C15-H21···O3(C=O)<br>*(NH3+)N2-H31···O5(COO-) & (CH2)C15-H21···O3(C=O)
	$C^2_2(11)$	(OH2)O6-H33···O1(COO-) & (CH2cycl)C17-H25···O6(OH2)
	$C^2_2(12)$	*(NH3+)N2-H22···O4(COO-) & (CH2)C13-H16···O3(C=O)<br>*(NH3+)N2-H31···O5(COO-) & (CH2)C13-H16···O3(C=O)
	$C^2_2(13)$	(NH2+)N1-H12···O2(COO-) & (CH2)C13-H16···O3(C=O)
	$C^2_2(14)$	*(NH3+)N2-H22···O4(COO-) & (CH2cycl)C17-H24···O5(COO-)<br>*(CH2)C13-H16···O3(C=O) & (CH2)C15-H21···O3(C=O)<br>*(CH2cycl)C17-H24···O5(COO-) & (CH2)C13-H16···O3(C=O)
	$C^2_2(15)$	*(NH2+)N1-H12···O2(COO-) & (CH2)C15-H21···O3(C=O)<br>*(CH2)C8-H9···O2(COO-) & (CH2)C13-H16···O3(C=O)
	$C^2_2(16)$	(CH2)C15-H21···O3(C=O) & (CH2cycl)C17-H24···O5(COO-)
	$C^2_2(17)$	*(CH2)C8-H9···O2(COO-) & (CH2)C15-H21···O3(C=O)<br>*(NH2+)N1-H12···O2(COO-) & (CH2cycl)C17-H24···O5(COO-)
	$C^2_2(18)$	*(NH3+)N2-H22···O4(COO-) & (CH2)C13-H16···O3(C=O)<br>*(NH3+)N2-H22···O4(COO-) & (CH2cycl)C17-H24···O5(COO-)<br>*(NH3+)N2-H23···O4(COO-) & (CH2)C13-H16···O3(C=O)<br>*(NH3+)N2-H23···O4(COO-) & (CH2cycl)C17-H24···O5(COO-)<br>*(NH3+)N2-H31···O5(COO-) & (CH2cycl)C17-H24···O5(COO-)<br>*(NH3+)N2-H31···O5(COO-) & (CH2)C13-H16···O3(C=O)
	$C^2_2(19)$	*(NH2+)N1-H12···O2(COO-) & (NH3+)N2-H22···O4(COO-)<br>*(NH2+)N1-H12···O2(COO-) & (NH3+)N2-H23···O4(COO-)<br>*(NH2+)N1-H12···O2(COO-) & (NH3+)N2-H31···O5(COO-)<br>*(CH2)C8-H9···O2(COO-) & (CH2cycl)C17-H24···O5(COO-)
	$C^2_2(20)$	*(NH3+)N2-H22···O4(COO-) & (CH2)C15-H21···O3(C=O)<br>*(NH3+)N2-H23···O4(COO-) & (CH2)C15-H21···O3(C=O)<br>*(NH3+)N2-H31···O5(COO-) & (CH2)C15-H21···O3(C=O)
	$D^3_3(8)$	((OH2)O6-H32···N1(NH2+))$_2$ & (NH2+)N1-H12···O2(COO-)
	$D^3_3(10)$	*((OH2)O6-H32···N1(NH2+))$_2$ & (NH2+)N1-H12···O2(COO-)<br>*((OH2)O6-H32···N1(NH2+))$_2$ & (CH2)C8-H9···O2(COO-)<br>*((OH2)O6-H33···O1(COO-))$_2$ & (CH2)C8-H9···O2(COO-)
	$D^3_3(11)$	*(CH2)C13-H16···O3(C=O) & ((OH2))O6-H32···N1(NH2+))$_2$<br>*(CH2cycl)C17-H24···O5(COO-) & ((CH2cycl)C17-H25···O6(OH2))$_2$
	$D^3_3(12)$	((COO-)O1-H11···O6(OH2))$_2$ & (NH2+)N1-H12···O2(COO-)
	$D^3_3(13)$	((OH2)O6-H32···N1(NH2+))$_2$ & (CH2)C15-H21···O3(C=O)
	$D^3_3(15)$	*((OH2)O6-H32···N1(NH2+))$_2$ & (CH2cycl)C17-H24···O5(COO-)<br>*(CH2)C13-H16···O3(C=O) & ((CH2cycl)C17-H25···O6(OH2))$_2$
	$D^3_3(17)$	*((OH2)O6-H32···N1(NH2+))$_2$ & (NH3+)N2-H22···O4(COO-)<br>*((OH2)O6-H32···N1(NH2+))$_2$ & (NH3+)N2-H23···O4(COO-)<br>*((OH2)O6-H32···N1(NH2+))$_2$ & (NH3+)N2-H31···O5(COO-)<br>*((OH2)O6-H32···N1(NH2+))$_2$ & (CH2)C13-H16···O3(C=O)<br>*(CH2)C15-H21···O3(C=O) & ((CH2cycl)C17-H25···O6(OH2))$_2$
	$D^3_3(18)$	(NH2+)N1-H12···O2(COO-) & ((CH2cycl)C17-H25···O6(OH2))$_2$
	$D^3_3(19)$	*((COO-)O1-H11···O6(OH2))$_2$ & (CH2)C13-H16···O3(C=O)<br>*((OH2)O6-H33···O1(COO-))$_2$ & (CH2)C15-H21···O3(C=O)<br>*(NH3+)N2-H22···O4(COO-) & ((CH2cycl)C17-H25···O6(OH2))$_2$<br>*(NH3+)N2-H23···O4(COO-) & ((CH2cycl)C17-H25···O6(OH2))$_2$<br>*(NH3+)N2-H31···O5(COO-) & ((CH2cycl)C17-H25···O6(OH2))$_2$
	$D^3_3(20)$	(CH2)C8-H9···O2(COO-) & ((CH2cycl)C17-H25···O6(OH2))$_2$
**GERXAE**	$C(5)$	*(CH2)C8-H9···O2(C=O_COOH)<br>*(NH3+)N2-H12···O2(C=O_COOH)

	C(6)	*$_{(CH2)}$C13-H16···O3$_{(C=O)}$<br>*$_{(CH2)}$C17-H24···**O5**$_{(COO-)}$
	C(8)	$_{(CH2)}$C15-H21···O3$_{(C=O)}$
	C(12)	*$_{(NH3+)}$N2-H22···**O4**$_{(COO-)}$<br>*$_{(NH3+)}$N2-H23···**O4**$_{(COO-)}$<br>*$_{(NH3+)}$N2-H31···**O5**$_{(COO-)}$
	D(2)	*$_{(OH\_COOH)}$**O1**-H11···O6$_{(OH2)}$<br>*$_{(OH2)}$O6-H32···**N1**$_{(NH)}$<br>*$_{(OH2)}$O6-H33···**O1**$_{(OH\_COOH)}$<br>*$_{(CH2)}$C17-H25···O6$_{(OH2)}$
level 2	$R^{1}_{2}(6)$	*$_{(NH)}$**N1**-H12···**O2**$_{(C=O\_COOH)}$ & $_{(CH2)}$C8-H9···**O2**$_{(C=O\_COOH)}$<br>*$_{(CH2)}$C13-H16···O3$_{(C=O)}$ & $_{(CH2)}$C15-H21···O3$_{(C=O)}$
	$R^{2}_{2}(8)$	($_{(OH2)}$O6-H32···**N1**$_{(NH)}$))$_{2}$ & $_{(CH2)}$C17-H25···O6$_{(OH2)}$
	$R^{2}_{2}(10)$	$_{(NH3+)}$N2-H23···**O4**$_{(COO-)}$ & $_{(CH2)}$C15-H21···O3$_{(C=O)}$
	$R^{2}_{2}(12)$	$_{(NH3+)}$N2-H23···**O4**$_{(COO-)}$ & $_{(CH2)}$C13-H16···O3$_{(C=O)}$
	$R^{2}_{2}(13)$	$_{(NH)}$**N1**-H12···**O2**$_{(C=O\_COOH)}$ & $_{(CH2)}$C13-H16···O3$_{(C=O)}$
	$R^{2}_{2}(14)$	*$_{(NH3+)}$N2-H23···**O4**$_{(COO-)}$ & $_{(CH2)}$C17-H24···**O5**$_{(COO-)}$<br>*$_{(CH2)}$C13-H16···O3$_{(C=O)}$ & $_{(CH2)}$C17-H24···**O5**$_{(COO-)}$
	$R^{2}_{2}(15)$	*$_{(NH)}$**N1**-H12···**O2**$_{(C=O\_COOH)}$ & $_{(CH2)}$C15-H21···O3$_{(C=O)}$<br>*$_{(CH2)}$C8-H9···**O2**$_{(C=O\_COOH)}$ & $_{(CH2)}$C13-H16···O3$_{(C=O)}$
	$R^{2}_{2}(16)$	$_{(CH2)}$C15-H21···O3$_{(C=O)}$ & $_{(CH2)}$C17-H24···**O5**$_{(COO-)}$
	$R^{2}_{2}(17)$	*$_{(NH)}$**N1**-H12···**O2**$_{(C=O\_COOH)}$ & $_{(CH2)}$C17-H24···**O5**$_{(COO-)}$<br>*$_{(CH2)}$C8-H9···**O2**$_{(C=O\_COOH)}$ & $_{(CH2)}$C15-H21···O3$_{(C=O)}$
	$R^{2}_{2}(19)$	*$_{(NH)}$**N1**-H12···**O2**$_{(C=O\_COOH)}$ & $_{(NH3+)}$N2-H23···**O4**$_{(COO-)}$<br>*$_{(CH2)}$C8-H9···**O2**$_{(C=O\_COOH)}$ & $_{(CH2)}$C17-H24···**O5**$_{(COO-)}$
	$R^{2}_{2}(16)$	$_{(NH3+)}$N2-H22···**O4**$_{(COO-)}$ & $_{(NH3+)}$N2-H23···**O4**$_{(COO-)}$
	$C^{1}_{2}(4)$	$_{(NH3+)}$N2-H22···**O4**$_{(COO-)}$ & $_{(NH3+)}$N2-H23···**O4**$_{(COO-)}$
	$C^{1}_{2}(11)$	$_{(OH\_COOH)}$**O1**-H11···O6$_{(OH2)}$ & $_{(CH2)}$C17-H25···O6$_{(OH2)}$
	$C^{1}_{2}(12)$	$_{(NH3+)}$N2-H31···**O5**$_{(COO-)}$ & $_{(CH2)}$C17-H24···**O5**$_{(COO-)}$
	$C^{2}_{2}(4)$	$_{(OH\_COOH)}$**O1**-H11···O6$_{(OH2)}$ & $_{(OH2)}$O6-H33···**O1**$_{(OH\_COOH)}$
	$C^{2}_{2}(6)$	$_{(NH3+)}$N2-H23···**O4**$_{(COO-)}$ & $_{(NH3+)}$N2-H31···**O5**$_{(COO-)}$
	$C^{2}_{2}(7)$	*$_{(OH\_COOH)}$**O1**-H11···O6$_{(OH2)}$ & $_{(OH2)}$O6-H32···**N1**$_{(NH)}$<br>*$_{(OH2)}$O6-H32···**N1**$_{(NH)}$ & $_{(OH2)}$O6-H33···**N1**$_{(NH)}$
	$C^{2}_{2}(10)$	*$_{(NH)}$**N1**-H12···**O2**$_{(C=O\_COOH)}$ & $_{(CH2)}$C8-H9···**O2**$_{(C=O\_COOH)}$<br>*$_{(NH3+)}$N2-H22···**O4**$_{(COO-)}$ & $_{(CH2)}$C15-H21···O3$_{(C=O)}$<br>*$_{(NH3+)}$N2-H31···**O5**$_{(COO-)}$ & $_{(CH2)}$C15-H21···O3$_{(C=O)}$
	$C^{2}_{2}(11)$	$_{(OH2)}$O6-H33···**O1**$_{(OH\_COOH)}$ & $_{(CH2)}$C17-H25···O6$_{(OH2)}$
	$C^{2}_{2}(12)$	*$_{(NH3+)}$N2-H22···**O4**$_{(COO-)}$ & $_{(CH2)}$C13-H16···O3$_{(C=O)}$<br>*$_{(NH3+)}$N2-H31···**O5**$_{(COO-)}$ & $_{(CH2)}$C13-H16···O3$_{(C=O)}$
	$C^{2}_{2}(13)$	$_{(NH)}$**N1**-H12···**O2**$_{(C=O\_COOH)}$ & $_{(CH2)}$C13-H16···O3$_{(C=O)}$
	$C^{2}_{2}(14)$	*$_{(CH2)}$C13-H16···O3$_{(C=O)}$ & $_{(CH2)}$C15-H21···O3$_{(C=O)}$<br>*$_{(CH2)}$C13-H16···O3$_{(C=O)}$ & $_{(CH2)}$C17-H24···**O5**$_{(COO-)}$
	$C^{2}_{2}(15)$	*$_{(NH)}$**N1**-H12···**O2**$_{(C=O\_COOH)}$ & $_{(CH2)}$C15-H21···O3$_{(C=O)}$<br>*$_{(NH3+)}$N2-H22···**O4**$_{(COO-)}$ & $_{(CH2)}$C17-H24···**O5**$_{(COO-)}$<br>*$_{(CH2)}$C8-H9···**O2**$_{(C=O\_COOH)}$ & $_{(CH2)}$C13-H16···O3$_{(C=O)}$
	$C^{2}_{2}(16)$	$_{(CH2)}$C15-H21···O3$_{(C=O)}$ & $_{(CH2)}$C17-H24···**O5**$_{(COO-)}$
	$C^{2}_{2}(17)$	*$_{(NH)}$**N1**-H12···**O2**$_{(C=O\_COOH)}$ & $_{(CH2)}$C17-H24···**O5**$_{(COO-)}$<br>*$_{(CH2)}$C8-H9···**O2**$_{(C=O\_COOH)}$ & $_{(CH2)}$C15-H21···O3$_{(C=O)}$
	$C^{2}_{2}(18)$	*$_{(NH3+)}$N2-H22···**O4**$_{(COO-)}$ & $_{(CH2)}$C13-H16···O3$_{(C=O)}$<br>*$_{(NH3+)}$N2-H22···**O4**$_{(COO-)}$ & $_{(CH2)}$C17-H24···**O5**$_{(COO-)}$<br>*$_{(NH3+)}$N2-H23···**O4**$_{(COO-)}$ & $_{(CH2)}$C13-H16···O3$_{(C=O)}$<br>*$_{(NH3+)}$N2-H23···**O4**$_{(COO-)}$ & $_{(CH2)}$C17-H24···**O5**$_{(COO-)}$<br>*$_{(NH3+)}$N2-H31···**O5**$_{(COO-)}$ & $_{(CH2)}$C13-H16···O3$_{(C=O)}$<br>*$_{(NH3+)}$N2-H31···**O5**$_{(COO-)}$ & $_{(CH2)}$C17-H24···**O5**$_{(COO-)}$
	$C^{2}_{2}(19)$	*$_{(NH)}$**N1**-H12···**O2**$_{(C=O\_COOH)}$ & $_{(NH3+)}$N2-H22···**O4**$_{(COO-)}$<br>*$_{(NH)}$**N1**-H12···**O2**$_{(C=O\_COOH)}$ & $_{(NH3+)}$N2-H23···**O4**$_{(COO-)}$<br>*$_{(NH)}$**N1**-H12···**O2**$_{(C=O\_COOH)}$ & $_{(NH3+)}$N2-H31···**O5**$_{(COO-)}$<br>*$_{(NH)}$**N1**-H12···**O2**$_{(C=O\_COOH)}$ & $_{(NH3+)}$N2-H32···**O4**$_{(COO-)}$<br>*$_{(CH2)}$C8-H9···**O2**$_{(C=O\_COOH)}$ & $_{(CH2)}$C17-H24···**O5**$_{(COO-)}$
	$C^{2}_{2}(20)$	*$_{(NH3+)}$N2-H22···**O4**$_{(COO-)}$ & $_{(CH2)}$C15-H21···O3$_{(C=O)}$<br>*$_{(NH3+)}$N2-H23···**O4**$_{(COO-)}$ & $_{(CH2)}$C15-H21···O3$_{(C=O)}$<br>*$_{(NH3+)}$N2-H31···**O5**$_{(COO-)}$ & $_{(CH2)}$C15-H21···O3$_{(C=O)}$
	$D^{3}_{3}(8)$	($_{(OH2)}$O6-H32···**N1**$_{(NH)}$))$_{2}$ & $_{(NH)}$**N1**-H12···**O2**$_{(C=O\_COOH)}$

	$D^3_3(10)$	*((OH2)O6-H32···N1(NH))2 & (CH2)C8-H9···O2(C=O_COOH)<br>*((OH2)O6-H33···O1(OH_COOH))2 & (NH)N1-H12···O2(C=O_COOH)<br>*((OH2)O6-H33···O1(OH_COOH))2 & (CH2)C8-H9···O2(C=O_COOH)
	$D^3_3(11)$	*((OH2)O6-H32···N1(NH))2 & (CH2)C13-H16···O3(C=O)<br>*(CH2)C17-H24···O5(COO-) & ((CH2)C17-H25···O6(OH2))2
	$D^3_3(12)$	*((OH_COOH)O1-H11···O6(OH2))2 & (NH)N1-H12···O2(C=O_COOH)<br>*((OH_COOH)O1-H11···O6(OH2))2 & (CH2)C8-H9···O2(C=O_COOH)
	$D^3_3(13)$	((OH2)O6-H32···N1(NH))2 & (CH2)C15-H21···O3(C=O)
	$D^3_3(15)$	*((OH2)O6-H32···N1(NH))2 & (CH2)C17-H24···O5(COO-)<br>*(CH2)C13-H16···O3(C=O) & (CH2)C17-H25···O6(OH2)
	$D^3_3(17)$	*((OH2)O6-H32···N1(NH))2 & (NH3+)N2-H22···O4(COO-)<br>*((OH2)O6-H32···N1(NH))2 & (NH3+)N2-H23···O4(COO-)<br>*((OH2)O6-H32···N1(NH))2 & (NH3+)N2-H31···O5(COO-)<br>*((OH2)O6-H33···O1(OH_COOH))2 & (CH2)C13-H16···O3(C=O)<br>*(CH2)C15-H21···O3(C=O) & ((CH2)C17-H25···O6(OH2))2
	$D^3_3(18)$	(NH)N1-H12···O2(C=O_COOH) & (CH2)C17-H25···O6(OH2)
	$D^3_3(19)$	*((OH_COOH)O1-H11···O6(OH2))2 & (CH2)C13-H16···O3(C=O)<br>*((OH2)O6-H33···O1(OH_COOH))2 & (CH2)C15-H21···O3(C=O)<br>*(NH3+)N2-H22···O4(COO-) & ((CH2)C17-H25···O6(OH2))2<br>*(NH3+)N2-H23···O4(COO-) & ((CH2)C17-H25···O6(OH2))2<br>*(NH3+)N2-H31···O5(COO-) & ((CH2)C17-H25···O6(OH2))2
	$D^3_3(20)$	(CH2)C8-H9···O2(C=O_COOH) & ((CH2)C17-H25···O6(OH2))2
**GERXEI**	$C(2)$	(OH_COOH)O2-H11···O2(OH_COOH)
	$C(5)$	*(NH)N1-H12···O1(C=O_COOH)<br>*(CH2)C8-H8···O1(C=O_COOH)
	$C(6)$	*(CH2)C7-H7···O1(C=O_COOH)<br>*(CH2)C7-H24···O5(COO-)<br>*(CH2)C13-H16···O3(C=O)
	$C(9)$	*(CH2cycl)C17-H25···O1(C=O_COOH)<br>*(CH2cycl)C17-H25···O2(OH_COOH)
	$C(12)$	(NH3+)N2-H22···O4(COO-)<br>(NH3+)N2-H23···O4(COO-)<br>(NH3+)N2-H31···O5(COO-)
	$C(15)$	(CHcycl)C4-H3···O5(COO-)
	$C^1_2(4)$	(NH3+)N2-H22···O4(COO-) & (NH3+)N2-H23···O4(COO-)
	$C^1_2(8)$	(NH)N1-H12···O1(C=O_COOH) & (CH2cycl)C17-H25···O1(C=O_COOH)
	$C^1_2(10)$	(CH2)C8-H8···O1(C=O_COOH) & (CH2cycl)C17-H25···O1(C=O_COOH)
	$C^1_2(11)$	*(OH_COOH)O2-H11···O2(OH_COOH) & (CH2cycl)C17-H25···O2(OH_COOH)<br>*(CH2)C7-H7···O1(C=O_COOH) & (CH2cycl)C17-H25···O1(C=O_COOH)
	$C^1_2(12)$	(NH3+)N2-H31···O5(COO-) & (CH2cycl)C17-H24···O5(COO-)
	$C^1_2(15)$	(CH2cycl)C17-H24···O5(COO-) & (CHcycl)C4-H3···O5(COO-)
	$C^1_2(17)$	(NH3+)N2-H31···O5(COO-) (CHcycl)C4-H3···O5(COO-)
	$C^2_2(6)$	*(NH3+)N2-H22···O4(COO-) & (NH3+)N2-H31···O5(COO-)<br>*(NH3+)N2-H23···O4(COO-) & (NH3+)N2-H31···O5(COO-)
	$C^2_2(9)$	*(OH_COOH)O2-H11···O2(OH_COOH) & (NH)N1-H12···O1(C=O_COOH)<br>*(OH_COOH)O2-H11···O2(OH_COOH) & (CH2)C8-H8···O1(C=O_COOH)
	$C^2_2(10)$	*(OH_COOH)O2-H11···O2(OH_COOH) & (CH2)C7-H7···O1(C=O_COOH)<br>*(NH)N1-H12···O1(C=O_COOH) & (CH2)C8-H8···O1(C=O_COOH)<br>*(NH)N1-H12···O1(C=O_COOH) & (CH2cycl)C17-H25···O2(OH_COOH)
	$C^2_2(11)$	*(NH)N1-H12···O1(C=O_COOH) & (CH2)C7-H7···O1(C=O_COOH)<br>*(CH2)C7-H7···O1(C=O_COOH) & (CH2)C8-H8···O1(C=O_COOH)
	$C^2_2(12)$	*(NH3+)N2-H22···O4(COO-) & (CH2)C13-H16···O3(C=O)<br>*(CH2)C8-H8···O1(C=O_COOH) & (CH2cycl)C17-H25···O1(C=O_COOH)<br>*(NH3+)N2-H31···O5(COO-) & (CH2)C13-H16···O3 (C=O)
	$C^2_2(13)$	*(OH_COOH)O2-H11···O2(OH_COOH) & (CH2cycl)C17-H25···O1(C=O_COOH)<br>*(NH)N1-H12···O1(C=O_COOH) & (CH2)C13-H16···O3(C=O)<br>*(NH)N1-H12···O1(C=O_COOH) & (CH2cycl)C17-H25···O2(OH_COOH)<br>*(CH2)C7-H7···O1(C=O_COOH) & (CH2cycl)C17-H25···O1(C=O_COOH)<br>*(CH2)C13-H16···O3(C=O) & (CH2cycl)C17-H25···O1(C=O_COOH)<br>*(CH2)C13-H16···O3(C=O) & (CH2cycl)C17-H25···O2(OH_COOH)<br>*(CH2cycl)C17-H24···O5(COO-) & (CH2cycl)C17-H25···O1(C=O_COOH)<br>*(CH2cycl)C17-H24···O5(COO-) & (CH2cycl)C17-H25···O2(OH_COOH)

	$C^2_2(14)$	*$_{(NH)}$**N1**-H12···**O1**$_{(C=O\_COOH)}$ & $_{(CH2cycl)}$C17-H25···**O1**$_{(C=O\_COOH)}$<br>*$_{(NH3+)}$N2-H22···**O4**$_{(COO-)}$ & $_{(CH2cycl)}$C17-H24···**O5**$_{(COO-)}$<br>*$_{(CH2)}$C8-H8···**O1**$_{(C=O\_COOH)}$ & $_{(CH2cycl)}$C17-H25···**O1**$_{(C=O\_COOH)}$<br>*$_{(CH2)}$C13-H16···O3$_{(C=O)}$ & $_{(CH2cycl)}$C17-H24···**O5**$_{(COO-)}$
	$C^2_2(15)$	*$_{(CH2)}$C7-H7···**O1**$_{(C=O\_COOH)}$ & $_{(CH2cycl)}$C17-H25···**O1**$_{(C=O\_COOH)}$<br>*$_{(CH2)}$C8-H8···**O1**$_{(C=O\_COOH)}$ & $_{(CH2)}$C13-H16···O3$_{(C=O)}$<br>*$_{(CH2)}$C13-H16···O3$_{(C=O)}$ & $_{(CH2cycl)}$C17-H25···**O1**$_{(C=O\_COOH)}$<br>*$_{(CH2)}$C13-H16···O3$_{(C=O)}$ & $_{(CH2cycl)}$C17-H25···**O2**$_{(OH\_COOH)}$<br>*$_{(CH2cycl)}$C17-H24···**O5**$_{(COO-)}$ & $_{(CH2cycl)}$C17-H25···**O2**$_{(OH\_COOH)}$
	$C^2_2(16)$	*$_{(OH\_COOH)}$**O2**-H11···**O2**$_{(OH\_COOH)}$ & $_{(CH2)}$C13-H16···O3$_{(C=O)}$<br>*$_{(CH2)}$C13-H16···O3$_{(C=O)}$ & $_{(CH2)}$C7-H7···**O1**$_{(C=O\_COOH)}$<br>*$_{(CH2cycl)}$C17-H24···**O5**$_{(COO-)}$ & $_{(CH2cycl)}$C17-H25···**O1**$_{(C=O\_COOH)}$<br>*$_{(CH2cycl)}$C17-H25···**O1**$_{(C=O\_COOH)}$ & $_{(CHcycl)}$C4-H3···**O5**$_{(COO-)}$<br>*$_{(CH2cycl)}$C17-H25···**O2**$_{(OH\_COOH)}$ & $_{(CHcycl)}$C4-H3···**O5**$_{(COO-)}$
level 2	$C^2_2(17)$	*$_{(NH)}$**N1**-H12···**O1**$_{(C=O\_COOH)}$ & $_{(CH2cycl)}$C17-H24···**O5**$_{(COO-)}$<br>*$_{(NH3+)}$N2-H22···**O4**$_{(COO-)}$ & $_{(CH2cycl)}$C17-H25···**O1**$_{(C=O\_COOH)}$<br>*$_{(NH3+)}$N2-H22···**O4**$_{(COO-)}$ & $_{(CH2cycl)}$C17-H25···**O2**$_{(OH\_COOH)}$<br>*$_{(NH3+)}$N2-H23···**O4**$_{(COO-)}$ & $_{(CH2cycl)}$C17-H25···**O1**$_{(C=O\_COOH)}$<br>*$_{(NH3+)}$N2-H23···**O4**$_{(COO-)}$ & $_{(CH2cycl)}$C17-H25···**O2**$_{(OH\_COOH)}$<br>*$_{(NH3+)}$N2-H31···**O5**$_{(COO-)}$ & $_{(CH2cycl)}$C17-H25···**O1**$_{(C=O\_COOH)}$<br>*$_{(NH3+)}$N2-H31···**O5**$_{(COO-)}$ & $_{(CH2cycl)}$C17-H25···**O2**$_{(OH\_COOH)}$<br>*$_{(CHcycl)}$C4-H3···**O5**$_{(COO-)}$ & $_{(CH2)}$C7-H7···**O1**$_{(C=O\_COOH)}$
	$C^2_2(18)$	*$_{(NH)}$**N1**-H12···**O1**$_{(C=O\_COOH)}$ & $_{(CHcycl)}$C4-H3···**O5**$_{(COO-)}$<br>*$_{(NH3+)}$N2-H22···**O4**$_{(COO-)}$ & $_{(CH2)}$C13-H16···O3$_{(C=O)}$<br>*$_{(NH3+)}$N2-H22···**O4**$_{(COO-)}$ & $_{(CH2cycl)}$C17-H24···**O5**$_{(COO-)}$<br>*$_{(NH3+)}$N2-H23···**O4**$_{(COO-)}$ & $_{(CH2cycl)}$C17-H24···**O5**$_{(COO-)}$<br>*$_{(NH3+)}$N2-H23···**O4**$_{(COO-)}$ & $_{(CH2)}$C13-H16···O3$_{(C=O)}$<br>*$_{(NH3+)}$N2-H31···**O5**$_{(COO-)}$ & $_{(CH2)}$C13-H16···O3$_{(C=O)}$<br>*$_{(NH3+)}$N2-H31···**O5**$_{(COO-)}$ & $_{(CH2cycl)}$C17-H24···**O5**$_{(COO-)}$<br>*$_{(CHcycl)}$C4-H3···**O5**$_{(COO-)}$ & $_{(CH2)}$C8-H8···**O1**$_{(C=O\_COOH)}$<br>*$_{(CH2cycl)}$C17-H25···**O1**$_{(C=O\_COOH)}$ & $_{(CH2cycl)}$C17-H25···**O2**$_{(OH\_COOH)}$
	$C^2_2(19)$	*$_{(NH)}$**N1**-H12···**O1**$_{(C=O\_COOH)}$ & $_{(NH3+)}$N2-H22···**O4**$_{(COO-)}$<br>*$_{(NH)}$**N1**-H12···**O1**$_{(C=O\_COOH)}$ & $_{(NH3+)}$N2-H23···**O4**$_{(COO-)}$<br>*$_{(NH)}$**N1**-H12···**O1**$_{(C=O\_COOH)}$ & $_{(NH3+)}$N2-H31···**O5**$_{(COO-)}$<br>*$_{(NH3+)}$N2-H22···**O4**$_{(COO-)}$ & $_{(CHcycl)}$C4-H3···**O5**$_{(COO-)}$<br>*$_{(NH3+)}$N2-H23···**O4** & $_{(CHcycl)}$C4-H3···**O5**$_{(COO-)}$<br>*$_{(CHcycl)}$C4-H3···**O5**$_{(COO-)}$ & $_{(CH2)}$C13-H16···O3$_{(C=O)}$<br>*$_{(CH2)}$C8-H8···**O1**$_{(C=O\_COOH)}$ & $_{(CH2cycl)}$C17-H24···**O5**$_{(COO-)}$
	$C^2_2(20)$	*($_{(OH\_COOH)}$**O2**-H11···**O2**$_{(OH\_COOH)}$)$_2$ & $_{(CH2cycl)}$C17-H24···**O5**$_{(COO-)}$<br>*$_{(NH)}$**N1**-H12···**O1**$_{(C=O\_COOH)}$ & $_{(CHcycl)}$C4-H3···**O5**$_{(COO-)}$<br>*$_{(CHcycl)}$C4-H3···**O5**$_{(COO-)}$ & $_{(CH2)}$C8-H8···**O1**$_{(C=O\_COOH)}$<br>*$_{(CH2)}$C7-H7···**O1**$_{(C=O\_COOH)}$ & $_{(CH2cycl)}$C17-H24···**O5**$_{(COO-)}$
	$R^1_2(5)$	$_{(CH2)}$C7-H7···**O1**$_{(C=O\_COOH)}$ & $_{(CH2)}$C8-H8···**O1**$_{(C=O\_COOH)}$
	$R^1_2(6)$	$_{(NH)}$**N1**-H12···**O1**$_{(C=O\_COOH)}$ & $_{(CH2)}$C8-H8···**O1**$_{(C=O\_COOH)}$
	$R^1_2(7)$	$_{(NH)}$**N1**-H12···**O1**$_{(C=O\_COOH)}$ & $_{(CH2)}$C7-H7···**O1**$_{(C=O\_COOH)}$
	$R^2_2(11)$	$_{(OH\_COOH)}$**O2**-H11···**O2**$_{(OH\_COOH)}$ & $_{(CH2cycl)}$C17-H25···**O2**$_{(OH\_COOH)}$
	$R^2_1(4)$	$_{(CH2cycl)}$C17-H25···**O1**$_{(C=O\_COOH)}$ & $_{(CH2cycl)}$C17-H25···**O2**$_{(OH\_COOH)}$
	$R^2_2(12)$	$_{(NH3+)}$N2-H23···**O4**$_{(COO-)}$ & $_{(CH2)}$C13-H16···O3$_{(C=O)}$
	$R^2_2(13)$	*$_{(OH\_COOH)}$**O2**-H11···**O2**$_{(OH\_COOH)}$ & $_{(CH2cycl)}$C17-H25···**O1**$_{(C=O\_COOH)}$<br>*$_{(NH)}$**N1**-H12···**O4**$_{(COO-)}$ & $_{(CH2)}$C13-H16···O3$_{(C=O)}$
	$R^2_2(14)$	*$_{(NH3+)}$N2-H23···**O4**$_{(COO-)}$ & $_{(CH2cycl)}$C17-H24···**O5**$_{(COO-)}$<br>*$_{(CH2)}$C13-H16···O3$_{(C=O)}$ & $_{(CH2cycl)}$C17-H24···**O5**$_{(COO-)}$
	$R^2_2(15)$	$_{(CH2)}$C8-H8···**O1**$_{(C=O\_COOH)}$ & $_{(CH2)}$C13-H16···O3$_{(C=O)}$
	$R^2_2(16)$	$_{(CH2)}$C7-H7···**O1**$_{(C=O\_COOH)}$ & $_{(CH2)}$C13-H16···O3$_{(C=O)}$
	$R^2_2(17)$	$_{(NH)}$**N1**-H12···**O1**$_{(C=O\_COOH)}$ & $_{(CH2cycl)}$C17-H24···**O5**$_{(COO-)}$
	$R^2_2(19)$	*$_{(NH)}$**N1**-H12···**O1**$_{(C=O\_COOH)}$ & $_{(NH3+)}$N2-H23···**O4**$_{(COO-)}$<br>*$_{(CH2)}$C8-H8···**O1**$_{(C=O\_COOH)}$ & $_{(CH2cycl)}$C17-H24···**O5**$_{(COO-)}$
	$R^2_2(20)$	$_{(CH2)}$C7-H7···**O1**$_{(C=O\_COOH)}$ & $_{(CH2cycl)}$C17-H24···**O5**$_{(COO-)}$
	$R^2_3(16)$	($_{(NH3+)}$N2-H22···**O4**$_{(COO-)}$)$_2$ & $_{(NH3+)}$N2-H23···**O4**$_{(COO-)}$
	$R^2_3(17)$	$_{(NH)}$**N1**-H12···**O1**$_{(C=O\_COOH)}$ & ($_{(CH2cycl)}$C17-H25···**O1**$_{(C=O\_COOH)}$)$_2$
	$R^2_3(19)$	$_{(CH2)}$C8-H8···**O1**$_{(C=O\_COOH)}$ & ($_{(CH2cycl)}$C17-H25···**O1**$_{(C=O\_COOH)}$)$_2$
	$R^2_3(20)$	$_{(CH2)}$C7-H7···**O1**$_{(C=O\_COOH)}$ & ($_{(CH2cycl)}$C17-H25···**O1**$_{(C=O\_COOH)}$)$_2$
	$R^3_3(11)$	*($_{(OH\_COOH)}$**O2**-H11···**O2**$_{(OH\_COOH)}$)$_2$ & $_{(NH)}$**N1**-H12···**O1**$_{(C=O\_COOH)}$

		*($_{(OH_COOH)}$O2-H11···O2$_{(OH_COOH)}$)$_2$ & $_{(CH2)}$C8-H8···O1$_{(C=O_COOH)}$
	$R^3_3(12)$	($_{(OH_COOH)}$O2-H11···O2$_{(OH_COOH)}$)$_2$ & $_{(CH2)}$C7-H7···O1$_{(C=O_COOH)}$
	$R^3_3(19)$	$_{(NH)}$N1-H12···O1$_{(C=O_COOH)}$ & ($_{(CH2cycl)}$C17-H25···O1$_{(C=O_COOH)}$)$_2$
	$R^4_4(18)$	*($_{(OH_COOH)}$O2-H11···O2$_{(OH_COOH)}$)$_2$ & ($_{(NH)}$N1-H12···O1$_{(C=O_COOH)}$)$_2$<br>*($_{(OH_COOH)}$O2-H11···O2$_{(OH_COOH)}$)$_2$ & ($_{(CH2)}$C8-H8···O1$_{(C=O_COOH)}$)$_2$<br>*($_{(OH_COOH)}$O2-H11···O2$_{(OH_COOH)}$)$_2$ & $_{(CH2)}$C13-H16···O3$_{(C=O)}$
	$R^4_4(20)$	($_{(CH2)}$C7-H7···O1$_{(C=O_COOH)}$)$_2$ & ($_{(OH_COOH)}$O2-H11···O2$_{(OH_COOH)}$)$_2$
**MCPRL01**	$C(6)$	*$_{(CH2cycl)}$C5-H7···O2$_{(C=O_COOH)}$<br>*$_{(CH2cycl)}$C5-H8···O2$_{(C=O_COOH)}$
	$C(7)$	*$_{(OH_COOH)}$O1-H1···O3$_{(C=O)}$<br>*$_{(CHcycl)}$C2-H2···S1
	$C(8)$	$_{(CH2)}$C8-H11···O1$_{(OH_COOH)}$
	$C(9)$	S1-H15···O2$_{(C=O_COOH)}$
level 2	$C^1_2(4)$	$_{(CH2cycl)}$C5-H7···O2$_{(C=O_COOH)}$ & $_{(CH2cycl)}$C5-H8···O2$_{(C=O_COOH)}$
	$C^1_2(9)$	$_{(CH2cycl)}$C5-H7···O2$_{(C=O_COOH)}$ & S1-H15···O2$_{(C=O_COOH)}$
	$C^2_2(6)$	$_{(CHcycl)}$C2-H2···S1 & S1-H15···O2$_{(C=O_COOH)}$
	$C^2_2(7)$	*$_{(CHcycl)}$C2-H2···S1 & $_{(CH2)}$C8-H11···O1$_{(OH_COOH)}$<br>*$_{(CH2)}$C8-H11···O1$_{(OH_COOH)}$ & S1-H15···O2$_{(C=O_COOH)}$
	$C^2_2(9)$	*$_{(OH_COOH)}$O1-H1···O3$_{(C=O)}$ & $_{(CH2cycl)}$C5-H7···O2$_{(C=O_COOH)}$<br>*$_{(OH_COOH)}$O1-H1···O3$_{(C=O)}$ & $_{(CH2cycl)}$C5-H8···O2$_{(C=O_COOH)}$
	$C^2_2(10)$	*$_{(OH_COOH)}$O1-H1···O3$_{(C=O)}$ & $_{(CHcycl)}$C2-H2···S1<br>*$_{(OH_COOH)}$O1-H1···O3$_{(C=O)}$ & S1-H15···O2$_{(C=O_COOH)}$<br>*$_{(CH2cycl)}$C5-H7···O2$_{(C=O_COOH)}$ & $_{(CH2)}$C8-H11···O1$_{(OH_COOH)}$<br>*$_{(CH2cycl)}$C5-H8···O2$_{(C=O_COOH)}$ & $_{(CH2)}$C8-H11···O1$_{(OH_COOH)}$
	$C^2_2(11)$	*$_{(CHcycl)}$C2-H2···S1 & $_{(CH2cycl)}$C5-H7···O2$_{(C=O_COOH)}$<br>*$_{(CHcycl)}$C2-H2···S1 & $_{(CH2cycl)}$C5-H8···O2$_{(C=O_COOH)}$
	$C^2_2(12)$	$_{(CH2cycl)}$C5-H7···O2$_{(C=O_COOH)}$ & $_{(CH2cycl)}$C5-H8···O2$_{(C=O_COOH)}$
	$C^2_2(13)$	*$_{(OH_COOH)}$O1-H1···O3$_{(C=O)}$ & $_{(CH2cycl)}$C5-H7···O2$_{(C=O_COOH)}$<br>*$_{(OH_COOH)}$O1-H1···O3$_{(C=O)}$ & $_{(CH2cycl)}$C5-H8···O2$_{(C=O_COOH)}$<br>*$_{(CHcycl)}$C2-H2···S1 & $_{(CH2cycl)}$C5-H7···O2$_{(C=O_COOH)}$<br>*$_{(CHcycl)}$C2-H2···S1 & $_{(CH2cycl)}$C5-H8···O2$_{(C=O_COOH)}$
	$C^2_2(14)$	*$_{(OH_COOH)}$O1-H1···O3$_{(C=O)}$ & $_{(CHcycl)}$C2-H2···S1<br>*$_{(CH2cycl)}$C5-H7···O2$_{(C=O_COOH)}$ & $_{(CH2)}$C8-H11···O1$_{(OH_COOH)}$<br>*$_{(CH2cycl)}$C5-H7···O2$_{(C=O_COOH)}$ & S1-H15···O2$_{(C=O_COOH)}$<br>*$_{(CH2cycl)}$C5-H8···O2$_{(C=O_COOH)}$ & $_{(CH2)}$C8-H11···O1$_{(OH_COOH)}$
	$C^2_2(15)$	*$_{(OH_COOH)}$O1-H1···O3$_{(C=O)}$ & $_{(CH2)}$C8-H11···O1$_{(OH_COOH)}$<br>*$_{(CHcycl)}$C2-H2···S1 & $_{(CH2)}$C8-H11···O1$_{(OH_COOH)}$<br>*$_{(CH2cycl)}$C5-H8···O2$_{(C=O_COOH)}$ & S1-H15···O2$_{(C=O_COOH)}$
	$C^2_2(16)$	*$_{(OH_COOH)}$O1-H1···O3$_{(C=O)}$ & S1-H15···O2$_{(C=O_COOH)}$<br>*$_{(CHcycl)}$C2-H2···S1 & S1-H15···O2$_{(C=O_COOH)}$
	$C^2_2(17)$	$_{(CH2)}$C8-H11···O1$_{(OH_COOH)}$ & S1-H13···O2$_{(C=O_COOH)}$
	$R^1_2(9)$	$_{(CH2cycl)}$C5-H8···O2$_{(C=O_COOH)}$ & S1-H15···O2$_{(C=O_COOH)}$
	$R^2_2(7)$	$_{(OH_COOH)}$O1-H1···O3$_{(C=O)}$ & $_{(CH2)}$C8-H11···O1$_{(OH_COOH)}$
	$R^2_3(10)$	($_{(CH2cycl)}$C5-H7···O2$_{(C=O_COOH)}$)$_2$ & $_{(CH2cycl)}$C5-H8···O2$_{(C=O_COOH)}$
	$R^2_3(15)$	($_{(CH2cycl)}$C5-H7···O2$_{(C=O_COOH)}$)$_2$ & S1-H15···O2$_{(C=O_COOH)}$
	$R^3_4(16)$	($_{(CH2cycl)}$C5-H7···O2$_{(C=O_COOH)}$)$_2$ & $_{(CH2cycl)}$C5-H8···O2$_{(C=O_COOH)}$
**YOZTIS**	$C(4)$	$_{(CH2)}$C6-H8···S1
	$C(6)$	*$_{(CH2)}$C13-H16···O5$_{(C=O)}$<br>*$_{(CH2cycl)}$C16-H23···O3$_{(C=O_COOH)}$
	$C(7)$	*$_{(OH_COOH)}$O1-H1···O4$_{(C=O)}$<br>*$_{(OH_COOH)}$O2-H2···O5$_{(C=O)}$
	$C(8)$	*$_{(CHcycl)}$C2-H3···S2<br>*$_{(CH2)}$C5-H6···O3$_{(C=O_COOH)}$<br>*$_{(CH3)}$C11-H11···O3$_{(C=O_COOH)}$<br>*$_{(CH2)}$C12-H14···S2
	$C(10)$	$_{(CH3)}$C15-H21···O4$_{(C=O)}$
level 2	$C^1_2(8)$	*$_{(OH_COOH)}$O2-H2···O5$_{(C=O)}$ & $_{(CH2cycl)}$C18-H16···O5$_{(C=O)}$<br>*$_{(CH2)}$C5-H6···O3$_{(C=O_COOH)}$ & $_{(CH2cycl)}$C16-H23···O3$_{(C=O_COOH)}$<br>*$_{(CH3)}$C11-H11···O3$_{(C=O_COOH)}$ & $_{(CH2cycl)}$C16-H23···O3$_{(C=O_COOH)}$
	$C^1_2(15)$	$_{(OH_COOH)}$O1-H1···O4$_{(C=O)}$ & $_{(CH3)}$C15-H21···O4$_{(C=O)}$
	$C^1_2(16)$	$_{(CHcycl)}$C2-H3···S2 & $_{(CH2)}$C12-H14···S2

	$C^2_2(8)$	(CHcycl)C2-H3···S2 & (CH2)C5-H6···O3(C=O_COOH)
	$C^2_2(9)$	*(OH_COOH)O1-H1···O4(C=O) & (CH2cycl)C16-H23···O3(C=O_COOH)<br>*(CH2)C12-H14···S2 & (CH2)C13-H19···O5(C=O)
	$C^2_2(10)$	*(CHcycl)C2-H3···S2 & (CH3)C11-H11···O3(C=O_COOH)<br>*(CHcycl)C2-H3···S2 & (CH3)C15-H21···O4(C=O)<br>*(CH2)C6-H8···S1 & (CH2)C12-H14···S2<br>*(CH2)C6-H8···S1 & (CH3)C15-H21···O4(C=O)
	$C^2_2(11)$	*(OH_COOH)O1-H1···O4(C=O) & (CHcycl)C2-H3···S2<br>*(OH_COOH)O2-H2···O5(C=O) & (CH2)C12-H14···S2
	$C^2_2(12)$	*(C=O_COOH)O6-H8···S1 & (CHcycl)C2-H3···S2<br>*(CH2)C6-H8···S1 & (CH2)C12-H14···S2
	$C^2_2(13)$	*(OH_COOH)O1-H1···O4(C=O) & (CH2cycl)C16-H23···O3(C=O_COOH)<br>*(OH_COOH)O2-H2···O5(C=O) & (CH2cycl)C18-H16···O5(C=O)
	$C^2_2(14)$	*(CHcycl)C2-H3···S2 & (CH2cycl)C16-H23···O3(C=O_COOH)<br>*(CH2)C5-H6···O3(C=O_COOH) & (CH2)C6-H8···S1<br>*(CH2)C5-H6···O3(C=O_COOH) & (CH3)C15-H21···O4(C=O)<br>*(CH2)C5-H6···O3(C=O_COOH) & (CH2cycl)C16-H23···O3(C=O_COOH)<br>*(CH2)C6-H8···S1 & (CH2)C13-H16···O5(C=O)<br>*(CH2)C6-H8···S1 & (CH3)C15-H21···O4(C=O)<br>*(CH3)C11-H11···O3(C=O_COOH) & (CH2cycl)C16-H23···O3(C=O_COOH)<br>*(CH2)C13-H16···O5(C=O) & (CH2)C12-H14···S2<br>*(CH3)C15-H21···O4 & (CH2)C12-H14···S2
		(OH_COOH)O1-H1···O4(C=O) & (CHcycl)C2-H3···S2<br>(OH_COOH)O1-H1···O4(C=O) & (CH2)C5-H6···O3(C=O_COOH)<br>*(OH_COOH)O1-H1···O4(C=O) & (CH3)C11-H11···O3(C=O_COOH)<br>*(OH_COOH)O2-H2···O5(C=O) & (CH2)C6-H8···S1<br>*(OH_COOH)O2-H2···O5(C=O) & (CH2)C12-H14···S2
	$C^2_2(16)$	*(CHcycl)C2-H3···S2 & (CH2)C5-H6···O3(C=O_COOH)<br>*(CHcycl)C2-H3···S2 & (CH2)C12-H14···S2<br>*(CHcycl)C2-H3···S2 & (CH3)C11-H11···O3(C=O_COOH)<br>*(CH2)C5-H6···O3(C=O_COOH) & (CH3)C11-H11···O3(C=O_COOH)<br>*(CH2)C6-H8···S1 & C11-H11···O3(C=O_COOH)<br>*(CH3)C11-H11···O3(C=O_COOH) & (CH3)C15-H21···O4(C=O)
	$C^2_2(17)$	*(OH_COOH)O1-H1···O4(C=O) & (CH2)C6-H8···S1<br>*(OH_COOH)O1-H1···O4(C=O) & (CH3)C15-H21···O4(C=O)
	$C^2_2(18)$	*(CHcycl)C2-H3···S2 & (CH3)C15-H21···O4(C=O)<br>*(CH2)C5-H6···O3(C=O_COOH) & (CH3)C15-H21···O4(C=O)<br>*(CH2)C6-H8···S1 & C16-H23···O3(C=O_COOH)<br>*(CH3)C11-H11···O3(C=O_COOH) & (CH3)C15-H21···O4(C=O)<br>*(CH2)C12-H14···S2 & (CH3)C15-H21···O4(C=O)<br>*(CH2)C13-H16···O5(C=O) & (CH3)C15-H21···O4(C=O)<br>*(CH2cycl)C16-H23···O3(C=O_COOH) & (CH3)C15-H21···O4(C=O)
	$C^2_2(19)$	(OH_COOH)O2-H2···O5(C=O) & (CH3)C15-H21···O4(C=O)
	$C^2_2(20)$	*(CHcycl)C2-H3···S2 & (CH2)C13-H16···O5(C=O)<br>*(CH2)C5-H6···O3(C=O_COOH) & (CH2)C12-H14···S2
	$R^1_2(6)$	(CH2)C5-H6···O3(C=O_COOH) & (CH3)C11-H11···O3(C=O_COOH)
	$R^2_2(9)$	*(OH_COOH)O1-H1···O4(C=O) & (CH2)C5-H6···O3(C=O_COOH)<br>*(OH_COOH)O1-H1···O4(C=O) & (CH3)C11-H11···O3(C=O_COOH)
	$R^2_2(10)$	(C=O_COOH)O6-H8···S1 & (CHcycl)C2-H3···S2
	$R^2_2(12)$	(CHcycl)C2-H3···S2 & C16-H23···O3(C=O_COOH)
	$R^2_2(14)$	(CH2)C6-H8···S1 & (CH2)C13-H16···O5(C=O)
	$R^2_2(18)$	(CH2)C6-H8···S1 & (CH2cycl)C16-H23···O3(C=O_COOH)
	$R^2_2(20)$	(CHcycl)C2-H3···S2 & (CH2)C13-H16···O5(C=O)

Other modified proline-based ACEI

**QOQWAU**	$S(8)$	(CH2)C17-H26···O3(C=O)
	$S(10)$	(CH2)C16-H25···O2(C=O_COOH)
	$C(8)$	*(OH_COOH)O1-H1···N2(NH)<br>*(CH3)C11-H16···O1(OH_COOH)
	$C(10)$	(CHcycl)C3-H6···O5(C=O)
level 2	$C^2_2(11)$	(OH_COOH)O1-H1···N2(NH) & (CHcycl)C3-H6···O5(C=O)
	$C^2_2(13)$	(CHcycl)C3-H6···O5(C=O) & (CH3)C11-H16···O1(OH_COOH)
	$C^2_2(16)$	(OH_COOH)O1-H1···N2(NH) & (CH3)C11-H16···O1(OH_COOH)

	$C^2_2(18)$	*(OH_COOH)O1-H1···N2(NH) & (CHcycl)C3-H6···O5(C=O)<br>*(CHcycl)C3-H6···O5(C=O) & (CH3)C11-H16···O1(OH_COOH)
	$R^2_2(6)$	(OH_COOH)O1-H1···N2(NH) & (CH3)C11-H16···O1(OH_COOH)
**EDALEC**	*C*(6)	(OH)O8-H40···O6(OH)<br>(CH2)C11-H12···N1(NH)
	C(15)	(CHcycl)C3-H3···O4(COO-)
	D(2)	*(CH2cycl)C18-H23···O7(OH)<br>*(CH2cycl)C18-H23···O8(OH)<br>*(CH2cycl)C18-H24···O7(OH)<br>*(CH2cycl)C19-H25···O7(OH)
	S(6)	(CH2cycl)C17-H22···O3(C=O)
level 2	$C^1_2(4)$	(CH2cycl)C18-H23···O7(OH) & (CH2cycl)C18-H24···O7 (OH)
	$C^1_2(5)$	*(CH2cycl)C18-H23···O7(OH) & (CH2cycl)C19-H25···O7(OH)<br>*(CH2cycl)C18-H24···O7(OH) & (CH2cycl)C19-H25···O7(OH)
	$C^2_2(8)$	(CH2cycl)C18-H23···O8(OH) & (CH2cycl)C18-H24···O7(OH)
	$C^2_2(9)$	(CH2cycl)C18-H23···O8(OH) & ((CH2cycl)C19-H25···O7(OH)
	$C^2_2(19)$	(CH2cycl)C11-H12···N1(NH) & (CHcycl)C3-H3···O4(COO-)
	$R^2_1(6)$	(CH2cycl)C18-H23···O7(OH) & (CH2cycl)C18-H23···O8(OH)
	$D^3_3(9)$	(CH2)C8-H40···O6(OH) & (CH2cycl)C18-H23···O8(OH)
	$D^3_3(13)$	*(OH)O8-H40···O6(OH) & ((CH2cycl)C18-H24···O7(OH))2<br>*(OH)O8-H40···O6(OH) & ((CH2cycl)C19-H25···O7(OH))2
**FIFGEG**	*D*(2)	*(NH)N2-H24···O6(solv)<br>*(CH3)C14-H20···O7(solv)
	C(5)	(CH2cycl)C2-H2···O4(COO-)
	C(8)	(CHcycl)C21-H26···O3(COO-)
	C(9)	(CH)C11-H13···O4(COO-)
level 2	$C^2_2(7)$	(CH)C11-H13···O4(COO-) & (CHcycl)C21-H26···O3(COO-)
	$C^2_2(14)$	(CH2cycl)C2-H2···O4(COO-) & (CH)C11-H13···O4(COO-)
	$C^2_2(17)$	(CH)C11-H13···O4(COO-) & (CHcycl)C21-H26···O3(COO-)
	$R^1_2(10)$	(CH2cycl)C2-H2···O4(COO-) & (CH)C11-H13···O4(COO-)
	$D^2_2(7)$	(NH)N2-H24···O6(solv) & (CH3)C14-H20···O7(solv)
	$D^3_3(14)$	((NH)N2-H24···O6(solv))2 & (CH)C11-H13···O4(COO-)
	$D^3_3(15)$	((NH)N2-H24···O6(solv))2 & (CHcycl)C21-H26···O3(COO-)
	$D^3_3(16)$	(CH)C11-H13···O4(COO-) & ((CH3)C14-H20···O7(solv))2
	$D^3_3(18)$	*((NH)N2-H24···O6(solv))2 & (CH2cycl)C2-H2···O4(COO-)<br>*(CH2cycl)C2-H2···O4(COO-) & ((CH3)C14-H20···O7(solv))2
	$D^3_3(19)$	((CH3)C14-H20···O7(solv))2 & (CHcycl)C21-H26···O3(COO-)
**IQISAE**	*S*(6)	(CH2)C6-H9···O1(C=O)
	S(8)	(CH2)C6-H10···O5(C=O)
	S(10)	(CH2)C5-H7···O5(C=O)
	C(8)	(NH)N2-H31···O4(C=O_COOH) & (CH3)C24-H33···O4(C=O_COOH)
	C(9)	(CHcycl)C9-H12···O1(C=O)
	C(10)	(CH3)C4-H5···O5(C=O)
	C(11)	(CH2)C15-H21···O1(C=O)
level 2	$C^1_2(16)$	(CHcycl)C9-H12···O1(C=O) & (CH2)C15-H21···O1(C=O)
	$C^2_2(13)$	(NH)N2-H31···O4(C=O_COOH) & (CH2)C15-H21···O1(C=O)
	$C^2_2(14)$	(NH)N2-H31···O4(C=O_COOH) & (CH3)C4-H5···O5(C=O)
	$C^2_2(15)$	(CH2)C5-H21···O1(C=O) & (CH3)C24-H33···O4(C=O_COOH)
	$C^2_2(16)$	*(NH)N2-H31···O4(C=O_COOH) & (CH3)C24-H33···O4(C=O_COOH)<br>*(CH3)C4-H5···O5(C=O) & (CH3)C24-H33···O4(C=O_COOH)
	$C^2_2(17)$	(CH3)C4-H5···O5(C=O) & (CHcycl)C9-H12···O1(C=O)
	$C^2_2(18)$	*(NH)N2-H31···O4(C=O_COOH) & (CH3)C4-H5···O5(C=O)<br>*(CH3)C4-H5···O5(C=O) & (CH3)C24-H33···O4(C=O_COOH)
	$C^2_2(19)$	*(NH)N2-H31···O4(C=O_COOH) & (CHcycl)C9-H12···O1(C=O)<br>*(NH)N2-H31···O4(C=O_COOH) & (CH2)C15-H21···O1(C=O)<br>*(CH3)C4-H5···O5(C=O) & (CHcycl)C9-H12···O1(C=O)<br>*(CH2)C15-H21···O1(C=O) & (CH3)C24-H33···O4(C=O_COOH)
	$C^2_2(20)$	(CHcycl)C9-H12···O1(C=O) & (CH2)C15-H21···O1(C=O)
	$R^1_2(6)$	(NH)N2-H31···O4(C=O_COOH) & (CH3)C24-H33···O4(C=O_COOH)

	$R^2_2(13)$	$_{(CH3)}$C4-H5···O5$_{(C=O)}$ & $_{(CH2)}$C15-H21···O1$_{(C=O)}$
**RUWBAM**	$S(8)$	$_{(CH2)}$C1-H5···O1$_{(C=O)}$
	$C(5)$	$_{(CH3)}$C12-H26···O1$_{(C=O)}$
	$C(7)$	$_{(CH3)}$C12-H24···O2$_{(C=O)}$
	$C(8)$	$_{(NH2+)}$N1-H14···O4$_{(C=O_COOH)}$
	$C(9)$	$_{(CHcycl)}$C17-H7···O3$_{(C-O-C)}$
	$D(2)$	*$_{(OH2)}$O6-H32···S1<br>*$_{(OH_COOH)}$O5-H15···Cl1<br>*$_{(OH2)}$O6-H33···Cl1<br>*$_{(NH2+)}$N1-H13···Cl1<br>*$_{(CH2cycl)}$C7-H16···O6$_{(OH2)}$<br>*$_{(CH2cycl)}$C9-H19···Cl1
level 2	$C^1_2(5)$	$_{(NH2+)}$N1-H13···Cl1 & $_{(CH)}$C4-H2···Cl1
	$C^1_2(8)$	*$_{(OH_COOH)}$O5-H15···Cl1 & $_{(CH2cycl)}$C9-H19···Cl1<br>*$_{(NH2+)}$N1-H13···Cl1 & $_{(CH2cycl)}$C9-H19···Cl1<br>*$_{(CH)}$C4-H2···O6$_{(OH2)}$ & $_{(CH2cycl)}$C7-H16···O6$_{(OH2)}$
	$C^1_2(9)$	$_{(OH_COOH)}$O5-H15···Cl1 & $_{(CH)}$C4-H2···Cl1
	$C^1_2(10)$	$_{(OH_COOH)}$O5-H15···Cl1 & $_{(NH2+)}$N1-H13···Cl1
	$C^2_2(6)$	$_{(OH2))}$O6-H32···S1 & $_{(CH2cycl)}$C7-H16···O6$_{(OH2)}$
	$C^2_2(10)$	$_{(CH3)}$C12-H24···O2$_{(C=O)}$ & $_{(CH3)}$C12-H26···O1$_{(C=O)}$
	$C^2_2(13)$	$_{(NH2+)}$N1-H14···O4$_{(C=O_COOH)}$ & $_{(CH3)}$C12-H26···O1$_{(C=O)}$
	$C^2_2(14)$	$_{(CH3)}$C12-H24···O2$_{(C=O)}$ & $_{(CHcycl)}$C17-H7···O3$_{(C-O-C)}$
	$C^2_2(15)$	$_{(NH2+)}$N1-H14···O4$_{(C=O_COOH)}$ & $_{(CH3)}$C12-H24···O2$_{(C=O)}$
	$C^2_2(16)$	$_{(CH3)}$C12-H24···O2$_{(C=O)}$ & $_{(CHcycl)}$C17-H7···O3$_{(C-O-C)}$
	$C^2_2(18)$	$_{(CH3)}$C12-H26···O1$_{(C=O)}$ & $_{(CHcycl)}$C17-H7···O3$_{(C-O-C)}$
	$C^2_2(19)$	$_{(NH2+)}$N1-H14···O4$_{(C=O_COOH)}$ & $_{(CHcycl)}$C17-H7···O3$_{(C-O-C)}$
	$R^1_2(7)$	$_{(CH)}$C4-H2···Cl1 & $_{(CH2cycl)}$C9-H19···Cl1
	$R^2_2(9)$	$_{(OH2)}$O6-H32···S1 & $_{(CH)}$C4-H2···O6$_{(OH2)}$
	$R^2_2(11)$	$_{(NH2+)}$N1-H14···O4$_{(C=O_COOH)}$ & $_{(CH3)}$C12-H26···O1$_{(C=O)}$
	$R^2_2(12)$	$_{(CH3)}$C12-H24···O2$_{(C=O)}$ & $_{(CH3)}$C12-H26···O1$_{(C=O)}$
	$R^2_2(13)$	$_{(NH2+)}$N1-H14···O4$_{(C=O)}$ & $_{(CH3)}$C12-H24···O2$_{(C=O)}$
	$D^1_2(3)$	*$_{(OH_COOH)}$O5-H15···Cl1 & $_{(OH2)}$O6-H33···Cl1<br>*$_{(OH2)}$O6-H33···Cl1 & $_{(NH2+)}$N1-H13···Cl1<br>*$_{(OH2)}$O6-H33···Cl1 & $_{(CH)}$C4-H2···Cl1<br>*$_{(OH2)}$O6-H33···Cl1 & $_{(CH2cycl)}$C9-H19···Cl1
	$D^2_1(3)$	$_{(CH)}$C4-H2···O6$_{(OH2)}$ & $_{(CH)}$C4-H2···Cl1
	$D^2_2(4)$	*$_{(OH2)}$O6-H33···Cl1 & $_{(CH)}$C4-H2···O6$_{(OH2)}$<br>*$_{(OH2)}$O6-H33···Cl1 & $_{(CH2cycl)}$C7-H16···O6$_{(OH2)}$
	$D^2_2(5)$	*$_{(OH2)}$O6-H32···S1 & $_{(OH2)}$O6-H33···Cl1
	$D^2_2(6)$	*$_{(OH2)}$O6-H32···S1 & $_{(CH2cycl)}$C9-H19···Cl1<br>*$_{(NH2+)}$N1-H13···Cl1 & $_{(CH)}$C4-H2···O6$_{(OH2)}$
	$D^2_2(7)$	$_{(CH2cycl)}$C7-H16···O6$_{(OH2)}$ & $_{(CH2cycl)}$C9-H19···Cl1
	$D^2_2(8)$	*$_{(OH_COOH)}$O5-H15···Cl1 & $_{(CH2cycl)}$C7-H16···O6$_{(OH2)}$<br>*$_{(CH)}$C4-H2···O6$_{(OH2)}$ & $_{(CH2cycl)}$C9-H19···Cl1
	$D^2_2(9)$	*$_{(OH_COOH)}$O5-H15···Cl1 & $_{(OH2)}$O6-H32···S1<br>*$_{(CH)}$C4-H2···Cl1 & $_{(CH2cycl)}$C7-H16···O6$_{(OH2)}$
	$D^2_2(10)$	*$_{(OH_COOH)}$O5-H15···Cl1 & $_{(CH)}$C4-H2···O6$_{(OH2)}$<br>*$_{(OH2)}$O6-H32···S1 & $_{(NH2+)}$N1-H13···Cl1<br>*$_{(OH2)}$O6-H32···S1 & $_{(CH)}$C4-H2···Cl1<br>*$_{(NH2+)}$N1-H13···Cl1 & $_{(CH2cycl)}$C7-H16···O6$_{(OH2)}$
	$D^3_3(10)$	*($_{(CH)}$C4-H2···O6$_{(OH2)}$)$_2$ & $_{(CH3)}$C12-H26···O1$_{(C=O)}$<br>*($_{(CH)}$C4-H2···Cl1)$_2$ & $_{(CH3)}$C12-H26···O1$_{(C=O)}$
	$D^3_3(12)$	*$_{(NH2+)}$N1-H13···Cl1 & $_{(CH3)}$C12-H24···O2$_{(C=O)}$<br>*$_{(NH2+)}$N1-H13···Cl1 & $_{(CH3)}$C12-H26···O1$_{(C=O)}$<br>*($_{(CH)}$C4-H2···O6$_{(OH2)}$)$_2$ & $_{(CH3)}$C12-H24···O2$_{(C=O)}$<br>*($_{(CH)}$C4-H2···Cl1)$_2$ & $_{(CH3)}$C12-H24···O2$_{(C=O)}$
	$D^3_3(13)$	*$_{(NH2+)}$N1-H13···Cl1 & $_{(NH2+)}$N1-H14···O4$_{(C=O_COOH)}$<br>*$_{(NH2+)}$N1-H14···O4$_{(C=O_COOH)}$ & ($_{(CH2cycl)}$C9-H19···Cl1)$_2$<br>*$_{(CH)}$C4-H2···Cl1 & $_{(NH2+)}$N1-H14···O4$_{(C=O_COOH)}$<br>*$_{(CH)}$C4-H2···O6$_{(OH2)}$ & $_{(NH2+)}$N1-H14···O4$_{(C=O_COOH)}$
	$D^3_3(14)$	($_{(CH2cycl)}$C9-H19···Cl1)$_2$ & $_{(CH3)}$C12-H26···O1$_{(C=O)}$
	$D^3_3(15)$	*$_{(NH2+)}$N1-H14···O4$_{(C=O_COOH)}$ & $_{(OH_COOH)}$O5-H15···Cl1

		*(NH2+)N1-H14···O4(C=O_COOH) & ((CH2cycl)C7-H16···O6(OH2))2
	D^3_3(16)	*((OH2)O6-H32···S1)2 & (NH2+)N1-H14···O4(C=O_COOH)<br>*((OH2)O6-H32···S1)2 & (CH3)C12-H26···O1(C=O)<br>*((NH2+)N1-H13···Cl1)2 & (CHcycl)C17-H7···O3(C-O-C)<br>*((CH2cycl)C7-H16···O6(OH2))2 & (CH3)C12-H26···O1(C=O)
	D^3_3(18)	*((OH_COOH)O5-H15···Cl1)2 & (CH3)C12-H26···O1(C=O)<br>*((CH)C4-H2···O6(OH2))2 & (CHcycl)C17-H7···O3(C-O-C)<br>*((CH)C4-H2···Cl1)2 & (CHcycl)C17-H7···O3(C-O-C)<br>*((CH2cycl)C9-H19···Cl1)2 & (CH3)C12-H24···O2(C=O)
	D^3_3(20)	*((OH2)O6-H32···S1)2 & (CH3)C12-H24···O2(C=O)<br>*((CH2cycl)C7-H16···O6(OH2))2 & (CHcycl)C18-H24···O2(C=O)
**TUHMOY**	*S*(6)	(CH)C19-H23···O4(Na-O)
**TUHMUE**	*S*(6)	(C=O)O6-H45···O2(C-O-Na)
	S(9)	(CH2cycl)C26-H21···O8(C=O)
	R(4)	(CH)C29-H23···S3
	R(7)	(CH3)C9-H10···O4(C=O)
	C(14)	(CH)C13-H14···O6(C=O)
	C(15)	(CH)C36-H31···O2(C-O-Na)

Table S21. Perindopril *tert*-butylamine impurities.

Impurity	*Chemical name*	*Structural formula*
**A**	(2*S*,3a*S*,7a*S*)-octahydro-1*H*-indole-2-carboxylic acid	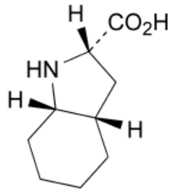
**B**<br>perlat	2*S*,3a*S*,7a*S*)-1-[(2*S*)-2-[[(1*S*)-1-carboxybutyl]amino] propanoyl]octahydro-1*H*-indole-2-carboxylic acid	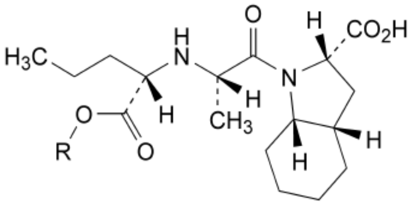 **R=H**
**C**	2*S*)-2-[(3*S*,5a*S*,9a*S*,10a*S*)-3-methyl-1,4-dioxodecahydropyrazino[1,2-*a*]indol-2(1*H*)-yl]pentanoic acid	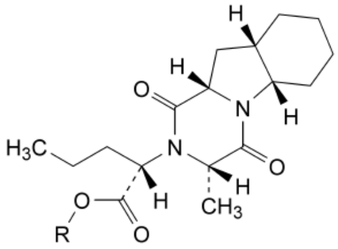 **R=H**
**D**	(2*S*)-2-[(3*S*,5aS,9a*S*,10a*R*)-3-methyl-1,4-dioxodecahydropyrazino[1,2-*a*]indol-2(1*H*)-yl]pentanoic acid	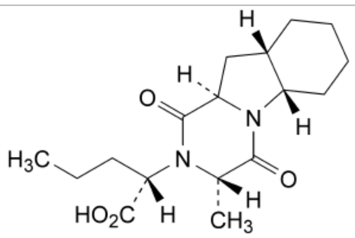
**E**	(2*S*,3a*S*,7a*S*)-1-[(2*S*)-2-[[(1*S*)-1-[(1-methylethoxy) carbonyl]butyl]amino]propanoyl]octahydro-1*H*-indole-	~B

	2-carboxylic acid		**R = $CH(CH_3)_2$**
**F**	ethyl (2*S*)-2-[(3*S*,5a*S*,9a*S*,10a*S*)-3-methyl-1,4-dioxodecahydropyrazino[1,2-*a*]indol-2(1*H*)-yl]pentanoate	~C	**R = C_2H_5**
**G**	(2*S*,3a*S*,7a*S*)-1-[(2*S*)-2-[(5*RS*)-3-cyclohexyl-2,4-dioxo-5-propylimidazolidin-1-yl]propanoyl]octahydro-1*H*-indole-2-<br>carboxylic acid,	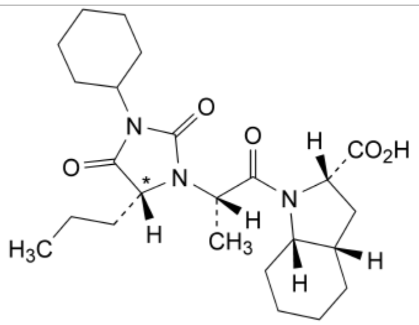	and epimer at C*
**H**	(2S,3aS,7aS)-1-[(2S)-2-[(5RS)-3-cyclohexyl-2-(cyclohexylimino)-4-oxo-5-propylimidazolidin-1-yl]propanoyl]octahydro-1H-indole-2-carboxylic acid	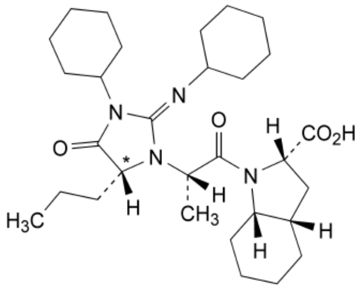	and epimer at C*
**I**	(2*S*,3a*S*,7a*S*)-1-[(2*S*)-2-[[(1*R*)-1-(ethoxycarbonyl)butyl]amino]<br>propanoyl]octahydro-1*H*-indole-2-carboxylic acid	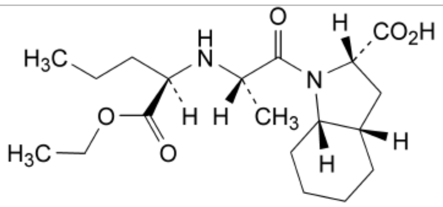	
**J**	(2S,3aS,7aS)-1-[(2S)-2-aminopropanoyl]octahydro-1H-indole-2-carboxylic acid	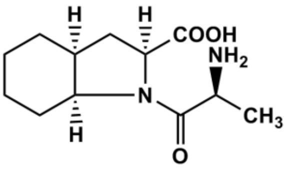	
**K**	(3S,5aS,9aS,10aS)-3-methyldecahydropyrazino[1,2-a]indole-1,4-dione	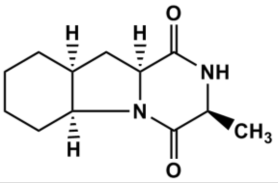	
**L**	(2S,3aS,7aS)-1-acetyloctahydro-1H-indole-2-carboxylic acid	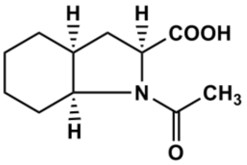	
**M**	(2S,3aS,7aS)-1-[(2S)-2-[[(1S)-1-(methoxycarbonyl)butyl]amino]propanoyl]octahydro-1H-indole-2-carboxylic acid	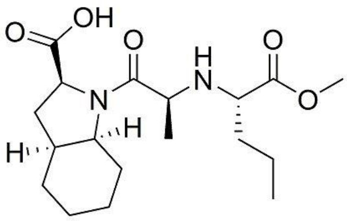	
**N**	(2S)-3-cyclohexyl-2-[[(2S)-2-[[(1S)-1-		

	(ethoxycarbonyl)butyl]amino]propanoyl]amino]propanoic acid	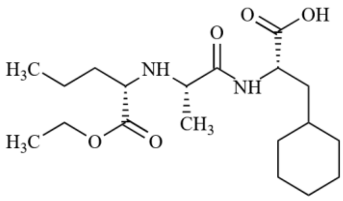
**O**	(2S,3aS,7aS)-1-[[(2S,3aS,7aS)-1-[(2S)-2-[[(1S)-1-(ethoxycarbonyl)butyl]amino]propanoyl]octahydro-1H-indol-2-yl]carbonyl]octahydro-1H-indole-2-carboxylic acid	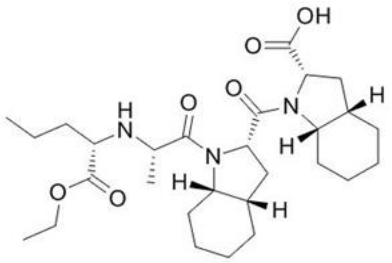
**P**	1-((1-ethoxy-1-oxopentan-2-yl)alanyl)octahydro-1H-indole-2-carboxylic acid	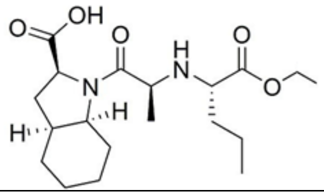

Table S22. ADMET parameters for perindopril-derived compounds.

	**FEFKEI**	**BECWIR**	**IVEGIA**	**UZOVAH03**	Perindopril arginine
**Absorption**					
Water solubilty (log mol/l)	-1.942	-2.325	-2.964	-2.735	-2.835
$CaCO_2$ permeability (log Papp 10^{-6} cm/s)	-0.21	0.03	-0.099	0.189	-0.515
Intestinal absorption (human, % absorbed)	15.219	35.618	30.149	39.969	0
Skin permeability (log Kp)	-2.735	-2.735	-2.735	-2.735	-2.735
P-glycoprotein substrate	no	no	no	no	no
P-glycoprotein I inhibitor	no	no	no	no	no
P-glycoprotein II inhibitor	no	no	no	no	no
**Distribution**					
VDss (human, log L/kg)	-1.129	-0.901	-0.852	-0.515	-0.83
Fraction unbound (human, Fu)	0.736	0.775	0.792	0.752	0.612
BBB permeability (log BB)	-0.526	-0.615	-0.613	-0.601	-1.669
CNS permeability (log PS)	-3.308	-3.225	-3.287	-3.147	-4.403
**Metabolism**					
CYP2D6 substrate	no	no	no	no	yes
CYP3A4 substrate	no	no	yes	yes	yes
CYP1A2 inhibitor	no	no	no	no	no
CYP2C19 inhibitor	no	no	no	no	no
CYP2C9 inhibitor	no	no	no	no	no
CYP2D6 inhibitor	no	no	no	no	no
CYP3A4 inhibitor	no	no	no	no	no
**Excretion**					
Total clearance (log ml/min/kg)	0.766	0.742	0.816	0.889	0.543
Renal OCT2 substrate	no	no	no	no	no
**Toxicity**					
AMES toxicity	no	no	no	no	no
hERG I inhibitor	no	no	no	no	no
hERG II inhibitor	no	no	no	no	no
hepatoxicity	yes	yes	yes	yes	yes
Skin sensitization	no	no	no	no	no

Table S23. ADMET parameters of perindopril impurities.

	**A**	**B**	**C/D**	**E**	**F** (BILNAN)	G	**H**	**I**	**J**
**Absorption**									
Water solubilty (log mol/l)	-2.389	-2.11	-1.478	-2.365	-3.895	-3.036	-2.892	-2.19	-1.172
$CaCO_2$ permeability (log Papp 10^{-6} cm/s)	1.16	-0.103	0.702	0.866	1.112	0.554	0.628	0.769	0.574
Intestinal absorption (human, % absorbed)	86.024	22.064	74.728	47.545	94.961	51.724	60.316	46.184	72.555
Skin permeability (log Kp)	-3.161	-2.735	-2.735	-2.735	-3.67	-2.735	-2.735	-2.735	-2.735

P-glycoprotein substrate	No	no	no	yes	no	No	Yes	No	No
P-glycoprotein I inhibitor	No	no	no	no	no	No	No	No	No
P-glycoprotein II inhibitor	No	no	no	no	no	No	No	No	No
**Distribution**									
VDss (human, log L/kg)	-0.369	-0.89	-0.918	-0.516	0.104	-0.644	-0.315	-0.55	-0.873
Fraction unbound (human, Fu)	0.732	0.613	0.57	0.566	0.461	0.418	0.363	0.668	0.74
BBB permeability (log BB)	-0.248	-1.071	-0.783	-0.934	-0.042	-1.094	-0.977	-0.968	-0.667
CNS permeability (log PS)	-3.002	-3.573	-3.46	-3.041	-3.016	-3.152	-3.109	-3.148	-3.453
**Metabolism**									
CYP2D6 substrate	No	yes	yes	no	no	No	No	No	No
CYP3A4 substrate	No	no	no	yes	yes	Yes	yes	No	No
CYP1A2 inhibitor	No	no	no	no	no	No	No	No	No
CYP2C19 inhibitor	No	no	no	no	no	No	No	No	No
CYP2C9 inhibitor	No	no	no	no	no	No	No	No	No
CYP2D6 inhibitor	No	no	no	no	no	No	No	No	No
CYP3A4 inhibitor	No	no	no	no	no	no	no	No	No
**Excretion**									
Total clearance (log ml/min/kg)	0.535	0.347	1.288	0.546	1.446	0.92	0.309	0.6	0.408
Renal OCT2 substrate	No	no	no	no	no	No	No	No	No
**Toxicity**									
AMES toxicity	No	no	no	no	no	No	No	No	No
hERG I inhibitor	No	no	no	no	no	No	no	No	no
hERG II inhibitor	No	no	no	no	no	No	No	No	No
hepatoxicity	Yes	yes	yes	yes	yes	No	Yes	Yes	Yes
Skin sensitization	no	no	no	no	no	no	no	no	no

	**K**	**L**	**M**	**N**	**O**	**P**			
**Absorption**									
Water solubilty (log mol/l)	-2.413	-1.069	-2.065	-2.606	-3.146	-2.19			
$CacO_2$ permeability (log Papp 10^{-6} cm/s)	1.193	1.203	0.049	0.899	0.571	0.769			
Intestinal absorption (human, % absorbed)	90.094	86.135	45.427	41.888	48.416	46.184			
Skin permeability (log Kp)	-3.851	-2.735	-2.735	-2.735	-2.735	-2.735			
P-glycoprotein substrate	No	No	No	Yes	Yes	No			
P-glycoprotein I inhibitor	No	No	No	No	No	No			
P-glycoprotein II inhibitor	No	No	No	No	No	No			
**Distribution**									
VDss (human, log L/kg)	0.135	-0.664	-0.582	-1.263	-0.516	-0.55			
Fraction unbound (human, Fu)	0.656	0.629	0.691	0.531	0.528	0.668			
BBB permeability (log BB)	-0.165	-0.221	-0.917	-0.968	-1.05	-0.968			
CNS permeability (log PS)	-3.003	-2.985	-3.149	-3.204	-3.204	-3.148			
**Metabolism**									
CYP2D6 substrate	No	No	No	No	No	No			
CYP3A4 substrate	No	No	No	Yes	Yes	No			
CYP1A2 inhibitor	No	No	No	No	No	No			
CYP2C19 inhibitor	No	No	No	No	No	No			
CYP2C9 inhibitor	No	No	No	No	No	No			
CYP2D6 inhibitor	No	No	No	No	No	No			
CYP3A4 inhibitor	No	No	No	No	No	No			
**Excretion**									
Total clearance (log ml/min/kg)	1.188	1.247	0.552	0.675	0.505	0.6			
Renal OCT2 substrate	no	No	No	No	No	No			
**Toxicity**									
AMES toxicity	No	No	No	No	No	No			
hERG I inhibitor	No	No	No	No	No	No			
hERG II inhibitor	No	No	No	No	No	No			
hepatoxicity	No	No	Yes	Yes	Yes	Yes			
Skin sensitization	no	no	no	no	no	no			

Table S24. ADMET parameters for proline-based ACEI compounds.

	**CIYNIH**	**DIVHOF01**	**GERXAE**	**GERXEI**	**GERWUX**01	**MCPRL01**	**TUHMOY**	**TUHMUE**	**YOZTIS**
**Absorption**									
Water solubilty (log mol/l)	-2.62	-2.767	-2.746	-2.736	-2.781	-1.184	-4.061	-2.975	-2.013
$CacO_2$ permeability (log Papp 10^{-6} cm/s)	-0.265	-0.402	-0.429	-0.393	-0.169	**1.174**	**1.16**	0.152	-0.114
Intestinal absorption (human, % absorbed)	**14.485**	**0**	**8.278**	**13.188**	**6.327**	80.006	42.781	**16.895**	**13.88**
Skin permeability (log Kp)	**-2.735**	**-2.735**	**-2.735**	**-2.735**	**-2.735**	**-2.735**	**-2.735**	**-2.735**	**-2.735**
P-glycoprotein substrate	no	**yes**	no	no	no	no	**yes**	**yes**	no
P-glycoprotein I inhibitor	no	no	no	no	no	no	no	no	no
P-glycoprotein II inhibitor	no	no	no	no	no	no	no	no	no
**Distribution**									

VDss (human, log L/kg)	**-2.465**	**-0.612**	**-1.836**	**-1.752**	**-1.832**	**-1.018**	**-1.024**	**-1.298**	**-1.443**
Fraction unbound (human, Fu)	0.519	0.59	0.606	0.582	0.65	0.661	0.001	0.078	0.485
BBB permeability (log BB)	**-0.578**	**-0.979**	**-0.883**	**-0.877**	**-0.306**	**-0.233**	**-0.688**	-1.015	-1.252
CNS permeability (log PS)	-3.963	-4.107	-4.311	-4.088	-4.745	-3.081	**-2.713**	-3.408	-3.321
**Metabolism**									
CYP2D6 substrate	no	no	no	no	no	no	no	no	no
CYP3A4 substrate	no	**yes**	no	no	no	no	**yes**	**yes**	no
CYP1A2 inhibitor	no	no	no	no	no	no	no	no	no
CYP2C19 inhibitor	no	no	no	no	no	no	no	no	no
CYP2C9 inhibitor	no	no	no	no	no	no	no	no	no
CYP2D6 inhibitor	no	no	no	no	no	no	no	no	no
CYP3A4 inhibitor	no	no	no	no	no	no	no	no	no
**Excretion**									
Total clearance (log ml/min/kg)	0.794	0.273	1.164	0.845	**1.213**	0.306	**-0.598**	-0.53	0.329
Renal OCT2 substrate	no	no	no	no	no	no	no	no	no
**Toxicity**									
AMES toxicity	no	no	no	no	no	no	no	no	no
hERG I inhibitor	no	no	no	no	no	no	no	no	no
hERG II inhibitor	no	no	no	no	no	no	no	no	no
hepatoxicity	**yes**	**yes**	**yes**	**yes**	**yes**	no	**yes**	**yes**	**yes**
Skin sensitization	no	no	no	no	no	no	no	no	no

Table S25. ADMET parameters for other modified proline-based ACEI compounds.

	EDALEC	FIFGEG	IQISAE	RUWBAM	QOQWAU
**Absorption**					
Water solubilty (log mol/l)	-2.738	-2.707	-2.685	-2.801	-2.738
$CacO_2$ permeability (log Papp 10^{-6} cm/s)	0.26	0.494	-0.35	0.767	0.26
Intestinal absorption (human, % absorbed)	50.127	**20.479**	**8.581**	**23.493**	50.127
Skin permeability (log Kp)	**-2.735**	**-2.735**	**-2.735**	**-2.735**	**-2.735**
P-glycoprotein substrate	**yes**	**yes**	**yes**	**yes**	**yes**
P-glycoprotein I inhibitor	no	no	no	no	no
P-glycoprotein II inhibitor	no	no	no	no	no
**Distribution**					
VDss (human, log L/kg)	**-0.351**	**-2.259**	**-1.045**	**-0.847**	**-0.351**
Fraction unbound (human, Fu)	0.679	0.403	0.875	0.662	0.679
BBB permeability (log BB)	**-0.693**	-1.29	-1.047	**-0.334**	**-0.693**
CNS permeability (log PS)	-3.032	-3.445	-4.485	-3.993	-3.092
**Metabolism**					
CYP2D6 substrate	no	**yes**	no	no	no
CYP3A4 substrate	**yes**	no	**yes**	**yes**	**yes**
CYP1A2 inhibitor	no	no	no	no	no
CYP2C19 inhibitor	no	no	no	no	no
CYP2C9 inhibitor	no	no	no	no	no
CYP2D6 inhibitor	no	no	no	no	no
CYP3A4 inhibitor	no	no	no	no	no
**Excretion**					
Total clearance (log ml/min/kg)	0.752	**0.466**	**1.269**	1.063	0.752
Renal OCT2 substrate	no	no	no	no	no
**Toxicity**					
AMES toxicity	no	no	no	no	no
hERG I inhibitor	no	no	no	no	no
hERG II inhibitor	no	no	no	no	no
hepatoxicity	**yes**	**yes**	**yes**	**yes**	**yes**
Skin sensitization	no	no	no	no	no

Table S26. SwissADME parameters for proline-based and modified-prolinebased ACEI compounds.

Physicochemical properties

	Mol. weight [g/mol]	*No. heavy atoms*	*No. arom. Heavy atoms*	*Fraction Csp3*	*No. rotatable bonds*	*No. H-bond acceptors*	*No. H-bond donors*	*Molar refractivity*	*TPSA [$Å^2$]*	*% ABS**
FEFKEI	358.43	25	0	0.82	8	6	3	95.32	123.58	66.36
BECWIR	418.55	28	0	0.84	8	6	2	112.28	150.63	57.03
IVEGIA	477.64	33	0	0.87	10	8	4	131.59	144.87	59.02
UZOVIP	459.62	32	0	0.87	10	7	3	128.54	135.64	62.20
UZOVAH	441.60	31	0	0.87	10	6	2	125.49	126.41	65.39

BILNAN01 (impurity F)	350.45	25	0	0.84	6	4	0	102.41	66.92	85.91
Impurity A	169.22	12	0	0.89	1	3	2	49.64	49.33	91.98
Impurity B	340.41	24	0	0.82	8	6	3	92.96	106.94	72.10
Impurity C	308.37	22	0	0.81	4	4	1	88.48	77.92	82.12
impurity E	382.49	27	0	0.85	10	6	2	106.90	95.94	75.90
Impurity G	447.57	32	0	0.83	7	5	1	132.22	98.23	75.11
Impurity H	528.73	38	0	0.87	8	5	1	162.04	93.52	76.74
Impurity I	368.47	26	0	0.84	10	6	2	102.09	95.94	75.90
Impurity J	240.30	17	0	0.83	3	4	2	67.06	83.63	80.15
Impurity K	222.28	16	0	0.83	0	2	1	67.38	49.41	91.95
**Impurity L**	211.26	15	0	0.82	2	3	1	59.55	57.61	89.12
**Impurity M**	354.44	25	0	0.83	9	6	2	97.28	95.94	75.901
**Impurity N**	370.48	26	0	0.84	13	6	3	100.19	104.73	72.87
**Impurity O**	519.67	37	0	0.86	12	7	2	148.13	116.25	68.89
**Impurity P**	368.47	26	0	0.84	10	6	2	102.09	95.94	75.901
Perindopril arginine	542.67	38	0	0.80	16	11	6	145.94	225.61	31.17

*% ABS = 109 – (0.345 · TPSA)

	Mol. weight [g/mol]	*No. Heavy atoms*	*No. Arom. Heavy atoms*	*Fraction Csp3*	*No. rotatable bonds*	*No. H-bond acceptors*	*No. H-bond donors*	*Molar refractivity*	*TPSA [Å^2]*	*% ABS*
**EDALEC**	550.66	39	6	0.68	14	9	5	147.60	187.10	44.45
**FIFGEG**	434.53	31	6	0.61	9	7	4	120.72	127.17	65.12
**IQISAE**	416.51	30	6	0.61	10	6	2	116.96	95.94	75.90
**RUWBAM**	521.09	33	6	0.59	11	6	3	137.53	160.35	53.68
**QOQWAU**	416.51	30	6	0.61	11	6	2	116.96	95.94	75.90

	Mol. weight [g/mol]	*No. Heavy atoms*	*No. Arom. Heavy atoms*	*Fraction Csp3*	*No. rotatable bonds*	*No. H-bond acceptors*	*No. H-bond donors*	*Molar refractivity*	*TPSA [Å^2]*	*% ABS*
**CIYNIH**	366.41	26	6	0.50	9	6	3	97.89	123.58	66.36
**DIVHOF01**	492.52	35	6	0.46	13	9	3	128.38	177.95	47.61
**GERXAE**	423.50	30	6	0.57	13	7	4	115.02	146.64	58.41
**GERXEI**	405.49	29	6	0.57	13	6	3	111.97	137.41	61.6
**GERWUX01**	423.50	30	6	0.57	13	6	3	114.33	154.33	55.76
**MCPRL01**	217.29	14	0	0.78	4	3	1	59.97	96.41	75.74
**TUHMOY**	585.64	40	6	0.70	16	7	0	155.62	122.85	66.62
**TUHMUE**	881.09	59	24	0.32	18	8	1	239.35	253.39	21.58
**YOZTIS**	432.55	28	0	0.78	11	6	2	117.16	165.82	51.79

Lipophilicity / Water Solubility

	Log Po/w (iLogP)	*Log Po/w (XLogP3)*	*Log Po/w (WLogP)*	*Log Po/w (MLogP)*	*Log Po/w (SILICOS-IT)*	*Consensus Log Po/w*	*Log S (ESOL)*	*Log S (Ali)*	*Log S (SILICOS-IT)*
**FEFKEI**	2.08	-0.26	-1.34	-3.59	0.72	-0.48	-1.37 Very sol.	-1.88 Very sol.	-1.14 Sol.
**BECWIR**	2.76	-0.40	-0.42	-3.21	0.72	-0.11	-1.65 Very sol.	-2.30 Sol.	-1.14 Sol.
**IVEGIA**	4.37	0.23	0.12	-3.78	1.68	0.52	-2.29 Sol.	-2.83 Sol.	-2.23 Sol.
**UZOVIP**	4.27	0.70	0.19	-3.01	1.68	0.77	-2.47 Sol.	-3.13 Sol.	-2.23 Sol.
**UZOVAH**	4.37	1.18	0.25	-2.23	1.68	1.05	-2.66 Sol.	-3.43 Sol.	-2.23 Sol.
**BILNAN01 (impurity F)**	3.51	2.91	1.35	1.75	1.80	2.26	-3.45 Sol.	-3.98 Sol.	-2.53 Sol.
**Impurity A**	1.46	-1.01	0.61	-1.21	0.80	0.13	-0.19	0.46	-0.83
**Impurity B**	2.10	0.21	1.08	-1.33	0.72	0.55	-1.55 Very sol.	-2.01 Sol.	-1.14 Sol.
**Impurity C**	2.30	1.81	0.48	1.04	0.76	1.28	-2.63 Sol.	-3.07 Sol.	-1.29 Sol.
**impurity E**	3.34	1.34	1.95	1.58	1.92	2.03	-2.40 Sol.	-2.96 Sol.	-2.25 Sol.
**Impurity G**	3.58	3.82	2.24	2.38	1.50	2.70	-4.56	-5.58	-2.06

							Moder. Sol.	Moder. Sol.	Sol.
**Impurity H**	4.35	5.71	3.81	3.58	3.36	4.16	-8.19	-7.44	-3.55
**Impurity I**	2.93	0.91	1.56	1.36	1.68	1.69	-2.04	-2.51	-2.23
**Impurity J**	1.39	-1.18	0.20	0.50	-0.10	0.18	-0.39 Very sol.	-0.08 Very Sol.	-0.15 Sol.
**Impurity K**	2.08	1.15	-0.10	0.92	0.71	0.95	-1.94	-1.78	-1.57
**Impurity L**	1.70	1.61	0.87	1.05	0.56	1.16	-2.03 Sol.	-2.43 Sol.	-0.46 Sol.
**Impurity M**	2.39	0.54	1.17	1.12	1.27	1.30	-1.78 Very sol.	-2.14 Sol.	-1.83 Sol.
**Impurity N**	3.47	1.56	2.24	1.36	2.59	2.24	-2.26 Sol.	-3.37 sol.	-3.30 Sol.
**Impurity O**	3.84	2.31	2.34	2.01	2.24	2.55	-3.73 Sol.	-4.39 Moder. Sol.	-2.89 Sol.
**Impurity P**	2.93	0.91	1.56	1.36	1.68	1.69	-2.04 Sol.	-2.51 Sol.	-2.23 Sol.
**Perindopril arginine**	2.40	-2.91	-1.83	-5.99	1.68	-1.33	-0.32 Very sol.	-1.27 Very sol.	-2.23 Sol.

	Log Po/w (iLogP)	*Log Po/w (XLogP3)*	*Log Po/w (WLogP)*	*Log Po/w (MLogP)*	*Log Po/w (SILICOS-IT)*	*Consensus Log Po/w*	*Log S (ESOL)*	*Log S (Ali)*	*Log S (SILICOS-IT)*
**EDALEC**	4.69	-0.91	-2.18	-3.78	2.82	0.13	-1.87 Very sol.	-2.54 Sol.	-4.30 Mod. Sol.
**FIFGEG**	3.17	0.77	1.52	-1.02	1.86	1.26	-2.57 Sol.	-3.02 Sol.	-3.22 Sol.
**IQISAE**	3.24	1.61	2.00	1.98	2.42	2.25	-2.92 Sol.	-3.24 Sol.	-3.91 Sol.
**RUWBAM**	0.00	1.20	-2.08	-2.49	3.06	-0.06	-3.24 Sol.	-4.16 Mod. Sol.	-4.38 Mod. Sol.
**QOQWAU**	3.06	1.43	2.00	1.98	2.57	2.21	-2.75 Sol.	-3.05 Sol.	-4.04 Mod. Sol.

	Log Po/w (iLogP)	*Log Po/w (XLogP3)*	*Log Po/w (WLogP)*	*Log Po/w (MLogP)*	*Log Po/w (SILICOS-IT)*	*Consensus Log Po/w*	*Log S (ESOL)*	*Log S (Ali)*	*Log S (SILICOS-IT)*
**CIYNIH**	1.79	-1.21	-1.68	-3.61	1.26	-0.69	-0.93 Very sol.	-0.89 Very sol.	-2.62 Sol.
**DIVHOF01**	3.51	-0.51	-1.43	-3.20	2.22	0.12	-1.84 Very sol.	-2.76 Sol.	-3.70 Sol.
**GERXAE**	2.17	-3.33	-1.26	-5.92	1.65	-1.34	0.34 Highly sol.	0.82 Highly sol.	-3.44 Sol.
**GERXEI**	2.26	-2.86	-1.20	-5.14	1.65	-1.06	0.15 Highly sol.	0.53 Highly sol.	-3.44 Sol.
**GERWUX01**	2.10	-3.33	-3.62	-7.39	1.65	-2.12	0.34 Highly sol.	0.67 Highly sol.	-3.44 Sol.
**MCPRL01**	1.46	0.34	0.25	0.45	0.61	0.62	-1.14 Very sol.	-1.93 Very sol.	-0.38 Sol.
**TUHMOY**	-9.37	6.24	4.41	3.74	4.76	1.95	-6.48 Poorly sol.	-8.61 Poorly sol.	-6.02 Poorly sol.
**TUHMUE**	-8.56	8.75	5.99	4.15	3.45	2.76	-9.93 Poorly sol.	-13.95 Insol.	-5.13 Mod. Sol.
**YOZTIS**	2.14	1.15	1.03	0.65	1.09	1.21	-2.52 Sol.	-4.23 Mod. Sol.	-0.75 Sol.

Pharmacokinetics

	GI absorption	*BBB permeant*	*P-gp substrate*	*CYP1A2 inhibitor*	*CYP2C19 inhibitor*	*CYP2C9 inhibitor*	*CYP2D6 inhibitor*	*CYP3A4 inhibitor*	*Log Kp [cm/s]*
**FEFKEI**	high	no	yes	no	no	no	no	no	-8.67
**BECWIR**	low	no	yes	no	no	no	no	no	-9.14
**IVEGIA**	low	no	yes	no	no	no	no	no	-9.05
**UZOVIP**	high	no	yes	no	no	no	no	no	-8.61
**UZOVAH**	high	no	yes	no	no	no	no	no	-8.16
**BILNAN01 (impurity F)**	high	yes	no	no	no	no	no	no	-6.37
**Impurity A**	high	yes	no	no	no	no	no	no	-8.05
**Impurity B**	high	no	yes	no	no	no	no	no	-8.23

**Impurity C**	high	no	no	no	no	no	no	no	-6.90
**impurity E**	high	no	yes	no	no	no	no	no	-7.68
Impurity G	high	no	no	no	no	yes	no	yes	-6.32
**Impurity H**	high	no	no	no	no	yes	no	yes	-5.47
**Impurity I**	high	no	yes	no	no	no	no	no	-7.90
**Impurity J**	high	no	no	no	no	no	no	no	-8.60
**Impurity K**	high	no	no	no	no	no	no	no	-6.84
**Impurity L**	high	yes	no	no	no	no	no	no	-6.45
**Impurity M**	high	no	yes	no	no	no	no	no	-8.08
**Impurity N**	high	no	yes	no	no	no	no	no	-7.45
**Impurity O**	high	no	no	no	no	no	no	yes	-7.83
**Impurity P**	high	no	yes	no	no	no	no	no	-7.90
**Perindopril arginine**	low	no	no	no	no	no	no	no	-11.68

	GI absorption	*BBB permeant*	*P-gp substrate*	*CYP1A2 inhibitor*	*CYP2C19 inhibitor*	*CYP2C9 inhibitor*	*CYP2D6 inhibitor*	*CYP3A4 inhibitor*	*Log Kp [cm/s]*
**EDALEC**	low	now	yes	no	no	no	no	no	-10.31
**FIFGEG**	high	no	yes	no	no	no	no	no	-8.40
**IQISAE**	high	no	yes	no	no	no	yes	yes	-7.70
**RUWBAM**	low	no	yes	no	no	no	no	no	-8.63
**QOQWAU**	high	no	yes	no	no	no	yes	yes	-7.83

	GI absorption	*BBB permeant*	*P-gp substrate*	*CYP1A2 inhibitor*	*CYP2C19 inhibitor*	*CYP2C9 inhibitor*	*CYP2D6 inhibitor*	*CYP3A4 inhibitor*	*Log Kp [cm/s]*
**CIYNIH**	low	no	no	no	no	no	no	no	-9.39
**DIVHOF01**	low	no	no	no	no	no	no	no	-9.67
**GERXAE**	low	no	no	no	no	no	no	no	-11.25
**GERXEI**	low	no	no	no	no	no	no	no	-10.80
**GERWUX01**	low	no	no	no	no	no	no	no	-11.25
**MCPRL01**	high	no	no	no	no	no	no	no	-7.38
**TUHMOY**	low	no	yes	no	no	no	no	no	-5.44
**TUHMUE**	low	now	yes	no	no	no	no	no	-5.46
**YOZTIS**	low	no	yes	no	no	no	no	no	-8.12

Drug-likeness & Medicinal Chemistry

	Lipinski	*Ghose*	*Veber*	*Egan*	*Muegge*	*Bioavailability score*	*Leadlikeness*	*Synthetic accessibility*
**FEFKEI**	Yes, 0	No, 1	yes	yes	yes	0.56	No, 2	3.96
**BECWIR**	Yes, 0	No, 1	No, 1	No, 1	No, 1	0.11	No, 2	4.25
**IVEGIA**	Yes, 0	No, 2	No, 1	No, 1	yes	0.55	No, 2	5.07
**UZOVIP**	Yes, 0	No, 1	yes	No, 1	yes	0.56	No, 2	4.95
**UZOVAH**	Yes, 0	No, 1	yes	yes	yes	0.55	No, 2	4.83
**BILNAN01 (impurity F)**	Yes,0	yes	yes	yes	yes	0.55	No, 1	4.12
**Impurity A**								
**Impurity B**	Yes, 0	yes	yes	yes	yes	0.56	No, 1	3.85
**Impurity C/D**	Yes, 0	yes	yes	yes	yes	0.56	yes	3.52
**impurity E**	Yes, 0	yes	yes	yes	yes	0.55	No, 2	4.31
Impurity G	Yes, 0	No, 1	yes	yes	yes	0.56	No, 2	4.77
**Impurity H**	Yes, 1	No, 3	yes	yes	No, 1	0.55	No, 3	6.03
**Impurity I**	Yes, 0	yes	yes	yes	yes	0.55	No, 2	4.20
**Impurity J**	Yes, 0	yes	yes	yes	yes	0.55	No, 1	3.09
**Impurity K**	Yes, 0	yes	yes	yes	yes	0.55	No, 1	2.96
**Impurity L**	Yes, 0	yes	yes	yes	yes	0.56	No, 1	2.71
**Impurity M**	Yes, 0	yes	yes	yes	yes	0.55	No, 2	4.00
**Impurity N**	Yes, 0	yes	No, 1	yes	yes	0.55	No, 2	3.98
**Impurity O**	Yes, 1	No, 3	No, 1	yes	yes	0.55	No, 2	5.48
**Impurity P**	Yes, 0	yes	yes	yes	yes	0.56	No, 2	4.20
**Perindopril arginine**	No, 3	No, 4	No, 2	No, 1	No, 5	0.17	No, 2	5.49

	Lipinski	*Ghose*	*Veber*	*Egan*	*Muegge*	*Bioavailability score*	*Leadlikeness*	*Synthetic accessibility*

**EDALEC**	No, 2	No, 4	No, 2	No, 1	No, 1	0.17	No, 2	5.10
**FIFGEG**	Yes, 0	yes	yes	yes	yes	0.56	No, 2	4.18
**IQISAE**	Yes, 0	yes	yes	yes	yes	0.55	No, 2	4.23
**RUWBAM**	Yes, 1	No, 3	No, 2	No, 1	No, 1	0.11	No, 2	5.15
**QOQWAU**	Yes, 0	yes	No, 1	yes	yes	0.55	No, 2	4.23

	Lipinski	*Ghose*	*Veber*	*Egan*	*Muegge*	*Bioavailability score*	*Leadlikeness*	*Synthetic accessibility*
**CIYNIH**	Yes, 1	No, 2	No, 2	No, 1	No, 1	0.11	No, 2	4.22
**DIVHOF01**	Yes, 0	yes	yes	yes	yes	0.56	No, 1	2.47
**GERXAE**	Yes, 0	No, 1	No, 2	No, 1	No, 1	0.55	No, 2	3.78
**GERXEI**	Yes, 0	No, 1	No, 1	No, 1	No, 1	0.55	No, 2	3.67
**GERWUX01**	Yes, 0	No, 1	No, 2	No, 1	No, 2	0.55	No, 2	3.78
**MCPRL01**	Yes, 0	No, 1	yes	yes	yes	0.56	No, 2	3.31
**TUHMOY**	Yes, 1	No, 3	No, 1	yes	No, 2	0.55	No, 3	6.05
**TUHMUE**	No, 2	No, 4	No, 2	No, 2	No, 4	0.11	No, 3	6.67
**YOZTIS**	Yes, 0	yes	No, 2	No, 1	No, 1	0.11	No, 2	4.18

***REFERENCES** (*vide* Tab. S1):

*Aakeroy, C.B.; Bahra, G.S.; Brown, C.R.; Hitchcock, P.B.; Patell, Y.; Seddon, K.R. *Acta Chem. Scand*. **1995**, 49, 762.

*Abdaoui, M.; Dewynter, G.; Toupet, L.; Montero, J.L. *Tetrahedron*. **2000**, 56, 2427.

*Albrecht, M.; Hai, Y.; Koksal, O.; Raabe, G.; Pan, F.; Valkonen, A.; Rissanen, K. *Chem. Eur. J.* **2016**, 22, 6596.

*Almansa, C.; Merce, R.; Tesson, N.; Farran, J.; Tomas, J.; Plata-Salaman, C.R. CSD Communication (Private Communication). **2017**.

*Ambika, R. CSD Communication (Private Communication). **2017**.

*Andersen, T.P.; Rasmusse, P.B.; Thomsen, I.; Lawesson, S.O.; Jorgensen, P.; Lindhardt, P. *Liebigs Ann*. **1986**, 269.

*Arman, H.D.; Kaulgud, T.; Tiekink, E.R.T. *Acta Crystallogr. E.* **2009**, 65, 02950.

*Athimoolan, S.; Natarajan, S. *Acta Crystallogr. C.* **2007**, 63, o283.

*Aubry, A.; Bayeul, D.; Mangeot, J.P.; Vidal, J.; Sterin, S.; Collet, A.; Lecoq, A.; Marraud, M. X. *Biopolymers*. **1991**, 31, 793.

*Babor, M.; Nievergelt, P.P.; Cejka, J.; Zvonicek, V.; Springler, B. *IUCrJ*, **2019**, 6, 145.

*Bakonyi, B.; Furegati, M.; Kramer, C.; La Vecchia, L.; Ossola, F. *J. Org. Chem*., **2013**, 78, 9328.

*Baldwin, J.E.; Hulme, C.; Edwards, A.J.; Schofield, C.J.; Parkes, K.E.B. *Tetrahedron Lett.* **1993**, 34, 1665.

*Bando, S.; Tanaka, N.; Ahida, T.; Kakudo, M. *Acta Cryst. B*. **1978**, 34, 3447.

*Basak, A.; Ghosh, S.C.; Das, A.K.; Bertolasi, V. Org. *Biomol. Chem.* **2005**, 3, 4050.

*Baert, F.; Lobry, M.; Warin, V. C.R. *Acad. Sci. C*, **1975,** 280, 743.

*Baird, P.D.; Dho, J.C.; Fleet, G.W.J.; Peach, J.M.; Prout, K.; Smith, P.W. *J.Chem. Soc., Perkin Trans.* 1. **1987**, 1785.

*Baldwin, J.E.; Bamford, S.J.; Fryer, A.M.; Rudolph, M.P.W.; Wood, M.E. *Tetrahedron*. **1997**, 53, 5255.

*Barker, E.C.; Goh, C.Y.; Jones, F.; Mocerino, M.; Skelton, B.W.; Becker, T.; Ogden, M.I. *Chemical Science*, **2015**, 6, 6133.

*Baumann, M.; Baxendale, I.R. *J. Org. Chem*. **2016**, 81, 11898.

*Bavoso, A.; Benedetti, E.; Di Blasio, B.; Pavone, V.; Toniolo, C.; Bonona, G.M. *Macromolecules*. **1982**, 15(1), 54.

*Bedekar, A.; Muller, G.; Mothuku, S.S.; Mande, H.; Tovar, R. *Tetrahderon Lett.* **2014**, 55, 1760.

*Bell, E.A.; Quereshi, M.Y.; Pryce, R.J.; Janzen, D.H.; Lemke, P.; Clardy, J. *J. Am. Chem. Soc*. **1980**, 102, 1409.

*Bendzinska-Berus, W.; Jelecki, M.; Kwit, M.; Rychlewska, U. *CrystEngComm* **2019**, 21, 3420.

*Benedetti, E.; Ciajolo, M.R.; Maisto, M.R. *Acta Crystallogr. B*. **1974**, 30, 1783.

*Benedetti, E.; Bonora, G.M.; Toniolo, C. *Macromolecules*. **1976**, 9(3), 417.

*Benedetti, E.; Ciajolo, A.; di Blasio, B.; Pavone, V.; Pedone, C.; Toniolo, C.; Bonora, G.M. *Int. J. Pept. Protein Res.* **1979**, 14, 130.

*Bernardi, F.; Garavelli, M.; Scatizzi, M.; Tomasini, C.; Trigari, V.; Crisma, M.; Formaggio, F.; Peggion, C.; Toniolo, *C. Chem. Eur. J.* **2002**, 8, 2516.

*Best, D.; Jenkinson, S.F.; Thompson, A.L.; Watkin, D.J.; Wilson, F.X.; Nash, R.J.; Fleet, G.W.J. …. Acta Crystallogr. E, 2009, 65, o2418-…

*Bhuyan, B.J.; Mugesh, G. *Org. Biomol. Chem.* **2011**, 9, 1356.

*Bialonska, A.; Ciunik, Z. *CrystEngComm* **2006**, 8, 66.

*Bien, J.T.; Desphande, P.P.; Dimarco, J.D.; Gougoutas, J.Z.; Grosso, J.A.; Lai, C.; Lobinger, H.; Nirschi, A.A.; Ramakrishnan, S.; Riebel, P.; Singh, J.; Wang, C. *US Patents* **2009**.

*Blankley, C.J.; Kaltenbronn, J.S.; DeJohn, D.E.; Werner, A.; Bennett, L.R.; Bobowski, G.; Krolls, U.; Johnson, D.R.; Pearlman, W.M.; Hoefle, M.L.; Essenburg, A.D.; Cohen, D.M.; Kaplan, H.R. *J. Med. Chem*. **1987**, 30, 992.

*Bowler, A.N.; Doyle, P.M.; Hitchcock, P.B.; Young, D.W. *Tetrahedron* **1997**, 53, 10545.

*Bunch, L.; Lijefors, T.; Greenwood, J.R.; Frydenvang, K.; Brauner-Osborne, H.; Krogsgaard-Larsen, P.; Madsen, U. *J. Org. Chem.* 2003, 68, 1489.

*Busetti, D.A.; Granozzi, G. *Tetrahedron*. **1982**, 38(22), 3329.

*Camus, F.; Norberg, B.; Bourry, A.; Rigo, B.; Durant, F. *Acta Crystallogr. E*, **2001**, 57, o439.

*Carreras, J.; Avenoza, A.; Busto, J.H.; Peregrina, J.M. *J. Org. Chem*. **2011**, 76(9), 3381.

*Chandan, N.; Moloney, M.G. *Org. Biomol. Chem.* **2008**, 6, 3664.

*Chandrarekha, M.; Krishnakumar, R.V.; Srinivasan, N. *CSD Communication* (Private Com.), 2016.

*Chesna, A.M.; Cox, J.M.; Basso, S.; Benedict, J.B. *Acta Crystallogr. E*, **2017**, 73, 369.

*Cantrell, B.E.; Zimmerman, D.M.; Monn, J.A.; Kamboj, R.K.; Hoo, K.H.; Tizzano, J.P.; Pullar, I.A.l Farrell, L.N.; Bleakman, D. *J. Med. Chem.* **1996**, 39, 3617.

*Chen, J.; Sheng, T.; Hu, S.; Xiang, S.; Fu, R.; Zhu, Q.; Wu, X. *J. Solid State Chem.* **2012**, 192, 255.

*Cheng, W.C.; Liu, Y.; Wong, M.; Olmstead, M.M.; Lam, K.S.; Kurth, M.J. *J. Org. Chem*. **2002**, 67, 5673.

*Cini, E.; Giorgi, G.; Rodriguez, M.; Taddei, M. *Synlett* **2009**, 1562.

*Ciunik, Z.; Glowiak, T. *Acta Crystallogr. B*. **1981**, 37, 693.

*Coggon, P. *J. Chem. Soc. B* **1970**, 838.

*Colapietro, M.; De Santis, P.; Nocilli, F.; Palleschi, A.; Spagna, R. *Acta Crystallogr. C.* **1985**, 41, 126.

*Colapietro, M.; de Santis, P.; Palleschi, A.; Spagna, R. *Biopolymers.* 1**986**, 25, 2227.

*Cruishank, D.W.J. *Acta Crystallogr*. **1959**, 12, 1052.

*Dai, H.F.; Chen, W.X.; Zhao, L.; Xiong, F.; Sheng, H.; Chen, F.E. *Adv. Synth. Catal.* **2008**, 350, 1635.

*Dai, J.; Wen, X.C. *Acta Crystallogr. E*. **2008**, 64, o1960.

*Dank, C.; Wurzer, R.; Felsinger, S.; Bugl, R.; Kahlig, H.; Hela, W.; Roller, A.; Gstach, H. *Molecules* **2020**, 25, 449.

*Davies, S.G.; Fletcher, A.M.; Lindsdall, S.M.; Roberts, P.M.; Thomson, J.E. *Org. Lett.* **2018**, 20, 4135.

*Delgado, G.E.; Sijas, L.E.; Mora, A.J.; Gonzales, T.; Briceno, A. *J. Chem. Cryst.* **2012**, 42, 388.

*Del Valle, J.R.; Goodman, M. *Angew. Chem. Int. Ed*. **2002**, 41, 1600.

*De Poli, M.; Moreto, A.; Crisma, M.; Peggion, C.; Formaggio, F.; Kaptein, B.; Broxterman, Q.B.; Toniolo, C. *Chem. Eur. J.* **2009,** 15, 8015.

*Desphande, P.P.; Singh, J.; Pullockaran, A.; Kissick, T.; Elsworth, B.A.; Gougoutas, J.Z.; Dimarco, J.; Fakes, M.; Reyes, M.; Lai, C.; Lobinger, H.; Denzel, T.; Ermann, P.; Crispino, G.; Randazzo, M.; Gao, Z.; Randazzo, R.; Lindrud, M.; Ross, V.; Buono, F.; Doubleday, W.W.; Leung, S.; Richberg, P.; Hughes, D.; Washburn, W.N.; Meng, W.; Mueller, R.J. *Org. Process Ress. Dev.* **2012**, 16, 577.

*Dick, M.; Sarai, N.S.; Martynowycz, M.W.; Gonen, T.; Arnold, F.H. *J. Am. Chem. Soc.* **2019**, 141, 19817.

*Dijnovic-Carugo, K.; Golic, L.; Leban, I.; Svete, J.; Stanovnik, B.; Tisler, M.; Tate, C. *Acta Crystallogr. C.* **1994**, 50, 239.

*Dittrich, B.; Strumpel, M.; Schafer, M.; Spackman, M.A.; Koritsanszky, T. *Acta Crystallogr.* **2006**, 62, 217.

*Dobrova, A.; Platzer, S.; Bacher, F.; Milunovic, M.N.M.; Dobrov, A.; Spengler, G.; Enyedy, E.A.; Novitchi, G.; Arion, V.B. *Dalton Trans.* **2016**, 45, 13427.

*Doi, M.; Nishi, Y.; Kritoshi, N.; Iwata, T.; Nago, M.; Nakano, H.; Uchiyama, S.; Nakazawa, T.; Kobayashi, Y. Simple and efficient syntheses of Boc- and Fmoc-protected 4®- and 4(S)-fluoroproline solely from 4(R)-hydroxyproline. *Tetrahedron.* **2002**, 58, 8453.

*Dong, C.M.; Chen, L.G.; Duan, X.M.; Shu, X.G.; Zeng, T.; Li, Y. *Acta Crystallogr. E.* **2005**, 61, o960.

*Donohoe, T.J.; O`Riordan, T.J.C.; Peifer, M.; Jones, C.R.; Miles, T.J. *Org. Lett.* **2012**, 14, 5460.

*Duanxiu, L.; Kong, M.; Li, J.; Deng, Z.; Zhang, H. *CrystEngComm.* **2018**, 20, 5112.

*Duggan, H.M.E.; Hitchcock, P.B.; Young, D.W. *Org. Biomol. Chem.* **2005**, 3, 2287.

*Dupont, L.; Dideberg, O.; Welter, A. *Acta Crystallogr. B* **1975**, 31, 1018.

*Dupont, L.; Lamotte, J.; Campsteyrn, H.; Vermeire, M.; Welter, A. *Acta Crystallogr. B* **1978**, 34, 850.

*Eberle, M.K.; Brzechffa, L.; Kahle, G.G.; Talati, S.; Weber, H.P. *J. Org. Chem.* **1980**, 45, 3143.

*Eckle, E.; Stezowski, J.J.; Hughes, R.E. *Acta Crystallogr. A*, **1978**, 34, S74.

*Edgeley, D.S.; Martinez, R.F.; Jenkinson, S.F.; Nash, R.J.; Fleet, G.W.J.; Thompson, A.L. *Acta Crystallogr. E* **2012**, 68, o2865.

*Emge, T.J.; Strickland, L.C.; Perkins, C.M. *Acta Crystallogr. C,* **1990**, 46, 286.

*Eustache, J.; Van de Weghe, P.; Le Nouen, D.; Uyehara, H.; Kabuto, C.; Yamamoto, Y. *J. Org. Chem.* **2005**, 70, 4043.

*Ficker, R.; Hiller, W.; Krauss, S.; Voelter, W. *Z .Krist. Cryst. Mater.* **1995**, 210, 960.

*Finke, P.E.; Shah, S.K.; Ashe, B.M.; Ball, R.G.; Blackblock, T.J.; Bonney, R.J.; Brause, K.A.; Chandler, G.O.; Cotton, M.; Davies, P.; Dellea, P.S.; Dorn Junior, C.P.; Fletcher, D.S.; O`Grady, L.A.; Hagmann, W.K.; Hand, K.M.; Knight, W.B.; Maycock, A.L.; Mumford, R.A.; Osinga, D.G.; Sohar, P.; Thompson, K.R.; Weston, H.; Doherty, J.B. *J. Med. Chem.* **1992**, 35, 3731.

*Flaig, R.; Koritsanszky, T.; Dittrich, B.; Wagner, A.; Luger, P. *J. Am. Chem. Soc.* **2002**, 124, 3407.

*Fleck, M.; Ghazaryan, V.V.; Petrosyan, A.M. *J. Mol. Struct.* **2015**, 1079, 460.

*Fleischhacker, W.; Riedl, T.; Vollenkle, H.; Noe, C.R. *Arch. Pharm. Chem. Life Sci.* **1996**, 329, 41.

*Flippen, J.L.; Gilardi, R.D. *ACS* **1976**, 73a.

*Foley, D.J.; Doveston, R.G.; Churcher, I.; Nelson, A.; Marsden, S.P. *Chem. Commun.* **2015**, 51, 11174.

*Foletti, C.; Trapp, N.; Loosli, S.; Lewandowski, B.; Wennemers, H. *Helv. Chim. Acta* **2019**, 102, e1900052.

*Fonari, M.S.; Chumakov, Y.M.; Duca, G.G.; Ganin, E.V.; Yavolovskii, A.A.; Lis, T.; Simonov, Y.A. *Tetrahedron,* **2006**, 62, 8779.

*Forbes, C.R.; Pandey, A.K.; Ganguly, H.K.; Yap, G.P.A.; Zondlo, N.J. *Org. Biomol. Chem.* **2016**, 14, 2327.

*Frebault, F.; Luparai, M.; Oliveira, M.T.; Goddard, R.; Maulide, N. *Angew. Chem. Int.* **2010**, 49, 5672.

*Fridrichsons, J.; McL.Mathieson, M. *Acta Crystallogr.,* **1962**, 15, 569.

*Fu, T.Y.; Scheffer, J.R.; Trotter, J. *Acta Crystallogr. C.* **1997**, 53, 1259.

*Fujisawa, I.; Kitamura, Y.; Kato, R.; Aoki, K. *J. Mol. Struct***. 2018**, 1163, 336.

*Fujimoto, Y.; Irreverre, F.; Karle, J.; Karle, I.L; Witkop, B. *J. Am. Chem. Soc.* **1971**, 93, 3471.

*Galitskii, N.M.; Deigin, V.I.; Saenger, W.; Pletnev, V.Z. *Bioorg. Khin.* **1977**, 3, 1445.

*Garbay-Jaureguiberry, C.; Arnoux, B.; Prange, T.; Wehri-Altenburger, S.; Pascard, C.; Roques, B.P. X-ray and NMR studies of L-4-hydrozyproline conformation in oligopeptides related to collagen. J. Am. Chem. Soc. 1980, 102, 1827-1837.

*Gharzaryan, V.V.; Fleck, M.; Petrosyan, A.M. Proc. of SPIE – *Int. Conf. Las. Phys*. **2011**, 7998, 79980F-..

*Ghazaryan, V.V.; Fleck, M.; Petrosyan, A.M. *J. Cryst. Growth* **2014**, 401, 857.

*Guenoun, F.; Zair, T.; Lamaty, F.; Pierrot, M.; Lazaro, R.; Viallefont, P. *Tetrahedron Lett.,* **1997**, 38, 1563.

*Hack, V.; Reuter, C.; Opitz, R.; Schmieder, P.; Beyermann, M.; Neudorfl, J.M.; Kuhne, R.; Schmalz, H.G. *Angew. Chem. Int.* **2013**, 52, 9539.

*Hammarstrom, L.G.J.; McLaughin, M.L.; Fronczek, F.R. *CSD Comuunication (Private Communication).* **2014.**

*Hanessian, S.; Reinhold, U.; Gentle, G. *Angew. Chem.,* **1997**, 36, 1881.

*Hanessian, S.; Sailes, H.; Therrien, E. *Tetrahedron.* **2003**, 59, 7047-7056.

*Hanessian, S.; Papeo, G.; Angiolini, M.; Fettis, K.; Beretta, M.; Munro, A. *J. Org. Chem*. **2003**, 68, 7204.

*Hanessian, S.; Shao, Z.; Warrier, J.S. *Org. Lett.* **2006**, 8, 4787.

*Hausin, R.J.; Codding, P.W. *J. Med. Chem.* **1990**, 33, 1940.

*He, H.; Huang, Y.; Zhang, Q.; Wang, J.R.; Mei, X. *Cryst. Growth Des.* **2016**, 16, 2348.

*He, H.; Zhang, Q.; Li, M.; Wang, J.R.; Mei, X. *Cryst. Growth Des.* **2017**, 17, 3989.

*Herdeis, C.; Aschenbrenner, A.; Kirfel, A.; Schwabenlander, F. *Tetrahedron: Asymm*. **1997**, 8, 2421.

*Hernandez-Paredas, J.; Olvera-Tapia, A.L.; Arenas-Garcia, J.I.; Hopfl, H.; Morales-Rojas, H.; Herrera-Ruiz, D.; Gonzaga-Morales, A.I.; Rodriguez-Fragoso, L. *CrystEngComm*. **2015**, 17, 5166.

*Hobart Junior, D.B.; Merola, J.S. *Acta Crystallogr. E*, **2012**, 68, o2490.

*Hobart, D.B.; Merola, J.S. *Acta Crystallogr. E*, **2013**, 69, m261.

*Hospital**,** M.; Courseille, C.; Leroy, F.; Roques, B.P. *Biopolymers,* **1979**, 18, 1141.

*Hu, X.; Shan, Z.; Chang, Q. *Tetrahedron: Asymm*. **2012**, 23, 1327.

*Hua, H.; Cheng, M.; Li, X.; Pei, Y. *Chem. Pharm. Bull.* **2002**, 50, 1393.

*Hursthouse, M.B.; Karaulov, A. *CSD Communication (Private Communication)* **2003**.

*Huy, P.; Neudorfl, J.M.; Schmalz, H.G. *Org. Lett.* **2011**, 13, 216.

*Iminov, R.T.; Tverdokhlebov, A.V.; Tolmachev, A.A.; Volovenko, Y.M.; Kostyruk, A.N.; Chernega, A.N.; Rusanov, E.B. *Heterocycles.* **2008**, 75, 1673.

*Issa, T.B.; Sayari, F.; Ghalla, H.; Benhamada, L. *J. Mol. Struct.* **2019**, 1178, 436.

*Janczak, J.; Luger, P. *Acta Crystallogr. C* **1997**, 53, 1954.

*Jenkinson, S.F.; Cowley, A.; Kato, A.; Kato, N.; Hollinshead, J.; Nash, R.J.; Booth, K.V.; Fleet, G.W.J. *Acta Crystallogr. E*. **2007**, 63, o3717.

*Jin, Z.M.; Pan, Y.J.; Hu, M.L.; Shen, L.; Li, M.C. *Cryst. Res. Technol*. **2003**, 38, 1009.

*Jones, F.T.; Palmer, K.J.; Black, D.R. *Anal. Chem*. **1953**, 25, 1929.

*Jones, G.P.; Naidu, B.P.; Paleg, L.G.; Tiekink, E.R.T. *Acta Crystallogr. C.* **1988**, 44, 1669.

*Jones, G.P.; Paleg, L.G.; Waisel, Y.; Solomon, A.; Beer, S.; Tiekink, E.R.T. *Acta Crystallogr. C*. **1995**, 51, 287.

*Kaczmarek, K.; Wojciechowski, J.; Wolf, W.M. *Acta Crystallogr. E*. **2010**, 66, o831.

*Kanao, S.; Nagashima, N. *J. Pharm. Soc. Jpn*. **1978**, 98, 293.

*Kale, S.S.; Kunjir, S.M.; Gawade, R.L.; Puranik, V.G.; Rajamohanan, P.R.; Sanjayan, G.J. *Chem. Commun*. **2014**, 50, 2886.

*Kamwaya, M.E.; Oster, O.; Bradaczek, H. *Acta Crystallogr. B.* **1981**, 37, 1564.

*Kamwaya, M.E.; Oster, O.; Bradaczek, H. *Acta Crystallogr. B.* **1981**, 37, 364.

*Kamwaya, M.E.; Oster, O.; Bradaczek, H.; Ponnuswamy, M.N.; Parthasarathy, S.; Nagaraj, R.; Balaram, P. *Acta Cryst. B*. **1982**, 38, 172.

*Karle, I.L. *Acta Crystallogr. B.* **1970**, 26, 765.

*Kartha, G.; Ashida, T.; Kakudo, M. *Acta Crystallogr. B.* **1974**, 30, 1861.

*Kaas, Sorensen, H. *Acta Chem. Scand*. **1977**, 31, 364.

*Kemp, D.S.; Curran, T.P.; Davis, W.M.; Boyd, J.G.; Muendel, C. *J. Org. Chem.* **1991**, 56, 6672.

*Kemp, D.S.; Rothman, J.H.; Curran, T.C.; Blanchard, D.E. *Tetrahedron Lett*. **1995**, 36, 3809.

*Kessler, H. Detection of hindered rotation and inversion by NMR spectroscopy. *Angew. Chem***. 1970**, 82, 237.

*Khokhar, A.R.; Xu, Q.; Al.-Baker, S.A.; Lumetta, G.J. *Inorg. Chim. Acta.* **1993**, 203, 121.

*Kim, H.; Yoo, D.; Choi, S.Y.; Chung, Y.K.; Kim, Y.G. *Tetrahedron: Asymm*. **2008**, 19, 1965.

*Kirmizibekmez, H.; Calis, I.; Perozzo, R.; Brun, R.; Donmez, A.A.; Linden, A.; Ruedi, P.; Tasdemir, D. *Planta Med.* **2004**, 70, 711.

*Klussmann, M.; White, A.J.P.; Armstrong, A.; Blackmond, D.G. *Angew. Chem. Int.* **2006**, 45, 7985.

*Kodama, K.; Hayashi, N.; Fujita, M.; Hirose, T. *RSC Advances*. **2014**, 4, 25609.

*Koetzle, T.F.; Lehmann, M.S.; Hamilton, W.C. *Acta Crystallogr. B,* **1973**, 29, 231.

*Kojima, T.; Yamane, T.; Ashida, T. *Acta Crystallogr. B*. **1978**, 34, 2896.

*Kolstoe, S.E.; Jenvey, M.C.; Purvis, A.; Light, M.E.; Thompson, D.; Hughes, P.; Pepys, M.B.; Wood, S.P. *Acta Crystallogr. D.* **2014**, 70, 2232.

*Kondratov, I.S.; Bugera, M.Y.; Cantrel, N.A.; Daniliuc, C.G.; Haufe, G. *J. Fluorine Chem*. **2018**, 211, 100.

*Koskinen, A.M.P.; Helaja, J.; Kumpulainen, E.T.T.; Koivisto, J.; Mansikkamaki, H.; Rissanen, K.; *J. Org. Chem*., **2005**, 70, 2005.

*Koyama, G. *Helv. Chim. Acta*. **1974**, 57, 2477.

*Kraatz, H.B.; Leek, D.M.; Houmam, A.; Enright, G.D.; Lusztyk, J.; Wayner, D.D.M. J. *Organomet. Chem.* **1999**, 589, 38.

*Krapcho, J.; Turk, C.; Cushman, D.W.; Powell, J.R.; DeForrest, J.M.; Spitzmiller, E.R.; Karanewsky, D.S.; Duggan, M.; Rovnyak, G.; Schwartz, J.; Natarajan, S.; Godfrey, J.D.; Ryono, D.E.; Neubeck, R.; Atwal, K.S.; Petrillo, E.W. *J. Med. Chem.* **1988**, 31, 1148.

*Krasnov, V.P.; Nizova, I.A.; Vigorov, A.Y.; Matveeva, T.V.; Levit, G.L.; Slepukhin, P.A.; Ezhnikova, M.A.; Kodess, M.I. *Eur. J. Org. Chem.* **2008**, 1802.

*Kubyshkin, V.S.; Mykhailuk, P.K.; Afonin, S.; Ulrich, A.S.; KOmarov, I.V. *Org. Lett.* **2012**, 14, 5254.

*Kumar, P.S.; Kapat, A.; Baskaran, S. *Tetrahedron Lett*., **2008**, 49, 1241.

*Kumara Swamy, K.C.; Kumaraswamy, S.; Kommana, P. *J. Am. Chem. Soc.* **2001**, 123, 12642.

*Kumberger, O.; Schmidbaur, H. *New J. Chem.* **1991**, 15, 401.

*Kumberger, O.; Riede, J.; Schmidbaur, H. *Chem. Ber.* **1992**, 125, 1829.

*Li, Y.R.; Cui, J.L.; Yu, S.Y.; Zhong, C.S.; Wang, N.; Wang, X. Chin. *J. Struct. Chem*. **2019**, 38, 1727.

*Liang, Y.F.; Chung, C.C.; Liao, D.J.; Lee, W.J.; Tu, Y.W.; Uang, B.J. *J. Org. Chem*. **2018**, 83, 10564.

*Liautard, V.; Jardel, D.; Davies, C.; Berlande, M.; Buffeteau, T.; Cavagnat, D.; Robert, F.; Vincent, J.M.; Landais, Y. *Chem. Eur. J.* **2013**, 19, 14532.

*Light, M.E. *CSD Communication*, **2019**.

*Linden, A.; Jimy, M.; Robinson, J.A. *CSD Communication* (Private Communication). **2018**.

*Lu, Y.; Nakatsuji, H.; Okumura, Y.; Yao, L.; Ishihara, K. J. AM. *Chem. Soc.* **2018**, 140, 6039.

*Liu, Z.; Hu, X.; Abramyan, A.M.; Csekei, M.; Kotschy A.; Huc, I.; Pophristic, V. *Chem. Eur. J.* **2017**, 23, 3605.

*Liu, T.; Yan, N.; Zhao, H.; Wang, Z.X.; Hu, X.G. *J. Fluorine Chem.* **2018**, 207, 18.

*Lubben, J.; Volkmann, C.; Grabowsky, S.; Edwards, A.; Morgenroth, W.; Fabbiani, F.P.A.; Sheldrick, G.M.; Dittrich, B. *Acta Crystallogr. A,* **2014**, 70, 309.

*Luparia, M.; Oliveira, M.T.; Audisio, D.; Frebault, F.; Goddard, R.; Maulide, N. *Angew. Chem.* **2011**, 50, 12631.

*Macias, B,; Villa, M.V.; Chicote, E.; Martin-Velasco, S.; Castineiras, A.; Borras, J. *Polyhedron.* **2002**, 21, 1899.

*Marchand, A.P.; Watson, W.H. *CSD Communication.* **2006**.

*Marsch, M.; Weiher, F.; Geyer, A. *CSD Communication.* **2019**.

*Marques, M.; Braga, W.; Sqiueira, J.; Prado, L.; Rocha, H.; Ferreira, F.; Ferreira, G.; Resende, J.A.L.C. *CSD Communication* (Private Communication). **2019**.

*Martin, S.F.; Bur, S.K. Vinylogous mannich reactions. Stereoselective formal synthesis of pumiliotoxin 251D. *Tetrahedron.* **1999**, 55, 8905.

*Mascitti, V.; Maurer, T.S.; Robinson, R.P.; Bian, J.; Boustany-Kari, C.M.; Brandt, T.; Collman, B.M.; Kalgutkar, A.S.; Klenotic, M.K.; Leininger, M.T.; Lowe, A.; Maguire, G.J.; Masterson, V.M.; Miao, Z.; Mukaiyama, E.; Patel, J.D.; Petterson, J.C.; Preville, C.; Samas, B.; She, L.; Sobol, Z.; Steppan, C.M.; Stevens, B.D.; Thuma, B.A.; Tugnait, M.; Zeng, D.; Zhu, T. *J. Med. Chem.* **2011**, 54, 2952.

*Matsumura, Y.; Ogura, K.; Kouchi, Y.; Iwasaki, F.; Onomura, O. *Org. Lett.* **2006**, 8, 3789.

*Mattson, A.; Boutelje, J.; Csoregh, I.; Hjmarsson, M.; Jacobsson, U.; Lindback, M.; Norin, T.; Szmulik, P.; Hult, K. *Biorog. Med. Chem*. **1994**, 2, 501.

*Maverick, A.W.; Fronczek, F.R.; Martone, D.P.; Bradbury, J.R. *J. Coord. Chem*. **1989**, 20, 149.

*Meesakul, P.; Jaidee, W.; Richardson, C.; Andersen, R.J.; Patrick, B.O.; Willis, A.C.; Muanprasat, C.; Wang, J.; Lei, X.; Hadsadee, S.; Jungsuttiwong, S. *Photochemistry.* **2020**, 171, 112248.

*Milne, P.J.; Oliver, D.W.; Roos, H.M. *J. Crystallogr. Spectrosc. Res.* **1993**, 23, 449.

***Milne, P.J.; Oliver, D.W.; Van Rooyen, P.H.; Roos, H.M. *J. Chem. Cryst.*, **1997**, 27, 167.

*Milunovic, M.N.M.; Enuedy, E.A.; Nagy, N.V.; Kiss, T.; Trondl, R.; Jakupec, M.A.; Keppler, B.K.; Krachler, R.; Novitchi, G.; Arion, V.B. *Inorg. Chem*. **2012**, 51, 9309.

*Milunovic, M.N.M.; Dobrova, A.; Novitvhi, G.; Gligorijevic, N.; Radulovic, S.; Kozisek, J.; Rapta, P.; Enyedy, E.A.; Arion, V.B. *Eur. J. Inorg. Chem*. **2017**, 4773.

*Minacheva, L.K.; Slyudkin, O.P.; Zefirov, Y.V.; Sakharova, V.G.; Porai-Koshits, M.A. *Koord. Khim*. **1989**, 15, 825.

*Mitsui, Y.; Tsuboi, M.; Itaka, Y. *Acta Crystallogr. B.* **1969**, 25, 2182.

*Moggach, S.A.; Parsons, S.; Sawyer, L. *Acta Crystallogr. E.* **2006**, 62, o1046.

*Mohana Priyadarshini, K.; Chandramohan, A.; Anadha Babu, G.; Ramasamy, P. *Solid State Sciences*. **2014**, 28, 95.

*Mossine, V.V.; Barnes, C.L.; Mawhinney, T.P. *J. Carbohydr. Chem.* **2007**, 26, 249.

*Muramulla, S.; Arman, H.D.; Zhao, C.G.; Tiekink, E.R.T. *Acta Crystallogr. E.: Struct. Rep. Online*. **2009**, 65, o3070.

*Mukherjee, R.; McCaddon, A.; Smith. C.A.; Brasch, N.E. *Inorg. Chem.* **2009**, 48, 9526.

*Myasnyanko, I.N.; Lukyanenko, E.R.; Rybakov, V.B.; Kurkin, A.V. *CSD Communication (Private Communication*). **2015**.

*Mykhailiuk, P.K.; Shishkina, S.V.; Shishkin, O.V.; Zaporozhets, O.A.; Komarov, I.V. *Tetrahedron*. **2011**, 67, 3091.

*Mykhailiuk, P.K.; Kubyshkin, V.; Bach, T.; Budisa, N. *J. Org. Chem*. **2017**, 82, 8831.

*Nair, C.M.K.; Nagaraj, R.; Ramaprasad, S.; Balaram, P.; Vijayan, M. *Acta Crystallogr. B.* **1981**, 37, 597.

*Subha Nandhini, M.; Krishnakumar, R.V.; Natarajan, S. *Acta Crystallogr. C.* **2001**, 57, 423.

*Naveen, B.; Mudiraj, A.; Khamushavalli, G.; Babu, P.P.; Nagarajan, R. *Eur. J. Med. Chem.* **2016**, 113, 201, 113.

*Nikiforovich, G.V.; Kover, K.E.; Kolodziej, S.A.; Nock, B.; George, C.; Deschamps, J.R.; Flippen-Anderson, J.L.; Marshall, G.R. *J. Am. Chem. Soc.* **1996**, 118, 959.

*Nomoto, K.; Takemoto, T.; Maeda, M.; In, Y.; Doi, M.; Inoue, M.; Ishida, M. *Biochem. Biophys. Res. Comm.* **1992**, 187, 325.

*Novitchi, G.; Vela, S.; Pilet, G.; Train, C.; Robert, V. *Dalton Trans.* **2017,** 46, 11154.

*Nugrahani, I.; Utami, D.; Ibrahim, S.; Nugraha, Y.P.; Uekusa, H. *Eur. J. Pharm Sci.* **2018**, 117, 168.

*Odame, F.; Hoste, E.C.; Betz, R.; Loob, K.; Tshentu, Z.R. *J. Mol. Struct.* **2015**, 38, 1099.

*Oliver, D.W.; Roos, H.M.; Milne, P.J. *J. Chem. Cryst.* **1995**, 25, 85.

*O`Neil, I.A.; Milner, N.D.; Barkley, J.V.; Low, C.M.R.; Kalindjian, S.B. *Synlett.* **1995**, 617.

*Oszbach, G.; Szabo, D.; Argay, G.; Kalman, A.; Neszmelyi, A. *J. Chem. Soc., Perkin Trans. 2.* 1983, 447.

*Panda, M.K.; Runcevski, T.; Husain, A.; Dinnebier, R.E.; Naumov, P. **2015**, 137, 1895.

*Pandey, A.K.; Naduthambi, D.; Thomas, K.M.; Zondlo, N.J. *J. Am. Chem. Soc.* **2014**, 135(11), 4333.

*Pandiarajan, S.; Sridhar, B.; Rajaram, R.K. *Acta Crystallogr. E.* **2002**, 58, o74.

*Pangerl, M.; Hohlein, I.; Trauner, D. *Synlett*, **2014**, 25, 741.

*Panneerselvam, K.; Chacko, K.K. *Acta Crystallogr. C.* **1989**, 106, 45.

*Panneerselvam, K.; Chacko, K.K.; Veena, K.R. *Acta Crystallogr. C.* **1990**, 46, 81-84.

*Pascard, C.; Guilhem, J.; Vincent, M.; Remond, G.; Portevin, B.; Laubie, M. *J. Med. Chem.* **1991,** 34, 663.

*Peifer, C.; Werz, O.; Poeckel, D.; Schollmeyer, D.; Laufer, S. *Acta Crystallogr. E.* **2006**, 62, o5138.

*de S. Pereira, A.C.; Kaplan, M.A.C.; Mala, J.G.S.; Gottlieb, O.R.; Nash, R.J.; Fleet, G.; Pearce, L.; Watkin, D.J.; Scofield, A.M. *Tetrahedron*. **1991**, 47, 5637.

*Pilati, T. CSD *Communication (Private Communication).* **2007**.

*Pinalli, R.; Brancatelli, G.; Pedrini, A.; Menozzi, D.; Hernandez, D.; Ballester, P.; Geremia, S.; Dalcanale, E. A. *J. Am. Chem. Soc.* **2016**, 138, 8569.

*Poulie, C.B.M.; Alcaide, A.; Krell-Jrgensen, M.; Larsen, Y.; Astier, E.; Brjrn-Yoshimoto, W.E.; Yi, F.; Syrenne, J.T.; Storgaard, M.; Nielsen, B.; Frydenvang, K.A.; Jensen, A.A.; Hansen, K.B.; Pickering, D.S.; Bunch, L. *ACS Chemical Neuroscience*, **2019**, 10, 29890.

*Pradeille, N.; Zerbe, O.; Mohle, K.; Linden, A.; Heimgartner, *H. Chem. Biodiversity.* **2005**, 2, 1127.

*Priya, G.; Kotmale, A.S.; Chakravarty, D.; Puranik, V,G.; Rajamohana, P.R.; Sanjayan, G.J. *Org. Biomol. Chem.* **2015**, 13, 2087.

*Qu, X.; Lu, J.; Zhao, C.; Boas, J.F.; Moubaraki, B.; Murray, K.S.; Siriwardana, A.; Bond, A.M.; Martin, L.L. *Angew. Chem. Int. Ed.* **2011**, 50, 1589.

*Rajagopal, K.; Krishnakumar, R.V.; Mostad, A.; Natarajan, S. *Acta Crystallogr. E.* **2003**, 59, o277.

*Rajalakshmi, P.; Srinivasan, N.; Krishnakumar, R.V.; Razak, L.A.; Rosli, M.M. *Acta Crystallogr. E.* **2013**, 69, 01297.

*Ramanathan, C.R.; Periasamy, M. *Tetrahedron: Asymm.* **1998**, 9, 2651.

*Ranatunga, S.; Del Valle, J.R. Synthesis of Boc-protected bicycloproline. *Tetrahedron Lett.* **2009**, 50, 2464.

*Rao, S.T.; Mallikarjunan, M. *ACS.* **1974**, 243.

*Ray, J.K.; Roy, B.C.; Chinnakali, K.; Razak, I.A.; Fun, H.K. *Acta Crystallogr. C,* **1997**, 53, 1622.

*Rayner, P.J.; Smith, J.C.; Denneval, C.; O`Brien, P.O.; Clarke, P.A.; Horan, R.A. *J. Chem. Commun.* **2016**, 52, 1354.

*Razzakov, N.A.; Tashkhodzhaev, B.; Aripova, S.F.; Tozhibaev, A.A. *Khim. Prir. Soedin.* **2005**, 46.

*Renzi, P.; Overgaard, J.; Bella, M. *Org. Biomol. Chem.* **2010**, 8, 980.

*Reuter, C.; Opitz, R.; Soicke, A.; Dohmen, S.; Barone, M.; Chiha, S.; Klein, M.T.; Neudorfl, J.M.; Kuhne, R.; Schmalz, H.G. *Chem. Eur. J.* **2015**, 21, 8464.

*Rheingold, A.L. *CSD Communication (Private Communication).* **2019**.

*Rogowska, P.; Cyranski, M.K.; Sporzynski, A.; Ciesielski, A. *Tetrahedron Lett.* **2006**, 47, 1389.

*Roy, R.; Deb, J.; Jana, S.S.; Dastidar, P. *Chem. Asian J.* **2014**, 9, 3196.

*Roy, R.; Dastidar, P. *Chem. Eur. J.* **2016**, 22, 14929.

*Sagnard, I.; Sasaki, N.A.; Chiaroni, A.; Riche, C.; Potier, P. *Tetrahedron Lett.* **1995**, 36, 3149.

*Sakai, R.; Oiwa, C.; Takaishi, K.; Kamiya, H.; Tagawa, M. *Tetrahedron Lett.* **1999**, 40, 6941.

*Salonen, P.; Peuronen, A.; Sinkhonen, J.; Lehtonen, A. *Inorg. Chim. Acta.* **2019**, 489, 108.

*Sasada, K. *Acta Crystallogr.* **1961**, 14, 1305.

*Sasaki, N.A.; Pauly, R.; Fontaine, C.; Chiaroni, A.; Riche, C.; Potier, P. *Tetrahedron Lett.* **1994**, 35, 241.

*Sawka-Dobrowolska, W.; Barycki, J. *Acta Crystallogr. C.* **1989**, 45, 606.

*Schollmeyer, D. *CSD Communication (private communication).* **2016**.

*Seijas, L.E.; Delgado, G.E.; Mora, A.J.; Bahsas, A.; Briceno, A. *Acta Crystallogr. C.* **2007**, 63, o303.

*Shamala, N.; Row, T.N.G.; Venkatesan, K. *Acta Crystallogr. B.* **1976**, 32, 3267.

*Slepokura, K.; Gawlowska, M. *Acta Crystallogr. C*. **2005**, 61, o25.

*Slyudkin, O.P.; Minacheva, L.Kh.; Kerzhenstsev, M.A.; Sadikov, G.G.; Antsyshkina, A.S.; Porai-Koshits, M.A. *Koord. Khim*. **1980**, 6, 1097.

*Smith, G.D.; Pletnev, V.Z.; Duax, W.L.; Balasubramanian, T.M.; Bosshard, H.E.; Czerwinski, E.W.; Kendrick, N.E.; Mathews, F.S.; Marshall, G.E. *J. Am. Chem. Soc*. **1981**, 103, 1493.

*Song, Y.; Wang, L.Y.; Liu, F.; Li, Y.T.; Wu, Z.Y.; Yan, C.W. *CrystEngComm.* **2019**, 21, 3064.

*Sowmya, N.S.; Vidyalakshmi, S.; Sampathkrishnan, S.; Srinivasan, T.; Velmurugan, D. *Acta Crystallogr. R.* **2013**, 69, o1723.

*Sridhar Prasad, G.; Vijayan, M. *Acta Crystallogr. B.* **1993**, 49, 348.

*Stavytskyi, V.; Voskoboinik, O.; Krasovska, N.; Shabelnyk, K.; Konovalova, I.; Shishkyna, S.; Kholodniak, S.; Kovalenko, S. J. *Heterocycl. Chem.* **2020**, 57.

*Stoykova, S.A.; Linden, A.; Heimgartner, H. *Acta Crystallogr. E.* **2013**, 69, o419.

*Surov, A.O.; Voronin, A.P.; Vener, M.V.; Churakov, A.V.; Perlovich, G.L. *CrystEngComm,* **2018**, 20, 6970.

*Swaminathan, S.; Chacko, K.K. *Cryst.Struct.Commun.* **1981**, 10, 469.

*Taira, Z.; Watson, W.H. *Acta Crystallogr. B.* **1977**, 33, 3823.

*Tanaka, J.; Kozima, T.; Ashida, T.; Tanaka, N.; Kakudo, M. Benzyloxycarbonylglycyl-L-proline. *Acta Crystallogr. B.* **1977**, 33, 116.

*Tarnawski, M.; Slepokura, K.; Kulis-Orzechowska, R.; Szelepin, B. *Carbohydr. Res.* **2007**, 342, 1264.

*Tilborg, A.; Springuel, G.; Norberg, B.; Wouters, J.; Leyssens, T. *CrystEngComm*, **2013**, 15, 3341.

*Timofeeva, T.V.; Kuhn, G.H.; Nesterov, V.V.; Nesterov, V.N.; Frazier, D.O.; Penn, B.G.; Antipin, M.Y. *CrystGrowthDes*. **2003**, 3, 383.

*Tlahuext, H.; Rosas-Valdez, E.; Lopez-Cardoso, M.; Roman-Bravo, Vargas-Pindea, G.; Montiel-Palma, V.; Cotero-Villegas, A.M.; del C. Perez-Redondo, M.; Dea-Olivares, R. *J. Mol. Struct.* **2018**, 68, 1169.

*Tolmachova, N.A.; Kondratov, I.S.; Dolovanyuk, V.G.; Pridma, S.O.; Chernykh, A.V.; Daniliuc, C.G.; Haufe, G. *Chem. Commun*. **2018,** 54, 9683.

*Torbeev, V.Y.; Fumi, E.; Ebert, M.O.; Schweizer, W.B.; Hilvert, D. *Helv. Chim. Acta*, **2012**, 95, 2411.

*Toscano, R.A.; Enriquez, R.G.; Reynolds, W.F.; Magos, G.A.l Gnecco, D. *Acta Crystallogr. C.* **1997**, 53, 1690.

*Tumanova, N.; Tumanov, N.; Robeyns, K.; Fischer, F.; Fusaro, L.; Morelle, F.; Ban, V.; Hautier, G.; Filinchuk, Y.; Wouters, J.;. Leyssens, T.; Emmerling, F. *Cryst. Growth Des.*, **2018**, 18, 954.

*Turdybekov, K.M.; Nurmagambetov, Zh.S.; Zharylgasina, G.T.; Anaev, A.A.; Turmuchambetov, A. Zh.; Mamamdaliev, S.M.; Adekenov, S.M. *Pharmazevtischeskii Bulleten.* **2013**, 138.

*Ueki, T.; Bando, S.; Ashida, T.; Kakudo, M. *Acta Crystallogr. B.* **1971**, 27, 2219.

*Vamos, M.; Ozboya, K.; Kobayashi, Y. *Synlett.* **2007**, 1595.

*Vartak, A.P.; Johnson, R.L. *Org. Lett.* **2006**, 8, 983.

*Vasiuta, R.I.; Gorichko, M.V. *Tetrahedron Lett.* **2014**, 55, 466.

*Venkatesan, G.; Babu, G.A.; Ramasamy, P.; Chandramohan, A. *J. Mol. Struct.* **2013**, 1033, 121.

*****Venkateshan, M.; Suresh, J. *J. Mol. Struct.* **2019**, 1180, 826.

*Viossat, B.; Khodadad, P.; Roder, N.; Guillard, O. *Acta Crystallogr. C.* **1990**, 46, 894.

*Voissat, B.; Michelet, A.; Khodadad, P.; Rodier, N. *Acta Crystallogr. C.* **1990**, 46, 1733.

*Wang, W.; Gu, J.; Zou, X.; Tong, W.; Gong, H. *Tetrahedron Lett.* **2015**, 56, 2684.

*Wang, N.; Du, S.; Li, D.; Jiang, X. *Org. Lett.* **2017**, 19, 3167.

*Wasserman, H.H.; Henke, S.L.; Nakanishi, E.; Schulte, G. *J. Org. Chem.*, **1992**, 57, 2641.

*Watase, H.; Tomiie, Y.; Nitta, L. *Bull. Chem. Soc. Jpn.* **1958**, 31, 714.

*Wartchow, J.; Kruger, J.; Habermehl, G.G. *CSD Communication (Private Communication).* **2014**.

*Watson, W.H.; Galloy, J.; Steglich, W.; Klein, C. *Acta Crystallogr. C.* **1983**, 39, 1240.

*Webb, T.R.; Eigenbrot, C. *J. Org. Chem.* **1991**, 56, 3009.

*Wen, Y.; Sheng, T.; Hu, S.; Ma, X.; Tan, C.; Wang, Y.; Sun, Z.; Xue, Z.; Wu, X. *Chem. Commun.* **2013**, 49, 10644.

*Wilhelm, P.; Lewandowski, B.; Trapp, N.; Wennemers, H. *J. Am. Chem. Soc.* **2014**, 136, 15829.

*Willams, G.D.; Wade, C.E.; Clarkson, G.J.; Wills, M. *Tetrahedron: Asymm.* **2007**, 18, 664.

*Wittland, C.; Florke, U.; Risch, N. *Synthesis.* **1997**, 1291.

*Wojnarska, J.; Ostrowska, K.; Gryl, M.; Stadnicka, K.M. *Acta Crystallogr. C.* **2019**, 75, 1228.

*Wu, S.; Tinant, B.; Declercq, J.P.; van Meersche, M. *Bull. Soc. Chim. Belg*. **1987**, 96, 437.

*Xinghua, F.; Jianjian, F.; Aizong, Z.; Juan, Y.; Guangke, H.; Hongjun, Z., Huaxue, Y. *Chin.J.Org.Chem.*, **2014**, 34, 1616.

*Yadav, B.; Balasubramanian, S.; Chavan, R.B.; Thipparaboina, R.; Naidu, V.G.M.; Shastri, N.R. *Cryst. Growth Des.* **2018**, 18, 1047.

*Yapp, D.T.T.; Steed, J.W.; Houghton, P. *J. Acta Crystallogr. E.* **2002**, 58, o988.

*Yuan, J.; Cai, Z.Q.; Huang, C.J.; Xu, W.R. *Acta Crystallogr. E*, **2010**, 66, o3258.

*Zhang, J.; Lei-Lei, L.; Zou, H.H.; Chen, J.J.; Yu, K.B.; Xie, R.G. *Chin. J. Chem.* **2004**, 22, 585.

*Zhang, J.; Guo, D.S.; Wang, L.H.; Wang, Z.; Liu, Y. *Soft Matter.* **2011**, 7, 1756.

*Zhang, L.; Zhang, C.J.; Zhang, D.B.; Wen, J.; Wei-Zhao, X.; Li, Y.; Gao, K. *Tetrahedron Lett.* **2014**, 55, 1815.
